# Insufficient CXCL13 secretion in leprosy foamy macrophages attenuates lymphocyte recruitment and antimicrobial protein production

**DOI:** 10.3389/fimmu.2025.1541954

**Published:** 2025-04-08

**Authors:** Chuan Wang, Yuan Zhang, Tingting Liu, Zihao Mi, Peidian Shi, Zhenzhen Wang, Wenchao Li, Honglei Wang, Hong Liu, Furen Zhang

**Affiliations:** ^1^ Hospital for Skin Diseases, Shandong First Medical University, Jinan, Shandong, China; ^2^ Shandong Provincial Institute of Dermatology and Venereology, Shandong Academy of Medical Sciences, Jinan, Shandong, China

**Keywords:** foamy macrophage, mycobacterial infection, CXCL13, antimicrobial proteins, NLRP12

## Abstract

**Background:**

Pathogens trigger metabolic reprogramming, leading to the formation of foamy macrophages (FMs). This process provides a favorable environment for bacterial proliferation and enables bacteria to evade immune killing.

**Objective:**

To elucidate the mechanisms by which pathogens escape immune surveillance and elimination via the formation of FMs.

**Methods:**

We constructed a FM model using monocyte-derived macrophages (MDMs) that were incubated with oxidized low-density lipoprotein (oxLDL). Subsequently, we employed bulk RNA-sequencing (bulk RNA-seq) to comprehensively analyze the immune responses in MDMs and FMs against *Mycobacterium leprae* (*M. leprae*) infection in samples from 10 healthy individuals.

**Results:**

We found that CXCL13, a component of the cytokine-cytokine receptor interaction pathway, was specifically upregulated in *M. leprae* infected MDMs, when compared with *M. leprae* infected FMs. Significantly, further functional analyses revealed that *in vitro* treatment with CXCL13 could enhance the expression of CXCR5, thereby promoting lymphocyte migration and secretion of antimicrobial proteins. Additionally, NLRP12 was found to be specifically and highly expressed in the NOD-like receptor signaling pathway, which was enriched in infected FMs. In macrophages, *M. leprae* infection increased CXCL13 expression via NF-κB signal pathway. Conversely, in FMs, mycobacteria induced upregulation of CXCL13 was suppressed by NLRP12 through the inhibition of p52 factor expression.

**Conclusion:**

In conclusion, the NLRP12/NF-κB/CXCL13 axis is crucial for the immune response of FMs after mycobacterial infection. These findings contribute to a deeper understanding of the pathological mechanisms of mycobacterial infection.

## Introduction

Macrophages are indispensable cells that play pivotal roles in innate immunity. Their biological functions are intricate and exhibit remarkable plasticity. During the interaction of host and intracellular pathogens, macrophages mediate processes such as recognition, phagocytosis and elimination through autophagy, apoptosis, antigen presentation and cytokine production (1). Nevertheless, pathogens can employ multiple strategies to survive and proliferate within macrophages, including inducing metabolic reprogramming and evading lysosomal degradation (2, 3).

One of the primary consequences of metabolic reprogramming is the accumulation of lipid droplets (LDs) in macrophages, which subsequently gives rise to foamy macrophages (FMs) (4). A diverse array of pathogens, including bacteria, parasites, fungi and viruses, can induce the formation of FMs. Pathogens trigger FMs formation by modulating lipid accumulation in macrophages, such as cholesterol, cholesteryl ester, and triacylglycerides (5–7). Notably, FMs can be formed not only by infected macrophages, but also by surrounding uninfected macrophages (8, 9). Although studies have revealed that FMs biogenesis is disease-specific, it has been observed that FMs not only serve as nutrient sources, but also regulate phagocytosis, cell death and inflammatory reaction in infectious diseases (6, 10, 11). Moreover, FMs can attenuate immune functions and antimicrobial capacities of macrophages, facilitating pathogen survival, and thus emerge as potential therapeutic targets in antimicrobial treatment (12, 13). However, the underlying mechanisms by which pathogens regulate immune responses and evade immune killing in FMs through LD accumulation remain to be thoroughly explored.

*M. leprae*, the causative agent of leprosy, is an intracellular bacterium that invades macrophages and Schwann cells to ravage skin and peripheral nerves. Leprosy presents a spectrum of clinical forms correlating with the host innate and adaptive immune response, making it a robust model for studying divergent immune responses correlating with the host reaction to the pathogen. Lepromatous leprosy (LL) is a disseminated and severe form of the disease, characterized by diffuse skin lesions and the massive proliferation of *M. leprae* in FMs, which was first described by Virchow in 1863 as one of the classic hallmarks of this clinical type. Modlin et al. (14) discovered that the lipids in FMs were involved in immune response, contributing to the pathogenesis of microbial infections. Furthermore, blocking lipid accumulation reduces the bacterial survival and enhances the mycobactericidal effect of rifampin (15, 16). Additionally, FMs are also evident in lesions infected with pathogenic mycobacteria, such as *M*. *tuberculosis, M*. *avium* and *M*. *marinum*, and support the massive reproduction of pathogens (10, 17). This indicates that FMs are critical for mycobacterial persistence in the host and the pathogenesis of mycobacterial infections. Therefore, in this study, we further elucidated the mechanism by which FMs regulate the host immune response and support the survival of intracellular mycobacteria using leprosy as a model.

To characterize the immune reaction of FMs in response to *M. leprae*, we established an *in vitro* FMs model using monocyte-derived macrophages (MDMs) from healthy individuals. Bulk RNA-sequencing was conducted on FMs and MDMs after *M. leprae* infection. Differential gene and functional enrichment analysis revealed that CXCL13 was specifically upregulated in *M. leprae* infected MDMs. Subsequently, we performed a series of molecular functional tests to investigate the potential pathogenic mechanisms involving CXCL13. We found that CXCL13 treatment could promote the expression of CXCR5 and the secretion of antimicrobial proteins, potentially enhancing intracellular bacterial clearance in macrophages. Correspondingly, in FMs, upregulated NLRP12 inhibited *M. leprae* induced CXCL13 by suppressing the expression of the p52 factor in NF-κB signal pathway. Our results uncovered the pathogen immune evasion mechanisms during mycobacterial infection in FMs, providing potential prospects for the treatment of related diseases.

## Materials and methods

### Study subjects

A cohort of 10 volunteers were involved in this experiment, including 6 men and 4 women aged 25 to 35 years. These individuals were meticulously screened to exclude those with infectious or immune-related disease. The study protocol was approved by the Ethics Committee of Hospital for Skin Diseases, Shandong First Medical University, and informed consent was obtained from the participants.

### Monocyte-derived macrophage differentiation

Forty milliliters of peripheral blood mononuclear cells (PBMCs) were isolated from healthy donors via density gradient. Subsequently, monocytes were purified from the PBMCs using Human CD14+ MicroBeads (130-050-201, Miltenyi Biotec). The isolated monocytes were cultured in RPMI 1640 medium (ATCC) supplemented with 10% fetal bovine serum (FBS, Gibco), and 1% Penicillin-Streptomycin (Gibco). Macrophages were then differentiated by M-CSF (50 ng/ml, Abcam) for seven days at 37°C in 5% CO_2_.

### Foamy macrophages formation

To induce the transformation of macrophages into FMs, various concentrations of oxLDL (YB-002, Yiyuan Biotechnologies) were added to the culture and incubated for 24 hours. Confocal microscopy and flow cytometry were employed to observe and quantify the lipid content, using Bodipy 493/503 (Thermo Fisher Scientific) for staining. Briefly, black poly-d-lysine coated glass 12-well plates were used in confocal microscopy. The cells were washed twice for 5 minutes in PBS and fixed in 4% paraformaldehyde for 10 minutes at room temperature. Subsequently, non-specific binding was blocked at room temperature with 1% BSA for 30 minutes. The cells were incubated with 0.5 μg/mL Bodipy 493/503 for 15 minutes at 37°C and then washed three times rapidly. The cell nuclei were stained with DAPI for 5 minutes at room temperature, and images were captured using an LSM 980 confocal microscope. To determine the optimal concentration of oxLDL for inducing FMs, cells were stained with Bodipy 493/503 as that of confocal microscope mentioned above. They were harvested using 0.25% trypsin-EDTA, centrifugated at 300 × g at 4°C for 5 minutes, and resuspended in PBS. Data were acquired on the FACSAria Fusion flow cytometer (BD Biosciences) and analyzed with the FlowJo software (BD Biosciences).

### Phagocytosis of *M. leprae*

*M. leprae* was kindly provided by Institute of Dermatology, Chinese Academy of Medical Science and was grown in athymic (nu/nu) mouse foot pad as previously described (18). For the analysis of phagocytic capacity, macrophages were incubated with *M. leprae* labeled with PHK26 (Millipore Sigma) at a multiplicity of infection (MOI) of 10:1 for 4 hours at 37°C in 5% CO_2_ environment. The cellular lipid was stained with Bodipy 493/503 and the cells were collected for flow cytometry analysis as described above. Data acquisition and analysis were performed using the FACSAria Fusion flow cytometer and FlowJo software, respectively.

### Infection of cells with *M. leprae*

Monocytes were seeded in 6-well plates (1×10^6^ cells/well) and differentiated into MDMs and FMs. The human MDMs and FMs were then infected with *M. leprae* at a MOI of 10:1 for 24 hours at 37°C in 5% CO2. After collecting the supernatants, the cells were washed twice by PBS and collected in Trizol Reagent (Thermo Fisher Scientific, Carlsbab, CA, USA). Simultaneously, uninfected MDMs and FMs were also harvested as control. Cells were stored at -80°C until RNA sequencing.

### RNA isolation and sequencing data analysis

Total RNA was extracted using Trizol and its quality was assessed using Qutbit (Thermo). After rRNA removal, cDNA libraries were generated. Sequencing libraries were generated using VAHTSTM Total RNA-seq (H/M/R) Library Prep Kit for Illumina^®^. The libraries were sequenced as 151-bp paired-end reads using Illumina Novaseq6000. The clean reads obtained after processing using Skewer version 0.2.2 were aligned to the genome reference genome using STAR version 2.5.3a. The mapped reads were assembled into transcripts and merged using StringTie to obtain a set of unified transcripts. Then, the set of transcripts was compared to Ensembl gene annotations using gffcompare version 0.9.9c. The expression levels of all genes were quantified using StringTie version 1.3.1. Differentially expressed genes (DEGs) were selected according to false discovery rate (FDR) < 0.05 and absolute fold-change > 1.5 (|log_2_FC| = 0.585). Kyoto Encyclopedia of Genes and Genomes (KEGG) was evaluated separately for up- and downregulated DEGs with g: Profiler (http://biit.cs.ut.ee/gprofiler/gost).

### Enzyme linked immunosorbent assay

To measure the concentration of CXCL13 in the supernatants, the Human CXCL13 ELISA Kit (EK0739, Boster Biological Technology) was used according to the manufacturer’s directions. The absorbance was measured at 450 nm using the GlowMax-Multi detection system (Promega). The concentrations were determined based on the standard curve generated using GraphPad prism version 8.0 (GraphPad Software, Inc.).

### Multiple immunohistochemistry

Skin tissues were fixed in neutral buffered formalin and embedded in paraffin. Non-specific binding was blocked by 5% bovine serum albumin (BSA) blocking buffer (Solarbio) after antigen retrieval using TE buffer (pH 9.0). The slides were incubated with primary antibodies overnight at 4°C or for 2 hours at 37°C, followed by incubation with the goat anti-rat secondary antibodies. Color development was performed using a Four-color multiple fluorescent immunohistochemical staining kit (Absin) in accordance with the manufacturer’s instructions. The sections were imaged using the EVOSTM FL Auto 2 Imaging System (Thermofisher). Rabbit anti–human ADRP (1:200, 15294-1-AP, proteintech), antibody and CXCL13 (1:200, 10927-1-AP, proteintech) antibody were used.

### Real-time quantitative polymerase chain reaction

Total RNA was extracted using total RNA extraction Kit (LS1040, Promega) according to instructions. The isolated RNA was reverse transcribed into cDNA using the Reverse Transcription Kit (A2801, Promega). QPCR was performed using SYBR Green (4368706, Applied Biosystems) as the fluorescent reporter on an Applied Biosystems QuantStudio 5 PCR instrument. The qPCR conditions consisted of an initial holding stage at 95°C for 10 minutes, followed by 40 cycles of 15 seconds at 95°C, 1 minute at 60°C and a melt curve stage of 15 seconds at 95°C, 1 minute at 60°C and 15 seconds at 95°C. The primer sequences were as follows: GAPDH, forward, 5′-GTCTCCTCTGACTTCAACAGCG-3′; GAPDH, reverse, 5′- ACCACCCTGTTGCTGTAGCCAA-3′; CXCL13, forward, 5′- TATCCCTAGACGCTTCATTGATCG-3′; CXCL13, reverse, 5′- CCATTCAGCTTGAGGGTCCACA-3′; NLRP12, forward, 5′-CAGGCATGATGCTGCTTTGCGA-3′; NLRP12, reverse, 5′-AGCACAGAAGCCATCTCCTGAC-3′. GAPDH was used as an internal control. The relative abundance of the gene was calculated by using the equation 2^-(ΔΔCt)^.

### Western blot

Cells were homogenized in RIPA lysis buffer (R0020, SolarBio) supplemented with complete protease inhibitor and phosphatase inhibitor (P1049, SolarBio). Equal amounts of protein were separated by 10% sulfate-polyacrylamide gel electrophoresis (SDS-PAGE), and transferred onto a methanol-activated polyvinylidene difluoride (PVDF) membrane (0.45 μm) (Amersham Biosciences). After a 1 hour blocking period at room temperature using 5% BSA, the membranes were immunoblotted with antibodies against p100 (3017, CST), NLRP12 (PA5-21027, Invitrogen) and β-actin (HC201, Transgen) overnight at 4°C, followed by incubation with secondary antibodies at room temperature in Tris-buffered saline and Tween 20 (0.5% [v/v]). Immunoreactive proteins were detected using an Amersham Imager 600 with ECL substrate reagent (Millipore).

### Flow cytometry

One million PBMCs were cultured in a 12-well plate in 1 ml RPMI 1640 medium with 10% FBS with or without 10ng/mL CXCL13 for 24 hours at 37°C in 5% CO_2_. The cells were surface stained with CXCR5-PerCP/Cyanine5.5 (356910, BioLegend), CD3-Brilliant Violet 786 (740961, BD Biosciences), CD4-Alexa Fluor 700 (317426, BioLegend), CD8-Brilliant Violet 605 (344742, BioLegend) and CD19-Brilliant Violet 510 (363024, BioLegend). Intracellular stains were done following Fix/Perm kit (BD Biosciences) and stained with GNLY-Phycoerythrin (PE) (348004, BioLegend), GZMB-FITC (372206, BioLegend), PRF-Pacific Blue (308118, BioLegend) and TNF-α-APC/Cyanine7 (502944, BioLegend). All flow cytometry data were analyzed using FlowJo version 10.8.1. Fixable Viability Stain 780 (BD Biosciences) staining was used to gate out dead cells.

### Inhibitor treatment

THP-1 cells were differentiated into macrophages by PMA (200 ng/ml, Abcam). The cells were then pretreated with BAY 11-7085/BAY 11-7083 (HY-10257, MCE), or p38 MAPK-IN-1 (HY-12839, MCE) for 0.5 hour before *M. leprae* infection. The inhibitors were used at a final concentration of 10 μM. The cells were harvested for RNA and protein extraction at different time points.

### Small interfering RNA

Human NLRP12 siRNA (si-NLRP12) and nonspecific control siRNA (si-NC) were synthesized by GenePharma (Shanghai, China). The siRNAs were transfected into cells using Lipofectamine™ RNAiMAX transfection reagent (13778-075, Invitrogen) according to the manufacturer’s protocol. RNA extraction and immunoblotting assay were performed 48 hours after transfection.

### Statistics

All data were analyzed using Graphpad Prism 8.0 Software. The differences between the means of experimental groups were analyzed using the two-tailed Student *t-test*. Wilcoxon signed rank test was used for flow cytometry results. A P < 0.05 was considered statistically significant.

## Results

### High lipid-laden FMs showed enhanced phagocytic ability

**Figure 1:**
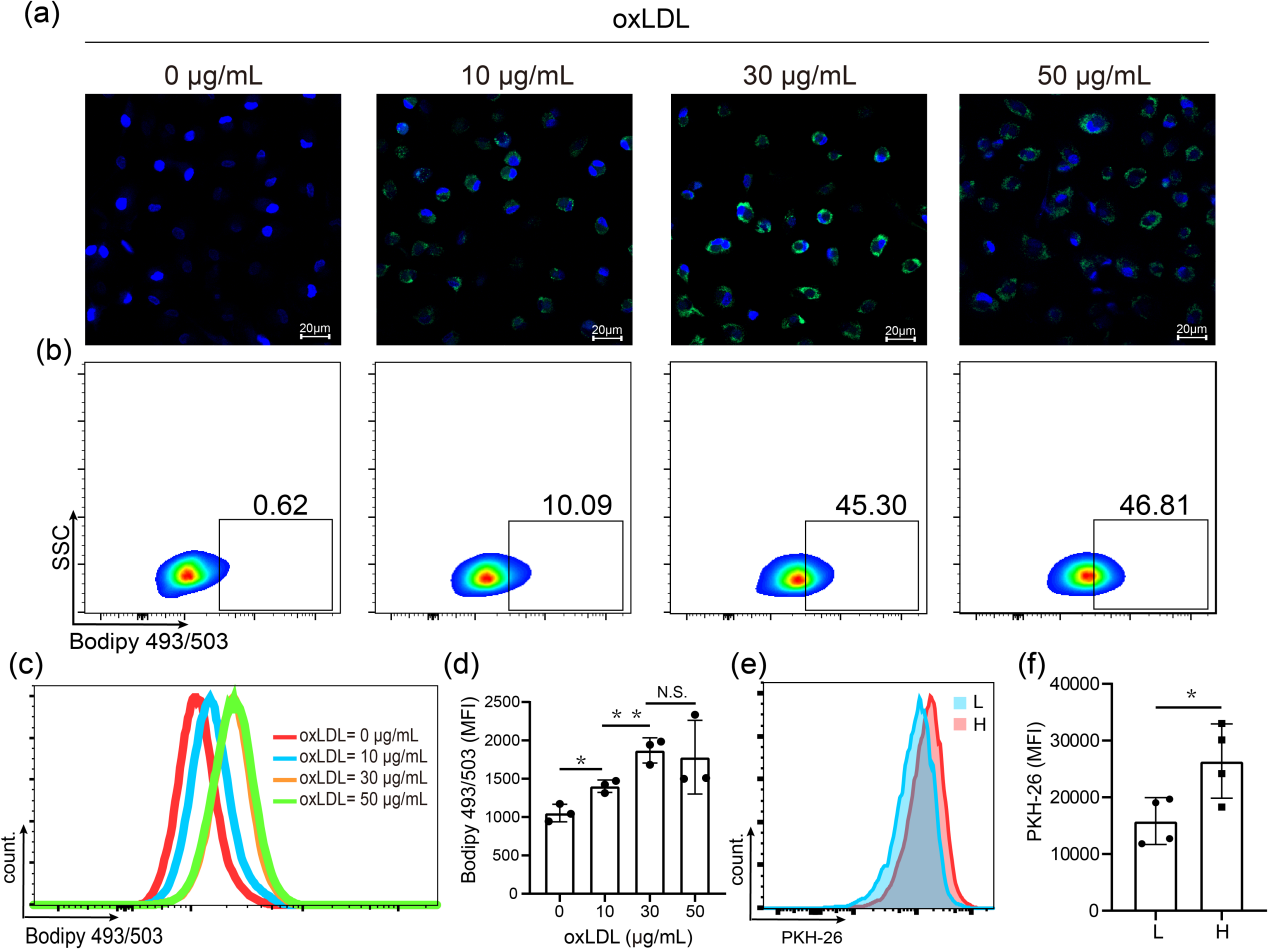
OxLDL-induced high lipid loaden foamy macrophages showed enhanced phagocytic ability. **(a)** Visualization of lipid in MDMs treated 24 h with 0, 10, 30 and 50 μg/mL oxLDL by confocal microphage. Nuclei (blue, DAPI); lipids (green, Bodipy 493/503). **(b)** Proportion of high lipid laden macrophages assessed by flow cytometry analysis. **(c)** Histogram plot for BODIPY 493/503 fluorescence. **(d)** MFIs of BODIPY 493/503-labelled lipid. **(e)** Histogram plot for PKH-26-labelled *M. leprae*. **(f)** MFIs of *M. leprae* in low (L) and high (H) lipid-laden macrophages after treated with 30 μg/mL oxLDL. DAPI: 2-(4-Amidinophenyl)-6-indolecarbamidine dihydrochloride, MFI: Mean Fluorescence Intensity. Differences between the mean of experimental groups were analyzed using the two-tailed Student *t-test*. *, ** or N.S. indicates p-value <0.05, <0.01 or no statistical significance respectively.

In previous studies of tuberculosis, experimental models of FMs and approaches for dissecting the mechanisms of lipid accumulation and consumption have been developed (19). Moreover, OxLDL, a cholesterol-rich component of lipoprotein, has been detected in LL lesions (15, 20). Consequently, we loaded human MDMs with oxLDL for FMs formation *in vitro*. We incubated MDMs with 10, 30 or 50 μg/mL oxLDL for 24 hours. Subsequently, the cells were stained with Bodipy 493/503, and confocal fluorescence microscopy and flow cytometry were performed. The results demonstrated a dose-dependent increase in both intracellular lipid levels (**Figure 1a**) and the percentage of high lipid-loaded cells (**Figure 1b**) following oxLDL treatment. Further analysis revealed no significant difference in the median fluorescence intensity (MFI) of lipids between the 30 and 50 μg/mL treatment groups (**Figures 1c, d**). Therefore, 30 μg/mL oxLDL was selected as the optimal concentration for inducing FM formation in our study.

Subsequently, we compared the phagocytic capacity of *M. leprae* in MDMs and FMs. The cells were exposed to PHK26-labelled *M.* leprae for 24 hours, and lipid staining was carried out using Bodipy 493/503. We observed that all cells exhibited fluorescence (**Supplementary Figure S1A**). OxLDL-treated macrophages were then divided into low and high lipid laden FMs (**Supplementary Figure S1B**). We then assessed the MFI of *M. leprae* in both low lipid laden and high lipid laden FMs. The findings indicated that the MFI of *M. leprae* was significantly higher in high lipid-laden FMs compared to low lipid-laden FMs (**Figures 1e, f**). In summary, oxLDL effectively induces the formation of FMs and oxLDL-induced high lipid-laden FMs display enhanced phagocytosis of *M. leprae*.

### Heterogeneity and similarity of MDMs and FMs after *M. leprae* infection

We generated human MDMs and oxLDL-induced FMs *in vitro*. Subsequently, both cells were separately infected by *M. leprae* for 24 hours *in vitro*, followed by RNA-sequencing (**Supplementary Figure S2A**). Principal component analysis (PCA) of the sequencing data revealed distinct patterns between uninfected and infected samples. Notably, there was a high degree of overlap between infected MDMs and infected FMs (**Supplementary Figure S2B**).

**Figure 2:**
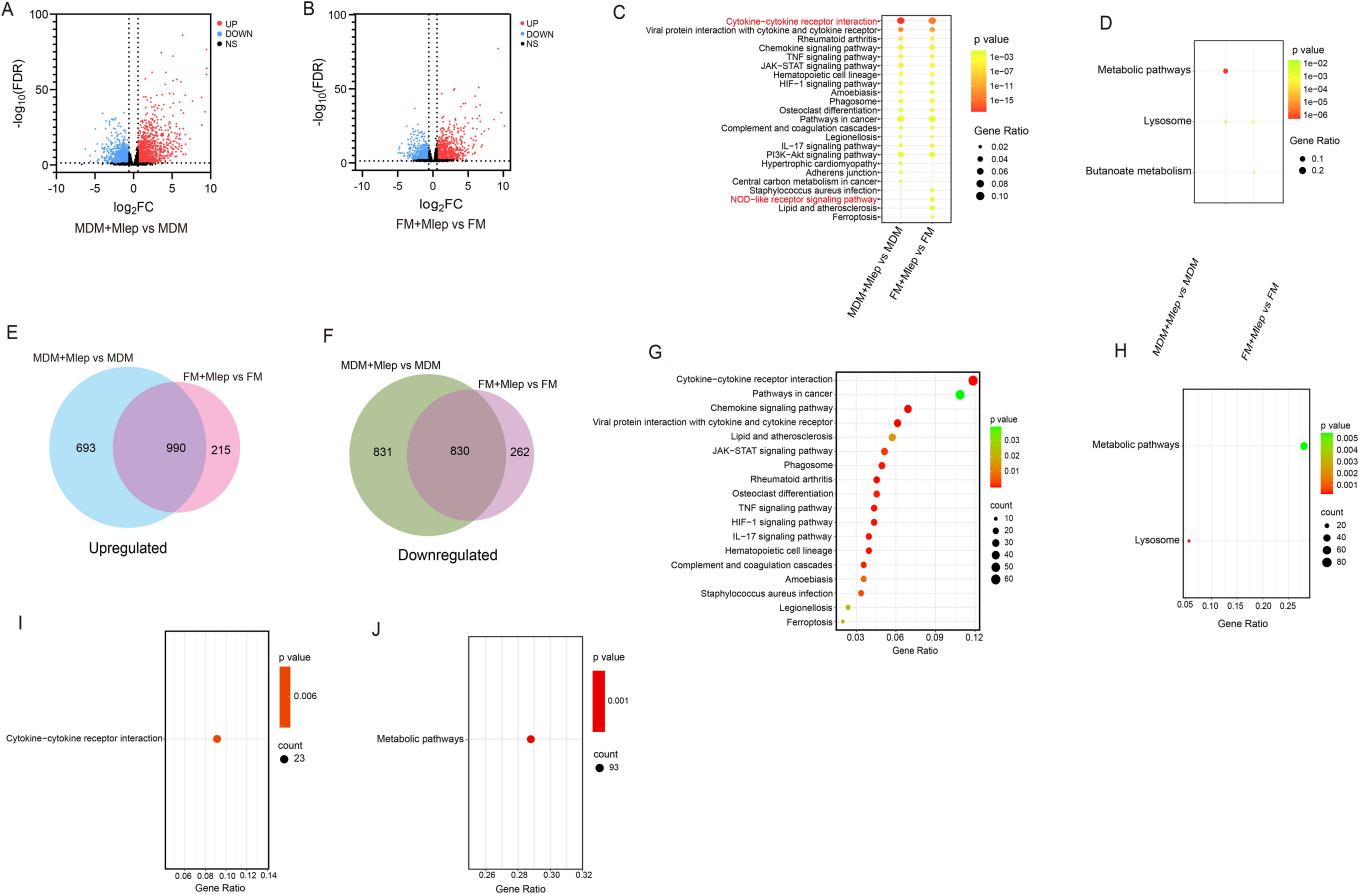
RNA-seq analysis of *M. leprae*-infected MDMs and FMs. Volcano plot showing considerably upregulated (red dots) and downregulated genes (blue dots) in *M. leprae*-infected MDMs **(A)** and FMs **(B)**. KEGG enrichment analysis of upregulated **(C)** and downregulated **(D)** DEGs in *M. leprae*-infected MDMs vs uninfected MDMs and *M. leprae*-infected FMs vs uninfected-FMs. Venn diagram of the number of upregulated **(E)** and downregulated **(F)** DEGs in *M. leprae* infected MDMs vs uninfected MDMs and *M. leprae* infected FMs vs uninfected FMs, confirming numerous shared genes (FDR<0.05 and |log_2_FC|>0.585). KEGG enrichment analysis of upregulated **(G)** and downregulated **(H)** DEGs that shared in *M. leprae*-infected MDMs vs uninfected MDMs and *M. leprae*-infected FMs vs uninfected FMs. KEGG enrichment analysis of specific upregulated **(I)** and downregulated **(J)** DEGs in *M. leprae*-infected MDMs vs uninfected MDMs. KEGG, Kyoto Encyclopedia of Genes and Genomes; DEG, Differential expressed gene.

To comprehensively analyze the similarities and differences in immune responses between MDMs and FMs, we selected the differential expressed genes (DEGs) (FDR < 0.05 and |log_2_FC| > 0.585) of MDMs and FMs after infection, respectively. 3,344 genes (1,683 upregulated and 1,661 downregulated genes, **Figure 2A**, **Supplementary Table S1**) in infected MDMs and 2,297 genes (1,207 upregulated and 1,111 downregulated genes, **Figure 2B**, **Supplementary Table S2**) in infected FMs were identified. We observed that the lipid metabolism indicators, such as PLIN2 and CD36 in uninfected FMs, were significantly increased compared to uninfected MDMs, suggesting the successful establishment of foam cell model *in vitro* (**Supplementary Figure S3**). Functional enrichment analysis of the DEGs from MDMs and FMs after infection were further performed respectively. The upregulated genes were predominantly enriched in cytokine-cytokine receptor interaction pathway (KEGG:04060, **Figure 2C**). Concurrently, the downregulated genes were mainly enriched in lysosome pathway (KEGG:04142, **Figure 2D**). Additionally, Venn diagram analysis revealed that 990 upregulated and 830 downregulated genes were shared between *M. leprae*-infected MDMs and FMs (**Figures 2E, F**). KEGG pathway analysis of these shared DEGs yielded enrichment results that were largely consistent with those of *M. leprae*-infected MDMs and FMs (**Figures 2G, H**). Collectively, these findings indicated that *M. leprae* may induce similar immune responses in both MDMs and FMs, particularly excessive inflammatory cytokine production.

The difference in bactericidal ability between MDMs and FMs may be attributed to specifically expressed genes in each cell type. Therefore, we further explored the specifically enriched pathways. In *M. leprae*-infected FMs, these pathways included staphylococcus aureus infection (KEGG:05150), NOD-like receptor signaling pathway (KEGG:04621), lipid and atherosclerosis (KEGG:05417) and ferroptosis (KEGG:04216) (**Figure 2C**). In *M. leprae*-infected MDMs, pathways such as hypertrophic cardiomyopathy (KEGG:05410), adherens junction (KEGG:04520) and central carbon metabolism in cancer (KEGG:05230) were upregulated (**Figure 2C**). Moreover, Venn diagram analysis identified specific DEGs of *M. leprae*-infected MDMs and FMs (**Figures 2E, F**). Only cytokine-cytokine receptor interaction (KEGG:04060) was enriched in specific upregulated DEGs from MDMs, and only metabolic pathway (KEGG:01100) was enriched in specific downregulated DEGs from MDMs through the KEGG pathway analysis. Unfortunately, no pathways were enriched in the DEGs from FMs (**Figures 2I, J**). These results indicate that the specific enrichment pathways from **Figures 2C, D** were not validated in the specific DEGs obtained from the Venn diagram analysis.

### CXCL13 is upregulated in infected MDMs specifically

**Figure 3:**
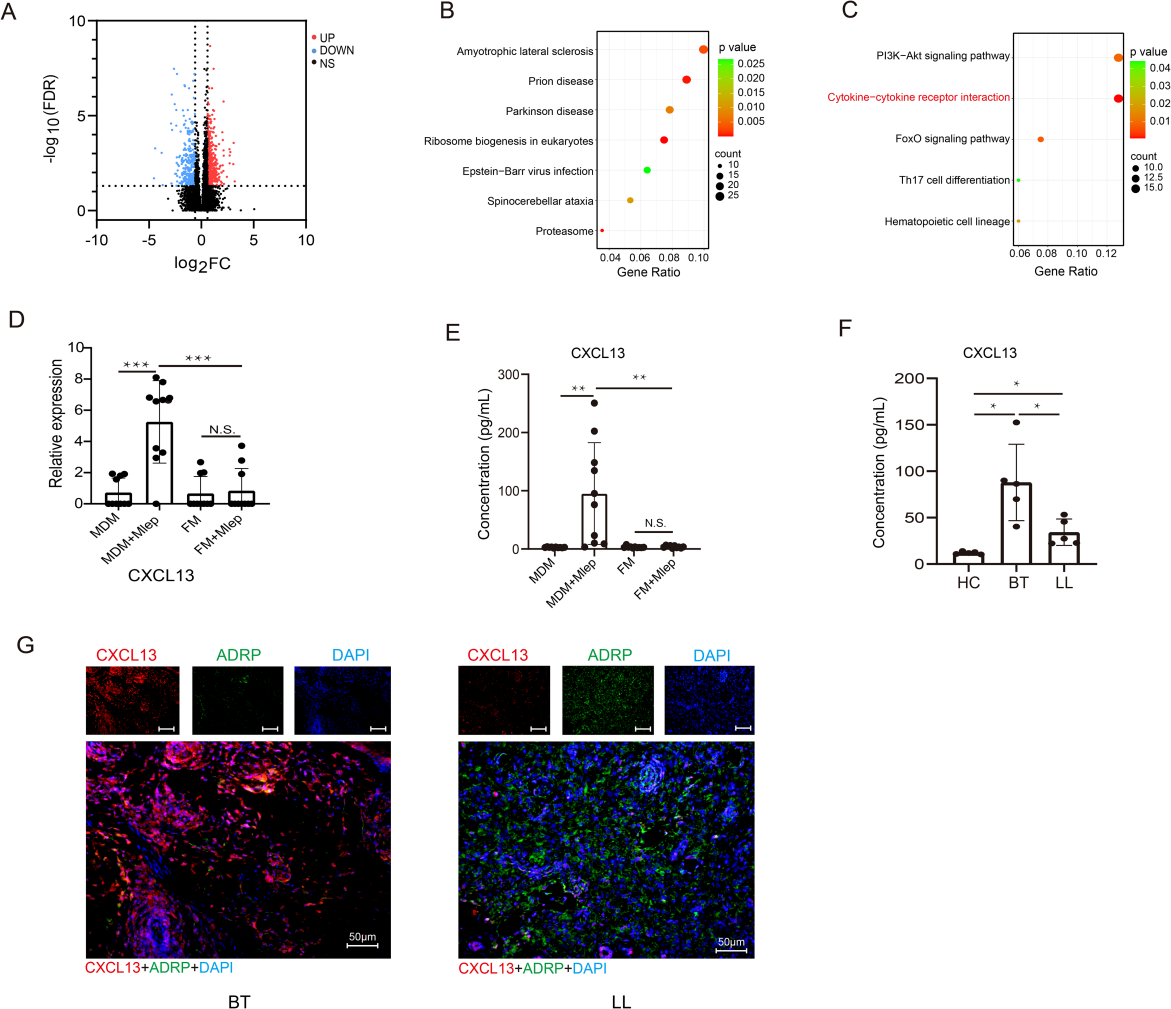
CXCL13 is upregulated in infected MDMs specifically. **(A)** Volcano plot showing considerably upregulated (red dots) and downregulated genes (blue dots) in *M. leprae*-infected FMs vs *M.* leprae-infected MDMs. KEGG enrichment analysis of upregulated **(B)** and downregulated **(C)** DEGs in *M. leprae*-infected FMs vs *M. leprae*-infected MDMs. **(D)** Expression of CXCL13 in different groups (n=10 per group). Expression abundance of CXCL13 in the supernatants of MDMs, FMs, *M. leprae*-infected MDMs and FMs (n=10 per group) **(E)**, and in the serum of HCs and leprosy patients (n=5 per group) **(F)**. **(G)** mIHC showing the co-localization of CXCL13 and FMs marker ADRP in leprosy lesions from BT and LL subtypes (n=5 per group). BT, borderline tuberculoid leprosy; LL, lepromatous leprosy; HC, healthy control; KEGG, Kyoto Encyclopedia of Genes and Genomes; DEG, Differential expressed gene. Differences between the mean of experimental groups were analyzed using the two-tailed Student *t-test*. *, **, *** or N.S. indicates p-value <0.05, <0.01, <0.001 or no statistical significance respectively.

We continued to directly compare and analyze the transcriptome profiles of MDMs and FMs after infection. Initially, we identified 593 upregulated and 342 downregulated DEGs (FDR < 0.05 and |log_2_FC| > 0.585) (**Figure 3A**, **Supplementary Table S3**). KEGG pathway analysis re-confirmed that cytokine-cytokine receptor interaction (KEGG:04060) was still enriched in downregulated DEGs (**Figures 3B, C**). Among 17 DEGs within this aforementioned pathway, CXCL13 exhibited the most significant difference in expression based on the bulk-RNA sequencing data (**Figure 3D**, **Supplementary Figure S4**). At the protein level, the concentration of CXCL13 was significantly increased in supernatant of MDMs infected by *M. leprae*. In contrast, in supernatant of FMs, *M. leprae* infection did not induce the expression of CXCL13 (**Figure 3E**).

We also examined CXCL13 levels in the serum and tissues of patients with LL and borderline tuberculoid (BT) leprosy, which is another subtype of leprosy that presents restrict bacillus growth leading to localized disease. As depicted in **Figure 3F**, compared with healthy controls, there was a significant increase of serum CXCL13 levels in leprosy patients, especially those with BT leprosy. Simultaneously, mIHC revealed that CXCL13 also significantly increased in skin lesions from BT subtype of leprosy. Moreover, in the LL subtype, an inverse correlation was observed between the expression level of CXCL13 and that of ADRP, a well-recognized marker for FMs (**Figure 3G**). These findings suggest that CXCL13 hyposecretion may be closely related to the clinical manifestations of LL leprosy.

### CXCL13 promotes the recruitment of CXCR5+ lymphocytes and secretion of antimicrobial proteins

CXCL13, a chemokine, plays a crucial role in recruiting lymphocytes via CXCR5 to mediate protective immunity (21). Given that the CXCL13/CXCR5 can dictate the homing and motility of CXCR5+ B and T cells (21–23), in this section, PBMCs from healthy individuals were stimulated using CXCL13 followed by cytometry analysis *in vitro*. The result demonstrated that CXCL13 could upregulate the expression of CXCR5 on both B and T cells (**Supplementary Figure S5**).

**Figure 4:**
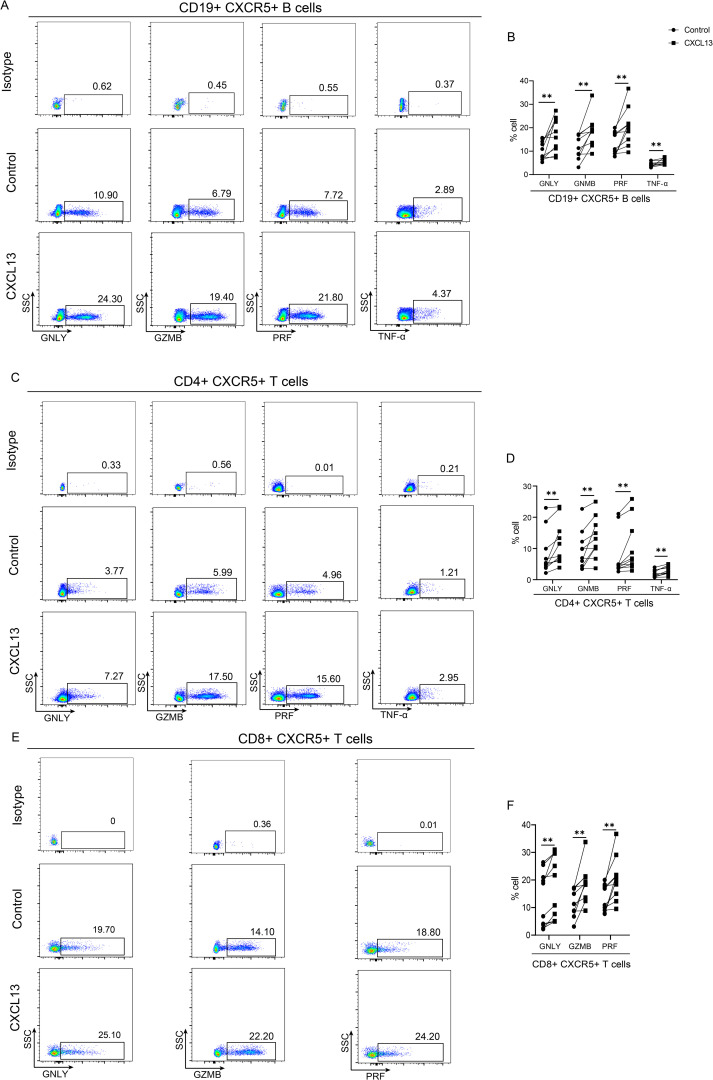
CXCL13 promotes the secretion of antimicrobial proteins. PBMCs were collected from healthy volunteers and were stimulated by 10 ng/mL CXCL13 for 24(h) The expressions of GNLY, GZMB, PRF and TNF-α in CD19+CXCR5+ B **(A)** and CD4+ CXCR5+ T cells were detected by flow cytometry **(C)**. The expressions of GNLY, GZMB and PRF in CD8+CXCR5+ T cells were detected by flow cytometry **(E)**. Each connected symbol represents paired samples from one individual donor in CD19+CXCR5+ B **(B)**, CD4+ CXCR5+ **(D)** and CD8+CXCR5+ **(F)** T cells (n=10 per group). PBMC, Peripheral blood mononuclear cell. Wilcoxon signed rank test was performed. ** indicates p-value <0.01.

To explore the functional impacts of CXCL13, the expression of bactericidal proteins, such as cytotoxic granule proteins granzyme B (GZMB), perforin (PRF) and granulysin (GNLY) and pro-inflammatory cytokines TNF-α, were examined after stimulation with CXCL13 *in vitro*. We found that CXCL13 could promote the secretion of nearly all bactericidal proteins in lymphocytes. Specifically, GNLY, GZMB, PRF and TNF-α were significantly overexpressed in CD19+ CXCR5+ B cells (**Figures 4A, B**), GNLY, GZMB, PRF and TNF-α were differentially high expressed in CD4+CXCR5+ (**Figures 4C, D**), while GNLY, GZMB and PRF were significantly overexpressed in CD8+ CXCR5+ T cells (**Figures 4E, F**). Collectively, these findings indicate that CXCL13 could enhance the expression of CXCR5 and bactericidal proteins secretion, which are associated with cytotoxic B- and T-cell responses.

### CXCL13 is suppressed by NLRP12 through the regulation of NF-κB signaling

**Figure 5:**
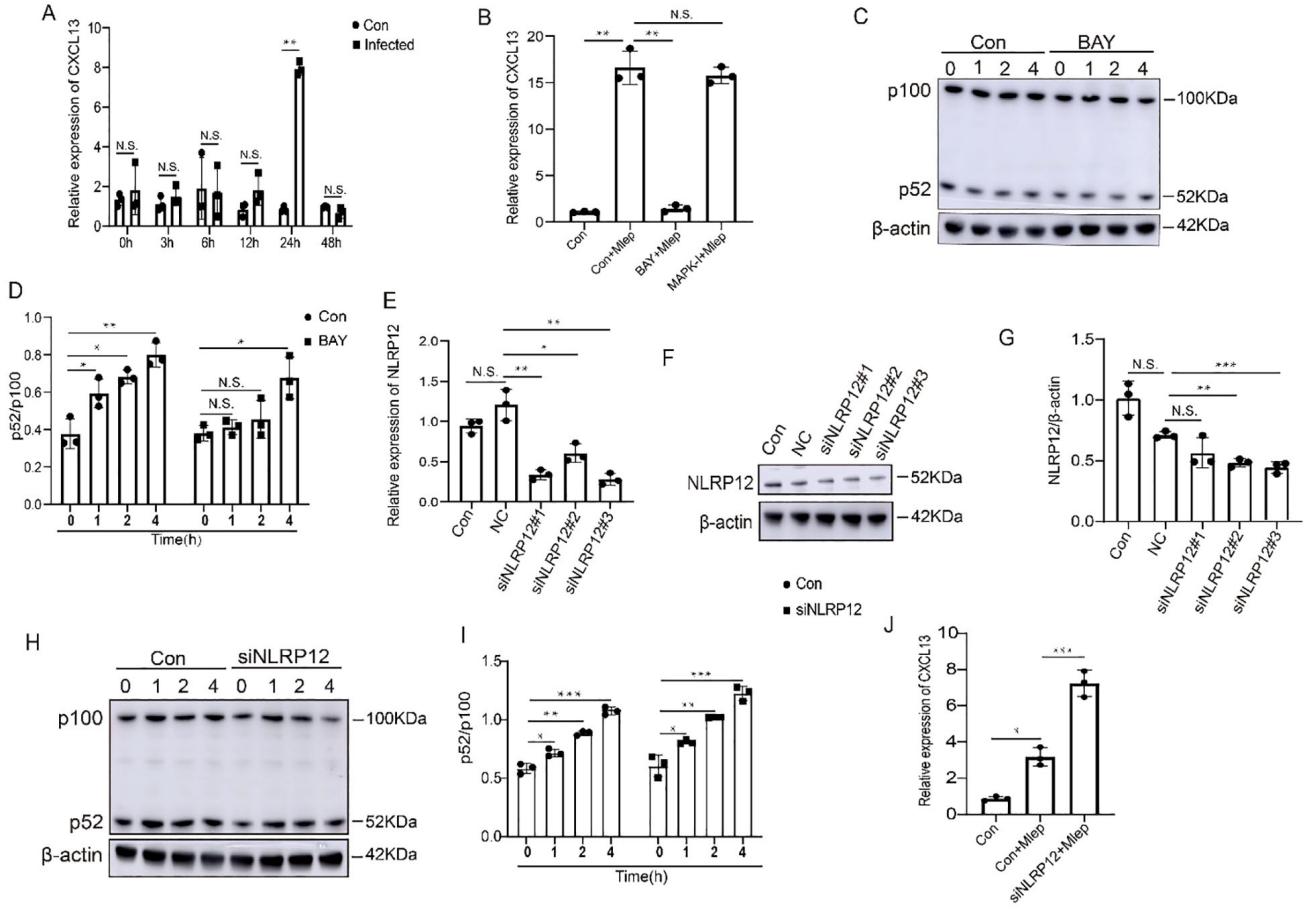
NLRP12 regulates expression of CXCL13 by non-canonical NF-κB signaling. **(A)** THP-1-derived macrophages were infected with *M. leprae* for the indicated time and CXCL13 expression was analyzed by qPCR (n=3). **(B)** THP-1-derived macrophages were infected with *M. leprae* for 24 h after pretreatment with BAY 11-7085/BAY 11-7083 or p38 MAPK-IN-1 and CXCL13 expression was analyzed by qPCR (n=3). **(C, D)** THP-1-derived macrophages were infected with *M. leprae* for the indicated time after pretreatment with BAY 11-7085/BAY 11-7083 and the p100, p52 and β-actin protein expression was assessed by WB (n=3). Three siRNAs targeting the human NLRP12 gene were designed and their efficiency was examined by qPCR **(E)** and WB **(F, G)** (n=3). Choosed siNLRP12#3 was transfected into THP-1-derived macrophages and subsequently challenged with *M. leprae*, then the p100, p52 and β-actin protein expression was assessed by WB **(H, I)**, and CXCL13 expression was analyzed by qPCR **(J)** (n=3). qPCR: quantitative polymerase chain reaction, WB: Western blotting. Con: control, NC: negative control. BAY: BAY 11-7085/BAY 11-7083. Differences between the mean of experimental groups were analyzed using the two-tailed Student *t-test*. *, **, *** or N.S. indicates p-value <0.05, <0.01, <0.001 or no statistical significance respectively.

The intrinsic mechanism of CXCL13 hyposecretion in FMs was not explored by previous reports. Given that THP-1 cells are a widely recognized and commonly used macrophage model. Previous studies (24–26) have delved into the functions of FMs derived from THP-1 cells in both infectious and non-infectious diseases. In our current study, we harnessed THP-1 cells to conduct mechanistic investigations. Our findings revealed that CXCL13 expression was significantly increased after infection for 24 hours in THP-1 cells (**Figure 5A**). It has been previously confirmed that bacterial infection can activate Toll-like receptors (TLRs) and downstream signal pathway, thereby promoting cytokines secretion in macrophages. To investigate the regulatory pathway of CXCL13, we treated THP-1 cells with inhibitors of the NF-κB pathway and the mitogen-activated protein kinase (MAPK) pathway in the presence of *M. leprae in vitro*. We found that only the IκBα kinase inhibitor (BAY 11-7085/BAY 11-7083), an inhibitor of the NF-κB pathway, could suppress the *M. leprae*-induced upregulation of CXCL13 in THP-1 cells (**Figure 5B**). Previous studies have demonstrated that non-canonical NF-κB pathway regulates the production of CXCL13 (27). The ratio of p52/p100, which are two key proteins in the non-canonical NF-κB signaling pathway, serves as an indicator of the activation state of this pathway. In *M. leprae* infected THP-1-derived macrophages, the p52/p100 ratios were significantly increased. However, in BAY 11-7085/BAY 11-7083 treated macrophages, the p52/p100 ratios at 1 and 2 hours post-infection showed no significant difference compared to those before infection. Nevertheless, when the infection time was extended to 4 hours, the ratio of these two proteins showed a significant upregulation (**Figures 5C, D**). These results indicated that non-canonical NF-κB pathway could play a key role in *M. leprae-*induced CXCL13 expression.

It has been previously reported that NLRP12 can act as a negative regulator of NF-κB signaling (28, 29). In the present study, we found that in *M. leprae*-infected FMs, NLRP12 is one of the specifically upregulated genes and was involved in the NOD-like receptor signaling pathway (**Figure 2C**). To confirm the effect of NLRP12 on the non-canonical NF-κB pathway in macrophages, we initially suppressed the expression of NLRP12 in THP-1 cells (**Figures 5E–G**). Subsequently, *M. leprae* infection can upregulate the p52/p100 ratios. Moreover, the knockdown of NLRP12 exacerbates the aforementioned upregulation (**Figures 5H, I**). We also observed that the knockdown of NLRP12 promoted the expression of CXCL13 (**Figure 5J**). Collectively, these findings indicate that NLRP12 inhibits CXCL13 expression by suppressing the activation of p52 in non-canonical NF-κB pathway.

## Discussion

Cellular metabolism serves as a critical driving force for macrophage activation during pathogen infection. Among various metabolic processes, lipid homeostasis and metabolism can be dynamically altered in response to physiological stimuli that trigger macrophage activation, thereby modulating macrophage functions (30, 31). In macrophages, the formation of FMs is a direct consequence of abnormal lipid metabolism induced by mycobacterial infections, such as those caused by *M. leprae* and *M. tuberculosis*. FMs play a pivotal role in *M. leprae* infection by facilitating bacterial persistence in the host (32, 33). In our study, we successfully constructed FMs *in vitro* and demonstrated their enhanced phagocytic ability. Subsequently, transcriptional profiles of MDMs and FMs were gained by bulk-RNA sequencing. Our findings revealed that the hyposecretion of CXCL13 in FMs cannot induce the expression of CXCR5 and the secretion of bactericidal proteins by lymphocytes, which in turn promotes the growth of *M. leprae* in LL. The results deepen the understanding of the pathological mechanisms underlying mycobacterial infection, and provide promising prospect for the development of diagnosis and treatment for related diseases.

Tissue-resident macrophages are the first line of defense against pathogens, mediating immunity through recognition of pattern recognition receptors (PRPs) and pathogen-associated molecular patterns (PAMPs) (34). Accompanied by significant reprogramming of lipid metabolism, pathogens can induce the formation of lipid-laden FMs. FMs provide energy and replication sites for pathogens and are involved in immune inflammatory responses (10, 13). Cholesterol, one of the host lipid molecules that accumulate in *M. leprae*-infected macrophages, can recruit TACO to prevent phagosome-lysosome fusion, thereby protecting mycobacteria from degradation in lysosomes (15, 35). Consistent with these findings, our results showed that high lipid-laden FMs can phagocytize more *M. leprae*. However, previous studies have reported conflicting results regarding the phagocytic capacity of FMs. Some studies have shown that the phagocytic capacity remains largely unaffected in oxLDL-treatment macrophages, while others have demonstrated a decrease in phagocytic capacity in oleic-acid-induced FMs (11, 36). These discrepancies may be attributed to the use of different inducers and varying concentrations of oxLDL in the FMs models.

CXCL13, secreted by monocyte-like and mature macrophages, plays a pathogenic role and regulate lymphoid neogenesis in human inflammatory disease (22, 37). It has been well-established that CXCL13 is a major chemoattractant for B cell and is centrally involved in the recruitment of B cells and certain T cell subsets via CXCR5 (38–40). Our results are consistent with previous reports, demonstrating that CXCL13 promotes the expression of CXCR5 *in vitro*. Moreover, the CXCL13 not only exhibits chemotactic activity depending on CXCR5 but also acts regulatory roles in chronic inflammation, infectious diseases, autoimmune disorders, neurological conditions and tumors (41, 42). In *M. tuberculosis*-infected mouse lungs, CXCL13 is not required for generation of IFN-γ responses, but is essential for the spatial arrangement of lymphocytes within granulomas, optimal activation of phagocytes, and the subsequent control of mycobacterial growth (43). An increase in CXCL13 expression leads to the accumulation of activated CD4+CXCR5+ T cells, which produce pro-inflammatory cytokines such as IFN-γ, TNF-α, and IL-2 to combat *M. tuberculosis*. The absence of CXCR5/CXCL13 axis can lead to increased susceptibility to tuberculosis (21, 44). CXCL13 can also mediate the recruitment of CD8+ CXCR5+ T cells to produce IFN-γ and IL-21, thereby controlling HBV infection (23). Cytokine like CXCL13 induce B-cell homing and clonal selection in intraportal lymphoid aggregates, which are correlate with extrahepatic clinical manifestations of HCV infection (45). Furthermore, CXCL13 is also associated with the progression of HIV-infected disease, neuroborreliosis, neurosyphilis, influenza A virus infection and SARS-CoV-2 (46). These studies underscore the significance of CXCL13 in pathogen elimination during infectious diseases. In leprosy, CXCL13 was associated with lipid metabolism, inflammatory response, and cellular immune response. It can distinguish contacts from patients and is down regulated after multidrug therapy (MDT) in multibacillary leprosy (42, 47). In the present study, we found that CXCL13 could induce the secretion of antimicrobial protein by regulating the migration of CXCR5+ lymphocytes during *M. leprae* infection. The role of B lymphocytes is rarely understood in host defense against pathogens (48, 49). Hagn et al. (50) reported that human B cells secrete active GZMB when stimulated *in vitro* by the Epstein-Barr virus (EBV). In our study, we were the first to observe that, apart from GZMB, the expression of GNLY, PRF and TNF-α increase in B cells after CXCL13 stimulation. Furthermore, it has been reported that both CD4+ cytotoxic T lymphocytes (CTLs) and CD8+ CTLs expressing GNLY, GZMB, PRF proteins exhibit antimicrobial activity against intracellular bacteria and in HIV-1 infection (51, 52). Collectively, macrophages-derived CXCL13 may facilitate the accumulation of B, CD4+T and CD8+T cells and induce the secretion of bactericidal proteins, thereby promoting the clearance of mycobacteria.

Macrophages recognize invading *M. leprae* through a series of cell-surface and intracellular PRPs, such as TLRs. This recognition triggers signaling pathways, including the NF-κB and MAPK pathways, leading to upregulation of CXCL13 expression (53, 54). Additionally, prior research has reported that in the absence of IL-23, the establishment of long-term immunity against tuberculosis is impaired. This impairment is attributed to the decreased expression of CXCL13 within B-cell follicles and the diminished capacity of T cells to migrate from blood vessels into the lesion (42). Previously published studies have also indicated that during tuberculosis infection, the expression of CXCL13 is regulated by multiple factors, including IL-17 (55) and the leptin receptor (56). In our study, we added specific pathway inhibitors into cell culture medium and found that *M. leprae* induced CXCL13 expression is dependent on non-canonical NF-κB pathway. In FMs, the inhibition of p52 might be responsible for CXCL13 hyposecretion. Evidence suggests that NLRP12 can negatively regulate noncanonical NF-κB signaling pathway in colon inflammation and tumorigenesis (29). In our study, we found that NLRP12 was specifically increased in FMs after infection. *M. leprae* induced CXCL13 expression was restored in NLRP12^-/-^ macrophages. It has been reported that NLRP12 negatively regulates noncanonical NF-κB signaling by directly interacting with and inducing the proteasomal degradation of NIK (57). We also observed that the expression p52, a downstream factor of NIK in the pathway, was reversed in NLRP12^-/-^ macrophages. In summary, the down-regulation of CXCL13 probably is due to the inhibition of noncanonical NF-κB signaling by highly expressed NLRP12 in FMs.

THP-1 cells were a widely utilized macrophage model. Wichers et al. have reported functional similarity and differences between MDMs and THP-1 macrophages. In an M1 setting, there were more similarities presented between THP-1 cells and MDMs. However, when interested in M2 models, the differences and the objectives should be more considered (58). FMs derived from aforementioned cells exhibited numerous resemblances, particularly in terms of the inflammatory response and the intracellular accumulation of cholesterol-esters (59, 60). Moreover, previous studies have successfully constructed FMs using THP-1 cells and explored their functions in infectious and non-infectious diseases. Suzuki et al. found that in THP-1 derived FMs, *M. leprae* infection could induce PPAR-δ and PPAR-γ expressions in a bacterial load-dependent manner, leading to accumulation of intracellular lipids to accommodate *M. leprae* parasitization (24). The expression of AIM enlarged FMs formation by enhancing intracellular lipid content in THP-1 cells. *M. tuberculosis*-infected AIM-expressing cells upregulated the production of Beclin 1 and LC3II, as well as enhanced mycobacterial phagosomes and LC3 co-localization (25). In THP-1 derived FMs, ox-LDL promoted TRIM64 expression, subsequently enhanced pyroptosis, and inflammation in the development of atherosclerosis (26). In our study, consistent with previous studies, THP-1 cells were used to conduct mechanistic investigations.

Our study is not without its limitations. First and foremost, although we found that CXCL13 hyposecretion is dependent on NLRP12-mediated suppression of non-canonical NF-κB signaling in FMs, the precise mechanism by which intracellular lipid accumulation affects NLRP12 expression remains to be elucidated after *M. leprae* infection. Emerging research has suggested that host-derived metabolites regulate the activation of NOD-like receptor (NLR) members (61). It will be an interesting study to reveal the association between NLRs and lipid metabolism. Moreover, the current study did not incorporate foamy dendritic cells. Salinas-Carmona et al. have reported that *Nocardia brasiliensis* could induce formation of foamy Macrophages and dendritic cells (DCs) both *in vitro* and in murine models (62). Marrow derived DCs (BMDCs) were susceptible to *M. tuberculosis* infection. Such an infection induced delayed lipid droplet accumulation and proinflammatory response (63). Existing evidence shows that dendritic cells were activated during leprosy (64), which holds great promise for future functional investigations into foamy dendritic cells. Last but not least, in our study, considering that pathogen induced FMs are more adept at reflecting the pathophysiological state of the disease, the utilization of oxLDL induced FMs could potentially be another shortcoming.

In conclusion, bulk-RNA sequencing of MDMs and FMs revealed that CXCL13 expression was specifically increased in *M. leprae* infected MDMs. Additionally, CXCL13 promoted the expression of CXCR5 and the secretion of bactericidal proteins by lymphocytes. In FMs, CXCL13 expression was suppressed by NLRP12 by inhibiting p52 factor in the non-canonical NF-κB pathway (**Supplementary Figure S6**). Overall, NLRP12/NF-κB/CXCL13 axis might serve as a potential target for enhancing host immunity in FMs. Our findings offer promising prospects for the prevention and treatment of leprosy and other mycobacterial infections.

## Data Availability

The data presented in the study are deposited in the OMIX, China National Center for Bioinformation/Beijing Institute of Genomics, Chinese Academy of Sciences (https://ngdc.cncb.ac.cn/omix/view/OMIX009614: accession number OMIX009614).
