# Supplementary material for: Insufficient CXCL13 secretion in leprosy foamy macrophages attenuates lymphocyte recruitment and antimicrobial protein production
Source: Front Immunol. 2025 Apr 8;16:1541954. doi: 10.3389/fimmu.2025.1541954 (PMC12011874; doi:10.3389/fimmu.2025.1541954)
Supplement: Supplementary file 1 [file Table1.docx]

***Supplementary Material***

Title

Insufficient CXCL13 Secretion in leprosy Foamy Macrophages Attenuates Lymphocyte Recruitment and Antimicrobial Protein Production

**Authors**:

Chuan Wang^1,2#^, Yuan Zhang^1,2#^, Tingting Liu^1,2^, Zihao Mi^1,2^, Peidian Shi^1,2^, Zhenzhen Wang^1,2^, Wenchao Li^1,2^, Yonghu Sun^1,2^, Honglei Wang^1,2^, Hong Liu^1,2*^, Furen Zhang^1,2*^,

^1^Hospital for Skin Diseases, Shandong First Medical University, 250022 Jinan, Shandong, China.

^2^Shandong Provincial Institute of Dermatology and Venereology, Shandong Academy of Medical Sciences, 250022 Jinan, Shandong, China.

**^#^** These authors contributed equally to this work.

***** **Corresponding author**:

Prof. Furen Zhang

Tel: 86-0531-87298801

Fax: 86-0531-87984734

E-mail: zhangfuren@hotmail.com

Prof. Hong Liu

Tel: 86-0531-87298870

Fax: 86-0531-87984734

E-mail: hongyue2519@hotmail.com


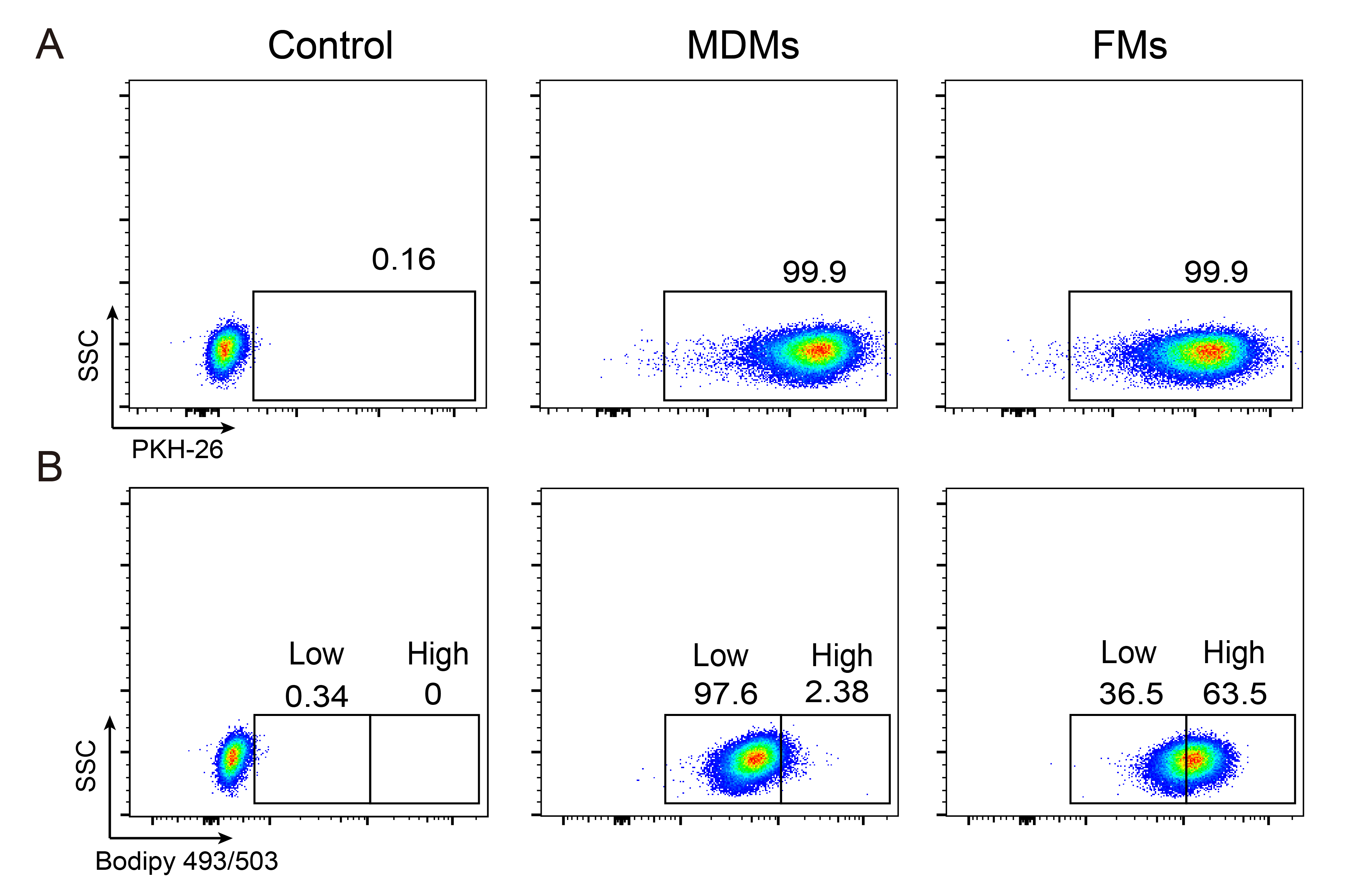


**Figure S1. Foamy macrophages phagocyte more *M. leprae*.**

**(A)** Proportion of MDMs and FMs to phagocytosis of *M. leprae* assessed by flow cytometry analysis. **(B)** Proportion of high- and low-lipid laden macrophages to phagocytosis of *M. leprae* assessed by flow cytometry analysis in MDMs and oxLDL-treated MDMs.

**
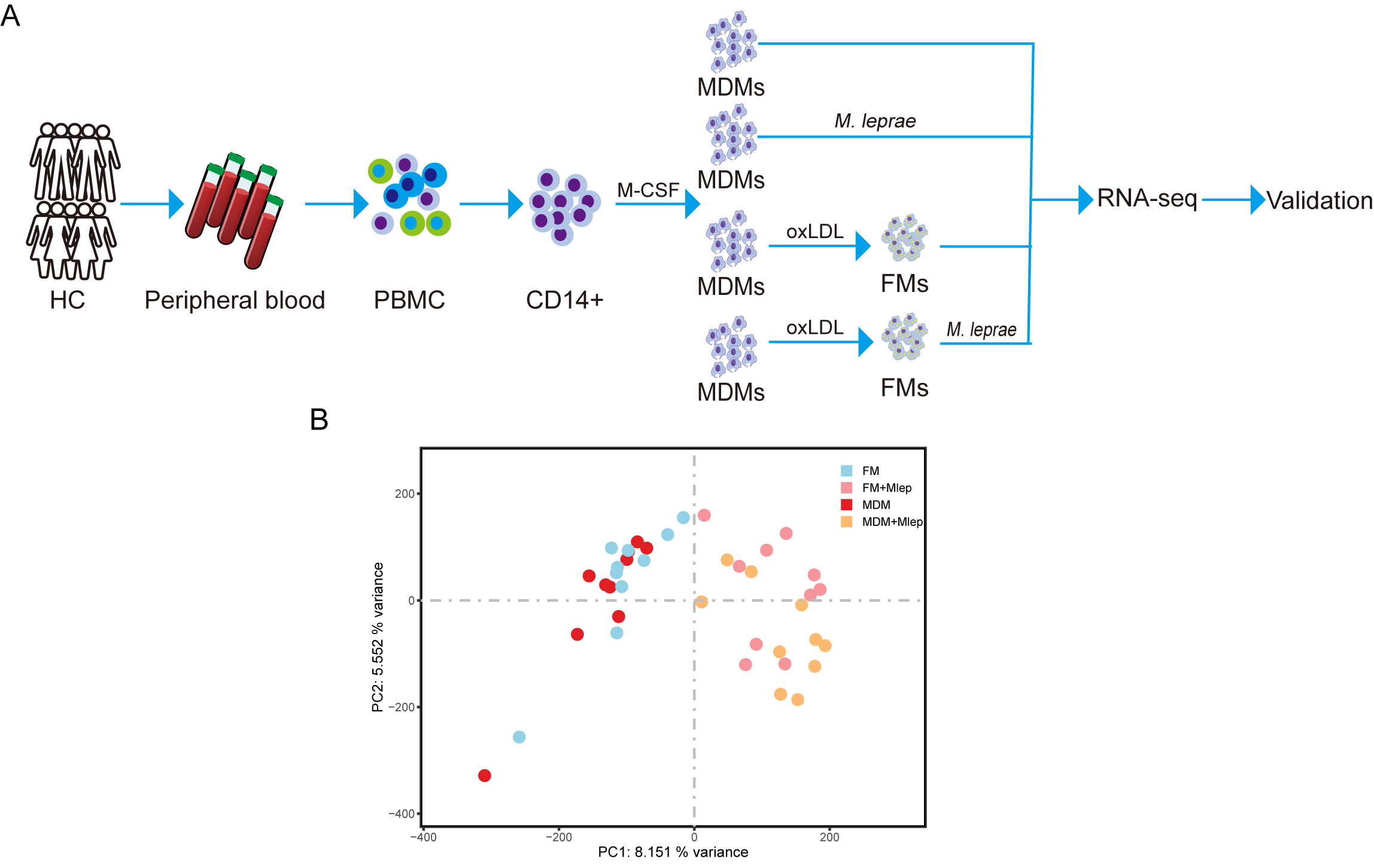
**

**Figure S2. Overall study design and general analysis of transcriptome.**

**(A)** PBMC was extracted from the venous blood of healthy volunteers, induced into MDMs and FMs, and then infected with *M. leprae* for transcriptome sequencing and validation. **(B)** Principal component analysis (PCA) of RNA-seq expression values from *M. leprae*-infected- and uninfected- MDMs and FMs. HC: healthy control, PBMC: Peripheral blood mononuclear cell.

**
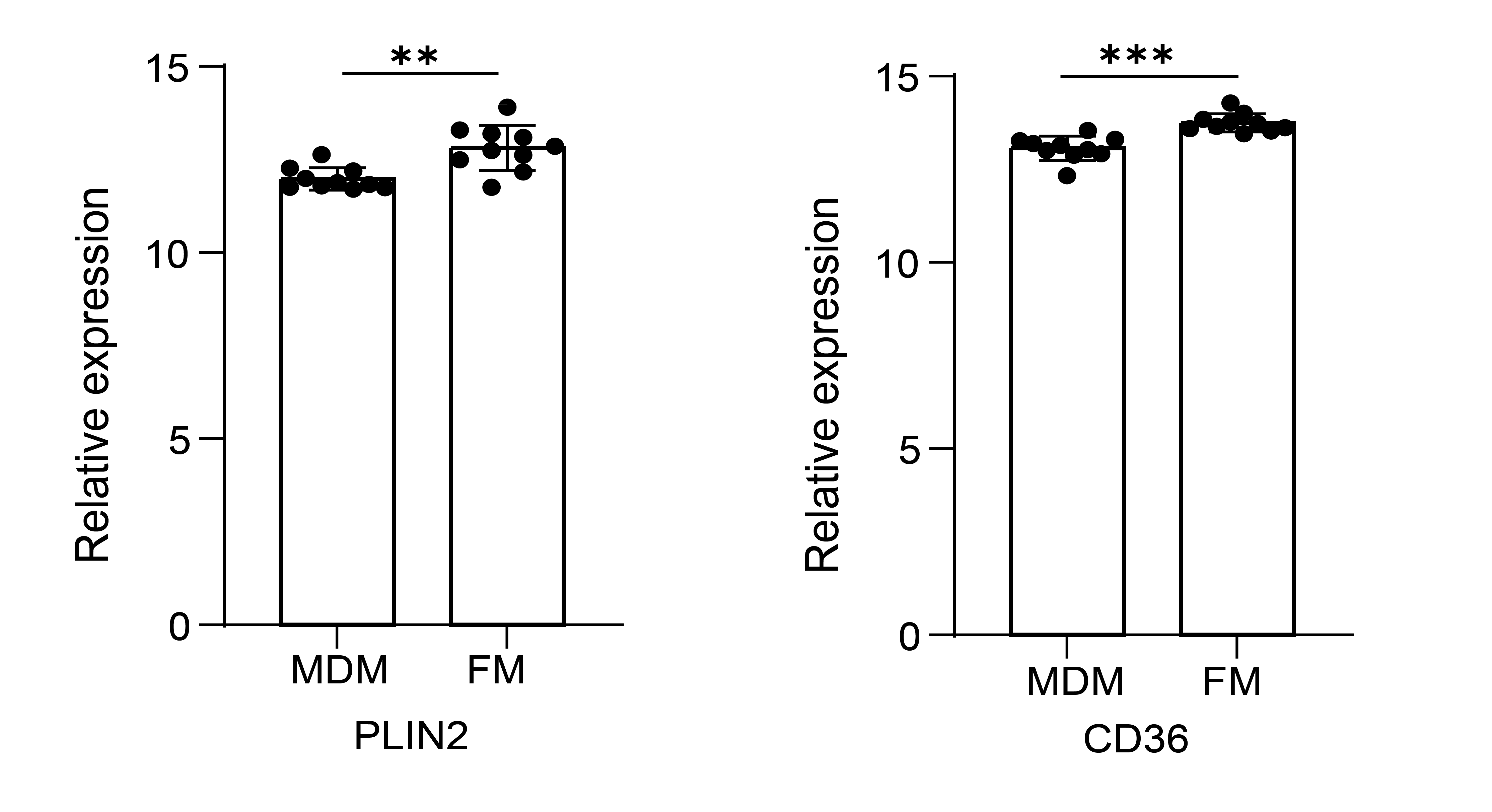
**

**Figure S3 PLIN2 and CD36 were significantly increased in uninfected foam cells**

Expression of PLIN2 and CD36 were analyzed in RNA sequencing from uninfected MDMs and FMs (n=10).Differences between the mean of experimental groups were analyzed using the two-tailed Student *t-test*. ** and *** indicates p-value <0.01, <0.001 respectively.

**
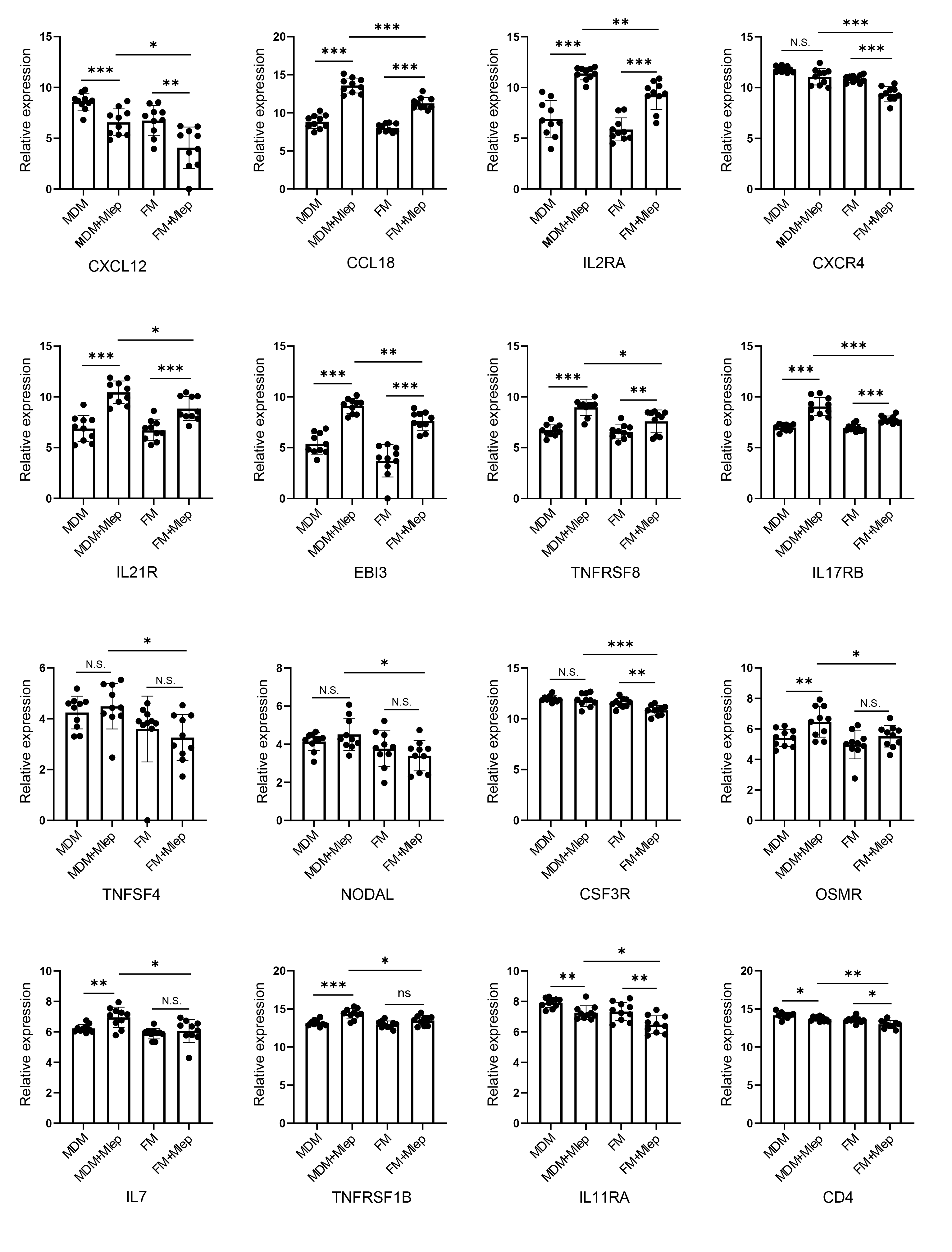
**

**Figure S4 Other 16 genes from the cytokine-cytokine receptor interaction pathway**

Expression of IL7, OSMR, TNFRSF1B, CXCL12, CCL8, IL2RA, CXCR4, IL21R, EBI3, TNFRSF8, IL17RB, TNFSF4, NODAL, CSF3R, IL11RA and CD4 in different groups were analyzed (n=10). Differences between the mean of experimental groups were analyzed using the two-tailed Student *t-test*. *, **, *** or N.S. indicates p-value <0.05, <0.01, <0.001 or no statistical significance respectively.

**
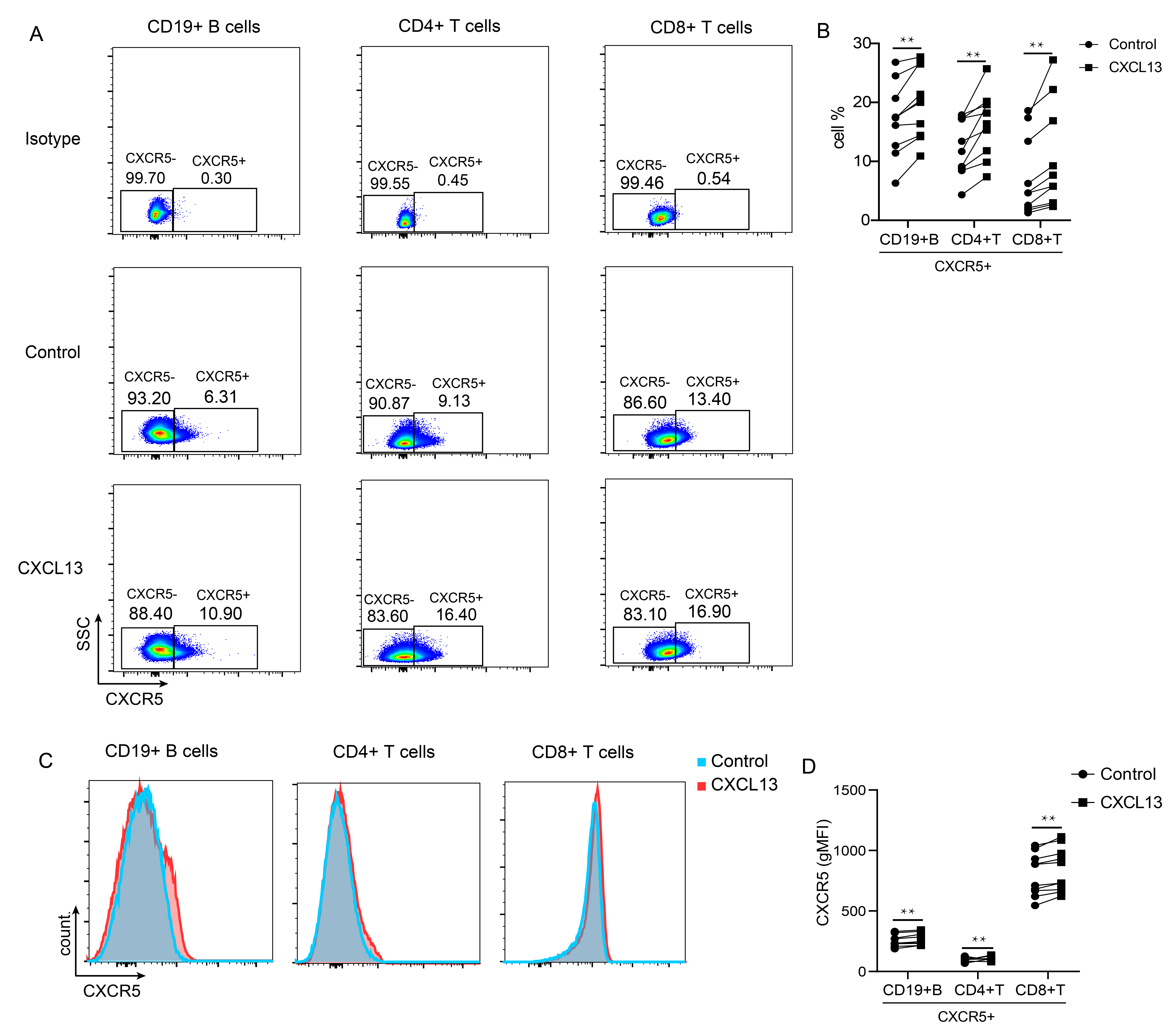
**

**Figure S5. CXCL13 induces the expression of CXCR5 in lymphocytes.**

PBMCs were collected from healthy volunteers and were stimulated by 10 ng/mL CXCL13 for 24 h. **(A)** The expression of CXCR5 in CD19+ B cells, CD4+ and CD8+ T cells was analyzed by flow cytometry. **(B)** The results were made Wilcoxon signed rank test (n=10 per group). The migratory response of PBMCs to CXCL13 treatment were detected by the transwell migration assay. **(C)** Histogram plot for CXCR5. **(D)** Geometric MFIs of CXCR5 in CD19+ B cells, CD4+ and CD8+ T cells (n=10 per group). PBMC: Peripheral blood mononuclear cell. Significance was determined by Wilcoxon signed rank test. ** indicates P < 0.01.


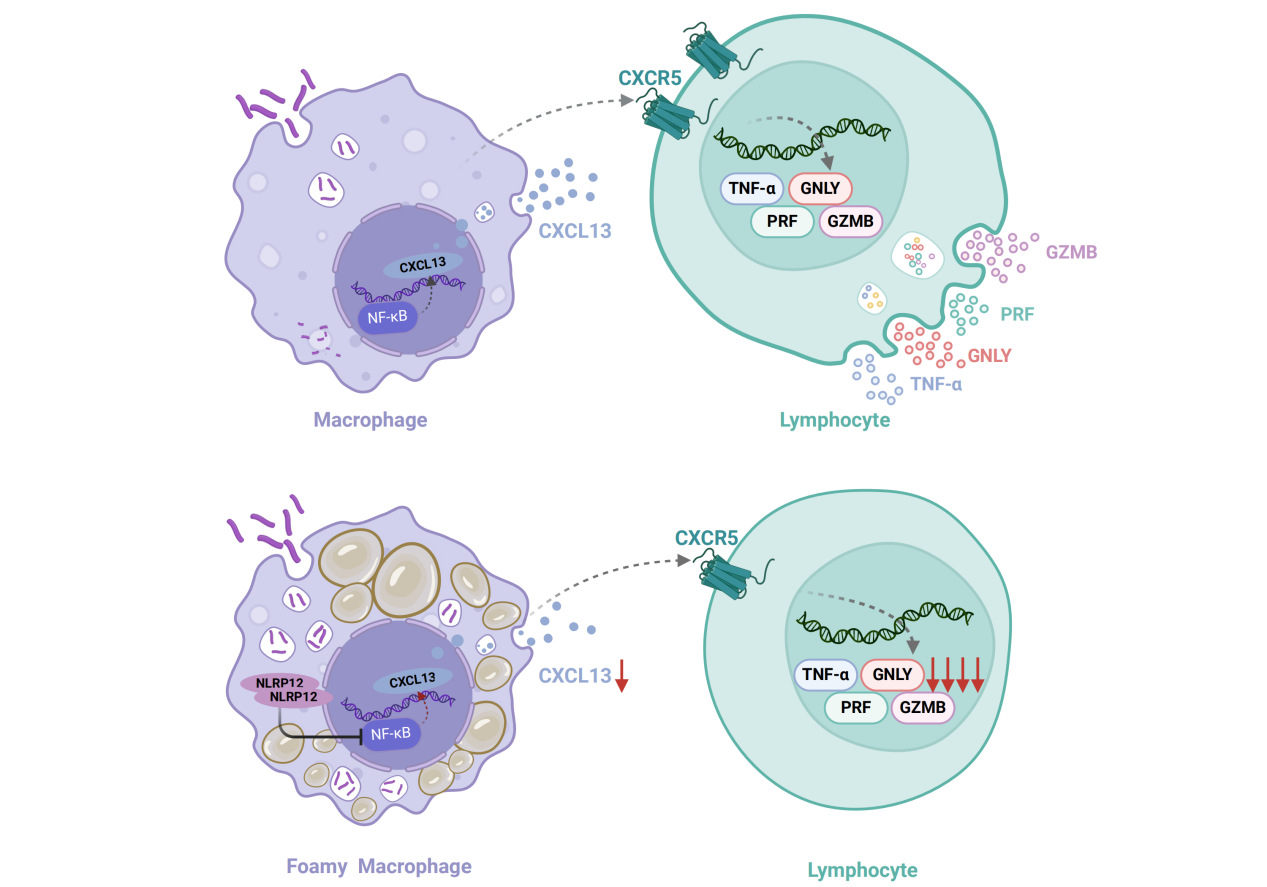


**Figure S6 A schematic mechanism model of CXCL13 suppress immune evasion of *M. leprae* via lymphocytes migration and bactericidal protein secretion**

CXCL13 could promote CXCR5+ lymphocytes migration and bactericidal protein secretion in lymphocytes. In FMs, CXCL13 expression was suppressed by NLRP12 through inhibiting p52 factor that was from non-canonical NF-κB pathway.

Table S1. DEGs in MDM+Mlep vs MDM.

| **Gene_ID** | **Symbol** | **MDM** | **MDM+Mlep** | **MDM+Mlep_vs_MDM_log2FoldChange** | **MDM+Mlep_vs_MDM_Pvalue** | **MDM+Mlep_vs_MDM_FDR** |
| --- | --- | --- | --- | --- | --- | --- |
| ENSG00000168685 | IL7R | 4.888922 | 14.39541 | 9.5064875 | 9.57E-69 | 8.38E-65 |
| ENSG00000163735 | CXCL5 | 5.1409372 | 14.603337 | 9.4623995 | 1.62E-64 | 8.85E-61 |
| ENSG00000186407 | CD300E | 4.9125052 | 14.372429 | 9.4599241 | 1.23E-81 | 2.69E-77 |
| ENSG00000205358 | MT1H | 3.5284493 | 12.802474 | 9.2740246 | 5.30E-39 | 5.27E-36 |
| ENSG00000205364 | MT1M | 0.236344 | 9.0894008 | 8.8530568 | 8.14E-30 | 3.60E-27 |
| ENSG00000125144 | MT1G | 4.1713382 | 13.013623 | 8.8422843 | 7.53E-49 | 1.57E-45 |
| ENSG00000143546 | S100A8 | 5.5854285 | 13.22416 | 7.6387316 | 3.02E-46 | 5.29E-43 |
| ENSG00000050730 | TNIP3 | 2.9025699 | 10.22551 | 7.3229399 | 3.22E-20 | 5.07E-18 |
| ENSG00000172379 | ARNT2 | 0.4149598 | 7.1782864 | 6.7633266 | 2.80E-09 | 8.36E-08 |
| ENSG00000149968 | MMP3 | 0.588826 | 7.3334816 | 6.7446556 | 1.85E-16 | 1.79E-14 |
| ENSG00000101331 | CCM2L | 0.1911951 | 6.9205014 | 6.7293063 | 3.23E-23 | 6.93E-21 |
| ENSG00000169715 | MT1E | 4.7907303 | 11.502343 | 6.7116125 | 1.72E-50 | 4.43E-47 |
| ENSG00000038427 | VCAN | 7.5369171 | 14.009291 | 6.4723736 | 4.07E-38 | 3.87E-35 |
| ENSG00000144681 | STAC | 3.8163957 | 10.277154 | 6.460758 | 1.82E-27 | 6.03E-25 |
| ENSG00000114805 | PLCH1 | 1.1956219 | 7.6385458 | 6.442924 | 3.12E-09 | 9.21E-08 |
| ENSG00000187193 | MT1X | 6.2041395 | 12.582515 | 6.3783751 | 1.68E-91 | 7.36E-87 |
| ENSG00000136960 | ENPP2 | 6.8109395 | 13.054921 | 6.2439815 | 1.29E-13 | 8.01E-12 |
| ENSG00000196611 | MMP1 | 2.0080015 | 8.2335992 | 6.2255977 | 1.46E-15 | 1.23E-13 |
| ENSG00000138316 | ADAMTS14 | 2.1321787 | 8.3035148 | 6.1713361 | 2.73E-11 | 1.16E-09 |
| ENSG00000125148 | MT2A | 6.7515415 | 12.917983 | 6.1664413 | 4.92E-40 | 5.12E-37 |
| ENSG00000112394 | SLC16A10 | 6.6526643 | 12.808869 | 6.1562051 | 1.99E-59 | 7.27E-56 |
| ENSG00000117090 | SLAMF1 | 2.5693764 | 8.6391885 | 6.0698122 | 4.65E-20 | 7.22E-18 |
| ENSG00000163221 | S100A12 | 0.4086216 | 6.4417779 | 6.0331563 | 8.31E-24 | 1.89E-21 |
| ENSG00000198417 | MT1F | 5.1158296 | 11.096638 | 5.9808081 | 9.39E-46 | 1.58E-42 |
| ENSG00000117594 | HSD11B1 | 5.1180861 | 11.098294 | 5.9802081 | 3.23E-27 | 1.03E-24 |
| ENSG00000274736 | CCL23 | 2.5577519 | 8.5291233 | 5.9713714 | 1.36E-22 | 2.74E-20 |
| ENSG00000074410 | CA12 | 2.8002722 | 8.726139 | 5.9258668 | 1.08E-16 | 1.07E-14 |
| ENSG00000163736 | PPBP | 0 | 5.9208109 | 5.9208109 | 5.38E-15 | 4.13E-13 |
| ENSG00000152766 | ANKRD22 | 0 | 5.8701112 | 5.8701112 | 2.00E-20 | 3.17E-18 |
| ENSG00000108702 | CCL1 | 1.468051 | 7.2627193 | 5.7946683 | 4.55E-13 | 2.57E-11 |
| ENSG00000099998 | GGT5 | 1.5449282 | 7.2216415 | 5.6767133 | 1.24E-23 | 2.78E-21 |
| ENSG00000152952 | PLOD2 | 4.474645 | 10.123944 | 5.6492992 | 1.64E-17 | 1.82E-15 |
| ENSG00000124731 | TREM1 | 4.9087444 | 10.513335 | 5.6045901 | 5.25E-61 | 2.30E-57 |
| ENSG00000183019 | MCEMP1 | 3.1452031 | 8.7275011 | 5.582298 | 8.77E-08 | 1.97E-06 |
| ENSG00000139178 | C1RL | 4.0692345 | 9.6514419 | 5.5822074 | 6.05E-22 | 1.11E-19 |
| ENSG00000115008 | IL1A | 2.5953992 | 8.160648 | 5.5652489 | 4.40E-14 | 2.94E-12 |
| ENSG00000170956 | CEACAM3 | 2.2084152 | 7.7637996 | 5.5553844 | 8.71E-31 | 4.28E-28 |
| ENSG00000105976 | MET | 2.1093944 | 7.6479268 | 5.5385323 | 7.52E-29 | 2.97E-26 |
| ENSG00000138135 | CH25H | 1.7847664 | 7.2989485 | 5.5141821 | 4.20E-11 | 1.73E-09 |
| ENSG00000112195 | TREML2 | 0.410019 | 5.9020062 | 5.4919873 | 1.61E-20 | 2.59E-18 |
| ENSG00000254521 | SIGLEC12 | 2.5940806 | 8.0052965 | 5.4112159 | 1.27E-07 | 2.76E-06 |
| ENSG00000105374 | NKG7 | 1.5832851 | 6.9914551 | 5.40817 | 1.04E-14 | 7.75E-13 |
| ENSG00000137801 | THBS1 | 7.7734977 | 13.14174 | 5.3682424 | 3.50E-24 | 8.37E-22 |
| ENSG00000157064 | NMNAT2 | 0.4761153 | 5.7932732 | 5.3171578 | 1.23E-18 | 1.57E-16 |
| ENSG00000133116 | KL | 1.3784094 | 6.5927361 | 5.2143267 | 6.83E-13 | 3.75E-11 |
| ENSG00000166689 | PLEKHA7 | 4.4608206 | 9.6362079 | 5.1753873 | 2.79E-51 | 8.14E-48 |
| ENSG00000184557 | SOCS3 | 6.8597921 | 12.014608 | 5.1548156 | 6.99E-45 | 1.05E-41 |
| ENSG00000275718 | CCL15 | 2.052927 | 7.1924218 | 5.1394948 | 1.85E-18 | 2.31E-16 |
| ENSG00000174837 | ADGRE1 | 7.4029677 | 12.532529 | 5.1295612 | 2.94E-60 | 1.17E-56 |
| ENSG00000151948 | GLT1D1 | 4.3304271 | 9.4385215 | 5.1080943 | 5.44E-77 | 5.95E-73 |
| ENSG00000108688 | CCL7 | 3.3924714 | 8.4952126 | 5.1027412 | 3.80E-13 | 2.19E-11 |
| ENSG00000078081 | LAMP3 | 0.4731475 | 5.5737055 | 5.100558 | 6.12E-16 | 5.50E-14 |
| ENSG00000166527 | CLEC4D | 4.4042865 | 9.4952643 | 5.0909778 | 1.92E-36 | 1.59E-33 |
| ENSG00000262406 | MMP12 | 3.2000158 | 8.2282597 | 5.0282438 | 1.83E-09 | 5.65E-08 |
| ENSG00000136244 | IL6 | 0.7248529 | 5.7512634 | 5.0264106 | 5.60E-11 | 2.24E-09 |
| ENSG00000166670 | MMP10 | 1.1497122 | 6.1373593 | 4.9876471 | 1.46E-13 | 8.99E-12 |
| ENSG00000125538 | IL1B | 6.8810781 | 11.810034 | 4.9289561 | 7.22E-25 | 1.84E-22 |
| ENSG00000124479 | NDP | 0.7963298 | 5.6594104 | 4.8630806 | 8.81E-10 | 2.89E-08 |
| ENSG00000157551 | KCNJ15 | 2.5748879 | 7.4347993 | 4.8599113 | 6.99E-08 | 1.60E-06 |
| ENSG00000137757 | CASP5 | 2.3018599 | 7.1225798 | 4.8207199 | 1.80E-14 | 1.28E-12 |
| ENSG00000131203 | IDO1 | 0.5801812 | 5.3928917 | 4.8127105 | 1.30E-18 | 1.66E-16 |
| ENSG00000173918 | C1QTNF1 | 0.4303819 | 5.232178 | 4.8017961 | 2.43E-17 | 2.64E-15 |
| ENSG00000205846 | CLEC6A | 3.6031892 | 8.3927589 | 4.7895697 | 7.88E-28 | 2.74E-25 |
| ENSG00000105855 | ITGB8 | 5.091463 | 9.8721393 | 4.7806763 | 5.96E-42 | 7.25E-39 |
| ENSG00000275385 | CCL18 | 8.834275 | 13.590635 | 4.7563595 | 2.95E-31 | 1.52E-28 |
| ENSG00000104918 | RETN | 1.6523964 | 6.405275 | 4.7528786 | 9.07E-05 | 0.0009476 |
| ENSG00000172551 | MUCL1 | 0.6686844 | 5.377268 | 4.7085836 | 9.29E-09 | 2.52E-07 |
| ENSG00000160307 | S100B | 2.132968 | 6.8374889 | 4.7045208 | 2.28E-28 | 8.53E-26 |
| ENSG00000016391 | CHDH | 4.6352905 | 9.3209067 | 4.6856162 | 1.34E-51 | 4.18E-48 |
| ENSG00000171049 | FPR2 | 3.842937 | 8.4931055 | 4.6501685 | 5.08E-32 | 2.78E-29 |
| ENSG00000124882 | EREG | 5.0430626 | 9.6722864 | 4.6292237 | 5.92E-17 | 6.16E-15 |
| ENSG00000142512 | SIGLEC10 | 8.4345455 | 13.027476 | 4.5929309 | 1.92E-23 | 4.20E-21 |
| ENSG00000106178 | CCL24 | 5.6583824 | 10.244585 | 4.5862026 | 4.92E-20 | 7.58E-18 |
| ENSG00000130413 | STK33 | 1.2741629 | 5.8319639 | 4.557801 | 8.83E-23 | 1.81E-20 |
| ENSG00000105639 | JAK3 | 6.9383863 | 11.474768 | 4.5363818 | 3.20E-17 | 3.42E-15 |
| ENSG00000132965 | ALOX5AP | 6.6302783 | 11.163863 | 4.5335845 | 2.17E-30 | 1.04E-27 |
| ENSG00000156234 | CXCL13 | 0.7210008 | 5.2526369 | 4.5316361 | 1.26E-10 | 4.75E-09 |
| ENSG00000110436 | SLC1A2 | 3.3309747 | 7.8218916 | 4.4909169 | 1.92E-26 | 5.64E-24 |
| ENSG00000163421 | PROK2 | 2.5230948 | 7.0093268 | 4.486232 | 1.26E-15 | 1.08E-13 |
| ENSG00000167105 | TMEM92 | 0 | 4.4798998 | 4.4798998 | 1.54E-19 | 2.22E-17 |
| ENSG00000185499 | MUC1 | 2.2038437 | 6.6760346 | 4.4721909 | 9.71E-22 | 1.73E-19 |
| ENSG00000134460 | IL2RA | 6.9051257 | 11.377083 | 4.4719569 | 1.14E-11 | 5.21E-10 |
| ENSG00000138356 | AOX1 | 1.7154738 | 6.1824071 | 4.4669333 | 1.00E-06 | 1.77E-05 |
| ENSG00000153976 | HS3ST3A1 | 0.2721532 | 4.7359024 | 4.4637492 | 1.03E-08 | 2.77E-07 |
| ENSG00000148344 | PTGES | 1.4258383 | 5.8790024 | 4.4531642 | 8.36E-11 | 3.23E-09 |
| ENSG00000117322 | CR2 | 0.3899861 | 4.8311979 | 4.4412117 | 2.78E-08 | 6.90E-07 |
| ENSG00000100336 | APOL4 | 1.9556507 | 6.3799715 | 4.4243208 | 3.31E-13 | 1.93E-11 |
| ENSG00000130707 | ASS1 | 1.3008077 | 5.6625367 | 4.3617291 | 4.76E-20 | 7.37E-18 |
| ENSG00000181577 | C6orf223 | 0.5313613 | 4.8649522 | 4.3335908 | 4.73E-08 | 1.13E-06 |
| ENSG00000123689 | G0S2 | 4.6691932 | 8.9962326 | 4.3270394 | 6.21E-16 | 5.55E-14 |
| ENSG00000163710 | PCOLCE2 | 3.5162235 | 7.8076681 | 4.2914446 | 1.48E-17 | 1.65E-15 |
| ENSG00000163739 | CXCL1 | 7.8119853 | 12.101748 | 4.2897625 | 2.51E-17 | 2.72E-15 |
| ENSG00000150510 | FAM124A | 3.8083537 | 8.0231117 | 4.214758 | 6.08E-14 | 3.98E-12 |
| ENSG00000173391 | OLR1 | 7.9406971 | 12.134377 | 4.1936802 | 6.65E-66 | 4.16E-62 |
| ENSG00000103196 | CRISPLD2 | 4.119808 | 8.2742326 | 4.1544246 | 4.26E-26 | 1.20E-23 |
| ENSG00000105835 | NAMPT | 10.774366 | 14.90136 | 4.1269943 | 1.82E-29 | 7.59E-27 |
| ENSG00000258947 | TUBB3 | 1.6800823 | 5.8044236 | 4.1243413 | 0.0058709 | 0.0324138 |
| ENSG00000198734 | F5 | 0.8850368 | 5.0089749 | 4.1239381 | 3.00E-08 | 7.40E-07 |
| ENSG00000135111 | TBX3 | 0.7934869 | 4.9152138 | 4.1217268 | 0.0005713 | 0.0045908 |
| ENSG00000205755 | CRLF2 | 0.6808268 | 4.7989141 | 4.1180873 | 9.37E-05 | 0.0009752 |
| ENSG00000018280 | SLC11A1 | 9.7634299 | 13.875538 | 4.1121078 | 1.10E-57 | 3.71E-54 |
| ENSG00000197769 | MAP1LC3C | 0.9575522 | 5.0414973 | 4.0839451 | 1.18E-11 | 5.36E-10 |
| ENSG00000165272 | AQP3 | 1.5059722 | 5.5540173 | 4.0480451 | 2.29E-14 | 1.61E-12 |
| ENSG00000121807 | CCR2 | 3.8934794 | 7.9290769 | 4.0355975 | 1.48E-11 | 6.56E-10 |
| ENSG00000196739 | COL27A1 | 0.6909438 | 4.7097473 | 4.0188035 | 4.06E-07 | 7.83E-06 |
| ENSG00000172724 | CCL19 | 0.7584402 | 4.7752406 | 4.0168005 | 3.41E-06 | 5.25E-05 |
| ENSG00000105419 | MEIS3 | 0.2578975 | 4.2234355 | 3.9655379 | 1.55E-11 | 6.85E-10 |
| ENSG00000142949 | PTPRF | 2.47298 | 6.4240106 | 3.9510306 | 1.05E-12 | 5.60E-11 |
| ENSG00000248099 | INSL3 | 0 | 3.9481797 | 3.9481797 | 2.76E-16 | 2.59E-14 |
| ENSG00000138821 | SLC39A8 | 10.398802 | 14.344451 | 3.9456497 | 1.00E-35 | 8.11E-33 |
| ENSG00000122641 | INHBA | 5.3264273 | 9.2548839 | 3.9284566 | 6.61E-15 | 4.99E-13 |
| ENSG00000140511 | HAPLN3 | 1.5960283 | 5.5198207 | 3.9237923 | 3.34E-06 | 5.15E-05 |
| ENSG00000185033 | SEMA4B | 6.3007083 | 10.223585 | 3.9228763 | 6.80E-44 | 9.92E-41 |
| ENSG00000120833 | SOCS2 | 3.7763353 | 7.6944438 | 3.9181085 | 1.51E-14 | 1.09E-12 |
| ENSG00000129682 | FGF13 | 0.2579069 | 4.127925 | 3.8700181 | 1.02E-10 | 3.89E-09 |
| ENSG00000162433 | AK4 | 6.1243197 | 9.9936426 | 3.8693229 | 1.16E-19 | 1.69E-17 |
| ENSG00000105889 | STEAP1B | 3.769367 | 7.6343703 | 3.8650033 | 3.87E-19 | 5.36E-17 |
| ENSG00000148483 | TMEM236 | 6.1283255 | 9.9851257 | 3.8568002 | 1.12E-23 | 2.52E-21 |
| ENSG00000105352 | CEACAM4 | 4.3223528 | 8.1619722 | 3.8396193 | 1.49E-22 | 2.99E-20 |
| ENSG00000078295 | ADCY2 | 0.3818618 | 4.2206606 | 3.8387988 | 5.70E-08 | 1.33E-06 |
| ENSG00000124875 | CXCL6 | 0.3829433 | 4.1119302 | 3.7289868 | 7.12E-08 | 1.63E-06 |
| ENSG00000161958 | FGF11 | 2.7434897 | 6.4690739 | 3.7255842 | 7.15E-11 | 2.81E-09 |
| ENSG00000171051 | FPR1 | 8.7206203 | 12.444959 | 3.7243385 | 9.37E-51 | 2.56E-47 |
| ENSG00000105246 | EBI3 | 5.3919824 | 9.1136035 | 3.7216211 | 5.42E-24 | 1.27E-21 |
| ENSG00000099985 | OSM | 5.1563063 | 8.8739373 | 3.717631 | 1.32E-22 | 2.67E-20 |
| ENSG00000159403 | C1R | 1.9736543 | 5.6732032 | 3.699549 | 1.83E-06 | 3.04E-05 |
| ENSG00000140563 | MCTP2 | 5.2211502 | 8.8870588 | 3.6659087 | 2.70E-22 | 5.16E-20 |
| ENSG00000169429 | CXCL8 | 11.001044 | 14.663504 | 3.6624601 | 2.02E-13 | 1.22E-11 |
| ENSG00000166920 | C15orf48 | 7.3745678 | 11.036986 | 3.6624187 | 6.41E-17 | 6.63E-15 |
| ENSG00000185291 | IL3RA | 6.979482 | 10.639828 | 3.6603457 | 9.56E-41 | 1.05E-37 |
| ENSG00000205362 | MT1A | 0 | 3.6421588 | 3.6421588 | 2.24E-13 | 1.34E-11 |
| ENSG00000166741 | NNMT | 0 | 3.6402213 | 3.6402213 | 1.91E-06 | 3.14E-05 |
| ENSG00000178726 | THBD | 5.2673338 | 8.9067457 | 3.6394119 | 2.68E-26 | 7.76E-24 |
| ENSG00000172986 | GXYLT2 | 1.5906523 | 5.2207511 | 3.6300988 | 1.85E-12 | 9.42E-11 |
| ENSG00000256660 | CLEC12B | 1.5774002 | 5.2063801 | 3.6289798 | 4.00E-06 | 6.05E-05 |
| ENSG00000005448 | WDR54 | 2.4412288 | 6.0686645 | 3.6274357 | 1.76E-23 | 3.88E-21 |
| ENSG00000111424 | VDR | 8.6168503 | 12.232258 | 3.615408 | 2.12E-79 | 3.10E-75 |
| ENSG00000163220 | S100A9 | 11.14132 | 14.737564 | 3.5962444 | 1.35E-33 | 9.40E-31 |
| ENSG00000129521 | EGLN3 | 2.265094 | 5.841244 | 3.57615 | 2.76E-08 | 6.88E-07 |
| ENSG00000203747 | FCGR3A | 10.907725 | 14.472303 | 3.5645789 | 3.95E-28 | 1.44E-25 |
| ENSG00000196878 | LAMB3 | 6.6663979 | 10.230901 | 3.5645027 | 9.30E-23 | 1.90E-20 |
| ENSG00000103522 | IL21R | 6.8863181 | 10.449953 | 3.5636348 | 1.74E-11 | 7.61E-10 |
| ENSG00000176105 | YES1 | 4.2972362 | 7.851957 | 3.5547208 | 1.88E-08 | 4.81E-07 |
| ENSG00000101333 | PLCB4 | 1.8071292 | 5.3615934 | 3.5544642 | 4.64E-13 | 2.61E-11 |
| ENSG00000198753 | PLXNB3 | 1.4919638 | 5.0459296 | 3.5539658 | 4.63E-11 | 1.89E-09 |
| ENSG00000258227 | CLEC5A | 7.271713 | 10.816662 | 3.544949 | 3.57E-15 | 2.83E-13 |
| ENSG00000128573 | FOXP2 | 0.9869946 | 4.5230551 | 3.5360605 | 0.0002022 | 0.0019026 |
| ENSG00000146592 | CREB5 | 5.2974155 | 8.832407 | 3.5349915 | 9.43E-13 | 5.06E-11 |
| ENSG00000259207 | ITGB3 | 6.5694984 | 10.084273 | 3.5147743 | 7.66E-12 | 3.59E-10 |
| ENSG00000120875 | DUSP4 | 3.2539365 | 6.7639669 | 3.5100305 | 3.08E-08 | 7.56E-07 |
| ENSG00000168209 | DDIT4 | 6.8147324 | 10.32365 | 3.5089172 | 7.91E-18 | 9.07E-16 |
| ENSG00000182580 | EPHB3 | 0.9589723 | 4.4678088 | 3.5088365 | 2.95E-06 | 4.63E-05 |
| ENSG00000271503 | CCL5 | 8.0244723 | 11.526981 | 3.5025088 | 2.52E-17 | 2.72E-15 |
| ENSG00000169908 | TM4SF1 | 1.1738453 | 4.6705501 | 3.4967048 | 0.0001683 | 0.0016225 |
| ENSG00000179299 | NSUN7 | 3.1759801 | 6.6724976 | 3.4965174 | 2.47E-15 | 2.02E-13 |
| ENSG00000115457 | IGFBP2 | 1.2897492 | 4.7548164 | 3.4650672 | 0.0006606 | 0.0052053 |
| ENSG00000182566 | CLEC4G | 2.0887817 | 5.537687 | 3.4489053 | 1.33E-10 | 4.99E-09 |
| ENSG00000185052 | SLC24A3 | 2.1543515 | 5.6011077 | 3.4467561 | 2.14E-13 | 1.28E-11 |
| ENSG00000174807 | CD248 | 0.5459663 | 3.9916497 | 3.4456834 | 3.05E-11 | 1.28E-09 |
| ENSG00000143847 | PPFIA4 | 4.3556483 | 7.7865835 | 3.4309351 | 2.63E-14 | 1.85E-12 |
| ENSG00000168229 | PTGDR | 2.6444507 | 6.0707483 | 3.4262976 | 3.08E-09 | 9.09E-08 |
| ENSG00000204020 | LIPN | 0.2378173 | 3.6441194 | 3.4063022 | 3.73E-11 | 1.55E-09 |
| ENSG00000165474 | GJB2 | 6.9014204 | 10.302876 | 3.4014557 | 6.13E-13 | 3.39E-11 |
| ENSG00000139194 | RBP5 | 1.219508 | 4.5981977 | 3.3786897 | 2.59E-13 | 1.53E-11 |
| ENSG00000119681 | LTBP2 | 8.4871019 | 11.858459 | 3.3713572 | 4.81E-17 | 5.05E-15 |
| ENSG00000171517 | LPAR3 | 1.4266081 | 4.7963607 | 3.3697526 | 1.96E-05 | 0.0002473 |
| ENSG00000102794 | ACOD1 | 1.1154913 | 4.4830802 | 3.367589 | 1.19E-06 | 2.05E-05 |
| ENSG00000124785 | NRN1 | 0.6759667 | 4.0354752 | 3.3595085 | 4.15E-08 | 9.97E-07 |
| ENSG00000120160 | EQTN | 0.9443765 | 4.274864 | 3.3304875 | 3.82E-08 | 9.25E-07 |
| ENSG00000125810 | CD93 | 7.3268883 | 10.64512 | 3.3182314 | 1.31E-26 | 3.92E-24 |
| ENSG00000103569 | AQP9 | 10.67253 | 13.972049 | 3.299519 | 3.03E-43 | 4.15E-40 |
| ENSG00000152760 | TCTEX1D1 | 3.5632047 | 6.8555913 | 3.2923867 | 6.13E-05 | 0.0006748 |
| ENSG00000157542 | KCNJ6 | 0.5426021 | 3.8025261 | 3.259924 | 6.02E-06 | 8.71E-05 |
| ENSG00000151136 | BTBD11 | 3.2806818 | 6.4918182 | 3.2111363 | 1.59E-12 | 8.18E-11 |
| ENSG00000244405 | ETV5 | 8.7079336 | 11.890229 | 3.1822951 | 2.31E-15 | 1.89E-13 |
| ENSG00000059804 | SLC2A3 | 9.8220647 | 12.99729 | 3.1752253 | 3.08E-29 | 1.25E-26 |
| ENSG00000169385 | RNASE2 | 4.2100484 | 7.3826226 | 3.1725742 | 5.41E-20 | 8.28E-18 |
| ENSG00000104415 | WISP1 | 1.1331374 | 4.2905701 | 3.1574327 | 0.0001883 | 0.0017862 |
| ENSG00000117394 | SLC2A1 | 5.9521547 | 9.0903067 | 3.138152 | 2.89E-22 | 5.47E-20 |
| ENSG00000198019 | FCGR1B | 6.6127437 | 9.7488682 | 3.1361245 | 1.53E-33 | 1.03E-30 |
| ENSG00000120162 | MOB3B | 8.4276535 | 11.562328 | 3.1346746 | 3.04E-41 | 3.41E-38 |
| ENSG00000162745 | OLFML2B | 11.141771 | 14.271436 | 3.1296655 | 2.01E-41 | 2.31E-38 |
| ENSG00000147852 | VLDLR | 5.0839669 | 8.2117567 | 3.1277898 | 1.33E-17 | 1.49E-15 |
| ENSG00000019169 | MARCO | 10.980129 | 14.097564 | 3.1174349 | 2.74E-12 | 1.36E-10 |
| ENSG00000188766 | SPRED3 | 1.786541 | 4.8957813 | 3.1092404 | 0.0007415 | 0.0057446 |
| ENSG00000171033 | PKIA | 2.9474368 | 6.0449166 | 3.0974798 | 1.10E-14 | 8.11E-13 |
| ENSG00000182782 | HCAR2 | 0.8708442 | 3.9613812 | 3.090537 | 1.77E-09 | 5.47E-08 |
| ENSG00000128274 | A4GALT | 2.3020105 | 5.3764932 | 3.0744827 | 3.41E-09 | 9.96E-08 |
| ENSG00000113749 | HRH2 | 8.9861276 | 12.060419 | 3.0742911 | 3.12E-31 | 1.59E-28 |
| ENSG00000138411 | HECW2 | 1.4472005 | 4.4947422 | 3.0475417 | 6.90E-08 | 1.59E-06 |
| ENSG00000197208 | SLC22A4 | 4.686256 | 7.7049223 | 3.0186663 | 2.82E-50 | 6.87E-47 |
| ENSG00000151012 | SLC7A11 | 11.149475 | 14.162801 | 3.0133268 | 2.96E-22 | 5.59E-20 |
| ENSG00000138772 | ANXA3 | 0.6271217 | 3.6398469 | 3.0127252 | 5.00E-05 | 0.0005668 |
| ENSG00000072274 | TFRC | 12.535039 | 15.545227 | 3.010188 | 5.46E-25 | 1.41E-22 |
| ENSG00000132623 | ANKEF1 | 4.2646273 | 7.2601782 | 2.9955509 | 2.00E-15 | 1.66E-13 |
| ENSG00000162747 | FCGR3B | 4.4537841 | 7.4348909 | 2.9811067 | 1.39E-10 | 5.18E-09 |
| ENSG00000070729 | CNGB1 | 1.8912247 | 4.8723228 | 2.9810981 | 6.99E-07 | 1.28E-05 |
| ENSG00000119915 | ELOVL3 | 0.4081518 | 3.3884554 | 2.9803035 | 6.90E-08 | 1.59E-06 |
| ENSG00000213949 | ITGA1 | 3.3758616 | 6.3514958 | 2.9756342 | 4.86E-24 | 1.15E-21 |
| ENSG00000101000 | PROCR | 6.2004436 | 9.1748125 | 2.9743689 | 1.84E-35 | 1.44E-32 |
| ENSG00000188778 | ADRB3 | 0 | 2.9715537 | 2.9715537 | 3.26E-09 | 9.56E-08 |
| ENSG00000170837 | GPR27 | 3.9952818 | 6.9596312 | 2.9643494 | 6.78E-11 | 2.68E-09 |
| ENSG00000168421 | RHOH | 2.1236728 | 5.0739821 | 2.9503093 | 6.60E-11 | 2.62E-09 |
| ENSG00000164683 | HEY1 | 0.5864774 | 3.5342529 | 2.9477755 | 1.51E-07 | 3.21E-06 |
| ENSG00000183762 | KREMEN1 | 5.4766306 | 8.4225307 | 2.9459 | 4.87E-13 | 2.74E-11 |
| ENSG00000142303 | ADAMTS10 | 4.5075931 | 7.4524325 | 2.9448393 | 1.42E-05 | 0.0001858 |
| ENSG00000187583 | PLEKHN1 | 1.5358841 | 4.4775897 | 2.9417056 | 6.73E-09 | 1.88E-07 |
| ENSG00000087510 | TFAP2C | 3.7013699 | 6.6411365 | 2.9397666 | 3.50E-22 | 6.58E-20 |
| ENSG00000104972 | LILRB1 | 9.9843124 | 12.912431 | 2.9281186 | 1.25E-28 | 4.81E-26 |
| ENSG00000137673 | MMP7 | 7.499089 | 10.425684 | 2.926595 | 1.52E-05 | 0.0001976 |
| ENSG00000182585 | EPGN | 1.607558 | 4.5218838 | 2.9143258 | 0.0004672 | 0.0038766 |
| ENSG00000183185 | GABRR3 | 0 | 2.911397 | 2.911397 | 1.36E-09 | 4.30E-08 |
| ENSG00000172752 | COL6A5 | 0 | 2.9065941 | 2.9065941 | 9.57E-09 | 2.60E-07 |
| ENSG00000064886 | CHI3L2 | 6.063955 | 8.9616886 | 2.8977336 | 9.73E-19 | 1.26E-16 |
| ENSG00000156206 | CFAP161 | 2.6951239 | 5.5826236 | 2.8874997 | 3.67E-15 | 2.91E-13 |
| ENSG00000139572 | GPR84 | 8.3476315 | 11.232937 | 2.8853054 | 1.03E-27 | 3.54E-25 |
| ENSG00000123610 | TNFAIP6 | 5.2942662 | 8.1774658 | 2.8831996 | 1.31E-14 | 9.58E-13 |
| ENSG00000136237 | RAPGEF5 | 1.1600655 | 4.0408305 | 2.880765 | 5.75E-06 | 8.38E-05 |
| ENSG00000196083 | IL1RAP | 7.3831909 | 10.262625 | 2.8794343 | 6.89E-23 | 1.46E-20 |
| ENSG00000169083 | AR | 2.6715444 | 5.5470819 | 2.8755375 | 3.71E-10 | 1.30E-08 |
| ENSG00000186431 | FCAR | 7.4631875 | 10.326178 | 2.8629902 | 3.42E-25 | 8.92E-23 |
| ENSG00000255330 | SOGA3 | 1.856525 | 4.7148303 | 2.8583053 | 0.0037997 | 0.0226901 |
| ENSG00000144476 | ACKR3 | 1.0486303 | 3.9055451 | 2.8569148 | 0.0001568 | 0.0015266 |
| ENSG00000163734 | CXCL3 | 8.3201922 | 11.173739 | 2.8535469 | 5.75E-23 | 1.22E-20 |
| ENSG00000198535 | C2CD4A | 0 | 2.8507333 | 2.8507333 | 0.0001894 | 0.0017956 |
| ENSG00000139567 | ACVRL1 | 6.330269 | 9.166228 | 2.835959 | 2.56E-13 | 1.51E-11 |
| ENSG00000153064 | BANK1 | 3.8519251 | 6.6849007 | 2.8329756 | 5.48E-13 | 3.05E-11 |
| ENSG00000120885 | CLU | 6.1705271 | 8.9948824 | 2.8243553 | 1.67E-11 | 7.31E-10 |
| ENSG00000198814 | GK | 10.6913 | 13.509821 | 2.8185207 | 1.67E-43 | 2.35E-40 |
| ENSG00000134532 | SOX5 | 0.1925188 | 3.0075551 | 2.8150362 | 6.54E-05 | 0.0007134 |
| ENSG00000141622 | RNF165 | 0.2558565 | 3.0614171 | 2.8055606 | 0.0006106 | 0.004856 |
| ENSG00000075426 | FOSL2 | 10.120078 | 12.912369 | 2.7922908 | 4.27E-18 | 5.12E-16 |
| ENSG00000159618 | ADGRG5 | 0.4592123 | 3.2456753 | 2.786463 | 5.54E-06 | 8.10E-05 |
| ENSG00000108950 | FAM20A | 8.9335377 | 11.714381 | 2.7808428 | 8.01E-40 | 8.15E-37 |
| ENSG00000162493 | PDPN | 8.2891202 | 11.058854 | 2.7697335 | 1.03E-16 | 1.03E-14 |
| ENSG00000182541 | LIMK2 | 9.6725481 | 12.43958 | 2.7670316 | 7.09E-37 | 6.09E-34 |
| ENSG00000058085 | LAMC2 | 0.9930435 | 3.7182962 | 2.7252527 | 3.01E-08 | 7.42E-07 |
| ENSG00000114948 | ADAM23 | 0.4495275 | 3.1719937 | 2.7224662 | 1.47E-05 | 0.0001907 |
| ENSG00000143217 | NECTIN4 | 6.6049645 | 9.3263208 | 2.7213563 | 4.53E-19 | 6.20E-17 |
| ENSG00000012779 | ALOX5 | 9.737822 | 12.458989 | 2.7211671 | 1.02E-32 | 6.19E-30 |
| ENSG00000169439 | SDC2 | 10.130482 | 12.845585 | 2.7151029 | 5.07E-32 | 2.78E-29 |
| ENSG00000143226 | FCGR2A | 12.275169 | 14.986858 | 2.7116889 | 8.75E-23 | 1.81E-20 |
| ENSG00000102265 | TIMP1 | 10.592894 | 13.302819 | 2.709925 | 2.41E-21 | 4.13E-19 |
| ENSG00000104974 | LILRA1 | 6.5073402 | 9.2148648 | 2.7075246 | 6.48E-19 | 8.59E-17 |
| ENSG00000153707 | PTPRD | 0.1672071 | 2.8613156 | 2.6941085 | 7.15E-08 | 1.63E-06 |
| ENSG00000143797 | MBOAT2 | 6.3167689 | 9.0076067 | 2.6908379 | 1.54E-17 | 1.72E-15 |
| ENSG00000165023 | DIRAS2 | 1.710601 | 4.394809 | 2.6842079 | 0.0024056 | 0.0154326 |
| ENSG00000135426 | TESPA1 | 1.1370924 | 3.8184537 | 2.6813613 | 3.14E-07 | 6.27E-06 |
| ENSG00000156265 | MAP3K7CL | 5.5075674 | 8.1874785 | 2.6799111 | 1.36E-15 | 1.15E-13 |
| ENSG00000099139 | PCSK5 | 8.1914512 | 10.854239 | 2.662788 | 2.11E-42 | 2.72E-39 |
| ENSG00000061656 | SPAG4 | 3.6304299 | 6.2830535 | 2.6526236 | 8.82E-11 | 3.39E-09 |
| ENSG00000182326 | C1S | 5.0553133 | 7.7036115 | 2.6482983 | 1.22E-09 | 3.90E-08 |
| ENSG00000114315 | HES1 | 0 | 2.6374208 | 2.6374208 | 2.04E-05 | 0.0002563 |
| ENSG00000137331 | IER3 | 9.9474562 | 12.581005 | 2.633549 | 1.22E-11 | 5.50E-10 |
| ENSG00000136052 | SLC41A2 | 9.1510494 | 11.773644 | 2.6225948 | 5.91E-28 | 2.12E-25 |
| ENSG00000163568 | AIM2 | 2.743726 | 5.3602988 | 2.6165728 | 3.17E-06 | 4.92E-05 |
| ENSG00000225940 | C5orf67 | 0.2567042 | 2.8665926 | 2.6098884 | 0.00034 | 0.0029598 |
| ENSG00000139053 | PDE6H | 1.0878995 | 3.6882909 | 2.6003914 | 2.13E-05 | 0.0002665 |
| ENSG00000132003 | ZSWIM4 | 6.7084326 | 9.2913812 | 2.5829486 | 2.09E-15 | 1.72E-13 |
| ENSG00000114251 | WNT5A | 6.345764 | 8.9243383 | 2.5785744 | 3.16E-15 | 2.54E-13 |
| ENSG00000117595 | IRF6 | 1.6283045 | 4.1951014 | 2.566797 | 1.21E-05 | 0.0001618 |
| ENSG00000142621 | FHAD1 | 6.8457584 | 9.4037244 | 2.557966 | 1.10E-13 | 6.94E-12 |
| ENSG00000111058 | ACSS3 | 3.8024818 | 6.358738 | 2.5562562 | 2.13E-07 | 4.39E-06 |
| ENSG00000145428 | RNF175 | 5.8976347 | 8.4529623 | 2.5553276 | 1.25E-13 | 7.84E-12 |
| ENSG00000112715 | VEGFA | 8.5009595 | 11.053821 | 2.552862 | 4.38E-21 | 7.32E-19 |
| ENSG00000002587 | HS3ST1 | 7.8666088 | 10.41746 | 2.5508515 | 7.20E-23 | 1.51E-20 |
| ENSG00000127507 | ADGRE2 | 10.713832 | 13.255394 | 2.5415621 | 6.14E-19 | 8.17E-17 |
| ENSG00000150637 | CD226 | 6.5016891 | 9.039971 | 2.5382819 | 2.80E-14 | 1.95E-12 |
| ENSG00000204345 | CD300LD | 0 | 2.5295823 | 2.5295823 | 4.53E-05 | 0.0005188 |
| ENSG00000160963 | COL26A1 | 0.4109753 | 2.9369387 | 2.5259634 | 9.78E-05 | 0.0010126 |
| ENSG00000088826 | SMOX | 7.6561088 | 10.178999 | 2.5228907 | 1.62E-28 | 6.17E-26 |
| ENSG00000111674 | ENO2 | 6.5622852 | 9.0827071 | 2.5204218 | 2.11E-14 | 1.49E-12 |
| ENSG00000101188 | NTSR1 | 0 | 2.5181738 | 2.5181738 | 0.0002343 | 0.0021534 |
| ENSG00000150337 | FCGR1A | 9.4334162 | 11.949713 | 2.5162969 | 1.85E-26 | 5.47E-24 |
| ENSG00000106689 | LHX2 | 0.81652 | 3.3312031 | 2.5146832 | 0.000381 | 0.0032608 |
| ENSG00000183023 | SLC8A1 | 11.418538 | 13.918979 | 2.500441 | 4.32E-26 | 1.21E-23 |
| ENSG00000167244 | IGF2 | 1.653548 | 4.1518862 | 2.4983382 | 7.46E-06 | 0.0001061 |
| ENSG00000026508 | CD44 | 12.687374 | 15.183753 | 2.4963785 | 2.10E-37 | 1.87E-34 |
| ENSG00000120156 | TEK | 1.1854984 | 3.6812127 | 2.4957143 | 0.0001091 | 0.0011137 |
| ENSG00000122870 | BICC1 | 1.6495059 | 4.1444039 | 2.494898 | 0.0015765 | 0.0108725 |
| ENSG00000123405 | NFE2 | 1.2399138 | 3.7344795 | 2.4945658 | 4.63E-05 | 0.000529 |
| ENSG00000029153 | ARNTL2 | 7.4997808 | 9.9942985 | 2.4945176 | 1.64E-20 | 2.62E-18 |
| ENSG00000120708 | TGFBI | 14.722731 | 17.207376 | 2.4846454 | 2.28E-33 | 1.49E-30 |
| ENSG00000102445 | RUBCNL | 8.3795356 | 10.8629 | 2.483364 | 1.81E-15 | 1.51E-13 |
| ENSG00000154380 | ENAH | 4.1054053 | 6.5811105 | 2.4757052 | 3.39E-17 | 3.61E-15 |
| ENSG00000168874 | ATOH8 | 1.156057 | 3.6316019 | 2.4755449 | 1.15E-05 | 0.0001547 |
| ENSG00000198003 | CCDC151 | 0.5127691 | 2.9866015 | 2.4738324 | 0.0002466 | 0.0022511 |
| ENSG00000148926 | ADM | 9.2529253 | 11.713882 | 2.4609567 | 6.24E-28 | 2.22E-25 |
| ENSG00000282988 | HIST1H3D | 6.6278963 | 9.0883321 | 2.4604358 | 0.0005751 | 0.004616 |
| ENSG00000125430 | HS3ST3B1 | 7.0301575 | 9.4864558 | 2.4562983 | 9.90E-16 | 8.63E-14 |
| ENSG00000140450 | ARRDC4 | 8.9480553 | 11.399568 | 2.4515128 | 3.38E-34 | 2.46E-31 |
| ENSG00000170379 | TCAF2 | 5.474488 | 7.9189564 | 2.4444684 | 1.79E-10 | 6.57E-09 |
| ENSG00000206052 | DOK6 | 2.3324167 | 4.7760367 | 2.44362 | 4.36E-09 | 1.26E-07 |
| ENSG00000111012 | CYP27B1 | 6.29228 | 8.7334495 | 2.4411695 | 1.12E-08 | 2.99E-07 |
| ENSG00000133317 | LGALS12 | 0.2109952 | 2.6488872 | 2.437892 | 2.51E-06 | 3.98E-05 |
| ENSG00000166265 | CYYR1 | 0.86999 | 3.3042804 | 2.4342903 | 1.92E-05 | 0.0002438 |
| ENSG00000138378 | STAT4 | 6.2716264 | 8.7030036 | 2.4313772 | 1.23E-09 | 3.93E-08 |
| ENSG00000100678 | SLC8A3 | 0.3231677 | 2.7447147 | 2.421547 | 0.0009242 | 0.0069307 |
| ENSG00000115963 | RND3 | 6.8343065 | 9.2535707 | 2.4192643 | 2.15E-07 | 4.42E-06 |
| ENSG00000072952 | MRVI1 | 3.3565799 | 5.7711771 | 2.4145971 | 1.13E-09 | 3.63E-08 |
| ENSG00000122862 | SRGN | 12.488572 | 14.901836 | 2.4132634 | 6.47E-66 | 4.16E-62 |
| ENSG00000134333 | LDHA | 11.563941 | 13.976929 | 2.4129879 | 1.38E-25 | 3.72E-23 |
| ENSG00000073756 | PTGS2 | 6.1135111 | 8.5256569 | 2.4121457 | 2.40E-13 | 1.43E-11 |
| ENSG00000113070 | HBEGF | 7.8925916 | 10.297149 | 2.4045569 | 3.88E-18 | 4.68E-16 |
| ENSG00000221937 | TAS2R40 | 0.4132075 | 2.8168868 | 2.4036793 | 4.68E-06 | 6.96E-05 |
| ENSG00000166523 | CLEC4E | 8.8885795 | 11.291773 | 2.4031936 | 1.37E-11 | 6.12E-10 |
| ENSG00000164741 | DLC1 | 5.9473644 | 8.3467878 | 2.3994233 | 1.78E-15 | 1.48E-13 |
| ENSG00000231256 | C17orf105 | 0.4198176 | 2.8192226 | 2.399405 | 2.24E-05 | 0.0002788 |
| ENSG00000006468 | ETV1 | 0.1439024 | 2.5376545 | 2.393752 | 6.08E-07 | 1.13E-05 |
| ENSG00000173281 | PPP1R3B | 7.8308539 | 10.220615 | 2.389761 | 1.86E-23 | 4.08E-21 |
| ENSG00000136286 | MYO1G | 10.447645 | 12.833979 | 2.3863343 | 9.24E-28 | 3.18E-25 |
| ENSG00000186648 | CARMIL3 | 6.254757 | 8.6385644 | 2.3838074 | 5.73E-11 | 2.29E-09 |
| ENSG00000080031 | PTPRH | 0.7705992 | 3.1513944 | 2.3807952 | 3.15E-06 | 4.90E-05 |
| ENSG00000197646 | PDCD1LG2 | 9.0457891 | 11.415424 | 2.369635 | 2.49E-42 | 3.11E-39 |
| ENSG00000143382 | ADAMTSL4 | 10.531374 | 12.898205 | 2.3668308 | 3.83E-30 | 1.78E-27 |
| ENSG00000117318 | ID3 | 7.7703038 | 10.133425 | 2.3631208 | 6.55E-11 | 2.60E-09 |
| ENSG00000075461 | CACNG4 | 1.6810414 | 4.0440734 | 2.363032 | 0.0055831 | 0.0311046 |
| ENSG00000073150 | PANX2 | 4.1944023 | 6.551693 | 2.3572907 | 2.47E-10 | 8.92E-09 |
| ENSG00000167077 | MEI1 | 5.991049 | 8.3431216 | 2.3520725 | 5.24E-14 | 3.46E-12 |
| ENSG00000170542 | SERPINB9 | 9.1550923 | 11.503425 | 2.3483326 | 1.59E-30 | 7.74E-28 |
| ENSG00000175130 | MARCKSL1 | 4.8932718 | 7.2405547 | 2.3472829 | 1.67E-11 | 7.31E-10 |
| ENSG00000141068 | KSR1 | 8.3605264 | 10.702593 | 2.342067 | 4.81E-27 | 1.49E-24 |
| ENSG00000135919 | SERPINE2 | 2.9734968 | 5.3056839 | 2.3321871 | 0.0020044 | 0.0132535 |
| ENSG00000148143 | ZNF462 | 6.4549099 | 8.7860173 | 2.3311074 | 9.09E-30 | 3.98E-27 |
| ENSG00000162494 | LRRC38 | 0 | 2.3258938 | 2.3258938 | 0.0005828 | 0.0046733 |
| ENSG00000152784 | PRDM8 | 3.1705051 | 5.483834 | 2.3133289 | 2.50E-11 | 1.07E-09 |
| ENSG00000184588 | PDE4B | 9.1365431 | 11.448201 | 2.3116584 | 1.21E-19 | 1.77E-17 |
| ENSG00000182853 | VMO1 | 4.8099768 | 7.117405 | 2.3074282 | 4.78E-17 | 5.03E-15 |
| ENSG00000119411 | BSPRY | 1.9626083 | 4.2682825 | 2.3056742 | 1.72E-05 | 0.00022 |
| ENSG00000149798 | CDC42EP2 | 3.2516228 | 5.5510112 | 2.2993884 | 0.0014015 | 0.0098412 |
| ENSG00000145832 | SLC25A48 | 3.5705441 | 5.8596919 | 2.2891479 | 1.89E-06 | 3.12E-05 |
| ENSG00000105825 | TFPI2 | 0.2123925 | 2.4949282 | 2.2825356 | 1.86E-05 | 0.0002361 |
| ENSG00000271321 | CTAGE6 | 1.9619204 | 4.2301043 | 2.2681839 | 4.94E-08 | 1.17E-06 |
| ENSG00000130396 | AFDN | 9.2294939 | 11.493559 | 2.2640656 | 3.27E-34 | 2.42E-31 |
| ENSG00000189430 | NCR1 | 0.6011404 | 2.8649221 | 2.2637817 | 7.25E-05 | 0.0007814 |
| ENSG00000067445 | TRO | 1.0820214 | 3.3440051 | 2.2619838 | 0.0075771 | 0.0399878 |
| ENSG00000072682 | P4HA2 | 6.9023305 | 9.1615946 | 2.2592641 | 1.18E-16 | 1.17E-14 |
| ENSG00000120949 | TNFRSF8 | 6.718775 | 8.9720943 | 2.2533192 | 9.03E-17 | 9.17E-15 |
| ENSG00000164023 | SGMS2 | 7.8628779 | 10.11616 | 2.2532819 | 7.31E-32 | 3.95E-29 |
| ENSG00000165621 | OXGR1 | 0 | 2.2492909 | 2.2492909 | 0.0007537 | 0.0058218 |
| ENSG00000050030 | NEXMIF | 1.038481 | 3.2875674 | 2.2490865 | 0.0012237 | 0.0087839 |
| ENSG00000135048 | TMEM2 | 11.60268 | 13.851737 | 2.2490565 | 6.49E-16 | 5.80E-14 |
| ENSG00000082397 | EPB41L3 | 12.412522 | 14.661044 | 2.2485223 | 2.12E-48 | 4.03E-45 |
| ENSG00000134954 | ETS1 | 4.9572589 | 7.2026751 | 2.2454161 | 6.92E-10 | 2.31E-08 |
| ENSG00000189367 | KIAA0408 | 0.4753194 | 2.7094447 | 2.2341252 | 0.0011372 | 0.0082467 |
| ENSG00000188313 | PLSCR1 | 9.8013147 | 12.035199 | 2.2338847 | 1.04E-45 | 1.69E-42 |
| ENSG00000069667 | RORA | 8.1303415 | 10.340149 | 2.2098074 | 2.78E-17 | 2.98E-15 |
| ENSG00000132185 | FCRLA | 3.741158 | 5.9509374 | 2.2097794 | 1.20E-05 | 0.000161 |
| ENSG00000164050 | PLXNB1 | 2.1035607 | 4.3108048 | 2.2072441 | 2.49E-05 | 0.0003058 |
| ENSG00000166068 | SPRED1 | 11.293998 | 13.498477 | 2.2044793 | 5.63E-24 | 1.31E-21 |
| ENSG00000105991 | HOXA1 | 2.2436608 | 4.4377158 | 2.194055 | 6.19E-05 | 0.0006803 |
| ENSG00000133800 | LYVE1 | 1.1865519 | 3.3764537 | 2.1899018 | 0.0028914 | 0.0180009 |
| ENSG00000158955 | WNT9B | 0.1802225 | 2.3632358 | 2.1830133 | 5.97E-05 | 0.0006596 |
| ENSG00000198483 | ANKRD35 | 1.1330303 | 3.3136434 | 2.1806131 | 0.0070435 | 0.0377129 |
| ENSG00000137101 | CD72 | 6.4086392 | 8.5891966 | 2.1805574 | 2.29E-11 | 9.84E-10 |
| ENSG00000134569 | LRP4 | 2.7838853 | 4.9614384 | 2.1775531 | 1.07E-08 | 2.86E-07 |
| ENSG00000265972 | TXNIP | 11.565973 | 13.743472 | 2.1774994 | 7.80E-14 | 5.00E-12 |
| ENSG00000165757 | JCAD | 0.3796922 | 2.5543786 | 2.1746864 | 0.0001207 | 0.0012156 |
| ENSG00000276409 | CCL14 | 0.4303819 | 2.6030015 | 2.1726197 | 0.000352 | 0.0030504 |
| ENSG00000109107 | ALDOC | 6.5672196 | 8.7379865 | 2.170767 | 1.43E-13 | 8.84E-12 |
| ENSG00000163217 | BMP10 | 0.8851476 | 3.0524757 | 2.1673281 | 0.0005287 | 0.004297 |
| ENSG00000117154 | IGSF21 | 0 | 2.1651865 | 2.1651865 | 3.92E-06 | 5.93E-05 |
| ENSG00000148482 | SLC39A12 | 3.1933925 | 5.3473384 | 2.1539459 | 1.30E-05 | 0.0001724 |
| ENSG00000175874 | CREG2 | 1.9625549 | 4.1162557 | 2.1537007 | 0.0002539 | 0.0023071 |
| ENSG00000162878 | PKDCC | 1.3075372 | 3.4605912 | 2.153054 | 0.000414 | 0.0034957 |
| ENSG00000140519 | RHCG | 2.1241004 | 4.2761206 | 2.1520202 | 0.0005555 | 0.0044817 |
| ENSG00000158428 | CATIP | 4.0215325 | 6.171587 | 2.1500546 | 3.57E-15 | 2.83E-13 |
| ENSG00000134827 | TCN1 | 2.1809102 | 4.3275241 | 2.1466139 | 2.41E-05 | 0.0002977 |
| ENSG00000188215 | DCUN1D3 | 8.8034333 | 10.946426 | 2.1429923 | 1.03E-18 | 1.32E-16 |
| ENSG00000197555 | SIPA1L1 | 10.333151 | 12.473007 | 2.1398562 | 7.46E-22 | 1.35E-19 |
| ENSG00000142627 | EPHA2 | 0 | 2.1360842 | 2.1360842 | 1.04E-05 | 0.0001412 |
| ENSG00000013619 | MAMLD1 | 5.0811287 | 7.2151096 | 2.1339809 | 9.59E-12 | 4.42E-10 |
| ENSG00000115828 | QPCT | 8.4533944 | 10.58164 | 2.1282455 | 7.48E-23 | 1.56E-20 |
| ENSG00000180210 | F2 | 1.2469358 | 3.3744265 | 2.1274907 | 0.0001926 | 0.0018225 |
| ENSG00000114737 | CISH | 7.847023 | 9.9732726 | 2.1262496 | 7.27E-28 | 2.54E-25 |
| ENSG00000100558 | PLEK2 | 4.0186947 | 6.1447929 | 2.1260982 | 3.49E-08 | 8.50E-07 |
| ENSG00000111817 | DSE | 10.854113 | 12.977656 | 2.1235434 | 3.02E-18 | 3.70E-16 |
| ENSG00000181374 | CCL13 | 6.3448682 | 8.466412 | 2.1215437 | 1.31E-08 | 3.44E-07 |
| ENSG00000128872 | TMOD2 | 8.7055173 | 10.826366 | 2.120849 | 3.47E-08 | 8.48E-07 |
| ENSG00000128918 | ALDH1A2 | 8.6246429 | 10.74415 | 2.1195072 | 9.21E-05 | 0.0009609 |
| ENSG00000136160 | EDNRB | 6.5859188 | 8.7046853 | 2.1187666 | 2.21E-06 | 3.57E-05 |
| ENSG00000152256 | PDK1 | 9.0327565 | 11.150303 | 2.1175469 | 3.95E-12 | 1.92E-10 |
| ENSG00000131355 | ADGRE3 | 4.6564838 | 6.7723715 | 2.1158876 | 2.33E-05 | 0.0002895 |
| ENSG00000101460 | MAP1LC3A | 5.3557135 | 7.4715826 | 2.1158691 | 3.16E-11 | 1.33E-09 |
| ENSG00000152229 | PSTPIP2 | 9.9776347 | 12.090536 | 2.1129015 | 2.13E-22 | 4.17E-20 |
| ENSG00000158352 | SHROOM4 | 3.054787 | 5.1656659 | 2.1108789 | 1.31E-07 | 2.84E-06 |
| ENSG00000130202 | NECTIN2 | 9.7036978 | 11.811959 | 2.1082614 | 1.89E-29 | 7.81E-27 |
| ENSG00000057704 | TMCC3 | 7.7061522 | 9.8107282 | 2.1045759 | 5.95E-06 | 8.62E-05 |
| ENSG00000111052 | LIN7A | 6.7149906 | 8.8146395 | 2.0996489 | 1.54E-09 | 4.82E-08 |
| ENSG00000181458 | TMEM45A | 5.5432525 | 7.6391724 | 2.0959199 | 2.63E-09 | 7.89E-08 |
| ENSG00000153208 | MERTK | 11.539112 | 13.631419 | 2.092307 | 2.25E-30 | 1.07E-27 |
| ENSG00000104341 | LAPTM4B | 4.6545186 | 6.7468007 | 2.0922821 | 8.56E-10 | 2.82E-08 |
| ENSG00000056736 | IL17RB | 6.9787133 | 9.0591144 | 2.0804012 | 4.45E-16 | 4.07E-14 |
| ENSG00000103647 | CORO2B | 1.0737347 | 3.1525476 | 2.0788129 | 0.0066254 | 0.0358292 |
| ENSG00000158258 | CLSTN2 | 1.6484743 | 3.7271571 | 2.0786828 | 0.0026278 | 0.0166295 |
| ENSG00000157168 | NRG1 | 0.623506 | 2.6995058 | 2.0759997 | 0.0011257 | 0.0081811 |
| ENSG00000132872 | SYT4 | 0 | 2.0687445 | 2.0687445 | 9.72E-06 | 0.0001335 |
| ENSG00000136830 | FAM129B | 10.949834 | 13.016079 | 2.066245 | 8.75E-15 | 6.56E-13 |
| ENSG00000169116 | PARM1 | 0.1802225 | 2.2460804 | 2.0658579 | 8.68E-05 | 0.0009135 |
| ENSG00000093134 | VNN3 | 5.031564 | 7.0972431 | 2.0656791 | 1.04E-11 | 4.80E-10 |
| ENSG00000185897 | FFAR3 | 1.2564923 | 3.3202849 | 2.0637927 | 0.0046677 | 0.0269186 |
| ENSG00000116741 | RGS2 | 8.131049 | 10.194788 | 2.0637389 | 3.56E-08 | 8.66E-07 |
| ENSG00000047597 | XK | 2.1773846 | 4.2393064 | 2.0619219 | 4.79E-05 | 0.0005459 |
| ENSG00000152953 | STK32B | 5.4636543 | 7.5178379 | 2.0541836 | 5.00E-12 | 2.41E-10 |
| ENSG00000108691 | CCL2 | 12.703602 | 14.754512 | 2.0509104 | 5.94E-07 | 1.10E-05 |
| ENSG00000165325 | DEUP1 | 1.3999172 | 3.4499079 | 2.0499907 | 0.00063 | 0.0049915 |
| ENSG00000143924 | EML4 | 11.451703 | 13.501423 | 2.0497199 | 4.32E-31 | 2.15E-28 |
| ENSG00000105376 | ICAM5 | 5.0561982 | 7.1052112 | 2.049013 | 8.66E-07 | 1.54E-05 |
| ENSG00000144285 | SCN1A | 0.7127518 | 2.7577295 | 2.0449776 | 0.005698 | 0.031619 |
| ENSG00000171873 | ADRA1D | 1.5799736 | 3.6204992 | 2.0405257 | 0.0025557 | 0.0162484 |
| ENSG00000184500 | PROS1 | 7.2493795 | 9.2811078 | 2.0317283 | 9.16E-14 | 5.83E-12 |
| ENSG00000175294 | CATSPER1 | 2.9389194 | 4.9678247 | 2.0289053 | 0.0013914 | 0.0097858 |
| ENSG00000174705 | SH3PXD2B | 11.179508 | 13.207333 | 2.0278259 | 2.31E-16 | 2.19E-14 |
| ENSG00000157557 | ETS2 | 11.103798 | 13.12505 | 2.0212513 | 2.95E-12 | 1.45E-10 |
| ENSG00000076554 | TPD52 | 4.7612546 | 6.7717647 | 2.0105101 | 6.61E-06 | 9.48E-05 |
| ENSG00000260314 | MRC1 | 14.593819 | 16.604075 | 2.0102565 | 1.25E-18 | 1.59E-16 |
| ENSG00000147896 | IFNK | 6.0894102 | 8.0862225 | 1.9968123 | 3.48E-21 | 5.88E-19 |
| ENSG00000189221 | MAOA | 7.8571218 | 9.8510037 | 1.9938819 | 2.76E-21 | 4.71E-19 |
| ENSG00000079101 | CLUL1 | 0.1672071 | 2.1597547 | 1.9925476 | 0.0010932 | 0.0079846 |
| ENSG00000139182 | CLSTN3 | 4.8741157 | 6.8663444 | 1.9922288 | 4.95E-07 | 9.35E-06 |
| ENSG00000229859 | PGA3 | 1.4932795 | 3.4854951 | 1.9922157 | 0.0002875 | 0.0025685 |
| ENSG00000156413 | FUT6 | 0.1439024 | 2.130038 | 1.9861356 | 4.91E-05 | 0.0005577 |
| ENSG00000087903 | RFX2 | 7.1147224 | 9.098304 | 1.9835816 | 5.96E-18 | 6.98E-16 |
| ENSG00000174125 | TLR1 | 10.852178 | 12.835526 | 1.9833476 | 1.21E-15 | 1.04E-13 |
| ENSG00000258083 | OR9A4 | 0.2968101 | 2.2798227 | 1.9830126 | 4.61E-05 | 0.0005271 |
| ENSG00000132164 | SLC6A11 | 1.5616282 | 3.544533 | 1.9829048 | 0.000212 | 0.0019746 |
| ENSG00000112096 | SOD2 | 13.291246 | 15.267958 | 1.9767125 | 7.24E-26 | 1.98E-23 |
| ENSG00000173597 | SULT1B1 | 3.6539662 | 5.6244228 | 1.9704566 | 2.82E-06 | 4.43E-05 |
| ENSG00000142549 | IGLON5 | 3.4164606 | 5.3865656 | 1.970105 | 5.27E-07 | 9.89E-06 |
| ENSG00000119686 | FLVCR2 | 9.0773742 | 11.046964 | 1.9695896 | 2.79E-22 | 5.32E-20 |
| ENSG00000113721 | PDGFRB | 2.4807728 | 4.442669 | 1.9618962 | 0.0016783 | 0.0114627 |
| ENSG00000197272 | IL27 | 2.7253974 | 4.6807821 | 1.9553846 | 6.41E-06 | 9.21E-05 |
| ENSG00000100731 | PCNX1 | 11.964902 | 13.919264 | 1.9543621 | 2.03E-32 | 1.19E-29 |
| ENSG00000166825 | ANPEP | 13.270674 | 15.223947 | 1.9532729 | 9.41E-19 | 1.22E-16 |
| ENSG00000173110 | HSPA6 | 8.3352424 | 10.287384 | 1.9521419 | 1.45E-09 | 4.56E-08 |
| ENSG00000182957 | SPATA13 | 9.1197433 | 11.068573 | 1.9488298 | 3.37E-10 | 1.19E-08 |
| ENSG00000196460 | RFX8 | 0.4018094 | 2.3472712 | 1.9454618 | 0.0010659 | 0.0078221 |
| ENSG00000064787 | BCAS1 | 2.1484097 | 4.0931412 | 1.9447315 | 1.40E-05 | 0.0001838 |
| ENSG00000138166 | DUSP5 | 6.3997968 | 8.3414475 | 1.9416507 | 1.20E-05 | 0.000161 |
| ENSG00000136379 | ABHD17C | 4.3732379 | 6.3139429 | 1.940705 | 2.08E-06 | 3.38E-05 |
| ENSG00000205502 | C2CD4B | 0 | 1.9380454 | 1.9380454 | 0.0001165 | 0.0011784 |
| ENSG00000165091 | TMC1 | 0.7855858 | 2.7227584 | 1.9371726 | 0.009231 | 0.0469174 |
| ENSG00000133816 | MICAL2 | 9.6024676 | 11.53851 | 1.9360425 | 1.14E-09 | 3.66E-08 |
| ENSG00000197702 | PARVA | 2.0612035 | 3.9971936 | 1.9359901 | 0.0016579 | 0.0113495 |
| ENSG00000243649 | CFB | 8.1207007 | 10.056591 | 1.93589 | 1.89E-11 | 8.21E-10 |
| ENSG00000196209 | SIRPB2 | 9.502839 | 11.432474 | 1.9296351 | 1.95E-10 | 7.13E-09 |
| ENSG00000261594 | TPBGL | 4.1598838 | 6.0893962 | 1.9295124 | 1.23E-06 | 2.12E-05 |
| ENSG00000102359 | SRPX2 | 3.8715053 | 5.7979303 | 1.9264249 | 1.06E-05 | 0.0001442 |
| ENSG00000103888 | CEMIP | 7.4247484 | 9.3490146 | 1.9242662 | 1.20E-07 | 2.61E-06 |
| ENSG00000172594 | SMPDL3A | 8.6450027 | 10.568062 | 1.9230593 | 2.26E-16 | 2.15E-14 |
| ENSG00000196358 | NTNG2 | 3.0360006 | 4.9571573 | 1.9211568 | 0.0001591 | 0.0015457 |
| ENSG00000120262 | CCDC170 | 8.413299 | 10.333763 | 1.9204643 | 4.28E-15 | 3.36E-13 |
| ENSG00000116299 | KIAA1324 | 2.5533663 | 4.4724161 | 1.9190498 | 2.31E-05 | 0.0002866 |
| ENSG00000090924 | PLEKHG2 | 9.2279108 | 11.143747 | 1.9158359 | 7.42E-18 | 8.54E-16 |
| ENSG00000024422 | EHD2 | 1.3059 | 3.2207584 | 1.9148584 | 0.0016623 | 0.0113731 |
| ENSG00000171476 | HOPX | 2.4235924 | 4.3360573 | 1.912465 | 0.0016472 | 0.011282 |
| ENSG00000155659 | VSIG4 | 11.432349 | 13.344134 | 1.9117847 | 2.35E-21 | 4.04E-19 |
| ENSG00000090659 | CD209 | 10.312355 | 12.223526 | 1.9111709 | 1.77E-16 | 1.72E-14 |
| ENSG00000256120 | SOX5-AS1 | 0.8655657 | 2.7760458 | 1.9104801 | 0.0001486 | 0.0014557 |
| ENSG00000172322 | CLEC12A | 7.8740386 | 9.7837991 | 1.9097605 | 2.77E-10 | 9.94E-09 |
| ENSG00000137462 | TLR2 | 9.8591335 | 11.768003 | 1.9088693 | 2.66E-11 | 1.13E-09 |
| ENSG00000187912 | CLEC17A | 1.8047382 | 3.7115749 | 1.9068366 | 7.09E-05 | 0.0007673 |
| ENSG00000157613 | CREB3L1 | 0 | 1.8986287 | 1.8986287 | 0.0001319 | 0.0013136 |
| ENSG00000179044 | EXOC3L1 | 0.4994004 | 2.3954065 | 1.8960061 | 0.0039041 | 0.0232284 |
| ENSG00000107249 | GLIS3 | 6.5067445 | 8.4007725 | 1.894028 | 9.58E-12 | 4.42E-10 |
| ENSG00000141497 | ZMYND15 | 7.7002917 | 9.5921715 | 1.8918798 | 5.18E-14 | 3.43E-12 |
| ENSG00000113369 | ARRDC3 | 10.411314 | 12.301214 | 1.8898996 | 9.33E-14 | 5.93E-12 |
| ENSG00000203499 | IQANK1 | 1.3304915 | 3.2193303 | 1.8888388 | 0.0007235 | 0.0056245 |
| ENSG00000119326 | CTNNAL1 | 5.4902996 | 7.3779774 | 1.8876777 | 3.22E-11 | 1.35E-09 |
| ENSG00000196352 | CD55 | 9.2124027 | 11.098992 | 1.8865889 | 1.05E-12 | 5.60E-11 |
| ENSG00000104951 | IL4I1 | 10.518562 | 12.404031 | 1.8854683 | 1.31E-13 | 8.12E-12 |
| ENSG00000137193 | PIM1 | 10.483692 | 12.360133 | 1.8764411 | 7.54E-19 | 9.94E-17 |
| ENSG00000163737 | PF4 | 0 | 1.8735631 | 1.8735631 | 5.64E-05 | 0.0006288 |
| ENSG00000108813 | DLX4 | 1.0431344 | 2.9160204 | 1.8728861 | 0.0003889 | 0.0033172 |
| ENSG00000134363 | FST | 0 | 1.8710967 | 1.8710967 | 4.78E-05 | 0.0005452 |
| ENSG00000108700 | CCL8 | 7.0303247 | 8.8997978 | 1.8694732 | 3.40E-05 | 0.0004022 |
| ENSG00000139610 | CELA1 | 1.0094253 | 2.8781742 | 1.8687489 | 0.0067844 | 0.0365269 |
| ENSG00000154229 | PRKCA | 9.9924419 | 11.859813 | 1.867371 | 3.43E-14 | 2.36E-12 |
| ENSG00000172403 | SYNPO2 | 1.8514732 | 3.7161813 | 1.8647081 | 0.0010847 | 0.0079323 |
| ENSG00000177453 | NIM1K | 2.532146 | 4.3936141 | 1.8614681 | 1.65E-06 | 2.77E-05 |
| ENSG00000067057 | PFKP | 9.0121995 | 10.873374 | 1.8611745 | 2.71E-12 | 1.35E-10 |
| ENSG00000127954 | STEAP4 | 3.5528516 | 5.4139516 | 1.8611001 | 0.000633 | 0.0050133 |
| ENSG00000100116 | GCAT | 2.8440561 | 4.7046176 | 1.8605615 | 0.0026292 | 0.0166357 |
| ENSG00000060558 | GNA15 | 10.224242 | 12.073176 | 1.8489342 | 3.04E-24 | 7.36E-22 |
| ENSG00000135821 | GLUL | 15.266965 | 17.111236 | 1.8442709 | 1.73E-26 | 5.15E-24 |
| ENSG00000110203 | FOLR3 | 2.981196 | 4.8252905 | 1.8440945 | 5.72E-05 | 0.0006363 |
| ENSG00000164038 | SLC9B2 | 2.8840859 | 4.7255176 | 1.8414318 | 2.18E-05 | 0.0002727 |
| ENSG00000153094 | BCL2L11 | 8.7229248 | 10.559849 | 1.8369238 | 2.20E-08 | 5.56E-07 |
| ENSG00000184005 | ST6GALNAC3 | 3.0376678 | 4.8736228 | 1.835955 | 0.0006101 | 0.0048545 |
| ENSG00000005486 | RHBDD2 | 8.2762406 | 10.109266 | 1.8330252 | 9.70E-29 | 3.79E-26 |
| ENSG00000130487 | KLHDC7B | 2.221796 | 4.0503446 | 1.8285486 | 1.28E-05 | 0.0001703 |
| ENSG00000102683 | SGCG | 4.0324454 | 5.858476 | 1.8260306 | 1.13E-05 | 0.0001523 |
| ENSG00000124491 | F13A1 | 11.183624 | 12.998818 | 1.8151931 | 0.0002094 | 0.0019594 |
| ENSG00000160179 | ABCG1 | 8.7857318 | 10.600411 | 1.8146793 | 5.21E-10 | 1.78E-08 |
| ENSG00000004468 | CD38 | 10.34417 | 12.156455 | 1.8122851 | 5.08E-18 | 6.01E-16 |
| ENSG00000167549 | CORO6 | 3.106635 | 4.9173324 | 1.8106974 | 1.61E-07 | 3.40E-06 |
| ENSG00000164181 | ELOVL7 | 3.9743266 | 5.7836146 | 1.809288 | 1.71E-05 | 0.0002198 |
| ENSG00000166341 | DCHS1 | 5.3514246 | 7.1602087 | 1.8087842 | 1.03E-05 | 0.0001406 |
| ENSG00000167034 | NKX3-1 | 0.3759924 | 2.184371 | 1.8083785 | 0.0008374 | 0.0063625 |
| ENSG00000166926 | MS4A6E | 2.1402691 | 3.9485064 | 1.8082373 | 0.0019024 | 0.0127035 |
| ENSG00000135047 | CTSL | 14.590487 | 16.398188 | 1.8077004 | 3.19E-16 | 2.97E-14 |
| ENSG00000106333 | PCOLCE | 3.4159127 | 5.2174858 | 1.8015731 | 7.04E-05 | 0.0007627 |
| ENSG00000159640 | ACE | 10.979207 | 12.779156 | 1.7999487 | 0.0050931 | 0.028873 |
| ENSG00000159450 | TCHH | 4.5884081 | 6.38339 | 1.7949819 | 6.19E-07 | 1.15E-05 |
| ENSG00000262874 | C19orf84 | 0.3778489 | 2.170221 | 1.7923721 | 0.0015027 | 0.0104473 |
| ENSG00000169136 | ATF5 | 10.283952 | 12.076081 | 1.7921288 | 1.25E-14 | 9.16E-13 |
| ENSG00000135318 | NT5E | 5.8113354 | 7.603304 | 1.7919686 | 2.85E-07 | 5.74E-06 |
| ENSG00000085117 | CD82 | 10.193989 | 11.98165 | 1.7876604 | 3.87E-19 | 5.36E-17 |
| ENSG00000131386 | GALNT15 | 0 | 1.7875337 | 1.7875337 | 0.000116 | 0.0011743 |
| ENSG00000197249 | SERPINA1 | 12.134314 | 13.920228 | 1.7859142 | 5.83E-09 | 1.64E-07 |
| ENSG00000175040 | CHST2 | 7.7645177 | 9.5437332 | 1.7792155 | 1.88E-06 | 3.10E-05 |
| ENSG00000167608 | TMC4 | 5.9533768 | 7.7325086 | 1.7791318 | 7.70E-09 | 2.12E-07 |
| ENSG00000127399 | LRRC61 | 3.6950929 | 5.4726467 | 1.7775538 | 0.0001789 | 0.0017084 |
| ENSG00000136449 | MYCBPAP | 1.5495804 | 3.3195701 | 1.7699898 | 0.0033173 | 0.0202322 |
| ENSG00000143545 | RAB13 | 10.321528 | 12.090687 | 1.7691588 | 5.12E-38 | 4.77E-35 |
| ENSG00000185070 | FLRT2 | 8.224905 | 9.9917447 | 1.7668397 | 7.68E-05 | 0.0008213 |
| ENSG00000116285 | ERRFI1 | 5.842794 | 7.6079194 | 1.7651253 | 2.21E-11 | 9.56E-10 |
| ENSG00000101977 | MCF2 | 3.0009206 | 4.7660004 | 1.7650798 | 1.20E-06 | 2.07E-05 |
| ENSG00000154310 | TNIK | 11.179388 | 12.943865 | 1.7644769 | 5.80E-47 | 1.06E-43 |
| ENSG00000171236 | LRG1 | 4.4372003 | 6.2004112 | 1.7632109 | 7.71E-07 | 1.40E-05 |
| ENSG00000184611 | KCNH7 | 3.9366595 | 5.6977016 | 1.7610421 | 6.36E-10 | 2.13E-08 |
| ENSG00000167703 | SLC43A2 | 12.022946 | 13.782164 | 1.7592184 | 7.18E-27 | 2.21E-24 |
| ENSG00000172817 | CYP7B1 | 4.4492381 | 6.2049442 | 1.7557061 | 5.47E-07 | 1.02E-05 |
| ENSG00000104154 | SLC30A4 | 8.3295814 | 10.083452 | 1.7538707 | 6.29E-11 | 2.50E-09 |
| ENSG00000154240 | CEP112 | 5.9711157 | 7.722632 | 1.7515162 | 1.07E-12 | 5.71E-11 |
| ENSG00000260903 | XKR7 | 2.6741609 | 4.420121 | 1.7459602 | 0.0003952 | 0.0033633 |
| ENSG00000149050 | ZNF214 | 4.1883643 | 5.9329527 | 1.7445884 | 0.0042763 | 0.0250815 |
| ENSG00000104112 | SCG3 | 4.0756677 | 5.8198345 | 1.7441668 | 2.86E-08 | 7.08E-07 |
| ENSG00000165685 | TMEM52B | 3.949393 | 5.6929976 | 1.7436046 | 0.0008301 | 0.0063163 |
| ENSG00000169992 | NLGN2 | 4.8047529 | 6.5462382 | 1.7414853 | 1.52E-06 | 2.57E-05 |
| ENSG00000022556 | NLRP2 | 7.0830231 | 8.8236237 | 1.7406005 | 3.22E-06 | 4.99E-05 |
| ENSG00000262664 | OVCA2 | 5.1360505 | 6.8732545 | 1.737204 | 6.67E-05 | 0.0007253 |
| ENSG00000138061 | CYP1B1 | 13.251319 | 14.988007 | 1.7366881 | 6.13E-17 | 6.35E-15 |
| ENSG00000115009 | CCL20 | 3.4946824 | 5.2220346 | 1.7273523 | 0.0001269 | 0.0012717 |
| ENSG00000166922 | SCG5 | 4.2448005 | 5.9715042 | 1.7267037 | 0.0002546 | 0.0023111 |
| ENSG00000160999 | SH2B2 | 6.6510004 | 8.3756259 | 1.7246255 | 1.67E-11 | 7.31E-10 |
| ENSG00000112038 | OPRM1 | 0 | 1.7237175 | 1.7237175 | 0.0003896 | 0.0033208 |
| ENSG00000111640 | GAPDH | 14.269378 | 15.98462 | 1.715242 | 3.36E-28 | 1.24E-25 |
| ENSG00000276410 | HIST1H2BB | 2.9641288 | 4.6784945 | 1.7143657 | 0.000253 | 0.0023002 |
| ENSG00000188786 | MTF1 | 10.718542 | 12.432612 | 1.7140702 | 1.86E-22 | 3.68E-20 |
| ENSG00000167850 | CD300C | 7.8141845 | 9.5280422 | 1.7138577 | 1.63E-08 | 4.23E-07 |
| ENSG00000139318 | DUSP6 | 9.7593166 | 11.472183 | 1.7128665 | 3.57E-05 | 0.0004196 |
| ENSG00000169397 | RNASE3 | 0.2109952 | 1.918902 | 1.7079069 | 0.0027766 | 0.0173999 |
| ENSG00000140859 | KIFC3 | 10.410775 | 12.115357 | 1.7045827 | 8.62E-22 | 1.55E-19 |
| ENSG00000170791 | CHCHD7 | 8.6264788 | 10.330658 | 1.704179 | 3.39E-19 | 4.73E-17 |
| ENSG00000169432 | SCN9A | 6.2515257 | 7.9536687 | 1.702143 | 1.71E-05 | 0.0002193 |
| ENSG00000168060 | NAALADL1 | 1.6850011 | 3.3844802 | 1.6994791 | 0.0085333 | 0.0440413 |
| ENSG00000170458 | CD14 | 13.858582 | 15.549565 | 1.6909828 | 3.80E-19 | 5.30E-17 |
| ENSG00000108511 | HOXB6 | 0.6602175 | 2.3493041 | 1.6890865 | 0.0080515 | 0.0419753 |
| ENSG00000148671 | ADIRF | 2.3156877 | 4.0045699 | 1.6888822 | 0.0020249 | 0.0133725 |
| ENSG00000204577 | LILRB3 | 9.8826357 | 11.569156 | 1.6865199 | 3.96E-17 | 4.21E-15 |
| ENSG00000169764 | UGP2 | 11.27328 | 12.956745 | 1.6834655 | 2.66E-31 | 1.39E-28 |
| ENSG00000275126 | HIST1H4L | 5.5838717 | 7.2666874 | 1.6828157 | 0.0001469 | 0.0014407 |
| ENSG00000163162 | RNF149 | 10.776133 | 12.457 | 1.6808665 | 3.01E-18 | 3.69E-16 |
| ENSG00000157782 | CABP1 | 0 | 1.6802165 | 1.6802165 | 0.0011504 | 0.0083375 |
| ENSG00000091428 | RAPGEF4 | 1.306063 | 2.9799818 | 1.6739188 | 0.0006221 | 0.0049383 |
| ENSG00000151322 | NPAS3 | 0.1570644 | 1.8304038 | 1.6733394 | 0.0004773 | 0.0039465 |
| ENSG00000136696 | IL36B | 0.9391204 | 2.6110442 | 1.6719238 | 0.0080294 | 0.0418848 |
| ENSG00000185100 | ADSSL1 | 4.7871796 | 6.4544169 | 1.6672373 | 5.16E-07 | 9.70E-06 |
| ENSG00000014914 | MTMR11 | 2.6575594 | 4.3247857 | 1.6672264 | 0.0001167 | 0.0011805 |
| ENSG00000125384 | PTGER2 | 8.2353916 | 9.8989262 | 1.6635346 | 7.55E-10 | 2.50E-08 |
| ENSG00000069535 | MAOB | 0 | 1.6604144 | 1.6604144 | 0.0005893 | 0.0047187 |
| ENSG00000219607 | PPP1R3G | 4.6463827 | 6.3027622 | 1.6563795 | 2.81E-06 | 4.41E-05 |
| ENSG00000108825 | PTGES3L-AARSD1 | 1.322401 | 2.9760774 | 1.6536765 | 3.12E-05 | 0.000373 |
| ENSG00000058866 | DGKG | 4.9152172 | 6.5642732 | 1.6490559 | 0.0002528 | 0.0022992 |
| ENSG00000165905 | LARGE2 | 1.9942203 | 3.6414616 | 1.6472413 | 0.0084792 | 0.0438031 |
| ENSG00000115919 | KYNU | 12.146384 | 13.793168 | 1.6467841 | 8.25E-21 | 1.36E-18 |
| ENSG00000264522 | OTUD7B | 6.7529998 | 8.3995654 | 1.6465656 | 6.62E-08 | 1.53E-06 |
| ENSG00000157766 | ACAN | 0 | 1.6462619 | 1.6462619 | 0.0007485 | 0.0057929 |
| ENSG00000150347 | ARID5B | 11.083131 | 12.728127 | 1.6449961 | 3.31E-19 | 4.65E-17 |
| ENSG00000101916 | TLR8 | 11.688422 | 13.331771 | 1.6433487 | 1.36E-18 | 1.73E-16 |
| ENSG00000108556 | CHRNE | 3.9495277 | 5.59145 | 1.6419224 | 2.43E-05 | 0.0002992 |
| ENSG00000133392 | MYH11 | 7.2761675 | 8.913227 | 1.6370595 | 4.48E-07 | 8.57E-06 |
| ENSG00000109743 | BST1 | 8.8494271 | 10.485827 | 1.6364002 | 9.52E-12 | 4.40E-10 |
| ENSG00000121594 | CD80 | 7.4472291 | 9.079004 | 1.6317749 | 1.07E-14 | 7.93E-13 |
| ENSG00000087303 | NID2 | 3.2088314 | 4.8382776 | 1.6294462 | 8.65E-05 | 0.0009114 |
| ENSG00000025708 | TYMP | 11.412749 | 13.039342 | 1.6265928 | 3.29E-14 | 2.27E-12 |
| ENSG00000169122 | FAM110B | 3.9820527 | 5.6084056 | 1.6263529 | 0.0039145 | 0.0232736 |
| ENSG00000168461 | RAB31 | 11.331722 | 12.95717 | 1.6254474 | 0.0016459 | 0.0112766 |
| ENSG00000143771 | CNIH4 | 8.5300325 | 10.155068 | 1.6250357 | 5.14E-14 | 3.41E-12 |
| ENSG00000278588 | HIST1H2BI | 5.548585 | 7.1717805 | 1.6231955 | 3.24E-05 | 0.000386 |
| ENSG00000127530 | OR7C1 | 2.0729425 | 3.6955118 | 1.6225694 | 0.0034883 | 0.0211423 |
| ENSG00000229183 | PGA4 | 1.7874341 | 3.4034466 | 1.6160125 | 0.0001777 | 0.001698 |
| ENSG00000112303 | VNN2 | 10.137555 | 11.751305 | 1.6137506 | 7.05E-11 | 2.77E-09 |
| ENSG00000123342 | MMP19 | 10.007642 | 11.620656 | 1.613014 | 2.06E-13 | 1.24E-11 |
| ENSG00000090376 | IRAK3 | 12.053031 | 13.664741 | 1.6117101 | 2.79E-49 | 6.44E-46 |
| ENSG00000196781 | TLE1 | 6.5525672 | 8.1631578 | 1.6105907 | 4.13E-12 | 2.00E-10 |
| ENSG00000205086 | C2orf91 | 0 | 1.6092317 | 1.6092317 | 0.0010453 | 0.0076889 |
| ENSG00000161921 | CXCL16 | 11.629631 | 13.238574 | 1.6089424 | 5.07E-29 | 2.02E-26 |
| ENSG00000182310 | SPACA6 | 7.3000845 | 8.906394 | 1.6063096 | 8.83E-07 | 1.57E-05 |
| ENSG00000074181 | NOTCH3 | 8.9887073 | 10.592248 | 1.6035403 | 1.36E-11 | 6.09E-10 |
| ENSG00000183153 | GJD3 | 4.6738912 | 6.2772561 | 1.6033649 | 7.60E-07 | 1.38E-05 |
| ENSG00000254087 | LYN | 12.760369 | 14.363363 | 1.6029942 | 7.09E-37 | 6.09E-34 |
| ENSG00000186583 | SPATC1 | 7.308448 | 8.9092028 | 1.6007549 | 2.71E-10 | 9.74E-09 |
| ENSG00000104290 | FZD3 | 5.6949614 | 7.2948829 | 1.5999215 | 2.30E-06 | 3.69E-05 |
| ENSG00000168214 | RBPJ | 11.805105 | 13.402712 | 1.597607 | 1.25E-16 | 1.23E-14 |
| ENSG00000168899 | VAMP5 | 6.3093393 | 7.9058121 | 1.5964728 | 4.52E-10 | 1.56E-08 |
| ENSG00000156966 | B3GNT7 | 6.7470457 | 8.3405213 | 1.5934756 | 0.0028663 | 0.0178622 |
| ENSG00000150048 | CLEC1A | 5.976403 | 7.5697056 | 1.5933026 | 2.81E-12 | 1.39E-10 |
| ENSG00000122884 | P4HA1 | 10.870592 | 12.461068 | 1.5904753 | 1.12E-29 | 4.79E-27 |
| ENSG00000126353 | CCR7 | 1.4146423 | 3.0043296 | 1.5896872 | 0.004399 | 0.0256666 |
| ENSG00000213892 | CEACAM16 | 2.472516 | 4.0589559 | 1.5864399 | 0.0007955 | 0.0060854 |
| ENSG00000161640 | SIGLEC11 | 5.0520429 | 6.6384012 | 1.5863583 | 0.0017224 | 0.0117051 |
| ENSG00000072694 | FCGR2B | 12.295906 | 13.881027 | 1.5851215 | 9.80E-10 | 3.19E-08 |
| ENSG00000102921 | N4BP1 | 11.701334 | 13.285432 | 1.5840976 | 8.74E-42 | 1.03E-38 |
| ENSG00000118473 | SGIP1 | 0.8148861 | 2.3989575 | 1.5840714 | 0.0052115 | 0.0293994 |
| ENSG00000157227 | MMP14 | 13.809949 | 15.392414 | 1.5824643 | 2.99E-24 | 7.27E-22 |
| ENSG00000106366 | SERPINE1 | 6.8498227 | 8.4309125 | 1.5810898 | 0.0006854 | 0.0053748 |
| ENSG00000148175 | STOM | 12.358116 | 13.938302 | 1.5801863 | 3.03E-33 | 1.95E-30 |
| ENSG00000185338 | SOCS1 | 3.1081482 | 4.6883141 | 1.5801659 | 0.0022053 | 0.0143363 |
| ENSG00000165030 | NFIL3 | 8.5624622 | 10.142616 | 1.5801539 | 4.65E-20 | 7.22E-18 |
| ENSG00000186047 | DLEU7 | 6.9853038 | 8.5614216 | 1.5761177 | 4.36E-16 | 3.99E-14 |
| ENSG00000272514 | CFAP206 | 1.5035547 | 3.0763631 | 1.5728084 | 0.0076383 | 0.0402285 |
| ENSG00000177575 | CD163 | 16.401902 | 17.97307 | 1.5711685 | 3.62E-08 | 8.79E-07 |
| ENSG00000242265 | PEG10 | 0.1834736 | 1.754533 | 1.5710594 | 0.0016525 | 0.0113162 |
| ENSG00000137507 | LRRC32 | 3.7306001 | 5.2958246 | 1.5652245 | 0.0015562 | 0.0107558 |
| ENSG00000121297 | TSHZ3 | 8.8730387 | 10.436397 | 1.5633584 | 2.49E-22 | 4.78E-20 |
| ENSG00000113389 | NPR3 | 0.8818697 | 2.4445212 | 1.5626515 | 0.0091128 | 0.0464294 |
| ENSG00000197747 | S100A10 | 10.752573 | 12.312676 | 1.5601036 | 5.62E-15 | 4.29E-13 |
| ENSG00000120318 | ARAP3 | 6.9433874 | 8.5018266 | 1.5584392 | 9.93E-05 | 0.001025 |
| ENSG00000165682 | CLEC1B | 4.4933505 | 6.0512145 | 1.557864 | 3.52E-05 | 0.0004144 |
| ENSG00000175354 | PTPN2 | 10.333187 | 11.887335 | 1.5541478 | 6.32E-20 | 9.54E-18 |
| ENSG00000163803 | PLB1 | 7.2537823 | 8.8058585 | 1.5520762 | 4.95E-09 | 1.41E-07 |
| ENSG00000151062 | CACNA2D4 | 8.300729 | 9.8526877 | 1.5519587 | 1.05E-15 | 9.11E-14 |
| ENSG00000007129 | CEACAM21 | 4.1581129 | 5.7061994 | 1.5480865 | 6.72E-05 | 0.0007297 |
| ENSG00000120217 | CD274 | 8.5496628 | 10.096044 | 1.546381 | 3.71E-05 | 0.0004349 |
| ENSG00000197093 | GAL3ST4 | 7.5582556 | 9.1043182 | 1.5460626 | 2.35E-06 | 3.76E-05 |
| ENSG00000203710 | CR1 | 12.380936 | 13.924375 | 1.5434393 | 8.06E-07 | 1.45E-05 |
| ENSG00000140795 | MYLK3 | 3.5260087 | 5.0689502 | 1.5429415 | 4.18E-08 | 1.00E-06 |
| ENSG00000198369 | SPRED2 | 8.8016816 | 10.341112 | 1.5394303 | 5.97E-08 | 1.39E-06 |
| ENSG00000011422 | PLAUR | 11.502289 | 13.038954 | 1.5366652 | 3.60E-13 | 2.09E-11 |
| ENSG00000179431 | FJX1 | 6.210395 | 7.7465028 | 1.5361077 | 8.94E-07 | 1.59E-05 |
| ENSG00000140807 | NKD1 | 4.0194759 | 5.5549926 | 1.5355167 | 1.21E-05 | 0.0001613 |
| ENSG00000213889 | PPM1N | 5.497238 | 7.0322848 | 1.5350468 | 7.58E-06 | 0.0001076 |
| ENSG00000007866 | TEAD3 | 2.2361328 | 3.7706255 | 1.5344927 | 0.0071919 | 0.038367 |
| ENSG00000147454 | SLC25A37 | 8.1934049 | 9.7272003 | 1.5337954 | 9.69E-09 | 2.63E-07 |
| ENSG00000123989 | CHPF | 4.6309729 | 6.1618039 | 1.530831 | 0.0017172 | 0.0116757 |
| ENSG00000232070 | TMEM253 | 1.689624 | 3.2198907 | 1.5302667 | 0.0014964 | 0.0104072 |
| ENSG00000136867 | SLC31A2 | 9.1070773 | 10.636686 | 1.529609 | 1.75E-61 | 8.50E-58 |
| ENSG00000111490 | TBC1D30 | 7.5967069 | 9.1247462 | 1.5280392 | 1.21E-11 | 5.48E-10 |
| ENSG00000111729 | CLEC4A | 9.7448172 | 11.271451 | 1.5266335 | 1.68E-29 | 7.08E-27 |
| ENSG00000013588 | GPRC5A | 1.4833452 | 3.0098632 | 1.5265179 | 0.0059982 | 0.0330042 |
| ENSG00000136541 | ERMN | 4.6352658 | 6.1546339 | 1.5193681 | 8.38E-09 | 2.29E-07 |
| ENSG00000136688 | IL36G | 0 | 1.518406 | 1.518406 | 0.0021506 | 0.0140479 |
| ENSG00000185262 | UBALD2 | 8.5017393 | 10.018717 | 1.5169777 | 7.14E-14 | 4.61E-12 |
| ENSG00000076356 | PLXNA2 | 7.9859713 | 9.5000819 | 1.5141106 | 2.74E-15 | 2.23E-13 |
| ENSG00000081041 | CXCL2 | 9.6417077 | 11.154205 | 1.5124968 | 6.39E-15 | 4.83E-13 |
| ENSG00000187116 | LILRA5 | 7.7883656 | 9.3004417 | 1.5120761 | 8.08E-08 | 1.83E-06 |
| ENSG00000175591 | P2RY2 | 5.6965931 | 7.2084311 | 1.5118379 | 1.27E-13 | 7.90E-12 |
| ENSG00000271079 | CTAGE15 | 1.9122514 | 3.4229004 | 1.5106491 | 2.17E-05 | 0.0002713 |
| ENSG00000180611 | MB21D2 | 8.0218883 | 9.5319517 | 1.5100634 | 5.07E-10 | 1.74E-08 |
| ENSG00000198088 | NUP62CL | 0 | 1.5087614 | 1.5087614 | 0.0016097 | 0.0110613 |
| ENSG00000184678 | HIST2H2BE | 8.2158779 | 9.7208067 | 1.5049288 | 1.46E-13 | 8.96E-12 |
| ENSG00000179403 | VWA1 | 0.2123925 | 1.7156622 | 1.5032697 | 0.0038885 | 0.0231513 |
| ENSG00000180776 | ZDHHC20 | 11.527794 | 13.029393 | 1.501599 | 1.80E-27 | 6.02E-25 |
| ENSG00000215114 | UBXN2B | 10.399373 | 11.900446 | 1.5010731 | 9.04E-19 | 1.18E-16 |
| ENSG00000228672 | PROB1 | 2.9570436 | 4.4515184 | 1.4944749 | 0.0028447 | 0.0177681 |
| ENSG00000125841 | NRSN2 | 5.6349961 | 7.1279124 | 1.4929163 | 2.77E-06 | 4.36E-05 |
| ENSG00000064393 | HIPK2 | 12.600541 | 14.092639 | 1.4920976 | 2.75E-27 | 8.92E-25 |
| ENSG00000174946 | GPR171 | 0.1925188 | 1.6838622 | 1.4913433 | 0.0029703 | 0.0184213 |
| ENSG00000126251 | GPR42 | 0.8283916 | 2.316493 | 1.4881014 | 0.0023747 | 0.015266 |
| ENSG00000027697 | IFNGR1 | 11.537093 | 13.022377 | 1.485284 | 1.29E-09 | 4.11E-08 |
| ENSG00000069974 | RAB27A | 9.2801214 | 10.765152 | 1.4850303 | 7.22E-28 | 2.54E-25 |
| ENSG00000184349 | EFNA5 | 0 | 1.4847781 | 1.4847781 | 0.003706 | 0.0222369 |
| ENSG00000164400 | CSF2 | 0.1672071 | 1.6459331 | 1.478726 | 0.0018223 | 0.0122642 |
| ENSG00000163746 | PLSCR2 | 1.0658411 | 2.5443738 | 1.4785327 | 0.0064788 | 0.0351929 |
| ENSG00000116260 | QSOX1 | 11.41654 | 12.893327 | 1.4767872 | 1.27E-17 | 1.43E-15 |
| ENSG00000151790 | TDO2 | 2.8936709 | 4.367882 | 1.4742111 | 0.0004 | 0.0033922 |
| ENSG00000164440 | TXLNB | 8.3588171 | 9.8273133 | 1.4684962 | 2.04E-06 | 3.33E-05 |
| ENSG00000113763 | UNC5A | 7.990504 | 9.458089 | 1.467585 | 0.0003012 | 0.0026723 |
| ENSG00000165935 | SMCO2 | 1.0297683 | 2.4906503 | 1.460882 | 0.0091917 | 0.0467495 |
| ENSG00000108684 | ASIC2 | 0.8206276 | 2.2812208 | 1.4605932 | 0.009738 | 0.049027 |
| ENSG00000000971 | CFH | 7.1753616 | 8.6317349 | 1.4563733 | 0.0002126 | 0.0019795 |
| ENSG00000153162 | BMP6 | 4.8254699 | 6.2804415 | 1.4549716 | 2.03E-06 | 3.32E-05 |
| ENSG00000123685 | BATF3 | 2.2605206 | 3.7137142 | 1.4531935 | 0.0017454 | 0.0118359 |
| ENSG00000249751 | ECSCR | 0 | 1.4526511 | 1.4526511 | 0.0037154 | 0.022281 |
| ENSG00000179241 | LDLRAD3 | 8.6832309 | 10.134525 | 1.4512941 | 1.85E-07 | 3.85E-06 |
| ENSG00000118094 | TREH | 2.0475159 | 3.4986153 | 1.4510993 | 0.0084755 | 0.0437895 |
| ENSG00000075399 | VPS9D1 | 8.5310902 | 9.9802145 | 1.4491243 | 4.58E-17 | 4.85E-15 |
| ENSG00000132718 | SYT11 | 9.756398 | 11.205489 | 1.4490907 | 3.36E-20 | 5.28E-18 |
| ENSG00000166507 | NDST2 | 9.7062442 | 11.154366 | 1.4481216 | 5.35E-15 | 4.12E-13 |
| ENSG00000117298 | ECE1 | 7.1938604 | 8.6348267 | 1.4409663 | 0.0007658 | 0.005902 |
| ENSG00000197930 | ERO1A | 11.519098 | 12.958794 | 1.4396954 | 9.22E-16 | 8.07E-14 |
| ENSG00000170385 | SLC30A1 | 11.874873 | 13.313823 | 1.4389502 | 3.54E-33 | 2.24E-30 |
| ENSG00000137693 | YAP1 | 0 | 1.4380006 | 1.4380006 | 0.005139 | 0.0290662 |
| ENSG00000133048 | CHI3L1 | 13.42847 | 14.866316 | 1.4378461 | 4.44E-05 | 0.0005101 |
| ENSG00000204021 | LIPK | 0.2399824 | 1.6758939 | 1.4359115 | 0.0010539 | 0.0077458 |
| ENSG00000001561 | ENPP4 | 9.4911162 | 10.926429 | 1.4353131 | 9.39E-18 | 1.07E-15 |
| ENSG00000205129 | C4orf47 | 1.2370747 | 2.670466 | 1.4333912 | 0.0056812 | 0.031542 |
| ENSG00000155158 | TTC39B | 7.6952843 | 9.1267245 | 1.4314402 | 0.0031283 | 0.0192725 |
| ENSG00000235910 | APOA1-AS | 4.3889072 | 5.8188821 | 1.4299749 | 1.34E-09 | 4.26E-08 |
| ENSG00000180549 | FUT7 | 5.1286466 | 6.5583651 | 1.4297186 | 1.09E-05 | 0.0001478 |
| ENSG00000102007 | PLP2 | 9.5064183 | 10.935841 | 1.4294222 | 2.47E-13 | 1.47E-11 |
| ENSG00000142166 | IFNAR1 | 12.603527 | 14.032764 | 1.4292373 | 6.57E-25 | 1.68E-22 |
| ENSG00000162746 | FCRLB | 5.4854999 | 6.9145735 | 1.4290736 | 2.06E-05 | 0.0002586 |
| ENSG00000147434 | CHRNA6 | 3.4177006 | 4.8462776 | 1.428577 | 0.0014209 | 0.009955 |
| ENSG00000139899 | CBLN3 | 7.1760271 | 8.6035854 | 1.4275583 | 8.85E-06 | 0.0001232 |
| ENSG00000184221 | OLIG1 | 3.0119143 | 4.439386 | 1.4274718 | 0.0001336 | 0.0013284 |
| ENSG00000068024 | HDAC4 | 9.5825624 | 11.00896 | 1.4263975 | 2.18E-15 | 1.79E-13 |
| ENSG00000277224 | HIST1H2BF | 7.1328371 | 8.5584632 | 1.4256261 | 1.46E-05 | 0.0001906 |
| ENSG00000161955 | TNFSF13 | 10.911758 | 12.335601 | 1.4238431 | 8.29E-20 | 1.23E-17 |
| ENSG00000197982 | C1orf122 | 7.8697252 | 9.2932642 | 1.423539 | 1.06E-06 | 1.85E-05 |
| ENSG00000159625 | DRC7 | 1.9734283 | 3.3956427 | 1.4222145 | 0.0018269 | 0.0122914 |
| ENSG00000129038 | LOXL1 | 0.2886651 | 1.7104535 | 1.4217884 | 0.0049666 | 0.0283054 |
| ENSG00000163251 | FZD5 | 9.4262786 | 10.846939 | 1.4206606 | 6.09E-15 | 4.63E-13 |
| ENSG00000181885 | CLDN7 | 5.7003569 | 7.1209517 | 1.4205948 | 9.50E-10 | 3.10E-08 |
| ENSG00000086300 | SNX10 | 10.349265 | 11.768942 | 1.4196769 | 3.11E-10 | 1.11E-08 |
| ENSG00000147036 | LANCL3 | 3.7696529 | 5.1872608 | 1.4176079 | 0.0006579 | 0.005188 |
| ENSG00000114757 | PEX5L | 3.1796189 | 4.596006 | 1.4163871 | 0.0003704 | 0.003184 |
| ENSG00000196569 | LAMA2 | 7.3332034 | 8.7469718 | 1.4137684 | 7.10E-11 | 2.79E-09 |
| ENSG00000144802 | NFKBIZ | 9.5869492 | 11.000169 | 1.4132201 | 4.74E-07 | 9.01E-06 |
| ENSG00000106780 | MEGF9 | 11.027294 | 12.438409 | 1.4111156 | 1.75E-20 | 2.79E-18 |
| ENSG00000254413 | CHKB-CPT1B | 7.5441047 | 8.9532372 | 1.4091326 | 3.11E-05 | 0.0003725 |
| ENSG00000165795 | NDRG2 | 5.888738 | 7.296639 | 1.407901 | 0.0004109 | 0.0034721 |
| ENSG00000155926 | SLA | 11.304327 | 12.711981 | 1.407654 | 8.64E-22 | 1.55E-19 |
| ENSG00000133561 | GIMAP6 | 8.4622256 | 9.8685822 | 1.4063565 | 2.49E-05 | 0.0003062 |
| ENSG00000177807 | KCNJ10 | 5.8507054 | 7.2560809 | 1.4053755 | 0.0054563 | 0.0305291 |
| ENSG00000180616 | SSTR2 | 6.2324085 | 7.6376781 | 1.4052696 | 0.0052813 | 0.029721 |
| ENSG00000068831 | RASGRP2 | 5.3679703 | 6.7709809 | 1.4030107 | 7.61E-05 | 0.0008159 |
| ENSG00000141526 | SLC16A3 | 11.621464 | 13.023932 | 1.4024676 | 4.34E-11 | 1.78E-09 |
| ENSG00000104881 | PPP1R13L | 4.193857 | 5.5956343 | 1.4017773 | 8.16E-05 | 0.0008662 |
| ENSG00000084764 | MAPRE3 | 7.88684 | 9.286071 | 1.399231 | 7.98E-22 | 1.44E-19 |
| ENSG00000177875 | CCDC184 | 0 | 1.398344 | 1.398344 | 0.0056439 | 0.0313706 |
| ENSG00000130590 | SAMD10 | 4.3478798 | 5.745519 | 1.3976392 | 9.80E-07 | 1.73E-05 |
| ENSG00000151952 | TMEM132D | 0 | 1.3973768 | 1.3973768 | 0.0066017 | 0.0357319 |
| ENSG00000273802 | HIST1H2BG | 6.6277688 | 8.0243352 | 1.3965664 | 3.46E-09 | 1.01E-07 |
| ENSG00000095794 | CREM | 8.6141878 | 10.006633 | 1.3924452 | 5.09E-19 | 6.88E-17 |
| ENSG00000160883 | HK3 | 11.392905 | 12.785345 | 1.39244 | 0.0003308 | 0.0028941 |
| ENSG00000203778 | FAM229B | 2.4838292 | 3.8758503 | 1.3920211 | 0.0041791 | 0.0246236 |
| ENSG00000203995 | ZYG11A | 0 | 1.3912845 | 1.3912845 | 0.0031932 | 0.0195981 |
| ENSG00000214274 | ANG | 6.8128502 | 8.2025701 | 1.3897199 | 7.45E-10 | 2.47E-08 |
| ENSG00000119938 | PPP1R3C | 0 | 1.3873169 | 1.3873169 | 0.0067547 | 0.0363962 |
| ENSG00000104419 | NDRG1 | 11.405815 | 12.790637 | 1.3848224 | 7.79E-16 | 6.88E-14 |
| ENSG00000176697 | BDNF | 2.95604 | 4.3386154 | 1.3825754 | 0.0001449 | 0.0014257 |
| ENSG00000029993 | HMGB3 | 7.2489213 | 8.6301397 | 1.3812184 | 4.95E-08 | 1.17E-06 |
| ENSG00000158457 | TSPAN33 | 9.6415803 | 11.021839 | 1.380259 | 2.94E-05 | 0.0003542 |
| ENSG00000197694 | SPTAN1 | 13.126271 | 14.506485 | 1.3802137 | 3.41E-24 | 8.19E-22 |
| ENSG00000168546 | GFRA2 | 4.2278934 | 5.6072288 | 1.3793353 | 0.0021823 | 0.0142233 |
| ENSG00000177181 | RIMKLA | 4.3743415 | 5.7511929 | 1.3768514 | 5.16E-05 | 0.0005815 |
| ENSG00000204815 | TTC25 | 4.0111324 | 5.3872651 | 1.3761327 | 2.67E-05 | 0.000324 |
| ENSG00000118785 | SPP1 | 15.589012 | 16.964287 | 1.3752744 | 3.19E-05 | 0.0003803 |
| ENSG00000196562 | SULF2 | 9.6688028 | 11.04237 | 1.3735671 | 3.16E-06 | 4.91E-05 |
| ENSG00000136167 | LCP1 | 14.388971 | 15.761366 | 1.3723956 | 1.18E-08 | 3.13E-07 |
| ENSG00000116544 | DLGAP3 | 0.1570644 | 1.5284921 | 1.3714277 | 0.0033781 | 0.0205598 |
| ENSG00000161638 | ITGA5 | 11.518754 | 12.885368 | 1.366614 | 5.92E-19 | 7.90E-17 |
| ENSG00000134853 | PDGFRA | 4.9157721 | 6.2798102 | 1.3640381 | 2.33E-05 | 0.0002886 |
| ENSG00000157933 | SKI | 10.298035 | 11.660037 | 1.3620021 | 5.90E-24 | 1.36E-21 |
| ENSG00000204195 | AWAT1 | 0.6500856 | 2.0110205 | 1.360935 | 0.0031238 | 0.0192478 |
| ENSG00000064932 | SBNO2 | 10.4168 | 11.777299 | 1.3604995 | 3.82E-14 | 2.59E-12 |
| ENSG00000064199 | SPA17 | 5.3387625 | 6.6953107 | 1.3565482 | 4.21E-13 | 2.40E-11 |
| ENSG00000151651 | ADAM8 | 9.4365852 | 10.793094 | 1.3565091 | 4.10E-07 | 7.92E-06 |
| ENSG00000158571 | PFKFB1 | 2.6682362 | 4.023895 | 1.3556589 | 0.000814 | 0.0062121 |
| ENSG00000198223 | CSF2RA | 10.295388 | 11.650045 | 1.3546565 | 1.10E-17 | 1.24E-15 |
| ENSG00000163462 | TRIM46 | 3.546417 | 4.8988728 | 1.3524558 | 9.83E-05 | 0.0010168 |
| ENSG00000135365 | PHF21A | 10.69089 | 12.041529 | 1.3506392 | 2.61E-11 | 1.11E-09 |
| ENSG00000189060 | H1F0 | 10.192676 | 11.541153 | 1.3484761 | 2.21E-06 | 3.57E-05 |
| ENSG00000123104 | ITPR2 | 14.105867 | 15.453852 | 1.3479853 | 3.36E-49 | 7.36E-46 |
| ENSG00000187240 | DYNC2H1 | 8.5994813 | 9.9470231 | 1.3475418 | 4.86E-17 | 5.09E-15 |
| ENSG00000102096 | PIM2 | 8.2180267 | 9.5655639 | 1.3475372 | 5.82E-09 | 1.64E-07 |
| ENSG00000115271 | GCA | 9.3335513 | 10.680903 | 1.347352 | 4.22E-28 | 1.53E-25 |
| ENSG00000257335 | MGAM | 5.6575541 | 7.0036112 | 1.3460571 | 9.98E-06 | 0.0001367 |
| ENSG00000186567 | CEACAM19 | 4.8026447 | 6.1481733 | 1.3455286 | 0.0003524 | 0.0030524 |
| ENSG00000107738 | VSIR | 11.021969 | 12.366372 | 1.3444034 | 3.70E-14 | 2.52E-12 |
| ENSG00000166927 | MS4A7 | 11.984607 | 13.328184 | 1.3435774 | 1.03E-13 | 6.54E-12 |
| ENSG00000146555 | SDK1 | 3.7399616 | 5.0818402 | 1.3418786 | 5.07E-05 | 0.0005739 |
| ENSG00000168386 | FILIP1L | 7.5552003 | 8.8945108 | 1.3393105 | 7.24E-07 | 1.32E-05 |
| ENSG00000122861 | PLAU | 12.395564 | 13.731756 | 1.336192 | 6.20E-07 | 1.15E-05 |
| ENSG00000277632 | CCL3 | 9.326323 | 10.662147 | 1.335824 | 0.0001586 | 0.001541 |
| ENSG00000163563 | MNDA | 11.144397 | 12.474414 | 1.3300176 | 3.62E-14 | 2.46E-12 |
| ENSG00000278463 | HIST1H2AB | 4.5833453 | 5.912642 | 1.3292966 | 0.000215 | 0.001999 |
| ENSG00000198121 | LPAR1 | 8.9663007 | 10.29431 | 1.328009 | 8.63E-10 | 2.84E-08 |
| ENSG00000145860 | RNF145 | 9.727159 | 11.051122 | 1.3239631 | 1.01E-09 | 3.28E-08 |
| ENSG00000113916 | BCL6 | 10.750677 | 12.072539 | 1.3218618 | 5.99E-12 | 2.85E-10 |
| ENSG00000075420 | FNDC3B | 13.05829 | 14.376549 | 1.3182585 | 1.12E-21 | 1.97E-19 |
| ENSG00000184271 | POU6F1 | 6.293164 | 7.6103395 | 1.3171755 | 0.0034954 | 0.0211735 |
| ENSG00000147894 | C9orf72 | 10.75575 | 12.072764 | 1.3170148 | 1.08E-16 | 1.07E-14 |
| ENSG00000106089 | STX1A | 5.0444243 | 6.3604057 | 1.3159814 | 3.65E-05 | 0.0004282 |
| ENSG00000124575 | HIST1H1D | 7.7049052 | 9.0205993 | 1.315694 | 0.0001027 | 0.0010554 |
| ENSG00000162897 | FCAMR | 0 | 1.3117083 | 1.3117083 | 0.0082092 | 0.042632 |
| ENSG00000143384 | MCL1 | 12.31598 | 13.624789 | 1.3088085 | 2.52E-18 | 3.12E-16 |
| ENSG00000188707 | ZBED6CL | 5.4700909 | 6.7784787 | 1.3083879 | 0.001933 | 0.0128786 |
| ENSG00000129673 | AANAT | 4.1055352 | 5.4077697 | 1.3022346 | 0.0007211 | 0.0056083 |
| ENSG00000158473 | CD1D | 5.103959 | 6.4057221 | 1.3017631 | 2.62E-05 | 0.0003189 |
| ENSG00000134755 | DSC2 | 10.9696 | 12.270464 | 1.3008639 | 1.83E-12 | 9.37E-11 |
| ENSG00000198719 | DLL1 | 7.0837835 | 8.3824404 | 1.2986569 | 2.01E-06 | 3.28E-05 |
| ENSG00000105339 | DENND3 | 11.472049 | 12.770176 | 1.2981263 | 1.41E-20 | 2.30E-18 |
| ENSG00000135503 | ACVR1B | 9.4840689 | 10.780488 | 1.2964189 | 2.12E-07 | 4.37E-06 |
| ENSG00000101665 | SMAD7 | 9.2776117 | 10.572961 | 1.2953494 | 2.41E-12 | 1.21E-10 |
| ENSG00000106701 | FSD1L | 8.599294 | 9.8941195 | 1.2948255 | 3.89E-09 | 1.13E-07 |
| ENSG00000109861 | CTSC | 14.583786 | 15.875001 | 1.2912142 | 3.47E-15 | 2.77E-13 |
| ENSG00000131873 | CHSY1 | 9.9729086 | 11.263564 | 1.2906552 | 8.75E-16 | 7.67E-14 |
| ENSG00000153214 | TMEM87B | 10.903922 | 12.193756 | 1.2898338 | 1.31E-17 | 1.47E-15 |
| ENSG00000163430 | FSTL1 | 2.3895961 | 3.6792276 | 1.2896316 | 0.0046944 | 0.0270516 |
| ENSG00000070018 | LRP6 | 3.1747663 | 4.4640455 | 1.2892792 | 0.0013763 | 0.0096937 |
| ENSG00000177383 | MAGEF1 | 5.9371447 | 7.2244071 | 1.2872624 | 1.24E-07 | 2.69E-06 |
| ENSG00000100368 | CSF2RB | 12.058847 | 13.34377 | 1.2849227 | 1.85E-24 | 4.56E-22 |
| ENSG00000065357 | DGKA | 8.8008318 | 10.084692 | 1.2838604 | 3.72E-09 | 1.08E-07 |
| ENSG00000102144 | PGK1 | 12.662066 | 13.945397 | 1.2833305 | 2.41E-13 | 1.43E-11 |
| ENSG00000171657 | GPR82 | 6.8864371 | 8.1684586 | 1.2820215 | 2.86E-05 | 0.0003444 |
| ENSG00000151715 | TMEM45B | 4.50343 | 5.7842924 | 1.2808624 | 2.58E-05 | 0.0003147 |
| ENSG00000179542 | SLITRK4 | 5.7384238 | 7.0181131 | 1.2796892 | 0.0001078 | 0.001102 |
| ENSG00000197321 | SVIL | 9.0638073 | 10.342194 | 1.2783864 | 6.18E-12 | 2.93E-10 |
| ENSG00000030419 | IKZF2 | 6.4891349 | 7.7674081 | 1.2782732 | 1.35E-06 | 2.31E-05 |
| ENSG00000183386 | FHL3 | 7.6288509 | 8.9063059 | 1.277455 | 2.05E-05 | 0.0002577 |
| ENSG00000131042 | LILRB2 | 11.471531 | 12.744615 | 1.2730837 | 9.61E-14 | 6.10E-12 |
| ENSG00000133454 | MYO18B | 0.431477 | 1.7006376 | 1.2691606 | 0.0095073 | 0.0481035 |
| ENSG00000274641 | HIST1H2BO | 5.2632788 | 6.5322709 | 1.2689922 | 0.0072553 | 0.0386024 |
| ENSG00000162817 | C1orf115 | 3.357529 | 4.6260366 | 1.2685076 | 0.0091167 | 0.0464387 |
| ENSG00000123096 | SSPN | 4.4761848 | 5.743546 | 1.2673612 | 9.17E-05 | 0.0009572 |
| ENSG00000116574 | RHOU | 10.220119 | 11.487335 | 1.2672162 | 1.24E-14 | 9.07E-13 |
| ENSG00000140564 | FURIN | 11.317091 | 12.584196 | 1.267105 | 3.78E-25 | 9.78E-23 |
| ENSG00000122786 | CALD1 | 3.2158002 | 4.4829018 | 1.2671016 | 0.0001645 | 0.0015909 |
| ENSG00000140022 | STON2 | 6.4075252 | 7.6734648 | 1.2659396 | 0.0003492 | 0.0030285 |
| ENSG00000058668 | ATP2B4 | 10.940418 | 12.206015 | 1.2655967 | 1.91E-07 | 3.98E-06 |
| ENSG00000103005 | USB1 | 9.9177944 | 11.181645 | 1.2638506 | 3.94E-21 | 6.63E-19 |
| ENSG00000107551 | RASSF4 | 13.100982 | 14.363157 | 1.2621753 | 2.89E-11 | 1.22E-09 |
| ENSG00000153885 | KCTD15 | 4.2937579 | 5.5545835 | 1.2608257 | 2.53E-06 | 4.00E-05 |
| ENSG00000101336 | HCK | 12.298382 | 13.558177 | 1.259795 | 2.10E-17 | 2.29E-15 |
| ENSG00000112290 | WASF1 | 6.1679535 | 7.426807 | 1.2588535 | 0.0002367 | 0.0021714 |
| ENSG00000243566 | UPK3B | 4.6754747 | 5.9340218 | 1.2585471 | 1.87E-05 | 0.0002371 |
| ENSG00000017260 | ATP2C1 | 11.416172 | 12.670368 | 1.2541964 | 7.33E-23 | 1.54E-20 |
| ENSG00000196118 | CCDC189 | 5.103799 | 6.3576075 | 1.2538085 | 4.48E-06 | 6.69E-05 |
| ENSG00000165507 | C10orf10 | 9.458788 | 10.710536 | 1.2517479 | 1.47E-06 | 2.50E-05 |
| ENSG00000105048 | TNNT1 | 4.5040168 | 5.7550698 | 1.251053 | 0.006306 | 0.0343896 |
| ENSG00000184584 | TMEM173 | 8.0380493 | 9.2877094 | 1.24966 | 4.34E-07 | 8.33E-06 |
| ENSG00000173530 | TNFRSF10D | 8.3900724 | 9.6384037 | 1.2483313 | 1.10E-05 | 0.0001484 |
| ENSG00000173846 | PLK3 | 9.0278036 | 10.275898 | 1.2480941 | 5.79E-08 | 1.35E-06 |
| ENSG00000135698 | MPHOSPH6 | 8.5308934 | 9.778406 | 1.2475127 | 2.89E-16 | 2.70E-14 |
| ENSG00000146700 | SSC4D | 4.6446845 | 5.8916103 | 1.2469257 | 1.42E-05 | 0.0001855 |
| ENSG00000164136 | IL15 | 7.1186574 | 8.3650154 | 1.2463581 | 1.04E-06 | 1.82E-05 |
| ENSG00000117228 | GBP1 | 8.5522303 | 9.7965825 | 1.2443523 | 3.74E-06 | 5.69E-05 |
| ENSG00000163291 | PAQR3 | 9.1397126 | 10.383512 | 1.2437989 | 0.0017766 | 0.0120114 |
| ENSG00000107968 | MAP3K8 | 10.718879 | 11.958886 | 1.240007 | 3.13E-13 | 1.83E-11 |
| ENSG00000085733 | CTTN | 9.1096843 | 10.349381 | 1.2396967 | 0.0015485 | 0.0107116 |
| ENSG00000178127 | NDUFV2 | 9.9990093 | 11.238002 | 1.2389923 | 1.97E-20 | 3.14E-18 |
| ENSG00000113532 | ST8SIA4 | 11.161655 | 12.400363 | 1.2387079 | 1.06E-08 | 2.85E-07 |
| ENSG00000143515 | ATP8B2 | 6.346396 | 7.5840264 | 1.2376304 | 0.0005267 | 0.004284 |
| ENSG00000162645 | GBP2 | 9.6908512 | 10.928076 | 1.2372253 | 1.33E-14 | 9.70E-13 |
| ENSG00000229894 | GK3P | 6.8946482 | 8.1284452 | 1.233797 | 1.55E-12 | 8.00E-11 |
| ENSG00000177663 | IL17RA | 12.871208 | 14.103178 | 1.2319705 | 5.46E-21 | 9.06E-19 |
| ENSG00000056972 | TRAF3IP2 | 7.4023905 | 8.6343428 | 1.2319523 | 7.15E-08 | 1.63E-06 |
| ENSG00000185650 | ZFP36L1 | 12.742825 | 13.973622 | 1.230797 | 2.66E-07 | 5.38E-06 |
| ENSG00000002933 | TMEM176A | 11.441938 | 12.672391 | 1.2304535 | 1.62E-08 | 4.20E-07 |
| ENSG00000112796 | ENPP5 | 1.5132326 | 2.7430822 | 1.2298496 | 0.0087392 | 0.0448656 |
| ENSG00000115165 | CYTIP | 10.00292 | 11.232455 | 1.2295349 | 2.38E-09 | 7.22E-08 |
| ENSG00000198682 | PAPSS2 | 10.020124 | 11.249184 | 1.2290609 | 6.94E-12 | 3.27E-10 |
| ENSG00000146376 | ARHGAP18 | 12.816063 | 14.044885 | 1.2288219 | 9.75E-32 | 5.20E-29 |
| ENSG00000135407 | AVIL | 7.8967369 | 9.1252761 | 1.2285391 | 4.60E-06 | 6.85E-05 |
| ENSG00000127838 | PNKD | 12.08593 | 13.314332 | 1.2284022 | 1.28E-11 | 5.74E-10 |
| ENSG00000142583 | SLC2A5 | 9.4013858 | 10.627792 | 1.2264064 | 0.0025788 | 0.0163616 |
| ENSG00000122729 | ACO1 | 10.980763 | 12.206686 | 1.225923 | 4.56E-15 | 3.56E-13 |
| ENSG00000125505 | MBOAT7 | 9.8146433 | 11.040296 | 1.2256522 | 1.26E-14 | 9.18E-13 |
| ENSG00000254122 | PCDHGB7 | 6.0278636 | 7.251621 | 1.2237574 | 0.0011668 | 0.0084418 |
| ENSG00000159388 | BTG2 | 11.149515 | 12.372288 | 1.2227731 | 1.76E-11 | 7.68E-10 |
| ENSG00000090339 | ICAM1 | 12.915428 | 14.135148 | 1.2197203 | 2.66E-13 | 1.56E-11 |
| ENSG00000130766 | SESN2 | 8.4941011 | 9.7130899 | 1.2189888 | 7.68E-08 | 1.74E-06 |
| ENSG00000059728 | MXD1 | 11.234597 | 12.450917 | 1.2163203 | 0.0055616 | 0.0310115 |
| ENSG00000159216 | RUNX1 | 11.057509 | 12.27372 | 1.2162104 | 7.83E-17 | 8.02E-15 |
| ENSG00000125744 | RTN2 | 7.4672999 | 8.6826397 | 1.2153398 | 2.30E-12 | 1.16E-10 |
| ENSG00000148677 | ANKRD1 | 1.8507599 | 3.065843 | 1.2150831 | 0.0052293 | 0.0294851 |
| ENSG00000165168 | CYBB | 15.553348 | 16.765015 | 1.2116669 | 5.00E-13 | 2.81E-11 |
| ENSG00000105501 | SIGLEC5 | 8.6582539 | 9.8689834 | 1.2107296 | 3.86E-06 | 5.86E-05 |
| ENSG00000011105 | TSPAN9 | 3.9081128 | 5.1185329 | 1.2104201 | 0.0003605 | 0.0031119 |
| ENSG00000124257 | NEURL2 | 3.0237396 | 4.2333041 | 1.2095645 | 0.0061434 | 0.0336506 |
| ENSG00000181690 | PLAG1 | 5.7966632 | 7.0057547 | 1.2090915 | 0.0001463 | 0.0014367 |
| ENSG00000142089 | IFITM3 | 9.4174137 | 10.626298 | 1.2088841 | 2.80E-05 | 0.0003382 |
| ENSG00000131969 | ABHD12B | 6.3424213 | 7.5505706 | 1.2081494 | 7.79E-06 | 0.00011 |
| ENSG00000112033 | PPARD | 11.306415 | 12.51214 | 1.2057251 | 7.55E-13 | 4.12E-11 |
| ENSG00000028137 | TNFRSF1B | 13.164116 | 14.369281 | 1.2051648 | 1.80E-08 | 4.63E-07 |
| ENSG00000240694 | PNMA2 | 6.3938968 | 7.599059 | 1.2051622 | 1.43E-05 | 0.0001865 |
| ENSG00000167600 | CYP2S1 | 9.5454343 | 10.75029 | 1.2048554 | 4.97E-09 | 1.41E-07 |
| ENSG00000115590 | IL1R2 | 5.498683 | 6.7033795 | 1.2046965 | 9.64E-05 | 0.0009994 |
| ENSG00000127311 | HELB | 8.9715462 | 10.175347 | 1.2038012 | 1.38E-09 | 4.38E-08 |
| ENSG00000108309 | RUNDC3A | 3.9531949 | 5.1560347 | 1.2028398 | 3.26E-05 | 0.0003877 |
| ENSG00000102897 | LYRM1 | 7.7070159 | 8.909473 | 1.2024571 | 1.09E-12 | 5.79E-11 |
| ENSG00000101017 | CD40 | 8.9889238 | 10.191374 | 1.2024501 | 7.26E-08 | 1.65E-06 |
| ENSG00000278828 | HIST1H3H | 5.3290461 | 6.5309643 | 1.2019182 | 0.0016088 | 0.011057 |
| ENSG00000161835 | GRASP | 4.6612914 | 5.8622261 | 1.2009346 | 6.47E-06 | 9.28E-05 |
| ENSG00000087253 | LPCAT2 | 9.4395927 | 10.640002 | 1.2004092 | 3.69E-08 | 8.92E-07 |
| ENSG00000136603 | SKIL | 10.815505 | 12.010693 | 1.1951884 | 2.18E-08 | 5.51E-07 |
| ENSG00000152207 | CYSLTR2 | 6.3341661 | 7.5292012 | 1.1950352 | 0.0008309 | 0.0063181 |
| ENSG00000273604 | EPOP | 7.795241 | 8.9900954 | 1.1948544 | 0.0001555 | 0.0015147 |
| ENSG00000167851 | CD300A | 8.2123043 | 9.4065263 | 1.1942221 | 0.0002841 | 0.0025424 |
| ENSG00000158270 | COLEC12 | 12.445146 | 13.637353 | 1.1922071 | 2.97E-08 | 7.33E-07 |
| ENSG00000100644 | HIF1A | 14.338348 | 15.530181 | 1.1918325 | 9.09E-08 | 2.04E-06 |
| ENSG00000185697 | MYBL1 | 4.0539735 | 5.2436288 | 1.1896553 | 0.0003074 | 0.0027182 |
| ENSG00000019144 | PHLDB1 | 8.3734353 | 9.5606421 | 1.1872068 | 4.03E-06 | 6.09E-05 |
| ENSG00000198805 | PNP | 8.5637515 | 9.7492401 | 1.1854887 | 7.30E-06 | 0.000104 |
| ENSG00000135862 | LAMC1 | 10.980379 | 12.16495 | 1.1845708 | 2.99E-08 | 7.38E-07 |
| ENSG00000177469 | CAVIN1 | 6.962603 | 8.1470625 | 1.1844594 | 0.0025557 | 0.0162484 |
| ENSG00000005249 | PRKAR2B | 6.8446199 | 8.028068 | 1.1834481 | 1.54E-06 | 2.59E-05 |
| ENSG00000087589 | CASS4 | 9.0250679 | 10.208161 | 1.1830934 | 1.50E-06 | 2.54E-05 |
| ENSG00000137491 | SLCO2B1 | 14.238716 | 15.421299 | 1.1825824 | 1.59E-10 | 5.87E-09 |
| ENSG00000144668 | ITGA9 | 7.0175469 | 8.1995642 | 1.1820172 | 5.18E-05 | 0.0005837 |
| ENSG00000005379 | TSPOAP1 | 6.0653283 | 7.2467688 | 1.1814405 | 2.21E-06 | 3.57E-05 |
| ENSG00000111669 | TPI1 | 12.587494 | 13.767377 | 1.1798832 | 6.61E-13 | 3.64E-11 |
| ENSG00000162723 | SLAMF9 | 6.0342191 | 7.2110317 | 1.1768127 | 0.0017947 | 0.0120998 |
| ENSG00000172216 | CEBPB | 10.405986 | 11.581466 | 1.1754798 | 6.03E-06 | 8.72E-05 |
| ENSG00000109762 | SNX25 | 8.0052933 | 9.1792388 | 1.1739454 | 1.68E-16 | 1.63E-14 |
| ENSG00000187325 | TAF9B | 7.9926749 | 9.1661832 | 1.1735083 | 9.05E-07 | 1.60E-05 |
| ENSG00000111319 | SCNN1A | 3.2382018 | 4.4104171 | 1.1722153 | 0.0038666 | 0.0230398 |
| ENSG00000197457 | STMN3 | 4.0110673 | 5.1823297 | 1.1712625 | 0.0005709 | 0.0045897 |
| ENSG00000183734 | ASCL2 | 4.0085762 | 5.1779167 | 1.1693405 | 0.0031524 | 0.0193959 |
| ENSG00000187783 | TMEM72 | 4.2006341 | 5.3693234 | 1.1686894 | 0.0016423 | 0.0112553 |
| ENSG00000164292 | RHOBTB3 | 7.8006655 | 8.9674155 | 1.16675 | 5.76E-08 | 1.34E-06 |
| ENSG00000153815 | CMIP | 11.84608 | 13.011613 | 1.1655331 | 2.06E-14 | 1.46E-12 |
| ENSG00000091106 | NLRC4 | 9.4139376 | 10.579331 | 1.1653938 | 1.89E-14 | 1.34E-12 |
| ENSG00000244482 | LILRA6 | 10.110392 | 11.2755 | 1.1651076 | 3.74E-07 | 7.29E-06 |
| ENSG00000117009 | KMO | 9.1971989 | 10.358749 | 1.1615505 | 1.09E-07 | 2.40E-06 |
| ENSG00000138448 | ITGAV | 12.705094 | 13.866151 | 1.161057 | 2.67E-12 | 1.33E-10 |
| ENSG00000253873 | PCDHGA11 | 5.9617033 | 7.1220683 | 1.160365 | 0.0013999 | 0.0098327 |
| ENSG00000150773 | PIH1D2 | 4.2329871 | 5.391679 | 1.1586919 | 0.0016588 | 0.0113523 |
| ENSG00000151239 | TWF1 | 10.31138 | 11.468801 | 1.1574214 | 6.82E-12 | 3.22E-10 |
| ENSG00000176171 | BNIP3 | 9.7275132 | 10.883962 | 1.1564488 | 4.37E-09 | 1.26E-07 |
| ENSG00000081320 | STK17B | 10.555921 | 11.710154 | 1.1542328 | 1.98E-09 | 6.05E-08 |
| ENSG00000113966 | ARL6 | 5.5131738 | 6.6662227 | 1.153049 | 0.0013259 | 0.0093967 |
| ENSG00000042980 | ADAM28 | 11.436179 | 12.589022 | 1.1528426 | 1.03E-05 | 0.0001403 |
| ENSG00000197557 | TTC30A | 6.4925288 | 7.6452392 | 1.1527104 | 1.55E-13 | 9.46E-12 |
| ENSG00000198520 | C1orf228 | 3.7610418 | 4.9136529 | 1.1526112 | 0.0052928 | 0.029767 |
| ENSG00000086062 | B4GALT1 | 12.563889 | 13.716469 | 1.1525799 | 4.60E-33 | 2.88E-30 |
| ENSG00000141505 | ASGR1 | 6.6655314 | 7.8139633 | 1.1484319 | 0.0039686 | 0.0235386 |
| ENSG00000169508 | GPR183 | 10.368655 | 11.516257 | 1.1476025 | 6.84E-13 | 3.75E-11 |
| ENSG00000125910 | S1PR4 | 2.5684367 | 3.7158339 | 1.1473972 | 0.0018152 | 0.0122242 |
| ENSG00000079332 | SAR1A | 10.809783 | 11.956445 | 1.1466627 | 0.0001153 | 0.0011689 |
| ENSG00000198795 | ZNF521 | 4.5627601 | 5.7084122 | 1.1456521 | 8.31E-05 | 0.0008795 |
| ENSG00000073921 | PICALM | 13.162439 | 14.307688 | 1.1452487 | 1.31E-12 | 6.87E-11 |
| ENSG00000110435 | PDHX | 9.5832717 | 10.72819 | 1.1449181 | 8.61E-17 | 8.79E-15 |
| ENSG00000158373 | HIST1H2BD | 7.4898762 | 8.6336784 | 1.1438022 | 1.31E-07 | 2.84E-06 |
| ENSG00000091136 | LAMB1 | 6.7573698 | 7.8985276 | 1.1411578 | 5.12E-09 | 1.45E-07 |
| ENSG00000271605 | MILR1 | 9.8742203 | 11.015338 | 1.1411175 | 1.25E-12 | 6.56E-11 |
| ENSG00000177989 | ODF3B | 8.0658087 | 9.2058802 | 1.1400714 | 0.0003502 | 0.0030364 |
| ENSG00000275713 | HIST1H2BH | 5.8862506 | 7.0262134 | 1.1399627 | 4.62E-05 | 0.0005276 |
| ENSG00000188396 | TCTEX1D4 | 4.6233925 | 5.7629707 | 1.1395782 | 0.0050942 | 0.028873 |
| ENSG00000163235 | TGFA | 8.1989279 | 9.3368699 | 1.137942 | 1.78E-06 | 2.97E-05 |
| ENSG00000128271 | ADORA2A | 3.9713784 | 5.1087615 | 1.1373831 | 0.0041709 | 0.0245917 |
| ENSG00000110079 | MS4A4A | 10.527867 | 11.664939 | 1.1370726 | 1.65E-08 | 4.27E-07 |
| ENSG00000174749 | C4orf32 | 7.1734044 | 8.3102121 | 1.1368076 | 1.57E-06 | 2.65E-05 |
| ENSG00000068366 | ACSL4 | 11.684745 | 12.819412 | 1.1346671 | 1.13E-17 | 1.28E-15 |
| ENSG00000166780 | C16orf45 | 6.2735627 | 7.4076063 | 1.1340436 | 5.95E-06 | 8.62E-05 |
| ENSG00000166016 | ABTB2 | 5.2775575 | 6.4106176 | 1.13306 | 5.03E-07 | 9.48E-06 |
| ENSG00000126458 | RRAS | 9.3376679 | 10.470632 | 1.1329645 | 3.93E-13 | 2.26E-11 |
| ENSG00000125454 | SLC25A19 | 9.836164 | 10.966403 | 1.1302392 | 1.05E-12 | 5.61E-11 |
| ENSG00000173334 | TRIB1 | 10.903534 | 12.032744 | 1.1292097 | 4.57E-08 | 1.09E-06 |
| ENSG00000163833 | FBXO40 | 5.3430317 | 6.4721608 | 1.1291291 | 1.34E-05 | 0.0001776 |
| ENSG00000104067 | TJP1 | 6.9782847 | 8.1072253 | 1.1289406 | 1.84E-06 | 3.05E-05 |
| ENSG00000172243 | CLEC7A | 11.709701 | 12.837772 | 1.1280707 | 3.11E-15 | 2.51E-13 |
| ENSG00000099250 | NRP1 | 13.445813 | 14.573355 | 1.1275414 | 8.75E-13 | 4.73E-11 |
| ENSG00000138080 | EMILIN1 | 7.5897676 | 8.7167241 | 1.1269566 | 0.0048873 | 0.0279314 |
| ENSG00000109771 | LRP2BP | 6.322731 | 7.449685 | 1.126954 | 4.95E-06 | 7.30E-05 |
| ENSG00000166446 | CDYL2 | 10.226572 | 11.35213 | 1.1255577 | 5.70E-19 | 7.65E-17 |
| ENSG00000240972 | MIF | 8.3912523 | 9.5163161 | 1.1250638 | 1.48E-07 | 3.16E-06 |
| ENSG00000065911 | MTHFD2 | 10.364996 | 11.488789 | 1.1237935 | 1.93E-10 | 7.07E-09 |
| ENSG00000183307 | TMEM121B | 7.9608557 | 9.083452 | 1.1225963 | 8.47E-08 | 1.91E-06 |
| ENSG00000156011 | PSD3 | 10.127554 | 11.249322 | 1.1217681 | 0.0004308 | 0.0036194 |
| ENSG00000131979 | GCH1 | 7.283805 | 8.4051787 | 1.1213737 | 7.74E-05 | 0.0008277 |
| ENSG00000137393 | RNF144B | 11.543429 | 12.663771 | 1.1203417 | 4.29E-14 | 2.88E-12 |
| ENSG00000176595 | KBTBD11 | 4.1042064 | 5.2241356 | 1.1199293 | 0.0027806 | 0.0174227 |
| ENSG00000077238 | IL4R | 10.912073 | 12.031444 | 1.1193707 | 5.38E-10 | 1.84E-08 |
| ENSG00000275302 | CCL4 | 8.6235481 | 9.741944 | 1.1183959 | 0.0005638 | 0.0045414 |
| ENSG00000171631 | P2RY6 | 9.570844 | 10.688494 | 1.1176499 | 1.21E-11 | 5.48E-10 |
| ENSG00000155893 | PXYLP1 | 5.0769334 | 6.1940602 | 1.1171268 | 6.74E-08 | 1.55E-06 |
| ENSG00000114446 | IFT57 | 7.032741 | 8.1498331 | 1.1170921 | 3.30E-10 | 1.17E-08 |
| ENSG00000115107 | STEAP3 | 9.8476621 | 10.962738 | 1.1150757 | 6.30E-08 | 1.46E-06 |
| ENSG00000256812 | CAPNS2 | 4.8305941 | 5.945064 | 1.1144699 | 1.07E-05 | 0.0001454 |
| ENSG00000159399 | HK2 | 12.640951 | 13.754317 | 1.113366 | 1.18E-14 | 8.72E-13 |
| ENSG00000145779 | TNFAIP8 | 10.299122 | 11.411951 | 1.1128294 | 1.41E-12 | 7.37E-11 |
| ENSG00000084731 | KIF3C | 6.8975315 | 8.0088057 | 1.1112742 | 1.13E-08 | 3.01E-07 |
| ENSG00000158825 | CDA | 8.4128626 | 9.5221955 | 1.1093329 | 7.52E-05 | 0.0008066 |
| ENSG00000198959 | TGM2 | 11.347323 | 12.456052 | 1.1087288 | 0.0008383 | 0.0063682 |
| ENSG00000133639 | BTG1 | 11.12365 | 12.230621 | 1.1069709 | 0.0006333 | 0.0050143 |
| ENSG00000103313 | MEFV | 7.9424698 | 9.0493198 | 1.10685 | 1.45E-10 | 5.38E-09 |
| ENSG00000089351 | GRAMD1A | 11.16436 | 12.271128 | 1.1067686 | 4.26E-06 | 6.40E-05 |
| ENSG00000164466 | SFXN1 | 8.3416033 | 9.4479493 | 1.106346 | 1.03E-07 | 2.29E-06 |
| ENSG00000133794 | ARNTL | 9.4080475 | 10.512697 | 1.1046498 | 8.83E-22 | 1.58E-19 |
| ENSG00000116194 | ANGPTL1 | 5.2980101 | 6.3945277 | 1.0965176 | 6.57E-05 | 0.0007158 |
| ENSG00000253537 | PCDHGA7 | 2.1867476 | 3.2825583 | 1.0958107 | 0.0041264 | 0.0243651 |
| ENSG00000134686 | PHC2 | 10.909079 | 12.004634 | 1.0955555 | 1.34E-10 | 5.00E-09 |
| ENSG00000163874 | ZC3H12A | 9.0781799 | 10.173568 | 1.0953878 | 2.05E-05 | 0.0002577 |
| ENSG00000163697 | APBB2 | 7.7734916 | 8.8668682 | 1.0933766 | 0.0002196 | 0.0020349 |
| ENSG00000136048 | DRAM1 | 12.407381 | 13.499565 | 1.0921838 | 3.21E-12 | 1.56E-10 |
| ENSG00000186074 | CD300LF | 10.476293 | 11.565428 | 1.0891349 | 3.40E-15 | 2.71E-13 |
| ENSG00000112297 | CRYBG1 | 12.124405 | 13.210526 | 1.0861204 | 7.90E-07 | 1.43E-05 |
| ENSG00000174326 | SLC16A11 | 3.2388791 | 4.3248224 | 1.0859433 | 0.0078051 | 0.0409244 |
| ENSG00000164733 | CTSB | 18.263218 | 19.348911 | 1.0856937 | 1.54E-13 | 9.42E-12 |
| ENSG00000106991 | ENG | 12.588667 | 13.674069 | 1.0854018 | 4.85E-10 | 1.67E-08 |
| ENSG00000144061 | NPHP1 | 4.4660175 | 5.5494253 | 1.0834078 | 1.15E-05 | 0.0001545 |
| ENSG00000267534 | S1PR2 | 10.135373 | 11.217448 | 1.0820753 | 2.54E-12 | 1.27E-10 |
| ENSG00000158714 | SLAMF8 | 12.675264 | 13.757298 | 1.0820335 | 2.85E-14 | 1.99E-12 |
| ENSG00000154783 | FGD5 | 9.1500165 | 10.230396 | 1.0803798 | 7.12E-07 | 1.30E-05 |
| ENSG00000155307 | SAMSN1 | 11.885397 | 12.965177 | 1.0797806 | 3.24E-11 | 1.35E-09 |
| ENSG00000184988 | TMEM106A | 10.878626 | 11.956602 | 1.0779759 | 2.45E-11 | 1.05E-09 |
| ENSG00000139505 | MTMR6 | 11.041123 | 12.118219 | 1.0770966 | 2.84E-32 | 1.61E-29 |
| ENSG00000104812 | GYS1 | 10.522385 | 11.599221 | 1.0768357 | 1.02E-08 | 2.74E-07 |
| ENSG00000030304 | MUSK | 4.4388166 | 5.5152403 | 1.0764237 | 6.40E-06 | 9.20E-05 |
| ENSG00000129667 | RHBDF2 | 10.626411 | 11.702338 | 1.0759273 | 6.58E-07 | 1.21E-05 |
| ENSG00000168297 | PXK | 10.018511 | 11.091653 | 1.0731417 | 1.30E-13 | 8.08E-12 |
| ENSG00000129355 | CDKN2D | 4.8631824 | 5.9348974 | 1.0717149 | 5.15E-05 | 0.0005806 |
| ENSG00000121552 | CSTA | 8.4433559 | 9.5139582 | 1.0706023 | 3.07E-09 | 9.06E-08 |
| ENSG00000062716 | VMP1 | 12.929476 | 13.999927 | 1.0704513 | 1.52E-06 | 2.57E-05 |
| ENSG00000129219 | PLD2 | 9.5934217 | 10.663536 | 1.070114 | 4.22E-19 | 5.81E-17 |
| ENSG00000070540 | WIPI1 | 10.674097 | 11.743533 | 1.0694358 | 1.69E-14 | 1.21E-12 |
| ENSG00000131724 | IL13RA1 | 12.580859 | 13.650086 | 1.0692272 | 4.44E-19 | 6.09E-17 |
| ENSG00000149177 | PTPRJ | 13.069697 | 14.138116 | 1.0684186 | 3.30E-32 | 1.85E-29 |
| ENSG00000118985 | ELL2 | 12.753698 | 13.820793 | 1.0670951 | 4.17E-10 | 1.45E-08 |
| ENSG00000164604 | GPR85 | 6.305278 | 7.3722096 | 1.0669316 | 9.21E-05 | 0.0009609 |
| ENSG00000161653 | NAGS | 5.0904658 | 6.1570107 | 1.0665449 | 0.0001146 | 0.0011628 |
| ENSG00000105784 | RUNDC3B | 6.164194 | 7.2291693 | 1.0649752 | 0.0058369 | 0.0322468 |
| ENSG00000187837 | HIST1H1C | 10.290112 | 11.355043 | 1.0649315 | 2.88E-10 | 1.03E-08 |
| ENSG00000152763 | WDR78 | 4.8352997 | 5.8976262 | 1.0623265 | 8.19E-05 | 0.000869 |
| ENSG00000140368 | PSTPIP1 | 9.7175463 | 10.779076 | 1.0615295 | 1.09E-06 | 1.90E-05 |
| ENSG00000105122 | RASAL3 | 7.6040365 | 8.6652042 | 1.0611677 | 1.89E-06 | 3.12E-05 |
| ENSG00000165181 | C9orf84 | 9.0436706 | 10.103343 | 1.0596721 | 4.94E-06 | 7.29E-05 |
| ENSG00000102755 | FLT1 | 7.7450766 | 8.8025987 | 1.0575221 | 0.0025956 | 0.0164467 |
| ENSG00000225953 | SATB2-AS1 | 3.1121897 | 4.1688764 | 1.0566868 | 0.0091717 | 0.0466701 |
| ENSG00000154127 | UBASH3B | 11.164338 | 12.219789 | 1.0554516 | 1.85E-10 | 6.76E-09 |
| ENSG00000158869 | FCER1G | 12.777442 | 13.831535 | 1.0540933 | 1.81E-16 | 1.75E-14 |
| ENSG00000249992 | TMEM158 | 3.6423848 | 4.696265 | 1.0538801 | 0.0030556 | 0.0188856 |
| ENSG00000095303 | PTGS1 | 11.011228 | 12.062967 | 1.0517393 | 1.79E-14 | 1.28E-12 |
| ENSG00000185201 | IFITM2 | 7.0394231 | 8.0911108 | 1.0516878 | 0.005543 | 0.0309396 |
| ENSG00000196923 | PDLIM7 | 8.9713255 | 10.02256 | 1.0512348 | 4.29E-06 | 6.44E-05 |
| ENSG00000196712 | NF1 | 12.068516 | 13.119351 | 1.0508352 | 2.33E-22 | 4.52E-20 |
| ENSG00000145623 | OSMR | 5.4135187 | 6.463105 | 1.0495863 | 0.0002036 | 0.0019123 |
| ENSG00000074370 | ATP2A3 | 10.007695 | 11.057262 | 1.0495671 | 1.95E-06 | 3.20E-05 |
| ENSG00000141298 | SSH2 | 11.506031 | 12.555041 | 1.0490106 | 1.40E-15 | 1.19E-13 |
| ENSG00000049249 | TNFRSF9 | 7.1593134 | 8.2068398 | 1.0475263 | 0.0074551 | 0.0394771 |
| ENSG00000165169 | DYNLT3 | 9.6068582 | 10.653688 | 1.0468295 | 3.40E-06 | 5.23E-05 |
| ENSG00000064666 | CNN2 | 9.7649688 | 10.810891 | 1.0459217 | 4.44E-18 | 5.31E-16 |
| ENSG00000133574 | GIMAP4 | 10.081567 | 11.125866 | 1.044299 | 0.0029083 | 0.0180877 |
| ENSG00000109113 | RAB34 | 8.4820291 | 9.5255863 | 1.0435572 | 1.68E-06 | 2.82E-05 |
| ENSG00000081237 | PTPRC | 14.046705 | 15.08959 | 1.0428848 | 1.69E-15 | 1.41E-13 |
| ENSG00000145819 | ARHGAP26 | 12.218692 | 13.259129 | 1.0404374 | 4.21E-10 | 1.47E-08 |
| ENSG00000162783 | IER5 | 9.0987773 | 10.136644 | 1.0378671 | 0.0001074 | 0.0010982 |
| ENSG00000242616 | GNG10 | 9.8243268 | 10.861897 | 1.0375699 | 1.05E-10 | 3.98E-09 |
| ENSG00000100902 | PSMA6 | 10.469176 | 11.50575 | 1.0365742 | 3.61E-14 | 2.46E-12 |
| ENSG00000006459 | KDM7A | 12.267109 | 13.302131 | 1.0350227 | 2.16E-28 | 8.16E-26 |
| ENSG00000065989 | PDE4A | 10.423594 | 11.454932 | 1.0313387 | 3.27E-09 | 9.58E-08 |
| ENSG00000156313 | RPGR | 8.0158648 | 9.0456419 | 1.0297771 | 4.77E-11 | 1.94E-09 |
| ENSG00000168268 | NT5DC2 | 9.2565434 | 10.285219 | 1.0286752 | 2.52E-07 | 5.12E-06 |
| ENSG00000170854 | RIOX2 | 8.6807446 | 9.7093007 | 1.028556 | 7.60E-10 | 2.51E-08 |
| ENSG00000151151 | IPMK | 10.448713 | 11.476163 | 1.0274497 | 5.58E-14 | 3.66E-12 |
| ENSG00000054967 | RELT | 11.006936 | 12.033335 | 1.0263992 | 1.11E-14 | 8.20E-13 |
| ENSG00000136997 | MYC | 7.5048147 | 8.5295453 | 1.0247306 | 0.0011576 | 0.0083834 |
| ENSG00000145901 | TNIP1 | 11.851226 | 12.87529 | 1.0240643 | 5.34E-08 | 1.25E-06 |
| ENSG00000128578 | STRIP2 | 5.4016621 | 6.424819 | 1.0231569 | 0.0011864 | 0.0085572 |
| ENSG00000204389 | HSPA1A | 11.630793 | 12.652749 | 1.0219561 | 4.15E-05 | 0.0004803 |
| ENSG00000203814 | HIST2H2BF | 7.6918924 | 8.7122677 | 1.0203754 | 1.95E-07 | 4.05E-06 |
| ENSG00000179921 | GPBAR1 | 5.192702 | 6.2126233 | 1.0199213 | 0.0001715 | 0.0016463 |
| ENSG00000129353 | SLC44A2 | 9.1724852 | 10.190862 | 1.0183772 | 3.34E-07 | 6.59E-06 |
| ENSG00000175066 | GK5 | 9.1490686 | 10.166046 | 1.0169773 | 1.64E-05 | 0.0002114 |
| ENSG00000116032 | GRIN3B | 3.4879278 | 4.5038663 | 1.0159385 | 0.0015298 | 0.0106094 |
| ENSG00000130702 | LAMA5 | 4.1377473 | 5.1528025 | 1.0150552 | 1.52E-06 | 2.56E-05 |
| ENSG00000104177 | MYEF2 | 5.3352118 | 6.3476848 | 1.012473 | 0.0002231 | 0.0020635 |
| ENSG00000140030 | GPR65 | 10.304958 | 11.315414 | 1.0104555 | 3.66E-10 | 1.29E-08 |
| ENSG00000167460 | TPM4 | 12.901334 | 13.910981 | 1.0096468 | 8.81E-10 | 2.89E-08 |
| ENSG00000058056 | USP13 | 7.9012061 | 8.9071142 | 1.0059081 | 3.31E-07 | 6.55E-06 |
| ENSG00000196932 | TMEM26 | 9.0742536 | 10.080021 | 1.0057669 | 0.0001175 | 0.001187 |
| ENSG00000203812 | HIST2H2AA3 | 9.3256531 | 10.331358 | 1.0057054 | 8.79E-11 | 3.38E-09 |
| ENSG00000167613 | LAIR1 | 12.456726 | 13.462248 | 1.005522 | 1.31E-07 | 2.84E-06 |
| ENSG00000069399 | BCL3 | 9.1752585 | 10.180631 | 1.0053721 | 2.48E-06 | 3.94E-05 |
| ENSG00000026103 | FAS | 7.3578681 | 8.3625756 | 1.0047075 | 0.0001001 | 0.0010317 |
| ENSG00000099785 | MARCHF2 | 10.203423 | 11.207928 | 1.0045049 | 1.62E-07 | 3.42E-06 |
| ENSG00000139496 | NUP58 | 11.377878 | 12.382248 | 1.0043706 | 2.74E-17 | 2.94E-15 |
| ENSG00000272196 | HIST2H2AA4 | 9.3241506 | 10.328294 | 1.0041434 | 8.56E-11 | 3.30E-09 |
| ENSG00000002549 | LAP3 | 11.671823 | 12.675677 | 1.0038544 | 3.45E-11 | 1.44E-09 |
| ENSG00000163297 | ANTXR2 | 11.909237 | 12.912712 | 1.0034757 | 1.36E-08 | 3.58E-07 |
| ENSG00000008056 | SYN1 | 3.2968053 | 4.2997571 | 1.0029519 | 0.0031335 | 0.0192963 |
| ENSG00000125354 | SEPTIN6 | 9.9116155 | 10.914118 | 1.0025025 | 1.03E-08 | 2.77E-07 |
| ENSG00000136630 | HLX | 9.4400576 | 10.441565 | 1.0015071 | 7.78E-06 | 0.0001099 |
| ENSG00000172638 | EFEMP2 | 5.7400862 | 6.7409046 | 1.0008185 | 5.09E-05 | 0.0005752 |
| ENSG00000054965 | FAM168A | 11.870034 | 12.870482 | 1.0004479 | 4.12E-21 | 6.91E-19 |
| ENSG00000136367 | ZFHX2 | 7.4105162 | 8.4109589 | 1.0004427 | 1.25E-05 | 0.0001661 |
| ENSG00000141854 | MISP3 | 5.0258393 | 6.0256951 | 0.9998558 | 4.07E-05 | 0.000473 |
| ENSG00000104093 | DMXL2 | 15.036765 | 16.036158 | 0.999393 | 2.10E-17 | 2.29E-15 |
| ENSG00000214212 | C19orf38 | 6.5540133 | 7.5530816 | 0.9990684 | 0.0002602 | 0.0023523 |
| ENSG00000177535 | OR2B11 | 5.0202071 | 6.0192513 | 0.9990442 | 0.0003057 | 0.0027069 |
| ENSG00000174007 | CEP19 | 6.8803643 | 7.8790275 | 0.9986632 | 1.39E-06 | 2.37E-05 |
| ENSG00000179841 | AKAP5 | 5.9300258 | 6.9280806 | 0.9980548 | 0.0010437 | 0.0076783 |
| ENSG00000130052 | STARD8 | 9.7600862 | 10.758119 | 0.9980329 | 4.88E-08 | 1.16E-06 |
| ENSG00000109072 | VTN | 5.7356356 | 6.7329651 | 0.9973295 | 2.90E-06 | 4.55E-05 |
| ENSG00000140853 | NLRC5 | 10.037548 | 11.027838 | 0.9902901 | 7.31E-12 | 3.44E-10 |
| ENSG00000010818 | HIVEP2 | 9.7871045 | 10.771447 | 0.984342 | 2.33E-05 | 0.0002886 |
| ENSG00000116128 | BCL9 | 7.0787413 | 8.0628997 | 0.9841584 | 2.06E-08 | 5.25E-07 |
| ENSG00000183160 | TMEM119 | 7.3378146 | 8.3216698 | 0.9838552 | 0.0050397 | 0.0286491 |
| ENSG00000073792 | IGF2BP2 | 8.226936 | 9.2096182 | 0.9826823 | 2.18E-06 | 3.53E-05 |
| ENSG00000171189 | GRIK1 | 6.766913 | 7.7399789 | 0.9730659 | 7.05E-07 | 1.29E-05 |
| ENSG00000278677 | HIST1H2AM | 7.8085632 | 8.7815388 | 0.9729757 | 0.0003467 | 0.0030093 |
| ENSG00000182809 | CRIP2 | 3.0630505 | 4.0351543 | 0.9721038 | 0.0002729 | 0.0024531 |
| ENSG00000011201 | ANOS1 | 10.521526 | 11.493378 | 0.9718529 | 1.38E-11 | 6.13E-10 |
| ENSG00000134575 | ACP2 | 11.65996 | 12.63153 | 0.9715699 | 1.29E-10 | 4.87E-09 |
| ENSG00000188994 | ZNF292 | 11.612162 | 12.58156 | 0.9693982 | 5.21E-08 | 1.23E-06 |
| ENSG00000105464 | GRIN2D | 6.9298136 | 7.8943201 | 0.9645065 | 0.0009807 | 0.0072937 |
| ENSG00000183696 | UPP1 | 9.1670468 | 10.130882 | 0.9638356 | 2.44E-06 | 3.88E-05 |
| ENSG00000105996 | HOXA2 | 4.1701232 | 5.1339353 | 0.9638121 | 0.0003775 | 0.003236 |
| ENSG00000213390 | ARHGAP19 | 9.208101 | 10.171623 | 0.9635216 | 4.73E-11 | 1.93E-09 |
| ENSG00000221968 | FADS3 | 8.2731261 | 9.2366286 | 0.9635025 | 4.33E-08 | 1.03E-06 |
| ENSG00000110448 | CD5 | 6.6582016 | 7.6216575 | 0.9634559 | 0.0004621 | 0.0038431 |
| ENSG00000154640 | BTG3 | 7.5800987 | 8.5412855 | 0.9611868 | 8.71E-10 | 2.86E-08 |
| ENSG00000196396 | PTPN1 | 11.653926 | 12.614776 | 0.9608498 | 2.58E-32 | 1.49E-29 |
| ENSG00000185621 | LMLN | 8.2824245 | 9.2429606 | 0.9605361 | 2.62E-07 | 5.31E-06 |
| ENSG00000165071 | TMEM71 | 7.7293586 | 8.6875521 | 0.9581935 | 7.85E-05 | 0.0008376 |
| ENSG00000080200 | CRYBG3 | 11.676482 | 12.633674 | 0.9571922 | 3.82E-13 | 2.20E-11 |
| ENSG00000187037 | GPR141 | 9.9746135 | 10.931485 | 0.9568713 | 1.00E-15 | 8.74E-14 |
| ENSG00000258818 | RNASE4 | 7.6192419 | 8.574051 | 0.9548091 | 7.05E-05 | 0.0007628 |
| ENSG00000091073 | DTX2 | 10.033817 | 10.987486 | 0.9536694 | 4.10E-10 | 1.43E-08 |
| ENSG00000101974 | ATP11C | 9.9606275 | 10.914156 | 0.9535281 | 1.68E-09 | 5.20E-08 |
| ENSG00000122694 | GLIPR2 | 10.119315 | 11.070794 | 0.9514796 | 6.32E-07 | 1.17E-05 |
| ENSG00000181036 | FCRL6 | 6.1428951 | 7.093427 | 0.9505319 | 5.15E-05 | 0.0005811 |
| ENSG00000167680 | SEMA6B | 10.828688 | 11.777803 | 0.9491151 | 1.80E-05 | 0.0002293 |
| ENSG00000124140 | SLC12A5 | 5.4109966 | 6.3583926 | 0.947396 | 0.0002392 | 0.0021918 |
| ENSG00000091262 | ABCC6 | 6.4739648 | 7.4196875 | 0.9457226 | 7.67E-06 | 0.0001086 |
| ENSG00000160791 | CCR5 | 11.282167 | 12.226409 | 0.9442418 | 7.45E-05 | 0.0008006 |
| ENSG00000172575 | RASGRP1 | 7.552597 | 8.4950972 | 0.9425002 | 2.44E-05 | 0.000301 |
| ENSG00000162734 | PEA15 | 12.245499 | 13.187236 | 0.9417376 | 0.0003465 | 0.0030088 |
| ENSG00000169403 | PTAFR | 12.850456 | 13.789718 | 0.9392628 | 6.33E-11 | 2.52E-09 |
| ENSG00000092929 | UNC13D | 10.432226 | 11.371303 | 0.9390773 | 4.56E-06 | 6.80E-05 |
| ENSG00000037749 | MFAP3 | 8.5829066 | 9.5219376 | 0.939031 | 0.0010517 | 0.0077319 |
| ENSG00000187775 | DNAH17 | 7.6110345 | 8.5498854 | 0.9388509 | 2.66E-05 | 0.0003234 |
| ENSG00000056558 | TRAF1 | 7.8261961 | 8.7648405 | 0.9386444 | 2.67E-05 | 0.000324 |
| ENSG00000185222 | TCEAL9 | 7.7036614 | 8.6417037 | 0.9380423 | 5.25E-08 | 1.23E-06 |
| ENSG00000146476 | ARMT1 | 9.0469489 | 9.9849315 | 0.9379825 | 7.82E-05 | 0.0008344 |
| ENSG00000123700 | KCNJ2 | 8.2925195 | 9.2299793 | 0.9374598 | 0.0037308 | 0.0223511 |
| ENSG00000145730 | PAM | 10.626553 | 11.560487 | 0.9339338 | 9.40E-07 | 1.66E-05 |
| ENSG00000125753 | VASP | 10.889714 | 11.823174 | 0.9334598 | 5.58E-06 | 8.15E-05 |
| ENSG00000178980 | SELENOW | 9.044341 | 9.9766181 | 0.932277 | 2.30E-07 | 4.70E-06 |
| ENSG00000132589 | FLOT2 | 10.680558 | 11.611938 | 0.9313806 | 3.73E-14 | 2.53E-12 |
| ENSG00000107338 | SHB | 8.9400661 | 9.8709545 | 0.9308885 | 9.78E-09 | 2.65E-07 |
| ENSG00000162430 | SELENON | 11.627264 | 12.557474 | 0.9302099 | 1.72E-10 | 6.31E-09 |
| ENSG00000173744 | AGFG1 | 10.766738 | 11.69558 | 0.928842 | 7.42E-07 | 1.35E-05 |
| ENSG00000158874 | APOA2 | 6.471113 | 7.3968478 | 0.9257347 | 0.0001716 | 0.0016471 |
| ENSG00000183723 | CMTM4 | 8.6034291 | 9.5288007 | 0.9253716 | 1.54E-08 | 4.02E-07 |
| ENSG00000171314 | PGAM1 | 11.032476 | 11.95726 | 0.9247836 | 5.73E-10 | 1.94E-08 |
| ENSG00000125772 | GPCPD1 | 11.746138 | 12.66901 | 0.9228718 | 2.33E-06 | 3.74E-05 |
| ENSG00000171488 | LRRC8C | 10.84101 | 11.763324 | 0.9223132 | 4.09E-12 | 1.98E-10 |
| ENSG00000138756 | BMP2K | 13.686564 | 14.608598 | 0.9220339 | 1.88E-09 | 5.77E-08 |
| ENSG00000137364 | TPMT | 9.5950729 | 10.515654 | 0.9205814 | 4.43E-07 | 8.50E-06 |
| ENSG00000137747 | TMPRSS13 | 5.8379393 | 6.7571141 | 0.9191749 | 0.000472 | 0.0039095 |
| ENSG00000010810 | FYN | 7.335973 | 8.2545274 | 0.9185544 | 0.000389 | 0.0033174 |
| ENSG00000259330 | INAFM2 | 10.121583 | 11.039261 | 0.9176778 | 3.24E-12 | 1.58E-10 |
| ENSG00000198018 | ENTPD7 | 9.9904587 | 10.908091 | 0.9176319 | 5.30E-10 | 1.81E-08 |
| ENSG00000110047 | EHD1 | 8.8017914 | 9.719312 | 0.9175206 | 0.0069202 | 0.0371711 |
| ENSG00000116191 | RALGPS2 | 7.6857811 | 8.6032282 | 0.9174471 | 6.73E-10 | 2.25E-08 |
| ENSG00000166188 | ZNF319 | 8.9740699 | 9.8913543 | 0.9172843 | 4.92E-10 | 1.69E-08 |
| ENSG00000070087 | PFN2 | 6.7945586 | 7.711701 | 0.9171424 | 5.60E-05 | 0.000625 |
| ENSG00000197405 | C5AR1 | 12.768198 | 13.684164 | 0.9159662 | 2.14E-16 | 2.04E-14 |
| ENSG00000134243 | SORT1 | 11.617897 | 12.533726 | 0.9158294 | 1.77E-05 | 0.0002258 |
| ENSG00000067225 | PKM | 14.79009 | 15.705212 | 0.9151222 | 2.17E-09 | 6.61E-08 |
| ENSG00000149485 | FADS1 | 11.506379 | 12.420792 | 0.9144138 | 7.39E-07 | 1.35E-05 |
| ENSG00000127947 | PTPN12 | 11.924803 | 12.838127 | 0.9133246 | 1.69E-09 | 5.23E-08 |
| ENSG00000135046 | ANXA1 | 12.419997 | 13.332786 | 0.9127887 | 1.92E-09 | 5.89E-08 |
| ENSG00000108352 | RAPGEFL1 | 5.5021226 | 6.4146801 | 0.9125574 | 0.0004668 | 0.003875 |
| ENSG00000143119 | CD53 | 12.464339 | 13.376582 | 0.9122434 | 1.43E-12 | 7.42E-11 |
| ENSG00000106723 | SPIN1 | 9.931563 | 10.842309 | 0.9107461 | 0.0002803 | 0.002512 |
| ENSG00000147650 | LRP12 | 9.5238154 | 10.433124 | 0.9093083 | 1.17E-07 | 2.56E-06 |
| ENSG00000117016 | RIMS3 | 7.4380266 | 8.3445292 | 0.9065026 | 0.0027297 | 0.0171649 |
| ENSG00000172466 | ZNF24 | 10.658093 | 11.563929 | 0.9058368 | 0.0004966 | 0.0040803 |
| ENSG00000085514 | PILRA | 12.323751 | 13.229303 | 0.9055523 | 2.32E-18 | 2.89E-16 |
| ENSG00000118564 | FBXL5 | 11.251072 | 12.156479 | 0.9054071 | 1.42E-15 | 1.20E-13 |
| ENSG00000166750 | SLFN5 | 11.912694 | 12.817222 | 0.9045282 | 2.69E-09 | 8.05E-08 |
| ENSG00000134242 | PTPN22 | 10.003479 | 10.907505 | 0.9040259 | 5.52E-10 | 1.88E-08 |
| ENSG00000274290 | HIST1H2BE | 6.3865274 | 7.2903059 | 0.9037785 | 0.0010742 | 0.0078724 |
| ENSG00000126243 | LRFN3 | 4.6002383 | 5.5040032 | 0.9037649 | 0.0002305 | 0.0021243 |
| ENSG00000135766 | EGLN1 | 10.324073 | 11.226117 | 0.9020435 | 9.11E-12 | 4.23E-10 |
| ENSG00000079691 | CARMIL1 | 8.8457048 | 9.7472758 | 0.901571 | 3.15E-09 | 9.27E-08 |
| ENSG00000145685 | LHFPL2 | 13.474904 | 14.376106 | 0.9012022 | 2.74E-14 | 1.92E-12 |
| ENSG00000134851 | TMEM165 | 10.994596 | 11.895765 | 0.9011683 | 4.27E-15 | 3.36E-13 |
| ENSG00000162636 | FAM102B | 10.734797 | 11.635579 | 0.9007822 | 1.82E-14 | 1.30E-12 |
| ENSG00000163635 | ATXN7 | 10.169933 | 11.069981 | 0.900048 | 0.0001444 | 0.0014221 |
| ENSG00000275379 | HIST1H3I | 6.5103687 | 7.4084405 | 0.8980718 | 0.0002317 | 0.0021326 |
| ENSG00000235750 | KIAA0040 | 9.0145634 | 9.911781 | 0.8972176 | 8.07E-06 | 0.0001137 |
| ENSG00000170345 | FOS | 8.6841579 | 9.581314 | 0.8971561 | 0.0014145 | 0.0099135 |
| ENSG00000116678 | LEPR | 8.2453836 | 9.1412263 | 0.8958427 | 2.33E-05 | 0.0002886 |
| ENSG00000198363 | ASPH | 12.525904 | 13.421133 | 0.8952292 | 2.50E-09 | 7.53E-08 |
| ENSG00000182578 | CSF1R | 14.316144 | 15.210178 | 0.8940342 | 6.98E-10 | 2.33E-08 |
| ENSG00000164506 | STXBP5 | 10.879282 | 11.772313 | 0.8930315 | 3.13E-09 | 9.23E-08 |
| ENSG00000173988 | LRRC63 | 5.9234841 | 6.8157267 | 0.8922426 | 6.90E-05 | 0.0007484 |
| ENSG00000244115 | DNAJC25-GNG10 | 8.8247751 | 9.7157106 | 0.8909355 | 2.21E-06 | 3.57E-05 |
| ENSG00000142192 | APP | 12.616417 | 13.505536 | 0.8891185 | 9.38E-13 | 5.05E-11 |
| ENSG00000139133 | ALG10 | 7.3277118 | 8.216822 | 0.8891102 | 0.0007697 | 0.0059246 |
| ENSG00000124225 | PMEPA1 | 7.0299522 | 7.9189136 | 0.8889614 | 0.0001144 | 0.0011613 |
| ENSG00000166224 | SGPL1 | 13.486363 | 14.372163 | 0.8857996 | 1.52E-18 | 1.92E-16 |
| ENSG00000165312 | OTUD1 | 9.9913897 | 10.876981 | 0.8855914 | 1.15E-13 | 7.19E-12 |
| ENSG00000147459 | DOCK5 | 12.097821 | 12.981514 | 0.8836933 | 2.29E-15 | 1.88E-13 |
| ENSG00000186818 | LILRB4 | 13.439482 | 14.322932 | 0.8834495 | 1.98E-10 | 7.20E-09 |
| ENSG00000148841 | ITPRIP | 9.5453399 | 10.428633 | 0.883293 | 1.11E-05 | 0.00015 |
| ENSG00000035403 | VCL | 11.437154 | 12.319886 | 0.8827317 | 2.74E-09 | 8.18E-08 |
| ENSG00000102780 | DGKH | 9.9868896 | 10.868458 | 0.8815683 | 7.39E-06 | 0.0001052 |
| ENSG00000157193 | LRP8 | 7.8671684 | 8.7485281 | 0.8813597 | 0.0011283 | 0.0081959 |
| ENSG00000140577 | CRTC3 | 11.040466 | 11.919955 | 0.8794881 | 2.58E-24 | 6.35E-22 |
| ENSG00000136371 | MTHFS | 6.5716505 | 7.4511053 | 0.8794548 | 1.22E-05 | 0.000163 |
| ENSG00000180817 | PPA1 | 7.7408763 | 8.6198719 | 0.8789956 | 0.000184 | 0.0017509 |
| ENSG00000211445 | GPX3 | 8.7526366 | 9.6307035 | 0.8780669 | 0.0001345 | 0.0013361 |
| ENSG00000028116 | VRK2 | 9.0801987 | 9.9581635 | 0.8779648 | 1.77E-11 | 7.70E-10 |
| ENSG00000176597 | B3GNT5 | 8.6783696 | 9.5559953 | 0.8776258 | 6.40E-06 | 9.21E-05 |
| ENSG00000164953 | TMEM67 | 7.0402165 | 7.9175675 | 0.877351 | 2.50E-07 | 5.10E-06 |
| ENSG00000152422 | XRCC4 | 8.1297826 | 9.0071106 | 0.877328 | 4.17E-13 | 2.38E-11 |
| ENSG00000158423 | RIBC1 | 3.834258 | 4.7115623 | 0.8773043 | 0.0017481 | 0.0118525 |
| ENSG00000155629 | PIK3AP1 | 14.288661 | 15.164632 | 0.8759715 | 5.21E-30 | 2.40E-27 |
| ENSG00000132356 | PRKAA1 | 10.876771 | 11.751649 | 0.874878 | 1.58E-10 | 5.86E-09 |
| ENSG00000186174 | BCL9L | 10.415153 | 11.289257 | 0.8741041 | 1.20E-08 | 3.17E-07 |
| ENSG00000142657 | PGD | 13.498605 | 14.369397 | 0.870792 | 2.03E-25 | 5.42E-23 |
| ENSG00000277075 | HIST1H2AE | 8.7938988 | 9.6644789 | 0.8705801 | 6.48E-05 | 0.0007077 |
| ENSG00000136933 | RABEPK | 8.2116158 | 9.0818959 | 0.8702801 | 3.00E-09 | 8.87E-08 |
| ENSG00000157833 | GAREM2 | 3.734974 | 4.605225 | 0.870251 | 0.0057086 | 0.0316594 |
| ENSG00000173221 | GLRX | 11.105255 | 11.975356 | 0.8701016 | 1.36E-05 | 0.000179 |
| ENSG00000183255 | PTTG1IP | 12.5583 | 13.427979 | 0.8696783 | 7.33E-12 | 3.45E-10 |
| ENSG00000104921 | FCER2 | 5.5743306 | 6.443835 | 0.8695044 | 0.0081749 | 0.0424971 |
| ENSG00000188641 | DPYD | 12.478923 | 13.347534 | 0.868612 | 9.17E-19 | 1.19E-16 |
| ENSG00000103257 | SLC7A5 | 6.8524226 | 7.7171054 | 0.8646827 | 0.0012642 | 0.0090285 |
| ENSG00000243646 | IL10RB | 11.116815 | 11.980975 | 0.8641599 | 0.0001069 | 0.0010945 |
| ENSG00000042062 | RIPOR3 | 7.1995216 | 8.0621554 | 0.8626338 | 1.05E-05 | 0.0001435 |
| ENSG00000122417 | ODF2L | 8.4322317 | 9.2948404 | 0.8626087 | 3.80E-05 | 0.0004435 |
| ENSG00000175489 | LRRC25 | 11.380022 | 12.24211 | 0.862088 | 2.40E-07 | 4.89E-06 |
| ENSG00000163191 | S100A11 | 12.826447 | 13.687021 | 0.8605737 | 0.000213 | 0.0019834 |
| ENSG00000104343 | UBE2W | 10.193985 | 11.054443 | 0.860459 | 4.08E-09 | 1.18E-07 |
| ENSG00000169379 | ARL13B | 7.8550675 | 8.7140952 | 0.8590278 | 1.43E-09 | 4.52E-08 |
| ENSG00000106829 | TLE4 | 10.218005 | 11.076602 | 0.8585966 | 4.72E-18 | 5.63E-16 |
| ENSG00000122068 | FYTTD1 | 9.8536825 | 10.711324 | 0.8576411 | 0.0011551 | 0.0083668 |
| ENSG00000143554 | SLC27A3 | 7.9776037 | 8.8335852 | 0.8559815 | 0.0001274 | 0.0012762 |
| ENSG00000167642 | SPINT2 | 9.5526313 | 10.408191 | 0.8555596 | 0.0006247 | 0.0049558 |
| ENSG00000168769 | TET2 | 12.374167 | 13.2297 | 0.8555337 | 1.27E-09 | 4.05E-08 |
| ENSG00000176845 | METRNL | 10.536377 | 11.391103 | 0.8547258 | 3.68E-06 | 5.61E-05 |
| ENSG00000184922 | FMNL1 | 12.086492 | 12.941029 | 0.8545379 | 1.54E-09 | 4.81E-08 |
| ENSG00000144228 | SPOPL | 9.5608537 | 10.412159 | 0.8513054 | 9.54E-06 | 0.0001314 |
| ENSG00000185947 | ZNF267 | 11.096225 | 11.946527 | 0.8503027 | 1.37E-05 | 0.0001804 |
| ENSG00000145362 | ANK2 | 6.3671698 | 7.2164454 | 0.8492756 | 0.0010043 | 0.0074408 |
| ENSG00000161647 | MPP3 | 5.6884148 | 6.5376222 | 0.8492073 | 0.0044511 | 0.0258913 |
| ENSG00000138119 | MYOF | 11.859224 | 12.708407 | 0.8491838 | 0.0020682 | 0.0135974 |
| ENSG00000185090 | MANEAL | 5.5635497 | 6.4124074 | 0.8488577 | 0.0041605 | 0.0245366 |
| ENSG00000117533 | VAMP4 | 9.9134705 | 10.759855 | 0.8463844 | 1.21E-11 | 5.46E-10 |
| ENSG00000102057 | KCND1 | 4.8900248 | 5.7357129 | 0.8456881 | 0.0006239 | 0.0049499 |
| ENSG00000171310 | CHST11 | 12.386768 | 13.23113 | 0.8443617 | 4.15E-06 | 6.26E-05 |
| ENSG00000165046 | LETM2 | 4.5380292 | 5.3823781 | 0.8443489 | 0.00185 | 0.0124181 |
| ENSG00000108854 | SMURF2 | 10.788353 | 11.63075 | 0.8423967 | 1.32E-12 | 6.89E-11 |
| ENSG00000075213 | SEMA3A | 8.4439614 | 9.2858046 | 0.8418432 | 0.0012609 | 0.0090072 |
| ENSG00000178719 | GRINA | 11.844745 | 12.686048 | 0.8413031 | 2.37E-07 | 4.84E-06 |
| ENSG00000167470 | MIDN | 10.157783 | 10.998527 | 0.8407436 | 0.0001098 | 0.0011187 |
| ENSG00000072401 | UBE2D1 | 10.614314 | 11.455019 | 0.8407053 | 2.40E-13 | 1.43E-11 |
| ENSG00000146457 | WTAP | 11.296912 | 12.137464 | 0.8405526 | 1.32E-07 | 2.84E-06 |
| ENSG00000204406 | MBD5 | 9.8288528 | 10.66802 | 0.8391675 | 5.28E-12 | 2.53E-10 |
| ENSG00000156642 | NPTN | 11.510668 | 12.349833 | 0.8391646 | 6.42E-18 | 7.46E-16 |
| ENSG00000183722 | LHFPL6 | 5.008105 | 5.8463557 | 0.8382507 | 0.0014283 | 0.0100003 |
| ENSG00000204673 | AKT1S1 | 10.15073 | 10.988719 | 0.8379887 | 1.50E-07 | 3.20E-06 |
| ENSG00000106546 | AHR | 12.434719 | 13.271637 | 0.8369178 | 5.18E-08 | 1.22E-06 |
| ENSG00000116514 | RNF19B | 11.094334 | 11.929886 | 0.8355526 | 3.93E-08 | 9.48E-07 |
| ENSG00000157350 | ST3GAL2 | 10.433527 | 11.268624 | 0.8350977 | 2.16E-06 | 3.50E-05 |
| ENSG00000183741 | CBX6 | 10.928642 | 11.762348 | 0.8337062 | 0.000114 | 0.0011577 |
| ENSG00000163513 | TGFBR2 | 12.936279 | 13.768319 | 0.8320399 | 3.78E-15 | 2.99E-13 |
| ENSG00000255823 | MTRNR2L8 | 7.387269 | 8.2190716 | 0.8318027 | 2.98E-06 | 4.67E-05 |
| ENSG00000087586 | AURKA | 7.3021732 | 8.1333559 | 0.8311827 | 9.61E-08 | 2.14E-06 |
| ENSG00000155966 | AFF2 | 5.108558 | 5.9392206 | 0.8306626 | 0.00017 | 0.0016355 |
| ENSG00000174791 | RIN1 | 6.9307591 | 7.7600021 | 0.829243 | 3.69E-06 | 5.63E-05 |
| ENSG00000180353 | HCLS1 | 12.740367 | 13.569123 | 0.8287552 | 4.16E-14 | 2.80E-12 |
| ENSG00000198513 | ATL1 | 5.8553345 | 6.6809674 | 0.8256329 | 0.0008517 | 0.0064583 |
| ENSG00000203965 | EFCAB7 | 6.2423533 | 7.0670442 | 0.8246909 | 0.0002361 | 0.0021677 |
| ENSG00000100266 | PACSIN2 | 10.967936 | 11.792075 | 0.8241388 | 6.19E-07 | 1.15E-05 |
| ENSG00000180773 | SLC36A4 | 10.12387 | 10.947345 | 0.823475 | 8.22E-10 | 2.71E-08 |
| ENSG00000100889 | PCK2 | 10.386592 | 11.209366 | 0.8227739 | 3.33E-05 | 0.0003953 |
| ENSG00000188868 | ZNF563 | 5.5651955 | 6.3861537 | 0.8209582 | 0.0004785 | 0.0039545 |
| ENSG00000276547 | PCDHGB5 | 6.0711214 | 6.8918422 | 0.8207208 | 0.0021782 | 0.0142011 |
| ENSG00000159128 | IFNGR2 | 12.186896 | 13.00759 | 0.8206945 | 5.56E-08 | 1.30E-06 |
| ENSG00000222009 | BTBD19 | 8.5796761 | 9.4001364 | 0.8204603 | 0.0045658 | 0.0264214 |
| ENSG00000075223 | SEMA3C | 9.9757376 | 10.795662 | 0.8199244 | 2.53E-06 | 4.02E-05 |
| ENSG00000137078 | SIT1 | 4.4106056 | 5.229904 | 0.8192983 | 0.0015404 | 0.0106655 |
| ENSG00000174238 | PITPNA | 11.730489 | 12.547218 | 0.8167293 | 3.66E-05 | 0.0004293 |
| ENSG00000151726 | ACSL1 | 12.284824 | 13.101268 | 0.8164439 | 1.80E-06 | 2.98E-05 |
| ENSG00000130449 | ZSWIM6 | 11.486787 | 12.302878 | 0.8160911 | 1.59E-12 | 8.19E-11 |
| ENSG00000136490 | LIMD2 | 8.7632755 | 9.5783845 | 0.815109 | 0.0008734 | 0.0066028 |
| ENSG00000163602 | RYBP | 9.9473128 | 10.762347 | 0.8150343 | 6.98E-10 | 2.33E-08 |
| ENSG00000075624 | ACTB | 16.563129 | 17.376318 | 0.8131882 | 0.0007246 | 0.0056311 |
| ENSG00000212916 | MAP10 | 6.4421051 | 7.2543505 | 0.8122454 | 0.000749 | 0.0057948 |
| ENSG00000183484 | GPR132 | 8.0181784 | 8.8294444 | 0.811266 | 3.94E-05 | 0.0004592 |
| ENSG00000181722 | ZBTB20 | 10.041125 | 10.851257 | 0.8101322 | 0.0014143 | 0.0099135 |
| ENSG00000213190 | MLLT11 | 8.7837124 | 9.5936304 | 0.809918 | 5.28E-09 | 1.49E-07 |
| ENSG00000117000 | RLF | 10.651154 | 11.460851 | 0.8096975 | 3.85E-08 | 9.31E-07 |
| ENSG00000167123 | CERCAM | 8.0963608 | 8.9058228 | 0.809462 | 0.0033034 | 0.0201639 |
| ENSG00000135245 | HILPDA | 6.5907737 | 7.3999903 | 0.8092166 | 0.0017512 | 0.0118664 |
| ENSG00000177666 | PNPLA2 | 9.9466753 | 10.755666 | 0.808991 | 0.0001002 | 0.0010324 |
| ENSG00000188001 | TPRG1 | 9.1987143 | 10.005799 | 0.8070844 | 4.22E-05 | 0.0004869 |
| ENSG00000086730 | LAT2 | 10.402804 | 11.209272 | 0.8064681 | 3.91E-07 | 7.57E-06 |
| ENSG00000204388 | HSPA1B | 10.866335 | 11.672049 | 0.8057136 | 0.0050574 | 0.0287268 |
| ENSG00000155903 | RASA2 | 10.237272 | 11.04209 | 0.8048181 | 3.04E-07 | 6.08E-06 |
| ENSG00000121350 | PYROXD1 | 9.6177393 | 10.421508 | 0.8037684 | 0.0003121 | 0.0027537 |
| ENSG00000179029 | TMEM107 | 7.7638565 | 8.5671995 | 0.803343 | 0.0003619 | 0.0031213 |
| ENSG00000133805 | AMPD3 | 12.192825 | 12.995771 | 0.8029457 | 2.10E-06 | 3.41E-05 |
| ENSG00000125462 | C1orf61 | 3.7813255 | 4.5835234 | 0.8021978 | 0.0077443 | 0.0406691 |
| ENSG00000151748 | SAV1 | 9.3339607 | 10.135351 | 0.8013904 | 2.67E-10 | 9.58E-09 |
| ENSG00000184260 | HIST2H2AC | 9.6576956 | 10.457988 | 0.8002926 | 6.15E-10 | 2.07E-08 |
| ENSG00000178750 | STX19 | 4.4643262 | 5.263996 | 0.7996698 | 0.007498 | 0.0396517 |
| ENSG00000113441 | LNPEP | 12.430919 | 13.229754 | 0.7988354 | 4.42E-14 | 2.95E-12 |
| ENSG00000135049 | AGTPBP1 | 10.761686 | 11.560452 | 0.7987653 | 1.22E-09 | 3.92E-08 |
| ENSG00000163466 | ARPC2 | 13.227487 | 14.025475 | 0.7979884 | 7.36E-11 | 2.88E-09 |
| ENSG00000117643 | MAN1C1 | 9.210167 | 10.006456 | 0.7962888 | 0.0070427 | 0.0377129 |
| ENSG00000143190 | POU2F1 | 10.768164 | 11.563896 | 0.7957313 | 0.0005447 | 0.0044065 |
| ENSG00000187391 | MAGI2 | 6.6212574 | 7.4163188 | 0.7950614 | 0.0059226 | 0.0326666 |
| ENSG00000169902 | TPST1 | 6.3093909 | 7.1039951 | 0.7946043 | 0.0029503 | 0.0183177 |
| ENSG00000134046 | MBD2 | 11.588278 | 12.381718 | 0.7934398 | 3.96E-05 | 0.0004606 |
| ENSG00000177674 | AGTRAP | 8.5798598 | 9.3726389 | 0.7927792 | 2.59E-05 | 0.0003157 |
| ENSG00000166002 | SMCO4 | 7.3006101 | 8.0914571 | 0.790847 | 1.89E-06 | 3.12E-05 |
| ENSG00000143322 | ABL2 | 12.183657 | 12.973158 | 0.7895009 | 8.97E-08 | 2.01E-06 |
| ENSG00000148459 | PDSS1 | 8.0407869 | 8.8299699 | 0.7891829 | 1.99E-06 | 3.26E-05 |
| ENSG00000104946 | TBC1D17 | 10.616222 | 11.404985 | 0.7887624 | 1.32E-07 | 2.84E-06 |
| ENSG00000115392 | FANCL | 7.1407932 | 7.9293694 | 0.7885762 | 0.0001127 | 0.0011462 |
| ENSG00000148690 | FRA10AC1 | 8.0630026 | 8.8485745 | 0.7855719 | 0.0014353 | 0.0100363 |
| ENSG00000175505 | CLCF1 | 5.4827021 | 6.2682274 | 0.7855254 | 0.0021324 | 0.0139482 |
| ENSG00000173598 | NUDT4 | 9.5499275 | 10.333537 | 0.7836097 | 0.0003052 | 0.0027035 |
| ENSG00000182197 | EXT1 | 6.5515355 | 7.334892 | 0.7833565 | 0.0041971 | 0.0247161 |
| ENSG00000185664 | PMEL | 4.2218541 | 5.00402 | 0.7821658 | 0.007478 | 0.0395698 |
| ENSG00000175105 | ZNF654 | 9.9731426 | 10.755139 | 0.7819962 | 7.29E-09 | 2.01E-07 |
| ENSG00000155657 | TTN | 11.062009 | 11.843548 | 0.7815386 | 1.58E-09 | 4.92E-08 |
| ENSG00000179104 | TMTC2 | 7.7248445 | 8.5063343 | 0.7814898 | 2.36E-06 | 3.78E-05 |
| ENSG00000166664 | CHRFAM7A | 7.3804581 | 8.1609081 | 0.7804501 | 3.26E-05 | 0.0003884 |
| ENSG00000166128 | RAB8B | 11.312674 | 12.092812 | 0.7801374 | 3.83E-10 | 1.34E-08 |
| ENSG00000180596 | HIST1H2BC | 9.9112819 | 10.691153 | 0.7798715 | 1.25E-06 | 2.15E-05 |
| ENSG00000153250 | RBMS1 | 11.758479 | 12.537912 | 0.7794331 | 4.53E-07 | 8.65E-06 |
| ENSG00000142235 | LMTK3 | 3.450979 | 4.2302565 | 0.7792775 | 0.0001153 | 0.0011688 |
| ENSG00000120129 | DUSP1 | 8.328641 | 9.1069072 | 0.7782662 | 0.0032015 | 0.0196408 |
| ENSG00000113583 | C5orf15 | 9.9237539 | 10.70169 | 0.7779366 | 1.38E-07 | 2.96E-06 |
| ENSG00000150938 | CRIM1 | 11.259805 | 12.037417 | 0.777612 | 0.0034725 | 0.0210604 |
| ENSG00000257093 | KIAA1147 | 12.755556 | 13.532445 | 0.7768893 | 1.65E-14 | 1.19E-12 |
| ENSG00000237765 | FAM200B | 8.2223935 | 8.9980324 | 0.775639 | 5.75E-06 | 8.38E-05 |
| ENSG00000080823 | MOK | 6.0580556 | 6.8334329 | 0.7753773 | 0.0025738 | 0.0163442 |
| ENSG00000139323 | POC1B | 9.3406138 | 10.115978 | 0.7753646 | 7.08E-08 | 1.62E-06 |
| ENSG00000159176 | CSRP1 | 10.717213 | 11.492112 | 0.7748989 | 0.0012258 | 0.0087965 |
| ENSG00000181004 | BBS12 | 6.9118356 | 7.6865763 | 0.7747407 | 0.0002604 | 0.0023537 |
| ENSG00000138496 | PARP9 | 10.839583 | 11.614026 | 0.7744431 | 4.25E-10 | 1.47E-08 |
| ENSG00000023902 | PLEKHO1 | 11.549771 | 12.323323 | 0.7735519 | 0.0003777 | 0.0032364 |
| ENSG00000106565 | TMEM176B | 12.804812 | 13.577478 | 0.7726657 | 0.0001389 | 0.0013739 |
| ENSG00000138175 | ARL3 | 8.3040426 | 9.076581 | 0.7725384 | 1.06E-07 | 2.35E-06 |
| ENSG00000164096 | C4orf3 | 10.074399 | 10.846049 | 0.7716507 | 0.0062559 | 0.0341645 |
| ENSG00000103528 | SYT17 | 8.4629134 | 9.2344204 | 0.771507 | 0.0009173 | 0.0068835 |
| ENSG00000132122 | SPATA6 | 7.8798036 | 8.6509035 | 0.7710999 | 9.62E-06 | 0.0001323 |
| ENSG00000270276 | HIST2H4B | 6.6722858 | 7.4429321 | 0.7706463 | 0.0015925 | 0.0109639 |
| ENSG00000180509 | KCNE1 | 8.8447935 | 9.6145156 | 0.7697221 | 0.0052564 | 0.0296151 |
| ENSG00000004478 | FKBP4 | 10.880828 | 11.649909 | 0.7690819 | 2.98E-08 | 7.37E-07 |
| ENSG00000006451 | RALA | 10.7343 | 11.50308 | 0.76878 | 0.0001235 | 0.0012409 |
| ENSG00000138101 | DTNB | 9.9219578 | 10.689788 | 0.7678301 | 0.0001487 | 0.0014563 |
| ENSG00000186806 | VSIG10L | 7.6194387 | 8.38717 | 0.7677313 | 6.94E-05 | 0.0007521 |
| ENSG00000146278 | PNRC1 | 12.174815 | 12.942255 | 0.7674408 | 1.55E-05 | 0.0002004 |
| ENSG00000172081 | MOB3A | 11.365093 | 12.132351 | 0.7672586 | 3.22E-09 | 9.46E-08 |
| ENSG00000134571 | MYBPC3 | 6.5955399 | 7.3619062 | 0.7663663 | 2.06E-05 | 0.0002592 |
| ENSG00000100504 | PYGL | 12.262691 | 13.028256 | 0.765565 | 6.44E-08 | 1.49E-06 |
| ENSG00000135926 | TMBIM1 | 12.573755 | 13.338211 | 0.7644564 | 1.20E-05 | 0.0001611 |
| ENSG00000158161 | EYA3 | 10.666313 | 11.430707 | 0.7643941 | 2.33E-09 | 7.07E-08 |
| ENSG00000163145 | C1QTNF7 | 4.2640043 | 5.0272642 | 0.7632599 | 0.0059591 | 0.0328342 |
| ENSG00000008311 | AASS | 6.5053608 | 7.2684422 | 0.7630814 | 0.0013731 | 0.0096774 |
| ENSG00000122707 | RECK | 7.4264756 | 8.1893982 | 0.7629225 | 2.81E-07 | 5.67E-06 |
| ENSG00000169188 | APEX2 | 9.1864795 | 9.9481566 | 0.7616772 | 2.14E-12 | 1.08E-10 |
| ENSG00000152213 | ARL11 | 9.9578225 | 10.719221 | 0.761399 | 1.12E-22 | 2.26E-20 |
| ENSG00000101350 | KIF3B | 9.8701752 | 10.630528 | 0.7603532 | 5.52E-18 | 6.50E-16 |
| ENSG00000249437 | NAIP | 12.468266 | 13.227649 | 0.7593832 | 3.47E-08 | 8.48E-07 |
| ENSG00000176170 | SPHK1 | 8.3980807 | 9.1567672 | 0.7586865 | 0.0046602 | 0.0268823 |
| ENSG00000168404 | MLKL | 9.7769626 | 10.53561 | 0.758647 | 1.13E-07 | 2.47E-06 |
| ENSG00000032444 | PNPLA6 | 12.013956 | 12.771765 | 0.7578092 | 1.27E-08 | 3.37E-07 |
| ENSG00000103507 | BCKDK | 10.415544 | 11.172231 | 0.7566867 | 4.31E-09 | 1.24E-07 |
| ENSG00000105514 | RAB3D | 10.291885 | 11.047664 | 0.7557788 | 2.39E-06 | 3.81E-05 |
| ENSG00000144566 | RAB5A | 10.737183 | 11.492564 | 0.7553806 | 2.55E-07 | 5.18E-06 |
| ENSG00000169756 | LIMS1 | 11.665994 | 12.420739 | 0.7547454 | 0.0053497 | 0.0300247 |
| ENSG00000204217 | BMPR2 | 12.102128 | 12.856045 | 0.7539175 | 5.62E-13 | 3.12E-11 |
| ENSG00000162927 | PUS10 | 8.8330919 | 9.5863545 | 0.7532626 | 4.12E-06 | 6.22E-05 |
| ENSG00000187231 | SESTD1 | 10.675392 | 11.42828 | 0.7528882 | 1.66E-07 | 3.49E-06 |
| ENSG00000137563 | GGH | 7.2492039 | 8.0012107 | 0.7520068 | 0.0005429 | 0.0043966 |
| ENSG00000095209 | TMEM38B | 8.7980116 | 9.5495944 | 0.7515827 | 0.0001221 | 0.0012291 |
| ENSG00000102362 | SYTL4 | 4.1296474 | 4.881221 | 0.7515736 | 0.0032745 | 0.0200209 |
| ENSG00000111254 | AKAP3 | 5.5401134 | 6.290971 | 0.7508576 | 0.0088194 | 0.0452033 |
| ENSG00000146858 | ZC3HAV1L | 5.397646 | 6.1483695 | 0.7507235 | 0.0082394 | 0.0427664 |
| ENSG00000196123 | KIAA0895L | 7.9919916 | 8.7423133 | 0.7503217 | 0.0002937 | 0.0026163 |
| ENSG00000125629 | INSIG2 | 8.8943551 | 9.6446217 | 0.7502666 | 0.0002475 | 0.002257 |
| ENSG00000068697 | LAPTM4A | 12.12575 | 12.875347 | 0.7495971 | 2.97E-10 | 1.06E-08 |
| ENSG00000186665 | C17orf58 | 6.0029384 | 6.7524431 | 0.7495047 | 0.0003872 | 0.0033039 |
| ENSG00000100916 | BRMS1L | 6.6767521 | 7.4245067 | 0.7477546 | 0.0001795 | 0.0017141 |
| ENSG00000187630 | DHRS4L2 | 7.9845752 | 8.7318842 | 0.747309 | 0.0072427 | 0.0385672 |
| ENSG00000106733 | NMRK1 | 9.4978554 | 10.244024 | 0.746169 | 5.94E-06 | 8.61E-05 |
| ENSG00000157077 | ZFYVE9 | 5.0268815 | 5.7729927 | 0.7461112 | 0.0087416 | 0.0448728 |
| ENSG00000134352 | IL6ST | 11.698254 | 12.444087 | 0.7458332 | 8.51E-11 | 3.29E-09 |
| ENSG00000111145 | ELK3 | 10.268241 | 11.013249 | 0.7450081 | 1.68E-09 | 5.20E-08 |
| ENSG00000072571 | HMMR | 5.7963092 | 6.5381186 | 0.7418094 | 0.0042697 | 0.0250492 |
| ENSG00000141753 | IGFBP4 | 10.178141 | 10.919005 | 0.740864 | 5.08E-05 | 0.0005746 |
| ENSG00000099814 | CEP170B | 7.7418001 | 8.4820101 | 0.7402099 | 0.0023074 | 0.0148878 |
| ENSG00000186645 | SPDYE17 | 3.3485202 | 4.0883173 | 0.7397971 | 0.0053079 | 0.029821 |
| ENSG00000169504 | CLIC4 | 12.308311 | 13.047904 | 0.7395922 | 1.32E-11 | 5.90E-10 |
| ENSG00000160584 | SIK3 | 10.971025 | 11.710597 | 0.7395719 | 3.32E-15 | 2.66E-13 |
| ENSG00000101745 | ANKRD12 | 11.396598 | 12.135718 | 0.73912 | 1.54E-09 | 4.81E-08 |
| ENSG00000128340 | RAC2 | 10.795547 | 11.534662 | 0.7391149 | 0.0083976 | 0.043459 |
| ENSG00000143641 | GALNT2 | 11.871831 | 12.610655 | 0.7388235 | 2.14E-08 | 5.41E-07 |
| ENSG00000135749 | PCNX2 | 7.6908018 | 8.4294898 | 0.738688 | 0.0005907 | 0.0047282 |
| ENSG00000079277 | MKNK1 | 11.913383 | 12.651286 | 0.7379039 | 3.55E-10 | 1.25E-08 |
| ENSG00000198843 | SELENOT | 11.130787 | 11.868327 | 0.7375395 | 4.57E-07 | 8.71E-06 |
| ENSG00000102349 | KLF8 | 7.1701897 | 7.9075472 | 0.7373575 | 0.0025506 | 0.0162208 |
| ENSG00000138413 | IDH1 | 13.050289 | 13.78711 | 0.7368208 | 7.65E-06 | 0.0001084 |
| ENSG00000167995 | BEST1 | 10.540671 | 11.277376 | 0.736705 | 1.70E-07 | 3.57E-06 |
| ENSG00000243660 | ZNF487 | 6.7512216 | 7.4870986 | 0.7358769 | 0.0004519 | 0.0037742 |
| ENSG00000185561 | TLCD2 | 6.6381489 | 7.3732596 | 0.7351107 | 0.00032 | 0.0028106 |
| ENSG00000138600 | SPPL2A | 11.980419 | 12.71534 | 0.7349214 | 9.75E-09 | 2.64E-07 |
| ENSG00000250510 | GPR162 | 7.7813752 | 8.515813 | 0.7344378 | 0.0044164 | 0.0257287 |
| ENSG00000131097 | HIGD1B | 3.5125344 | 4.2468648 | 0.7343304 | 0.0064073 | 0.0348565 |
| ENSG00000171223 | JUNB | 11.312219 | 12.046411 | 0.7341912 | 0.0012571 | 0.0089882 |
| ENSG00000137845 | ADAM10 | 13.158029 | 13.892108 | 0.7340789 | 6.12E-09 | 1.72E-07 |
| ENSG00000129071 | MBD4 | 10.399829 | 11.133097 | 0.7332683 | 7.69E-11 | 3.00E-09 |
| ENSG00000189308 | LIN54 | 9.7320257 | 10.464626 | 0.7325999 | 1.17E-11 | 5.33E-10 |
| ENSG00000178764 | ZHX2 | 9.1923805 | 9.924738 | 0.7323575 | 0.0001092 | 0.001114 |
| ENSG00000184900 | SUMO3 | 11.290866 | 12.022455 | 0.7315894 | 0.0056136 | 0.0312338 |
| ENSG00000125657 | TNFSF9 | 5.9299897 | 6.6614304 | 0.7314406 | 1.13E-05 | 0.0001526 |
| ENSG00000115421 | PAPOLG | 9.7376237 | 10.46892 | 0.7312963 | 2.34E-08 | 5.89E-07 |
| ENSG00000127824 | TUBA4A | 7.0903608 | 7.8215426 | 0.7311818 | 0.0042519 | 0.0249781 |
| ENSG00000170545 | SMAGP | 6.1333799 | 6.8641064 | 0.7307265 | 0.0005835 | 0.0046784 |
| ENSG00000121931 | LRIF1 | 8.2887548 | 9.0193451 | 0.7305903 | 6.00E-06 | 8.69E-05 |
| ENSG00000147168 | IL2RG | 9.4591566 | 10.18934 | 0.730183 | 0.0097456 | 0.0490598 |
| ENSG00000103145 | HCFC1R1 | 7.5995436 | 8.3271313 | 0.7275877 | 0.0014029 | 0.0098455 |
| ENSG00000104432 | IL7 | 6.2274065 | 6.9546266 | 0.7272201 | 0.0001649 | 0.0015944 |
| ENSG00000156959 | LHFPL4 | 5.1482244 | 5.8754069 | 0.7271825 | 0.002039 | 0.0134393 |
| ENSG00000188158 | NHS | 8.737067 | 9.4641681 | 0.7271011 | 9.98E-08 | 2.22E-06 |
| ENSG00000145365 | TIFA | 8.0420575 | 8.7690575 | 0.727 | 0.0002924 | 0.0026051 |
| ENSG00000110934 | BIN2 | 11.707024 | 12.433942 | 0.7269179 | 1.95E-16 | 1.86E-14 |
| ENSG00000175582 | RAB6A | 11.721737 | 12.447261 | 0.7255241 | 0.0005065 | 0.0041469 |
| ENSG00000167553 | TUBA1C | 11.759755 | 12.483184 | 0.7234283 | 2.49E-05 | 0.0003057 |
| ENSG00000058091 | CDK14 | 9.2073414 | 9.9306525 | 0.7233111 | 0.0002554 | 0.0023164 |
| ENSG00000136868 | SLC31A1 | 12.464874 | 13.18783 | 0.7229562 | 1.39E-08 | 3.64E-07 |
| ENSG00000138134 | STAMBPL1 | 4.7613516 | 5.4839685 | 0.7226169 | 0.0045938 | 0.0265662 |
| ENSG00000106034 | CPED1 | 10.485954 | 11.207707 | 0.7217536 | 6.41E-05 | 0.0007015 |
| ENSG00000126861 | OMG | 8.0247453 | 8.7452135 | 0.7204682 | 9.17E-06 | 0.000127 |
| ENSG00000170949 | ZNF160 | 10.005806 | 10.725975 | 0.7201687 | 2.10E-08 | 5.34E-07 |
| ENSG00000196792 | STRN3 | 9.9332453 | 10.653261 | 0.720016 | 1.84E-13 | 1.12E-11 |
| ENSG00000154217 | PITPNC1 | 9.4922702 | 10.211615 | 0.7193444 | 3.05E-08 | 7.50E-07 |
| ENSG00000196954 | CASP4 | 11.164472 | 11.883587 | 0.7191149 | 6.13E-07 | 1.14E-05 |
| ENSG00000188827 | SLX4 | 9.4446627 | 10.163771 | 0.7191084 | 6.02E-09 | 1.69E-07 |
| ENSG00000174442 | ZWILCH | 7.5078851 | 8.2269751 | 0.71909 | 2.76E-05 | 0.0003336 |
| ENSG00000132669 | RIN2 | 12.332371 | 13.049728 | 0.7173573 | 3.05E-06 | 4.75E-05 |
| ENSG00000148737 | TCF7L2 | 8.6649728 | 9.381722 | 0.7167491 | 0.0003332 | 0.0029111 |
| ENSG00000164442 | CITED2 | 11.000634 | 11.717369 | 0.7167355 | 0.0063301 | 0.034501 |
| ENSG00000074964 | ARHGEF10L | 11.758873 | 12.473514 | 0.7146408 | 3.63E-05 | 0.0004263 |
| ENSG00000137177 | KIF13A | 11.431306 | 12.145553 | 0.7142472 | 5.93E-26 | 1.63E-23 |
| ENSG00000197147 | LRRC8B | 9.2255093 | 9.9396164 | 0.7141071 | 9.88E-05 | 0.0010207 |
| ENSG00000197622 | CDC42SE1 | 11.734587 | 12.448408 | 0.7138219 | 2.91E-14 | 2.03E-12 |
| ENSG00000261794 | GOLGA8H | 7.3827691 | 8.0961264 | 0.7133572 | 0.0001583 | 0.001539 |
| ENSG00000145416 | MARCHF1 | 11.642034 | 12.355352 | 0.713318 | 0.0001193 | 0.0012024 |
| ENSG00000106772 | PRUNE2 | 6.9793684 | 7.6922099 | 0.7128414 | 0.00822 | 0.0426707 |
| ENSG00000123684 | LPGAT1 | 10.738727 | 11.449696 | 0.7109689 | 0.0064839 | 0.0352162 |
| ENSG00000101888 | NXT2 | 8.0241169 | 8.7347041 | 0.7105872 | 1.73E-05 | 0.0002212 |
| ENSG00000243749 | TMEM35B | 7.3197277 | 8.0299892 | 0.7102615 | 0.0087964 | 0.0451171 |
| ENSG00000179604 | CDC42EP4 | 6.892077 | 7.6020213 | 0.7099443 | 1.84E-05 | 0.0002344 |
| ENSG00000183726 | TMEM50A | 11.775803 | 12.483079 | 0.7072757 | 3.91E-13 | 2.25E-11 |
| ENSG00000163823 | CCR1 | 13.11398 | 13.820406 | 0.7064268 | 6.22E-13 | 3.43E-11 |
| ENSG00000114978 | MOB1A | 13.096943 | 13.802926 | 0.7059838 | 2.50E-09 | 7.53E-08 |
| ENSG00000054282 | SDCCAG8 | 10.613943 | 11.319711 | 0.7057681 | 1.12E-09 | 3.60E-08 |
| ENSG00000121741 | ZMYM2 | 12.263215 | 12.96866 | 0.7054449 | 4.25E-08 | 1.02E-06 |
| ENSG00000086619 | ERO1B | 9.9420121 | 10.647419 | 0.7054069 | 0.0001186 | 0.0011967 |
| ENSG00000136010 | ALDH1L2 | 8.1741325 | 8.8792452 | 0.7051127 | 0.0021427 | 0.014007 |
| ENSG00000139324 | TMTC3 | 9.0732996 | 9.7778494 | 0.7045498 | 2.21E-06 | 3.57E-05 |
| ENSG00000103811 | CTSH | 12.889489 | 13.59376 | 0.7042713 | 9.88E-07 | 1.74E-05 |
| ENSG00000160991 | ORAI2 | 9.2674303 | 9.9706787 | 0.7032484 | 0.0012052 | 0.0086697 |
| ENSG00000146373 | RNF217 | 9.9405674 | 10.643691 | 0.7031233 | 5.75E-07 | 1.07E-05 |
| ENSG00000167524 | SGK494 | 8.266622 | 8.9696019 | 0.7029799 | 5.55E-08 | 1.30E-06 |
| ENSG00000175970 | UNC119B | 8.573284 | 9.2760486 | 0.7027646 | 1.96E-06 | 3.22E-05 |
| ENSG00000132475 | H3F3B | 11.426763 | 12.126761 | 0.6999978 | 4.30E-11 | 1.77E-09 |
| ENSG00000127952 | STYXL1 | 8.2144899 | 8.9144219 | 0.699932 | 1.17E-08 | 3.10E-07 |
| ENSG00000198355 | PIM3 | 10.117741 | 10.815748 | 0.6980074 | 0.0047746 | 0.0273942 |
| ENSG00000145088 | EAF2 | 7.7469853 | 8.4443007 | 0.6973154 | 8.74E-05 | 0.0009182 |
| ENSG00000148288 | GBGT1 | 9.1333526 | 9.8305238 | 0.6971713 | 1.68E-17 | 1.85E-15 |
| ENSG00000058799 | YIPF1 | 9.5129531 | 10.210081 | 0.6971276 | 5.40E-11 | 2.17E-09 |
| ENSG00000136026 | CKAP4 | 10.259534 | 10.956632 | 0.6970989 | 0.0003199 | 0.00281 |
| ENSG00000109103 | UNC119 | 9.1494437 | 9.8463858 | 0.6969421 | 0.0003899 | 0.0033219 |
| ENSG00000176903 | PNMA1 | 10.198029 | 10.894918 | 0.6968885 | 2.45E-08 | 6.15E-07 |
| ENSG00000071073 | MGAT4A | 13.142101 | 13.838554 | 0.6964527 | 0.0031594 | 0.0194292 |
| ENSG00000143110 | C1orf162 | 11.103181 | 11.799548 | 0.6963666 | 3.01E-06 | 4.70E-05 |
| ENSG00000109083 | IFT20 | 7.1911717 | 7.8867175 | 0.6955458 | 4.92E-06 | 7.27E-05 |
| ENSG00000187118 | CMC1 | 8.6542372 | 9.3493664 | 0.6951291 | 0.0008792 | 0.0066389 |
| ENSG00000118217 | ATF6 | 12.11805 | 12.812768 | 0.6947176 | 2.59E-12 | 1.29E-10 |
| ENSG00000148848 | ADAM12 | 5.43242 | 6.1267158 | 0.6942957 | 0.0059059 | 0.0325861 |
| ENSG00000177272 | KCNA3 | 7.3728447 | 8.0669671 | 0.6941225 | 8.00E-05 | 0.0008515 |
| ENSG00000122970 | IFT81 | 7.0906701 | 7.7845037 | 0.6938335 | 0.0007176 | 0.0055863 |
| ENSG00000197381 | ADARB1 | 8.7740232 | 9.4676604 | 0.6936371 | 0.0055561 | 0.0309928 |
| ENSG00000050405 | LIMA1 | 9.5799139 | 10.273197 | 0.6932834 | 0.0002435 | 0.0022285 |
| ENSG00000129048 | ACKR4 | 5.3582126 | 6.0514535 | 0.6932409 | 0.002851 | 0.0177925 |
| ENSG00000143756 | FBXO28 | 9.956038 | 10.649029 | 0.6929908 | 0.0028659 | 0.0178621 |
| ENSG00000108582 | CPD | 12.256902 | 12.948969 | 0.6920667 | 8.27E-12 | 3.87E-10 |
| ENSG00000104973 | MED25 | 10.481575 | 11.172952 | 0.6913774 | 3.13E-14 | 2.17E-12 |
| ENSG00000058063 | ATP11B | 11.168825 | 11.859825 | 0.691 | 8.62E-10 | 2.84E-08 |
| ENSG00000105220 | GPI | 12.290324 | 12.980935 | 0.6906114 | 8.27E-07 | 1.48E-05 |
| ENSG00000144320 | LNPK | 11.076399 | 11.765689 | 0.6892909 | 1.01E-08 | 2.73E-07 |
| ENSG00000082074 | FYB1 | 13.165741 | 13.854742 | 0.6890017 | 1.16E-06 | 2.00E-05 |
| ENSG00000168298 | HIST1H1E | 9.7811399 | 10.469627 | 0.6884876 | 0.0001746 | 0.0016722 |
| ENSG00000100647 | SUSD6 | 12.716209 | 13.403863 | 0.6876544 | 2.25E-09 | 6.83E-08 |
| ENSG00000115170 | ACVR1 | 9.9236194 | 10.610785 | 0.687166 | 4.54E-09 | 1.30E-07 |
| ENSG00000196776 | CD47 | 11.670371 | 12.356868 | 0.6864972 | 1.82E-09 | 5.61E-08 |
| ENSG00000096433 | ITPR3 | 5.9139517 | 6.5999408 | 0.6859891 | 0.0016706 | 0.0114194 |
| ENSG00000079385 | CEACAM1 | 4.4459146 | 5.131232 | 0.6853174 | 0.0066747 | 0.0360425 |
| ENSG00000198804 | MT-CO1 | 17.441421 | 18.126603 | 0.6851819 | 0.0012975 | 0.0092335 |
| ENSG00000156050 | FAM161B | 8.5883061 | 9.2731678 | 0.6848617 | 5.66E-05 | 0.0006306 |
| ENSG00000114354 | TFG | 10.400652 | 11.085062 | 0.6844103 | 9.12E-09 | 2.48E-07 |
| ENSG00000188554 | NBR1 | 12.448421 | 13.131968 | 0.6835469 | 5.57E-14 | 3.66E-12 |
| ENSG00000165915 | SLC39A13 | 8.8677777 | 9.5507769 | 0.6829992 | 0.0011804 | 0.0085222 |
| ENSG00000119522 | DENND1A | 10.838571 | 11.520996 | 0.682425 | 6.78E-09 | 1.89E-07 |
| ENSG00000145817 | YIPF5 | 9.5020762 | 10.184279 | 0.6822029 | 0.0001095 | 0.0011167 |
| ENSG00000111679 | PTPN6 | 12.12469 | 12.806603 | 0.6819128 | 2.08E-07 | 4.29E-06 |
| ENSG00000134107 | BHLHE40 | 11.749374 | 12.431157 | 0.6817836 | 0.0001269 | 0.0012717 |
| ENSG00000073712 | FERMT2 | 5.8735077 | 6.5544249 | 0.6809172 | 0.0059117 | 0.0326105 |
| ENSG00000143228 | NUF2 | 4.7760544 | 5.4556418 | 0.6795874 | 0.0078213 | 0.0409971 |
| ENSG00000136021 | SCYL2 | 10.988464 | 11.666949 | 0.6784848 | 1.87E-09 | 5.75E-08 |
| ENSG00000123983 | ACSL3 | 11.387092 | 12.064686 | 0.6775942 | 3.35E-07 | 6.61E-06 |
| ENSG00000150782 | IL18 | 10.779865 | 11.457129 | 0.677264 | 0.0001409 | 0.0013904 |
| ENSG00000122557 | HERPUD2 | 10.283166 | 10.959604 | 0.6764386 | 1.18E-11 | 5.36E-10 |
| ENSG00000170832 | USP32 | 12.205502 | 12.88132 | 0.6758177 | 1.08E-11 | 4.96E-10 |
| ENSG00000119403 | PHF19 | 8.1056411 | 8.7813991 | 0.675758 | 0.0013435 | 0.0094958 |
| ENSG00000105767 | CADM4 | 4.2080464 | 4.8832478 | 0.6752014 | 0.0022303 | 0.0144654 |
| ENSG00000198668 | CALM1 | 12.322385 | 12.997541 | 0.6751565 | 0.005262 | 0.0296321 |
| ENSG00000162129 | CLPB | 10.49278 | 11.167058 | 0.6742787 | 1.26E-08 | 3.33E-07 |
| ENSG00000114268 | PFKFB4 | 10.104266 | 10.777721 | 0.6734545 | 0.0005167 | 0.004217 |
| ENSG00000101040 | ZMYND8 | 11.705467 | 12.378714 | 0.6732462 | 3.37E-18 | 4.08E-16 |
| ENSG00000141503 | MINK1 | 11.903359 | 12.576066 | 0.6727074 | 0.0001149 | 0.0011658 |
| ENSG00000197121 | PGAP1 | 7.2788698 | 7.9515499 | 0.6726801 | 0.005426 | 0.030391 |
| ENSG00000118292 | C1orf54 | 8.4709458 | 9.143534 | 0.6725881 | 5.11E-05 | 0.0005769 |
| ENSG00000170464 | DNAJC18 | 6.9585612 | 7.6311405 | 0.6725793 | 0.0008613 | 0.0065183 |
| ENSG00000149089 | APIP | 9.1761944 | 9.8487453 | 0.6725509 | 6.59E-05 | 0.0007177 |
| ENSG00000168264 | IRF2BP2 | 11.558242 | 12.228902 | 0.67066 | 5.07E-07 | 9.54E-06 |
| ENSG00000174130 | TLR6 | 11.185378 | 11.855369 | 0.6699909 | 2.31E-06 | 3.71E-05 |
| ENSG00000258315 | C17orf49 | 7.3774141 | 8.0455854 | 0.6681713 | 0.0003798 | 0.0032516 |
| ENSG00000109436 | TBC1D9 | 12.112301 | 12.780412 | 0.6681105 | 1.69E-07 | 3.56E-06 |
| ENSG00000111348 | ARHGDIB | 13.185186 | 13.853203 | 0.6680172 | 7.08E-08 | 1.62E-06 |
| ENSG00000164167 | LSM6 | 7.4470376 | 8.1138418 | 0.6668043 | 2.16E-05 | 0.0002702 |
| ENSG00000168610 | STAT3 | 12.683941 | 13.350535 | 0.6665947 | 1.44E-15 | 1.22E-13 |
| ENSG00000156273 | BACH1 | 12.35339 | 13.019398 | 0.6660081 | 1.62E-09 | 5.03E-08 |
| ENSG00000163512 | AZI2 | 9.8030647 | 10.468949 | 0.6658842 | 6.44E-08 | 1.49E-06 |
| ENSG00000196961 | AP2A1 | 11.492302 | 12.157639 | 0.6653372 | 2.63E-10 | 9.46E-09 |
| ENSG00000130775 | THEMIS2 | 12.002209 | 12.666396 | 0.6641868 | 3.00E-06 | 4.69E-05 |
| ENSG00000128581 | IFT22 | 5.8646198 | 6.5284112 | 0.6637914 | 0.0059608 | 0.0328359 |
| ENSG00000144746 | ARL6IP5 | 12.776335 | 13.439781 | 0.6634458 | 2.24E-11 | 9.63E-10 |
| ENSG00000114346 | ECT2 | 9.179661 | 9.8429832 | 0.6633222 | 6.67E-07 | 1.22E-05 |
| ENSG00000139629 | GALNT6 | 10.298644 | 10.959533 | 0.6608894 | 5.10E-06 | 7.50E-05 |
| ENSG00000145936 | KCNMB1 | 7.8831538 | 8.5438209 | 0.660667 | 0.0012596 | 0.009002 |
| ENSG00000116977 | LGALS8 | 12.057078 | 12.717695 | 0.6606173 | 8.20E-08 | 1.85E-06 |
| ENSG00000101337 | TM9SF4 | 10.896472 | 11.555793 | 0.6593205 | 2.24E-08 | 5.65E-07 |
| ENSG00000157216 | SSBP3 | 10.161769 | 10.819338 | 0.65757 | 1.29E-05 | 0.0001711 |
| ENSG00000183570 | PCBP3 | 5.2354514 | 5.8914659 | 0.6560145 | 0.007602 | 0.0400804 |
| ENSG00000178700 | DHFR2 | 8.7750816 | 9.4300857 | 0.6550041 | 0.0002291 | 0.002114 |
| ENSG00000111676 | ATN1 | 10.323719 | 10.978542 | 0.6548231 | 0.0001138 | 0.0011556 |
| ENSG00000179361 | ARID3B | 8.9754107 | 9.6289184 | 0.6535078 | 5.81E-06 | 8.46E-05 |
| ENSG00000205413 | SAMD9 | 10.847571 | 11.500959 | 0.6533886 | 3.46E-07 | 6.80E-06 |
| ENSG00000124788 | ATXN1 | 12.408967 | 13.061773 | 0.6528063 | 1.24E-07 | 2.69E-06 |
| ENSG00000139132 | FGD4 | 11.385502 | 12.037413 | 0.6519117 | 1.29E-05 | 0.000171 |
| ENSG00000136193 | SCRN1 | 7.9240089 | 8.5751624 | 0.6511535 | 0.0013779 | 0.0097003 |
| ENSG00000178694 | NSUN3 | 9.6607618 | 10.311832 | 0.6510699 | 1.33E-10 | 4.99E-09 |
| ENSG00000156671 | SAMD8 | 10.795251 | 11.44513 | 0.649879 | 4.40E-05 | 0.0005059 |
| ENSG00000217555 | CKLF | 7.8474603 | 8.4971318 | 0.6496715 | 6.73E-06 | 9.64E-05 |
| ENSG00000172738 | TMEM217 | 5.5818454 | 6.2289867 | 0.6471413 | 0.0012773 | 0.0091123 |
| ENSG00000100354 | TNRC6B | 12.734971 | 13.38207 | 0.6470993 | 0.0027078 | 0.0170525 |
| ENSG00000149084 | HSD17B12 | 11.180617 | 11.827105 | 0.6464886 | 0.0007788 | 0.0059835 |
| ENSG00000131788 | PIAS3 | 9.07074 | 9.716846 | 0.646106 | 4.07E-05 | 0.000473 |
| ENSG00000184007 | PTP4A2 | 12.256085 | 12.901345 | 0.645261 | 9.00E-11 | 3.46E-09 |
| ENSG00000144579 | CTDSP1 | 10.47049 | 11.115441 | 0.6449517 | 0.0093294 | 0.0473239 |
| ENSG00000116815 | CD58 | 9.7658473 | 10.410529 | 0.6446814 | 0.0015212 | 0.0105609 |
| ENSG00000126804 | ZBTB1 | 10.995192 | 11.639451 | 0.6442597 | 4.52E-05 | 0.0005176 |
| ENSG00000137343 | ATAT1 | 7.2545576 | 7.8978344 | 0.6432768 | 0.0021509 | 0.0140479 |
| ENSG00000156515 | HK1 | 12.372101 | 13.014948 | 0.6428467 | 0.0002666 | 0.0024059 |
| ENSG00000196924 | FLNA | 15.283983 | 15.926124 | 0.6421402 | 0.0050195 | 0.0285635 |
| ENSG00000171860 | C3AR1 | 12.725979 | 13.366905 | 0.6409266 | 3.49E-07 | 6.83E-06 |
| ENSG00000168894 | RNF181 | 9.5242179 | 10.164783 | 0.6405646 | 8.53E-11 | 3.29E-09 |
| ENSG00000101384 | JAG1 | 9.5593554 | 10.199873 | 0.6405172 | 0.0005026 | 0.0041249 |
| ENSG00000126003 | PLAGL2 | 10.551043 | 11.191303 | 0.6402596 | 0.001585 | 0.0109227 |
| ENSG00000177565 | TBL1XR1 | 12.189324 | 12.828972 | 0.6396481 | 6.63E-07 | 1.22E-05 |
| ENSG00000144959 | NCEH1 | 12.422054 | 13.06128 | 0.6392262 | 3.44E-06 | 5.28E-05 |
| ENSG00000168615 | ADAM9 | 13.726225 | 14.365138 | 0.6389131 | 8.29E-17 | 8.47E-15 |
| ENSG00000197238 | HIST1H4J | 9.7673801 | 10.406283 | 0.6389032 | 0.0058242 | 0.0321887 |
| ENSG00000101342 | TLDC2 | 7.616945 | 8.2557517 | 0.6388067 | 0.0067355 | 0.0363169 |
| ENSG00000197872 | FAM49A | 11.232935 | 11.870977 | 0.6380422 | 3.44E-05 | 0.000407 |
| ENSG00000109466 | KLHL2 | 9.9248864 | 10.562165 | 0.6372782 | 2.21E-09 | 6.72E-08 |
| ENSG00000133678 | TMEM254 | 8.0455757 | 8.6823283 | 0.6367526 | 0.002376 | 0.015272 |
| ENSG00000005059 | MCUB | 9.1121451 | 9.7488577 | 0.6367126 | 7.10E-08 | 1.62E-06 |
| ENSG00000133657 | ATP13A3 | 12.669843 | 13.30524 | 0.6353973 | 0.002982 | 0.0184804 |
| ENSG00000164327 | RICTOR | 11.238216 | 11.873439 | 0.6352229 | 6.21E-07 | 1.15E-05 |
| ENSG00000158019 | BABAM2 | 9.9957627 | 10.630891 | 0.6351285 | 4.83E-19 | 6.57E-17 |
| ENSG00000134996 | OSTF1 | 11.175536 | 11.810351 | 0.6348146 | 4.29E-06 | 6.44E-05 |
| ENSG00000163932 | PRKCD | 12.366561 | 13.001288 | 0.634727 | 2.24E-11 | 9.63E-10 |
| ENSG00000213639 | PPP1CB | 12.411905 | 13.045957 | 0.6340511 | 4.83E-07 | 9.15E-06 |
| ENSG00000172380 | GNG12 | 8.5969575 | 9.2307123 | 0.6337548 | 0.0015309 | 0.0106118 |
| ENSG00000086065 | CHMP5 | 10.331536 | 10.965164 | 0.6336282 | 2.25E-07 | 4.61E-06 |
| ENSG00000105866 | SP4 | 8.2757928 | 8.909391 | 0.6335983 | 5.56E-12 | 2.66E-10 |
| ENSG00000067182 | TNFRSF1A | 11.537361 | 12.17071 | 0.6333487 | 1.57E-07 | 3.32E-06 |
| ENSG00000174405 | LIG4 | 9.6180041 | 10.250754 | 0.6327495 | 0.0053039 | 0.0298102 |
| ENSG00000172893 | DHCR7 | 7.9316284 | 8.5643064 | 0.632678 | 0.0018418 | 0.0123763 |
| ENSG00000177426 | TGIF1 | 8.9689359 | 9.6014479 | 0.632512 | 0.0007551 | 0.0058306 |
| ENSG00000104765 | BNIP3L | 12.726043 | 13.358345 | 0.6323024 | 1.11E-05 | 0.0001502 |
| ENSG00000164125 | FAM198B | 12.710829 | 13.3424 | 0.6315707 | 6.05E-10 | 2.04E-08 |
| ENSG00000153989 | NUS1 | 11.190107 | 11.821393 | 0.6312862 | 0.0005353 | 0.0043417 |
| ENSG00000163412 | EIF4E3 | 10.264521 | 10.895759 | 0.6312373 | 0.0064877 | 0.0352286 |
| ENSG00000108061 | SHOC2 | 10.85042 | 11.481605 | 0.6311848 | 8.14E-05 | 0.0008647 |
| ENSG00000167996 | FTH1 | 17.455909 | 18.087006 | 0.6310973 | 0.0002343 | 0.0021534 |
| ENSG00000174695 | TMEM167A | 10.624985 | 11.254926 | 0.6299408 | 1.02E-06 | 1.79E-05 |
| ENSG00000198785 | GRIN3A | 8.196698 | 8.8266319 | 0.6299339 | 0.0002573 | 0.0023314 |
| ENSG00000146094 | DOK3 | 12.033045 | 12.661594 | 0.6285493 | 8.53E-09 | 2.33E-07 |
| ENSG00000119685 | TTLL5 | 10.002765 | 10.631048 | 0.6282829 | 3.53E-06 | 5.40E-05 |
| ENSG00000003987 | MTMR7 | 8.5891205 | 9.2172319 | 0.6281114 | 0.0006072 | 0.004836 |
| ENSG00000183077 | AFMID | 7.7069396 | 8.3350335 | 0.6280939 | 0.0034249 | 0.0208042 |
| ENSG00000197062 | ZSCAN26 | 9.0165664 | 9.6445469 | 0.6279806 | 2.89E-06 | 4.53E-05 |
| ENSG00000204681 | GABBR1 | 9.4838487 | 10.111192 | 0.6273434 | 0.0040961 | 0.0242073 |
| ENSG00000198142 | SOWAHC | 8.8260137 | 9.453173 | 0.6271593 | 0.0015818 | 0.010902 |
| ENSG00000074935 | TUBE1 | 6.6683299 | 7.2954723 | 0.6271424 | 0.0003702 | 0.0031835 |
| ENSG00000130821 | SLC6A8 | 8.3223389 | 8.9480797 | 0.6257408 | 5.23E-07 | 9.82E-06 |
| ENSG00000165959 | CLMN | 12.275427 | 12.900901 | 0.625474 | 1.35E-05 | 0.0001779 |
| ENSG00000271303 | SRXN1 | 10.303101 | 10.927713 | 0.6246121 | 0.0042575 | 0.0249919 |
| ENSG00000144597 | EAF1 | 10.984391 | 11.608528 | 0.624137 | 1.79E-05 | 0.0002282 |
| ENSG00000137312 | FLOT1 | 11.401803 | 12.024611 | 0.6228078 | 9.74E-07 | 1.72E-05 |
| ENSG00000203705 | TATDN3 | 8.1768696 | 8.7996402 | 0.6227706 | 0.0006057 | 0.0048281 |
| ENSG00000167306 | MYO5B | 6.1964303 | 6.8190557 | 0.6226255 | 0.0011635 | 0.0084195 |
| ENSG00000159346 | ADIPOR1 | 12.129598 | 12.752006 | 0.6224072 | 0.0001223 | 0.0012309 |
| ENSG00000145012 | LPP | 12.580494 | 13.202286 | 0.6217917 | 0.001261 | 0.0090072 |
| ENSG00000156735 | BAG4 | 10.270135 | 10.89125 | 0.6211151 | 5.08E-08 | 1.20E-06 |
| ENSG00000222046 | DCDC2B | 4.9555734 | 5.5765801 | 0.6210067 | 0.0065544 | 0.0355199 |
| ENSG00000160685 | ZBTB7B | 9.8789344 | 10.499873 | 0.6209383 | 0.0018304 | 0.0123131 |
| ENSG00000182952 | HMGN4 | 9.4642591 | 10.084884 | 0.6206252 | 4.53E-10 | 1.56E-08 |
| ENSG00000049239 | H6PD | 11.621126 | 12.241697 | 0.6205705 | 0.0018679 | 0.0125193 |
| ENSG00000104671 | DCTN6 | 8.646457 | 9.2667301 | 0.6202732 | 9.94E-05 | 0.0010259 |
| ENSG00000144843 | ADPRH | 9.2548097 | 9.8746965 | 0.6198869 | 0.0003739 | 0.003211 |
| ENSG00000198265 | HELZ | 12.838402 | 13.458256 | 0.619854 | 1.06E-06 | 1.85E-05 |
| ENSG00000182511 | FES | 9.8839826 | 10.503567 | 0.6195842 | 0.0022882 | 0.0147795 |
| ENSG00000079805 | DNM2 | 12.697617 | 13.317101 | 0.6194838 | 1.45E-15 | 1.22E-13 |
| ENSG00000099337 | KCNK6 | 10.189038 | 10.806743 | 0.6177048 | 4.11E-05 | 0.0004764 |
| ENSG00000127526 | SLC35E1 | 9.9846806 | 10.601941 | 0.61726 | 0.0008821 | 0.0066551 |
| ENSG00000162928 | PEX13 | 9.5867137 | 10.203706 | 0.6169923 | 7.26E-09 | 2.01E-07 |
| ENSG00000057657 | PRDM1 | 10.601981 | 11.218836 | 0.616855 | 0.0044067 | 0.0257015 |
| ENSG00000176463 | SLCO3A1 | 10.147904 | 10.76465 | 0.6167454 | 7.99E-09 | 2.19E-07 |
| ENSG00000082258 | CCNT2 | 10.380371 | 10.996744 | 0.616373 | 1.09E-05 | 0.0001476 |
| ENSG00000084093 | REST | 11.369875 | 11.985256 | 0.6153806 | 0.0002634 | 0.0023798 |
| ENSG00000172954 | LCLAT1 | 8.0272051 | 8.6419997 | 0.6147946 | 0.00697 | 0.037397 |
| ENSG00000127314 | RAP1B | 12.209239 | 12.823273 | 0.6140334 | 1.87E-06 | 3.10E-05 |
| ENSG00000165704 | HPRT1 | 8.4953707 | 9.1066177 | 0.611247 | 6.85E-06 | 9.79E-05 |
| ENSG00000130270 | ATP8B3 | 6.6072325 | 7.2180335 | 0.610801 | 0.0018913 | 0.0126548 |
| ENSG00000110906 | KCTD10 | 10.395221 | 11.004785 | 0.6095637 | 3.32E-06 | 5.12E-05 |
| ENSG00000075089 | ACTR6 | 7.8056224 | 8.4143814 | 0.608759 | 2.05E-06 | 3.35E-05 |
| ENSG00000182985 | CADM1 | 10.915132 | 11.523659 | 0.6085263 | 0.0035314 | 0.0213329 |
| ENSG00000163872 | YEATS2 | 10.989718 | 11.598023 | 0.6083047 | 8.77E-06 | 0.0001223 |
| ENSG00000133704 | IPO8 | 11.366213 | 11.974454 | 0.6082415 | 8.02E-11 | 3.12E-09 |
| ENSG00000082213 | C5orf22 | 9.5667031 | 10.174914 | 0.6082108 | 7.58E-13 | 4.14E-11 |
| ENSG00000133059 | DSTYK | 10.702227 | 11.310352 | 0.6081251 | 1.03E-22 | 2.10E-20 |
| ENSG00000074800 | ENO1 | 13.789804 | 14.396866 | 0.607062 | 2.70E-05 | 0.0003281 |
| ENSG00000110315 | RNF141 | 10.833842 | 11.440525 | 0.6066835 | 0.0001704 | 0.0016383 |
| ENSG00000163946 | FAM208A | 11.971822 | 12.57793 | 0.6061083 | 8.01E-06 | 0.0001129 |
| ENSG00000101347 | SAMHD1 | 13.428639 | 14.032782 | 0.6041425 | 5.15E-11 | 2.09E-09 |
| ENSG00000104331 | IMPAD1 | 11.171515 | 11.775474 | 0.6039593 | 3.51E-06 | 5.38E-05 |
| ENSG00000077721 | UBE2A | 10.090278 | 10.693777 | 0.6034992 | 4.39E-06 | 6.56E-05 |
| ENSG00000154175 | ABI3BP | 5.4391479 | 6.0423836 | 0.6032357 | 0.0052915 | 0.029763 |
| ENSG00000026751 | SLAMF7 | 12.001286 | 12.603395 | 0.6021091 | 0.002295 | 0.0148168 |
| ENSG00000167987 | VPS37C | 10.301832 | 10.90326 | 0.6014282 | 0.0002044 | 0.0019194 |
| ENSG00000120519 | SLC10A7 | 8.8519279 | 9.4526713 | 0.6007434 | 4.90E-05 | 0.0005572 |
| ENSG00000075151 | EIF4G3 | 12.239094 | 12.839461 | 0.6003671 | 1.46E-06 | 2.48E-05 |
| ENSG00000114013 | CD86 | 11.920668 | 12.52091 | 0.600242 | 1.92E-05 | 0.000243 |
| ENSG00000016864 | GLT8D1 | 8.7418975 | 9.3410854 | 0.5991879 | 1.71E-10 | 6.30E-09 |
| ENSG00000087157 | PGS1 | 10.234403 | 10.833069 | 0.5986655 | 1.79E-05 | 0.0002289 |
| ENSG00000089159 | PXN | 11.950692 | 12.547616 | 0.5969236 | 0.0013237 | 0.009385 |
| ENSG00000197299 | BLM | 6.7125714 | 7.3094049 | 0.5968335 | 0.0029632 | 0.0183852 |
| ENSG00000072062 | PRKACA | 11.360573 | 11.957176 | 0.5966031 | 9.90E-07 | 1.74E-05 |
| ENSG00000198938 | MT-CO3 | 14.387081 | 14.983477 | 0.5963962 | 0.0035555 | 0.0214603 |
| ENSG00000155096 | AZIN1 | 11.699145 | 12.295427 | 0.5962822 | 1.67E-05 | 0.000214 |
| ENSG00000133138 | TBC1D8B | 7.3913843 | 7.986726 | 0.5953417 | 5.94E-05 | 0.0006564 |
| ENSG00000102218 | RP2 | 10.332724 | 10.927708 | 0.5949843 | 1.13E-07 | 2.48E-06 |
| ENSG00000155975 | VPS37A | 9.7679693 | 10.362568 | 0.5945989 | 0.0007164 | 0.00558 |
| ENSG00000109332 | UBE2D3 | 12.163062 | 12.757566 | 0.5945036 | 3.32E-11 | 1.39E-09 |
| ENSG00000182504 | CEP97 | 7.8650785 | 8.4589336 | 0.5938551 | 0.0015254 | 0.0105845 |
| ENSG00000155366 | RHOC | 9.3581883 | 9.9512589 | 0.5930705 | 0.0066492 | 0.0359314 |
| ENSG00000148180 | GSN | 13.361563 | 13.954444 | 0.5928815 | 0.0054022 | 0.0302729 |
| ENSG00000138386 | NAB1 | 9.7602072 | 10.352175 | 0.591968 | 3.26E-06 | 5.04E-05 |
| ENSG00000112419 | PHACTR2 | 11.149446 | 11.741369 | 0.5919231 | 7.90E-07 | 1.43E-05 |
| ENSG00000151693 | ASAP2 | 7.1548957 | 7.7462305 | 0.5913348 | 0.0044717 | 0.0259902 |
| ENSG00000112335 | SNX3 | 11.697219 | 12.287891 | 0.5906716 | 9.16E-06 | 0.0001269 |
| ENSG00000134830 | C5AR2 | 8.8179358 | 9.4080679 | 0.5901321 | 0.0011699 | 0.0084626 |
| ENSG00000116701 | NCF2 | 13.746505 | 14.336385 | 0.5898797 | 0.0005185 | 0.0042248 |
| ENSG00000144560 | VGLL4 | 10.681233 | 11.270704 | 0.589471 | 3.32E-06 | 5.13E-05 |
| ENSG00000110876 | SELPLG | 11.002603 | 11.592067 | 0.5894648 | 0.008771 | 0.0450024 |
| ENSG00000180008 | SOCS4 | 9.7177332 | 10.306914 | 0.5891805 | 7.41E-05 | 0.0007965 |
| ENSG00000178467 | P4HTM | 8.5071926 | 9.0962596 | 0.589067 | 4.54E-06 | 6.77E-05 |
| ENSG00000167535 | CACNB3 | 6.1872799 | 6.7758034 | 0.5885235 | 0.0067362 | 0.0363169 |
| ENSG00000128708 | HAT1 | 9.3742468 | 9.9627595 | 0.5885127 | 1.25E-06 | 2.15E-05 |
| ENSG00000110852 | CLEC2B | 10.170096 | 10.758454 | 0.5883587 | 0.0002475 | 0.002257 |
| ENSG00000114439 | BBX | 10.663459 | 11.251561 | 0.5881022 | 0.0028462 | 0.0177748 |
| ENSG00000111647 | UHRF1BP1L | 11.49589 | 12.082822 | 0.5869317 | 1.26E-09 | 4.02E-08 |
| ENSG00000166971 | AKTIP | 7.9322433 | 8.5190373 | 0.586794 | 7.19E-05 | 0.0007753 |
| ENSG00000091317 | CMTM6 | 12.109134 | 12.695887 | 0.586753 | 2.19E-05 | 0.0002733 |
| ENSG00000139266 | MARCHF9 | 9.2316896 | 9.8183979 | 0.5867084 | 2.43E-05 | 0.0002995 |
| ENSG00000096060 | FKBP5 | 11.40138 | 11.987479 | 0.5860984 | 0.0004438 | 0.0037105 |
| ENSG00000134802 | SLC43A3 | 11.576376 | 12.162297 | 0.5859215 | 6.50E-06 | 9.32E-05 |
| ENSG00000162664 | ZNF326 | 9.1159876 | 9.7016545 | 0.5856669 | 0.0004837 | 0.0039924 |
| ENSG00000070371 | CLTCL1 | 7.2703722 | 7.8558161 | 0.585444 | 0.0009357 | 0.0070062 |
| ENSG00000112640 | PPP2R5D | 9.5958686 | 10.180931 | 0.5850623 | 3.17E-07 | 6.32E-06 |
| ENSG00000119636 | BBOF1 | 6.6667326 | 7.2517381 | 0.5850055 | 0.0057513 | 0.0318581 |
| ENSG00000172350 | ABCG4 | 8.4181991 | 2.1863055 | -6.231894 | 5.09E-13 | 2.85E-11 |
| ENSG00000255819 | KLRC4-KLRK1 | 6.6668907 | 1.0386668 | -5.628224 | 0.0005476 | 0.004428 |
| ENSG00000003096 | KLHL13 | 8.6336088 | 3.0213344 | -5.612274 | 2.75E-08 | 6.87E-07 |
| ENSG00000162692 | VCAM1 | 6.248865 | 1.2934741 | -4.955391 | 1.81E-11 | 7.88E-10 |
| ENSG00000174500 | GCSAM | 6.0979241 | 1.1501993 | -4.947725 | 3.97E-27 | 1.26E-24 |
| ENSG00000148488 | ST8SIA6 | 4.8861829 | 0.1963638 | -4.689819 | 1.25E-16 | 1.23E-14 |
| ENSG00000213809 | KLRK1 | 7.0005252 | 2.313736 | -4.686789 | 0.0002689 | 0.0024225 |
| ENSG00000122254 | HS3ST2 | 14.35432 | 9.7723587 | -4.581961 | 1.61E-05 | 0.0002071 |
| ENSG00000017427 | IGF1 | 12.104974 | 7.5923655 | -4.512609 | 2.32E-05 | 0.0002884 |
| ENSG00000134160 | TRPM1 | 4.482021 | 0.3701578 | -4.111863 | 7.01E-08 | 1.61E-06 |
| ENSG00000136943 | CTSV | 7.0215798 | 2.9244433 | -4.097136 | 5.20E-14 | 3.44E-12 |
| ENSG00000109819 | PPARGC1A | 4.9359101 | 0.878686 | -4.057224 | 8.56E-07 | 1.53E-05 |
| ENSG00000120457 | KCNJ5 | 14.018836 | 10.000041 | -4.018795 | 1.57E-16 | 1.54E-14 |
| ENSG00000117425 | PTCH2 | 9.2325408 | 5.2320772 | -4.000464 | 6.15E-38 | 5.61E-35 |
| ENSG00000163687 | DNASE1L3 | 4.4134172 | 0.4766066 | -3.936811 | 2.26E-13 | 1.35E-11 |
| ENSG00000149927 | DOC2A | 7.0095847 | 3.1293623 | -3.880222 | 8.84E-11 | 3.40E-09 |
| ENSG00000159713 | TPPP3 | 4.0910139 | 0.2827647 | -3.808249 | 1.18E-12 | 6.24E-11 |
| ENSG00000160460 | SPTBN4 | 7.6257097 | 3.8271594 | -3.79855 | 5.34E-11 | 2.15E-09 |
| ENSG00000144218 | AFF3 | 5.6275102 | 1.8439524 | -3.783558 | 8.55E-07 | 1.53E-05 |
| ENSG00000122224 | LY9 | 9.077395 | 5.3091761 | -3.768219 | 8.09E-05 | 0.0008597 |
| ENSG00000145708 | CRHBP | 7.8662584 | 4.1163824 | -3.749876 | 2.38E-13 | 1.42E-11 |
| ENSG00000040608 | RTN4R | 8.1677664 | 4.4311642 | -3.736602 | 1.79E-06 | 2.98E-05 |
| ENSG00000141696 | P3H4 | 5.2032429 | 1.4695899 | -3.733653 | 1.59E-05 | 0.0002053 |
| ENSG00000053108 | FSTL4 | 4.8660506 | 1.1352278 | -3.730823 | 4.25E-14 | 2.86E-12 |
| ENSG00000101197 | BIRC7 | 6.4050729 | 2.6901374 | -3.714935 | 1.89E-13 | 1.14E-11 |
| ENSG00000150551 | LYPD1 | 4.8448161 | 1.2154231 | -3.629393 | 0.0001393 | 0.0013765 |
| ENSG00000082438 | COBLL1 | 9.6066639 | 5.9986646 | -3.607999 | 1.98E-27 | 6.52E-25 |
| ENSG00000113645 | WWC1 | 5.0810075 | 1.5196182 | -3.561389 | 2.60E-07 | 5.27E-06 |
| ENSG00000138449 | SLC40A1 | 12.079255 | 8.5212147 | -3.55804 | 9.76E-33 | 6.02E-30 |
| ENSG00000069702 | TGFBR3 | 6.8663583 | 3.3418659 | -3.524492 | 1.55E-16 | 1.52E-14 |
| ENSG00000239961 | LILRA4 | 6.9891163 | 3.470409 | -3.518707 | 0.0043558 | 0.0254587 |
| ENSG00000183549 | ACSM5 | 8.7510318 | 5.2704445 | -3.480587 | 5.01E-21 | 8.34E-19 |
| ENSG00000106483 | SFRP4 | 6.3045131 | 2.8343648 | -3.470148 | 4.44E-13 | 2.52E-11 |
| ENSG00000172967 | XKR3 | 5.5997198 | 2.1325193 | -3.4672 | 1.69E-06 | 2.84E-05 |
| ENSG00000164047 | CAMP | 5.0811166 | 1.6682279 | -3.412889 | 3.48E-08 | 8.48E-07 |
| ENSG00000148219 | ASTN2 | 6.8680211 | 3.4561643 | -3.411857 | 1.12E-11 | 5.11E-10 |
| ENSG00000143850 | PLEKHA6 | 6.2364339 | 2.8282382 | -3.408196 | 2.42E-08 | 6.09E-07 |
| ENSG00000185668 | POU3F1 | 4.3294643 | 0.9266629 | -3.402801 | 0.0001031 | 0.0010589 |
| ENSG00000162849 | KIF26B | 6.7349655 | 3.3784622 | -3.356503 | 5.37E-15 | 4.13E-13 |
| ENSG00000183542 | KLRC4 | 4.5137255 | 1.1617254 | -3.352 | 0.0018785 | 0.0125803 |
| ENSG00000183833 | MAATS1 | 5.7196392 | 2.4122082 | -3.307431 | 9.23E-15 | 6.90E-13 |
| ENSG00000166897 | ELFN2 | 3.6878172 | 0.411444 | -3.276373 | 0.0005147 | 0.004204 |
| ENSG00000112294 | ALDH5A1 | 8.1157679 | 4.871515 | -3.244253 | 1.16E-19 | 1.69E-17 |
| ENSG00000185742 | C11orf87 | 6.6316776 | 3.4106155 | -3.221062 | 7.82E-11 | 3.04E-09 |
| ENSG00000168672 | FAM84B | 5.7557821 | 2.5473343 | -3.208448 | 1.51E-14 | 1.09E-12 |
| ENSG00000167191 | GPRC5B | 10.343053 | 7.1468016 | -3.196251 | 2.32E-05 | 0.0002878 |
| ENSG00000173930 | SLCO4C1 | 8.2130419 | 5.0271817 | -3.18586 | 5.68E-10 | 1.93E-08 |
| ENSG00000179097 | HTR1F | 3.8899263 | 0.72038 | -3.169546 | 2.26E-07 | 4.62E-06 |
| ENSG00000135253 | KCP | 10.579173 | 7.460849 | -3.118324 | 1.76E-05 | 0.0002246 |
| ENSG00000163171 | CDC42EP3 | 11.853674 | 8.7378185 | -3.115856 | 2.03E-31 | 1.07E-28 |
| ENSG00000166949 | SMAD3 | 8.4997266 | 5.3888399 | -3.110887 | 6.42E-30 | 2.90E-27 |
| ENSG00000226321 | CROCC2 | 4.2752637 | 1.1660154 | -3.109248 | 9.36E-13 | 5.04E-11 |
| ENSG00000103056 | SMPD3 | 8.0261581 | 4.9191752 | -3.106983 | 1.59E-32 | 9.55E-30 |
| ENSG00000161381 | PLXDC1 | 5.330837 | 2.2278672 | -3.10297 | 7.46E-06 | 0.0001061 |
| ENSG00000113494 | PRLR | 9.3552722 | 6.2808394 | -3.074433 | 1.04E-06 | 1.82E-05 |
| ENSG00000138435 | CHRNA1 | 3.3188214 | 0.2693194 | -3.049502 | 1.15E-06 | 1.99E-05 |
| ENSG00000163121 | NEURL3 | 4.1841237 | 1.160898 | -3.023226 | 4.33E-06 | 6.50E-05 |
| ENSG00000120471 | TP53AIP1 | 4.0388451 | 1.0294447 | -3.0094 | 7.06E-06 | 0.0001008 |
| ENSG00000182162 | P2RY8 | 12.022607 | 9.0239858 | -2.998621 | 2.70E-07 | 5.46E-06 |
| ENSG00000114019 | AMOTL2 | 4.0049618 | 1.0191236 | -2.985838 | 3.83E-07 | 7.43E-06 |
| ENSG00000144485 | HES6 | 5.2599758 | 2.2752854 | -2.98469 | 1.35E-12 | 7.05E-11 |
| ENSG00000155265 | GOLGA7B | 6.5978046 | 3.6342233 | -2.963581 | 0.0021921 | 0.014266 |
| ENSG00000137726 | FXYD6 | 10.495211 | 7.5344145 | -2.960796 | 1.61E-18 | 2.02E-16 |
| ENSG00000111816 | FRK | 8.4287783 | 5.4966787 | -2.9321 | 4.27E-11 | 1.76E-09 |
| ENSG00000116329 | OPRD1 | 5.0424122 | 2.1128935 | -2.929519 | 4.31E-09 | 1.24E-07 |
| ENSG00000176490 | DIRAS1 | 7.7000749 | 4.786415 | -2.91366 | 1.07E-05 | 0.0001454 |
| ENSG00000266964 | FXYD1 | 3.6804663 | 0.7818487 | -2.898618 | 6.64E-08 | 1.53E-06 |
| ENSG00000175262 | C1orf127 | 9.3916333 | 6.4962511 | -2.895382 | 3.15E-16 | 2.94E-14 |
| ENSG00000105366 | SIGLEC8 | 3.7295466 | 0.8387095 | -2.890837 | 0.0004942 | 0.0040632 |
| ENSG00000115306 | SPTBN1 | 11.095179 | 8.2093916 | -2.885787 | 1.36E-24 | 3.40E-22 |
| ENSG00000159685 | CHCHD6 | 7.7152786 | 4.8310736 | -2.884205 | 1.72E-17 | 1.89E-15 |
| ENSG00000172572 | PDE3A | 6.0579471 | 3.1880264 | -2.869921 | 1.87E-09 | 5.74E-08 |
| ENSG00000185614 | FAM212A | 5.7534716 | 2.890293 | -2.863179 | 6.92E-17 | 7.14E-15 |
| ENSG00000176533 | GNG7 | 9.1551894 | 6.3014701 | -2.853719 | 2.62E-12 | 1.31E-10 |
| ENSG00000182752 | PAPPA | 4.1186853 | 1.2750048 | -2.843681 | 0.0045202 | 0.0262101 |
| ENSG00000176485 | PLA2G16 | 6.7488284 | 3.9152521 | -2.833576 | 2.73E-11 | 1.16E-09 |
| ENSG00000006747 | SCIN | 8.3147107 | 5.4861459 | -2.828565 | 7.44E-34 | 5.25E-31 |
| ENSG00000235961 | PNMA6A | 6.1292076 | 3.3185261 | -2.810681 | 7.62E-13 | 4.15E-11 |
| ENSG00000151376 | ME3 | 8.3653393 | 5.5672621 | -2.798077 | 1.87E-22 | 3.68E-20 |
| ENSG00000116396 | KCNC4 | 10.353485 | 7.576556 | -2.776929 | 2.00E-06 | 3.27E-05 |
| ENSG00000204805 | FAM27E4 | 4.48515 | 1.7360166 | -2.749133 | 0.0018009 | 0.0121351 |
| ENSG00000133315 | MACROD1 | 5.7224985 | 2.9770927 | -2.745406 | 4.51E-10 | 1.56E-08 |
| ENSG00000061918 | GUCY1B3 | 4.1988027 | 1.4547817 | -2.744021 | 9.64E-05 | 0.0009994 |
| ENSG00000019991 | HGF | 12.437369 | 9.7475701 | -2.689799 | 2.42E-17 | 2.63E-15 |
| ENSG00000155719 | OTOA | 8.3830764 | 5.7012994 | -2.681777 | 4.46E-13 | 2.53E-11 |
| ENSG00000158806 | NPM2 | 3.4952952 | 0.8137448 | -2.68155 | 0.0001288 | 0.0012883 |
| ENSG00000100095 | SEZ6L | 4.9913649 | 2.3119889 | -2.679376 | 0.0003893 | 0.003319 |
| ENSG00000011028 | MRC2 | 13.82552 | 11.14764 | -2.677879 | 1.53E-33 | 1.03E-30 |
| ENSG00000124406 | ATP8A1 | 12.042644 | 9.3704995 | -2.672144 | 4.64E-34 | 3.33E-31 |
| ENSG00000168952 | STXBP6 | 2.8226587 | 0.1552686 | -2.66739 | 3.47E-05 | 0.0004097 |
| ENSG00000114529 | C3orf52 | 3.5631369 | 0.8964666 | -2.66667 | 4.46E-09 | 1.28E-07 |
| ENSG00000163485 | ADORA1 | 5.0499446 | 2.3861294 | -2.663815 | 7.77E-05 | 0.0008301 |
| ENSG00000101955 | SRPX | 7.4085011 | 4.7558056 | -2.652696 | 0.0012235 | 0.0087839 |
| ENSG00000154928 | EPHB1 | 6.5744007 | 3.9232663 | -2.651134 | 1.67E-09 | 5.19E-08 |
| ENSG00000141384 | TAF4B | 7.7774437 | 5.1478239 | -2.62962 | 0.0002914 | 0.0025983 |
| ENSG00000167775 | CD320 | 6.3408817 | 3.7201739 | -2.620708 | 5.25E-11 | 2.12E-09 |
| ENSG00000073737 | DHRS9 | 10.395268 | 7.7981703 | -2.597097 | 3.69E-13 | 2.13E-11 |
| ENSG00000166840 | GLYATL1 | 5.3915924 | 2.8019503 | -2.589642 | 1.42E-09 | 4.50E-08 |
| ENSG00000083067 | TRPM3 | 4.4411002 | 1.8583062 | -2.582794 | 0.0007119 | 0.0055529 |
| ENSG00000117632 | STMN1 | 9.3542112 | 6.7794784 | -2.574733 | 1.70E-27 | 5.73E-25 |
| ENSG00000205436 | EXOC3L4 | 3.1481909 | 0.5752684 | -2.572923 | 0.0021711 | 0.0141586 |
| ENSG00000236980 | C3orf84 | 5.0510813 | 2.4869661 | -2.564115 | 4.33E-06 | 6.49E-05 |
| ENSG00000102575 | ACP5 | 14.969738 | 12.420592 | -2.549146 | 7.58E-06 | 0.0001076 |
| ENSG00000103485 | QPRT | 10.539548 | 7.9986222 | -2.540926 | 4.23E-10 | 1.47E-08 |
| ENSG00000260691 | ANKRD20A1 | 5.9099185 | 3.3796529 | -2.530266 | 0.0005064 | 0.0041469 |
| ENSG00000136848 | DAB2IP | 5.188144 | 2.6816628 | -2.506481 | 8.13E-10 | 2.69E-08 |
| ENSG00000078098 | FAP | 3.6611529 | 1.1769431 | -2.48421 | 0.0007947 | 0.0060808 |
| ENSG00000101004 | NINL | 7.1830291 | 4.7015054 | -2.481524 | 9.75E-10 | 3.18E-08 |
| ENSG00000196208 | GREB1 | 6.8362139 | 4.3694356 | -2.466778 | 0.009755 | 0.0490959 |
| ENSG00000178038 | ALS2CL | 8.1176107 | 5.6621164 | -2.455494 | 5.67E-15 | 4.31E-13 |
| ENSG00000004846 | ABCB5 | 4.8837834 | 2.4338135 | -2.44997 | 0.0057089 | 0.0316594 |
| ENSG00000170298 | LGALS9B | 2.8684175 | 0.4276835 | -2.440734 | 4.30E-05 | 0.0004948 |
| ENSG00000171916 | LGALS9C | 3.2102571 | 0.7732266 | -2.437031 | 0.0066995 | 0.0361585 |
| ENSG00000163106 | HPGDS | 7.0618344 | 4.6299099 | -2.431925 | 1.22E-13 | 7.66E-12 |
| ENSG00000185527 | PDE6G | 6.088798 | 3.6651886 | -2.423609 | 1.52E-09 | 4.77E-08 |
| ENSG00000169224 | GCSAML | 3.4990291 | 1.0771431 | -2.421886 | 0.0047458 | 0.0272686 |
| ENSG00000181585 | TMIE | 3.0798206 | 0.6632608 | -2.41656 | 7.60E-05 | 0.0008143 |
| ENSG00000117791 | MTARC2 | 4.6057316 | 2.194853 | -2.410879 | 2.92E-06 | 4.57E-05 |
| ENSG00000203943 | SAMD13 | 5.9069141 | 3.4975544 | -2.40936 | 1.88E-09 | 5.77E-08 |
| ENSG00000196950 | SLC39A10 | 10.295368 | 7.8941627 | -2.401205 | 1.72E-33 | 1.14E-30 |
| ENSG00000128596 | CCDC136 | 5.8390176 | 3.4393026 | -2.399715 | 6.99E-09 | 1.94E-07 |
| ENSG00000198286 | CARD11 | 9.8502768 | 7.4510414 | -2.399235 | 1.57E-20 | 2.54E-18 |
| ENSG00000168389 | MFSD2A | 10.174418 | 7.7785805 | -2.395837 | 3.64E-06 | 5.55E-05 |
| ENSG00000267221 | C17orf113 | 5.8019248 | 3.4117254 | -2.390199 | 2.75E-08 | 6.86E-07 |
| ENSG00000125378 | BMP4 | 2.3898705 | 0 | -2.389871 | 1.73E-08 | 4.47E-07 |
| ENSG00000185518 | SV2B | 4.2336564 | 1.8461328 | -2.387524 | 0.0017407 | 0.0118115 |
| ENSG00000164684 | ZNF704 | 11.044356 | 8.6604465 | -2.383909 | 3.14E-07 | 6.27E-06 |
| ENSG00000205832 | C16orf96 | 6.1371191 | 3.7543243 | -2.382795 | 0.0002668 | 0.0024072 |
| ENSG00000095739 | BAMBI | 4.6158012 | 2.2346131 | -2.381188 | 0.0009555 | 0.0071288 |
| ENSG00000168490 | PHYHIP | 2.6461475 | 0.2773629 | -2.368785 | 1.86E-05 | 0.0002359 |
| ENSG00000155016 | CYP2U1 | 8.5973098 | 6.2286525 | -2.368657 | 4.53E-27 | 1.43E-24 |
| ENSG00000178015 | GPR150 | 4.9726543 | 2.6180487 | -2.354606 | 1.60E-07 | 3.39E-06 |
| ENSG00000185909 | KLHDC8B | 11.67973 | 9.3271199 | -2.352611 | 9.05E-05 | 0.0009465 |
| ENSG00000174473 | GALNTL6 | 2.5494115 | 0.2108268 | -2.338585 | 3.02E-06 | 4.71E-05 |
| ENSG00000234303 | CLYBL-AS1 | 2.756396 | 0.429436 | -2.32696 | 0.0003288 | 0.0028782 |
| ENSG00000107593 | PKD2L1 | 6.9641248 | 4.6378332 | -2.326292 | 2.13E-05 | 0.000267 |
| ENSG00000132613 | MTSS1L | 8.2146346 | 5.8952964 | -2.319338 | 7.56E-18 | 8.68E-16 |
| ENSG00000146409 | SLC18B1 | 11.657477 | 9.3415932 | -2.315884 | 1.42E-34 | 1.07E-31 |
| ENSG00000276203 | ANKRD20A3 | 5.3344375 | 3.0231883 | -2.311249 | 0.0001668 | 0.0016093 |
| ENSG00000135144 | DTX1 | 8.4049113 | 6.0959479 | -2.308963 | 6.84E-06 | 9.78E-05 |
| ENSG00000115263 | GCG | 4.0524594 | 1.7438919 | -2.308568 | 6.13E-08 | 1.42E-06 |
| ENSG00000115648 | MLPH | 8.3228059 | 6.0199399 | -2.302866 | 1.36E-11 | 6.09E-10 |
| ENSG00000156886 | ITGAD | 9.0182679 | 6.7164698 | -2.301798 | 5.95E-10 | 2.01E-08 |
| ENSG00000177103 | DSCAML1 | 2.2960683 | 0 | -2.296068 | 0.000792 | 0.0060694 |
| ENSG00000182612 | TSPAN10 | 7.1069829 | 4.8119573 | -2.295026 | 1.52E-09 | 4.77E-08 |
| ENSG00000012124 | CD22 | 10.211315 | 7.9178466 | -2.293468 | 2.39E-05 | 0.0002947 |
| ENSG00000122574 | WIPF3 | 6.538003 | 4.247951 | -2.290052 | 1.39E-05 | 0.0001821 |
| ENSG00000145147 | SLIT2 | 4.4598301 | 2.1709245 | -2.288906 | 3.91E-08 | 9.43E-07 |
| ENSG00000196664 | TLR7 | 12.700885 | 10.422081 | -2.278805 | 3.25E-10 | 1.15E-08 |
| ENSG00000162458 | FBLIM1 | 6.0423347 | 3.766507 | -2.275828 | 1.01E-08 | 2.73E-07 |
| ENSG00000132329 | RAMP1 | 3.253403 | 0.9820926 | -2.27131 | 0.0018438 | 0.0123867 |
| ENSG00000157510 | AFAP1L1 | 10.394417 | 8.136589 | -2.257828 | 1.89E-09 | 5.81E-08 |
| ENSG00000119401 | TRIM32 | 9.9196848 | 7.6656649 | -2.25402 | 1.18E-05 | 0.0001583 |
| ENSG00000111249 | CUX2 | 8.7710257 | 6.5190188 | -2.252007 | 1.26E-24 | 3.19E-22 |
| ENSG00000198794 | SCAMP5 | 6.8543139 | 4.6092559 | -2.245058 | 5.91E-06 | 8.58E-05 |
| ENSG00000175920 | DOK7 | 8.0550503 | 5.8180326 | -2.237018 | 1.57E-06 | 2.65E-05 |
| ENSG00000107282 | APBA1 | 11.795562 | 9.5631108 | -2.232451 | 4.04E-10 | 1.41E-08 |
| ENSG00000102878 | HSF4 | 9.5209732 | 7.2905568 | -2.230416 | 2.11E-13 | 1.27E-11 |
| ENSG00000134986 | NREP | 10.88997 | 8.6706215 | -2.219349 | 1.32E-24 | 3.33E-22 |
| ENSG00000188596 | CFAP54 | 2.7746057 | 0.5613121 | -2.213294 | 0.0053611 | 0.0300734 |
| ENSG00000169989 | TIGD4 | 3.0181549 | 0.8102682 | -2.207887 | 0.0040343 | 0.0238749 |
| ENSG00000120256 | LRP11 | 7.1800586 | 4.9748838 | -2.205175 | 0.0033806 | 0.0205723 |
| ENSG00000146755 | TRIM50 | 3.5790989 | 1.3805353 | -2.198564 | 0.0007926 | 0.0060699 |
| ENSG00000159871 | LYPD5 | 4.3480981 | 2.1504535 | -2.197645 | 0.002162 | 0.0141077 |
| ENSG00000120341 | SEC16B | 4.4327875 | 2.2364099 | -2.196378 | 0.0070705 | 0.0378158 |
| ENSG00000186326 | RGS9BP | 3.0919102 | 0.897741 | -2.194169 | 0.0005347 | 0.0043386 |
| ENSG00000122547 | EEPD1 | 10.525302 | 8.3362372 | -2.189064 | 3.78E-05 | 0.0004419 |
| ENSG00000145358 | DDIT4L | 6.3830515 | 4.2042761 | -2.178775 | 8.53E-05 | 0.0008997 |
| ENSG00000164116 | GUCY1A3 | 2.8536628 | 0.6796505 | -2.174012 | 0.0002973 | 0.0026418 |
| ENSG00000167601 | AXL | 7.9743185 | 5.8029249 | -2.171394 | 6.21E-11 | 2.48E-09 |
| ENSG00000020577 | SAMD4A | 12.107296 | 9.940946 | -2.16635 | 6.40E-22 | 1.17E-19 |
| ENSG00000186827 | TNFRSF4 | 6.3742182 | 4.2152289 | -2.158989 | 0.0001462 | 0.0014364 |
| ENSG00000255524 | NPIPB8 | 3.6776304 | 1.5199204 | -2.15771 | 6.24E-05 | 0.0006854 |
| ENSG00000161681 | SHANK1 | 4.6935901 | 2.5361789 | -2.157411 | 0.000606 | 0.0048282 |
| ENSG00000184305 | CCSER1 | 8.6276866 | 6.4739142 | -2.153772 | 4.06E-16 | 3.75E-14 |
| ENSG00000137976 | DNASE2B | 6.7868206 | 4.6401006 | -2.14672 | 1.24E-05 | 0.0001655 |
| ENSG00000214575 | CPEB1 | 6.5489348 | 4.4037197 | -2.145215 | 0.0041503 | 0.0244833 |
| ENSG00000157657 | ZNF618 | 8.9713936 | 6.8276804 | -2.143713 | 3.51E-14 | 2.41E-12 |
| ENSG00000064692 | SNCAIP | 2.4695446 | 0.3262375 | -2.143307 | 0.0002218 | 0.0020533 |
| ENSG00000104611 | SH2D4A | 3.8251413 | 1.6818983 | -2.143243 | 0.0007205 | 0.0056047 |
| ENSG00000151320 | AKAP6 | 7.4486529 | 5.3102848 | -2.138368 | 1.66E-06 | 2.78E-05 |
| ENSG00000079156 | OSBPL6 | 3.4222492 | 1.2845785 | -2.137671 | 4.53E-05 | 0.0005188 |
| ENSG00000005884 | ITGA3 | 10.519501 | 8.3886132 | -2.130888 | 7.01E-10 | 2.33E-08 |
| ENSG00000158715 | SLC45A3 | 7.9267876 | 5.7985741 | -2.128214 | 4.53E-15 | 3.55E-13 |
| ENSG00000174080 | CTSF | 9.2603264 | 7.135004 | -2.125322 | 4.66E-16 | 4.24E-14 |
| ENSG00000110195 | FOLR1 | 8.5015011 | 6.3816009 | -2.1199 | 6.60E-09 | 1.84E-07 |
| ENSG00000172247 | C1QTNF4 | 3.6626736 | 1.5475313 | -2.115142 | 0.0002062 | 0.0019341 |
| ENSG00000175600 | SUGCT | 3.0963028 | 0.9839685 | -2.112334 | 0.001901 | 0.0126963 |
| ENSG00000185313 | SCN10A | 2.1120504 | 0 | -2.11205 | 2.57E-06 | 4.06E-05 |
| ENSG00000168453 | HR | 4.6765991 | 2.5647792 | -2.11182 | 0.0011434 | 0.0082906 |
| ENSG00000143153 | ATP1B1 | 12.020036 | 9.9111123 | -2.108924 | 4.32E-16 | 3.96E-14 |
| ENSG00000139174 | PRICKLE1 | 6.4028382 | 4.296979 | -2.105859 | 2.19E-07 | 4.50E-06 |
| ENSG00000131910 | NR0B2 | 3.0051691 | 0.9060367 | -2.099132 | 7.88E-06 | 0.0001111 |
| ENSG00000183773 | AIFM3 | 7.3719373 | 5.2779134 | -2.094024 | 8.12E-08 | 1.84E-06 |
| ENSG00000130598 | TNNI2 | 7.7464566 | 5.6645107 | -2.081946 | 1.55E-11 | 6.85E-10 |
| ENSG00000165078 | CPA6 | 4.1910321 | 2.109104 | -2.081928 | 0.001008 | 0.007462 |
| ENSG00000126500 | FLRT1 | 4.8864631 | 2.812159 | -2.074304 | 0.0006788 | 0.005327 |
| ENSG00000135931 | ARMC9 | 12.475317 | 10.40129 | -2.074027 | 2.16E-22 | 4.20E-20 |
| ENSG00000148826 | NKX6-2 | 3.3990052 | 1.3271206 | -2.071885 | 0.0017918 | 0.0120853 |
| ENSG00000135914 | HTR2B | 10.359492 | 8.2879686 | -2.071524 | 1.58E-19 | 2.26E-17 |
| ENSG00000101440 | ASIP | 3.6106387 | 1.5399948 | -2.070644 | 3.73E-05 | 0.0004369 |
| ENSG00000131941 | RHPN2 | 5.0441057 | 2.9747215 | -2.069384 | 0.0003218 | 0.0028242 |
| ENSG00000136235 | GPNMB | 18.931201 | 16.869715 | -2.061486 | 1.91E-19 | 2.72E-17 |
| ENSG00000162407 | PLPP3 | 12.629399 | 10.581359 | -2.04804 | 0.000691 | 0.0054137 |
| ENSG00000123453 | SARDH | 5.0098293 | 2.963547 | -2.046282 | 0.0041477 | 0.024473 |
| ENSG00000230601 | TEX48 | 2.5300625 | 0.483837 | -2.046225 | 6.48E-05 | 0.0007077 |
| ENSG00000170323 | FABP4 | 8.9088552 | 6.8659249 | -2.04293 | 0.0007936 | 0.0060749 |
| ENSG00000141096 | DPEP3 | 4.8016804 | 2.7602476 | -2.041433 | 0.0020439 | 0.0134598 |
| ENSG00000109452 | INPP4B | 5.9863326 | 3.9452369 | -2.041096 | 1.03E-07 | 2.28E-06 |
| ENSG00000172236 | TPSAB1 | 2.9105555 | 0.8715087 | -2.039047 | 0.0007291 | 0.0056589 |
| ENSG00000103253 | HAGHL | 7.9741372 | 5.939149 | -2.034988 | 1.29E-15 | 1.11E-13 |
| ENSG00000171056 | SOX7 | 2.8495595 | 0.8167923 | -2.032767 | 0.0090685 | 0.046301 |
| ENSG00000107562 | CXCL12 | 8.5972513 | 6.5714072 | -2.025844 | 6.02E-05 | 0.0006639 |
| ENSG00000218819 | TDRD15 | 3.9551257 | 1.9369845 | -2.018141 | 1.02E-06 | 1.79E-05 |
| ENSG00000184261 | KCNK12 | 4.4568927 | 2.4430857 | -2.013807 | 0.0006085 | 0.0048435 |
| ENSG00000168427 | KLHL30 | 3.727091 | 1.7180546 | -2.009036 | 0.0011215 | 0.0081563 |
| ENSG00000078269 | SYNJ2 | 9.5761242 | 7.5711349 | -2.004989 | 1.44E-10 | 5.35E-09 |
| ENSG00000067177 | PHKA1 | 7.6504949 | 5.6463075 | -2.004187 | 1.85E-13 | 1.12E-11 |
| ENSG00000157470 | FAM81A | 5.5522108 | 3.5515963 | -2.000615 | 0.0002468 | 0.002252 |
| ENSG00000137198 | GMPR | 8.695553 | 6.6980523 | -1.997501 | 4.97E-20 | 7.64E-18 |
| ENSG00000176658 | MYO1D | 11.097294 | 9.1026922 | -1.994601 | 3.68E-07 | 7.17E-06 |
| ENSG00000196415 | PRTN3 | 2.2468111 | 0.2544503 | -1.992361 | 0.0024889 | 0.0159002 |
| ENSG00000010278 | CD9 | 14.253023 | 12.266909 | -1.986114 | 1.17E-15 | 1.01E-13 |
| ENSG00000205863 | C1QTNF9B | 3.2350194 | 1.2505897 | -1.98443 | 0.0021799 | 0.0142097 |
| ENSG00000182749 | PAQR7 | 8.2295627 | 6.2456579 | -1.983905 | 2.72E-15 | 2.21E-13 |
| ENSG00000100600 | LGMN | 17.117843 | 15.152577 | -1.965265 | 3.63E-08 | 8.79E-07 |
| ENSG00000141577 | CEP131 | 9.2239566 | 7.2596719 | -1.964285 | 2.90E-29 | 1.19E-26 |
| ENSG00000167261 | DPEP2 | 11.001425 | 9.0391635 | -1.962262 | 3.04E-10 | 1.08E-08 |
| ENSG00000120756 | PLS1 | 7.3211873 | 5.3742639 | -1.946923 | 5.45E-14 | 3.59E-12 |
| ENSG00000134955 | SLC37A2 | 14.153292 | 12.208159 | -1.945133 | 1.82E-09 | 5.62E-08 |
| ENSG00000188404 | SELL | 8.8625576 | 6.9196246 | -1.942933 | 2.25E-23 | 4.86E-21 |
| ENSG00000176092 | CRYBG2 | 3.4967686 | 1.5548197 | -1.941949 | 0.0047193 | 0.0271663 |
| ENSG00000180767 | CHST13 | 8.0768974 | 6.1358152 | -1.941082 | 1.11E-10 | 4.22E-09 |
| ENSG00000167363 | FN3K | 7.1399433 | 5.1997577 | -1.940186 | 2.49E-07 | 5.08E-06 |
| ENSG00000204228 | HSD17B8 | 5.7560999 | 3.8167869 | -1.939313 | 2.09E-08 | 5.31E-07 |
| ENSG00000196666 | FAM180B | 4.8082356 | 2.870023 | -1.938213 | 0.0001386 | 0.0013719 |
| ENSG00000129951 | PLPPR3 | 5.4697511 | 3.5317567 | -1.937994 | 1.59E-14 | 1.14E-12 |
| ENSG00000164649 | CDCA7L | 9.8944687 | 7.9584155 | -1.936053 | 9.35E-12 | 4.32E-10 |
| ENSG00000117245 | KIF17 | 5.8850579 | 3.9490319 | -1.936026 | 3.99E-10 | 1.40E-08 |
| ENSG00000185818 | NAT8L | 5.0221982 | 3.0891142 | -1.933084 | 0.0053079 | 0.029821 |
| ENSG00000090447 | TFAP4 | 7.9143297 | 5.9824317 | -1.931898 | 7.87E-07 | 1.42E-05 |
| ENSG00000183579 | ZNRF3 | 4.8689651 | 2.9376863 | -1.931279 | 0.0031479 | 0.0193717 |
| ENSG00000179163 | FUCA1 | 16.512733 | 14.581533 | -1.9312 | 0.0002093 | 0.001959 |
| ENSG00000172985 | SH3RF3 | 9.5767206 | 7.6515364 | -1.925184 | 0.0013957 | 0.0098099 |
| ENSG00000067533 | RRP15 | 3.0603121 | 1.1399025 | -1.92041 | 0.0001904 | 0.0018039 |
| ENSG00000143502 | SUSD4 | 3.7712901 | 1.8512236 | -1.920066 | 8.48E-05 | 0.0008955 |
| ENSG00000163554 | SPTA1 | 4.4660002 | 2.5479176 | -1.918083 | 0.0017935 | 0.0120949 |
| ENSG00000162144 | CYB561A3 | 12.808497 | 10.892841 | -1.915656 | 0.002116 | 0.0138597 |
| ENSG00000086159 | AQP6 | 6.8156936 | 4.9149582 | -1.900735 | 0.0014911 | 0.0103738 |
| ENSG00000233198 | RNF224 | 5.3531978 | 3.4578446 | -1.895353 | 2.74E-05 | 0.0003319 |
| ENSG00000179862 | CITED4 | 3.0336771 | 1.1442189 | -1.889458 | 0.0003958 | 0.0033658 |
| ENSG00000108176 | DNAJC12 | 3.7335657 | 1.8470711 | -1.886495 | 0.0004149 | 0.0035004 |
| ENSG00000154227 | CERS3 | 2.7861991 | 0.9046201 | -1.881579 | 0.0082894 | 0.0429799 |
| ENSG00000163331 | DAPL1 | 2.7100124 | 0.835188 | -1.874824 | 0.0049346 | 0.0281684 |
| ENSG00000175287 | PHYHD1 | 6.3321762 | 4.4593746 | -1.872802 | 9.07E-12 | 4.22E-10 |
| ENSG00000017483 | SLC38A5 | 3.6634168 | 1.7923753 | -1.871042 | 0.0030461 | 0.0188323 |
| ENSG00000257446 | ZNF878 | 5.1716099 | 3.3017228 | -1.869887 | 1.79E-13 | 1.09E-11 |
| ENSG00000112425 | EPM2A | 9.4463301 | 7.577143 | -1.869187 | 5.85E-16 | 5.28E-14 |
| ENSG00000181001 | OR52N1 | 2.5463468 | 0.6784406 | -1.867906 | 0.0012925 | 0.0092058 |
| ENSG00000147614 | ATP6V0D2 | 11.376256 | 9.5114304 | -1.864826 | 0.0023708 | 0.0152479 |
| ENSG00000054793 | ATP9A | 7.7149525 | 5.85579 | -1.859162 | 0.009763 | 0.0491136 |
| ENSG00000049246 | PER3 | 11.967007 | 10.113014 | -1.853993 | 1.28E-10 | 4.80E-09 |
| ENSG00000114487 | MORC1 | 2.6883532 | 0.8344123 | -1.853941 | 0.0010139 | 0.0075004 |
| ENSG00000178718 | RPP25 | 9.39879 | 7.54947 | -1.84932 | 1.55E-07 | 3.28E-06 |
| ENSG00000110799 | VWF | 8.0426505 | 6.2058154 | -1.836835 | 1.47E-06 | 2.49E-05 |
| ENSG00000176974 | SHMT1 | 9.8758812 | 8.0398049 | -1.836076 | 1.27E-13 | 7.91E-12 |
| ENSG00000130513 | GDF15 | 8.9020252 | 7.0664608 | -1.835564 | 4.22E-31 | 2.12E-28 |
| ENSG00000165092 | ALDH1A1 | 14.406858 | 12.571369 | -1.835489 | 4.64E-19 | 6.32E-17 |
| ENSG00000242715 | CCDC169 | 3.0426825 | 1.2075849 | -1.835098 | 0.0032026 | 0.019645 |
| ENSG00000101203 | COL20A1 | 1.8307887 | 0 | -1.830789 | 0.0002505 | 0.0022804 |
| ENSG00000184916 | JAG2 | 5.9824865 | 4.1531657 | -1.829321 | 6.88E-05 | 0.0007459 |
| ENSG00000186710 | CFAP73 | 4.4101438 | 2.5867549 | -1.823389 | 0.0009419 | 0.0070446 |
| ENSG00000198947 | DMD | 7.9197165 | 6.0991121 | -1.820604 | 9.54E-05 | 0.0009913 |
| ENSG00000164344 | KLKB1 | 5.3228707 | 3.5027616 | -1.820109 | 0.0054917 | 0.0306922 |
| ENSG00000162066 | AMDHD2 | 11.134749 | 9.3169572 | -1.817792 | 4.93E-11 | 2.00E-09 |
| ENSG00000131981 | LGALS3 | 14.382209 | 12.56538 | -1.81683 | 5.81E-16 | 5.26E-14 |
| ENSG00000138031 | ADCY3 | 11.644978 | 9.8310398 | -1.813938 | 1.94E-07 | 4.03E-06 |
| ENSG00000196220 | SRGAP3 | 11.828035 | 10.015515 | -1.81252 | 1.52E-08 | 3.96E-07 |
| ENSG00000143195 | ILDR2 | 2.8012635 | 0.9893244 | -1.811939 | 0.0051148 | 0.028948 |
| ENSG00000125246 | CLYBL | 7.5517811 | 5.7417742 | -1.810007 | 1.51E-08 | 3.94E-07 |
| ENSG00000219481 | NBPF1 | 13.60694 | 11.798298 | -1.808642 | 2.54E-16 | 2.40E-14 |
| ENSG00000198569 | SLC34A3 | 6.1058137 | 4.303589 | -1.802225 | 1.09E-11 | 4.98E-10 |
| ENSG00000104814 | MAP4K1 | 7.543612 | 5.7438359 | -1.799776 | 5.29E-18 | 6.24E-16 |
| ENSG00000245848 | CEBPA | 11.172731 | 9.3794835 | -1.793247 | 1.99E-09 | 6.09E-08 |
| ENSG00000241484 | ARHGAP8 | 6.5271393 | 4.7435624 | -1.783577 | 0.0090732 | 0.0463158 |
| ENSG00000132182 | NUP210 | 9.9568789 | 8.1752824 | -1.781596 | 7.81E-11 | 3.04E-09 |
| ENSG00000138336 | TET1 | 5.245718 | 3.466004 | -1.779714 | 7.07E-10 | 2.35E-08 |
| ENSG00000130208 | APOC1 | 12.47238 | 10.693756 | -1.778624 | 9.36E-11 | 3.59E-09 |
| ENSG00000074416 | MGLL | 14.428138 | 12.65682 | -1.771318 | 1.72E-08 | 4.46E-07 |
| ENSG00000101412 | E2F1 | 6.3538067 | 4.5879335 | -1.765873 | 4.94E-06 | 7.30E-05 |
| ENSG00000173208 | ABCD2 | 3.835407 | 2.0739369 | -1.76147 | 0.0017097 | 0.0116351 |
| ENSG00000117215 | PLA2G2D | 4.9107313 | 3.1517928 | -1.758938 | 0.0003821 | 0.0032666 |
| ENSG00000167968 | DNASE1L2 | 3.737412 | 1.9795636 | -1.757848 | 0.0001039 | 0.001066 |
| ENSG00000143842 | SOX13 | 9.7592862 | 8.0039835 | -1.755303 | 1.32E-07 | 2.85E-06 |
| ENSG00000112137 | PHACTR1 | 11.666086 | 9.9138381 | -1.752248 | 4.45E-06 | 6.65E-05 |
| ENSG00000090661 | CERS4 | 9.2738941 | 7.5257936 | -1.7481 | 1.13E-16 | 1.12E-14 |
| ENSG00000250479 | CHCHD10 | 7.8470242 | 6.0990137 | -1.74801 | 4.47E-11 | 1.83E-09 |
| ENSG00000164237 | CMBL | 8.1160053 | 6.3716612 | -1.744344 | 0.0001814 | 0.0017293 |
| ENSG00000142494 | SLC47A1 | 9.8392557 | 8.0968178 | -1.742438 | 2.51E-11 | 1.07E-09 |
| ENSG00000213366 | GSTM2 | 6.6531476 | 4.9151992 | -1.737948 | 6.21E-06 | 8.95E-05 |
| ENSG00000242574 | HLA-DMB | 13.829622 | 12.0924 | -1.737223 | 2.25E-08 | 5.68E-07 |
| ENSG00000154930 | ACSS1 | 11.387791 | 9.6561481 | -1.731643 | 4.47E-09 | 1.29E-07 |
| ENSG00000147883 | CDKN2B | 8.1971619 | 6.4664024 | -1.73076 | 5.03E-10 | 1.73E-08 |
| ENSG00000126368 | NR1D1 | 8.4306836 | 6.707784 | -1.7229 | 1.26E-06 | 2.16E-05 |
| ENSG00000184058 | TBX1 | 6.5647576 | 4.8420423 | -1.722715 | 3.51E-07 | 6.87E-06 |
| ENSG00000069424 | KCNAB2 | 13.248918 | 11.530794 | -1.718124 | 1.88E-08 | 4.81E-07 |
| ENSG00000163909 | HEYL | 4.991113 | 3.2745517 | -1.716561 | 0.006731 | 0.0363016 |
| ENSG00000010219 | DYRK4 | 11.164079 | 9.4563977 | -1.707681 | 2.05E-12 | 1.04E-10 |
| ENSG00000128253 | RFPL2 | 3.5984839 | 1.8915472 | -1.706937 | 0.0005036 | 0.004132 |
| ENSG00000105327 | BBC3 | 7.992399 | 6.2858006 | -1.706598 | 1.06E-11 | 4.87E-10 |
| ENSG00000084710 | EFR3B | 7.2922489 | 5.5883153 | -1.703934 | 1.33E-05 | 0.0001757 |
| ENSG00000183087 | GAS6 | 10.903848 | 9.2005434 | -1.703304 | 6.27E-09 | 1.76E-07 |
| ENSG00000185838 | GNB1L | 9.1710687 | 7.4689013 | -1.702167 | 4.76E-08 | 1.13E-06 |
| ENSG00000073536 | NLE1 | 5.5601279 | 3.8595316 | -1.700596 | 0.0084489 | 0.0436898 |
| ENSG00000092051 | JPH4 | 5.0908791 | 3.3997327 | -1.691146 | 0.0061059 | 0.033492 |
| ENSG00000185345 | PRKN | 6.731282 | 5.0402553 | -1.691027 | 2.01E-05 | 0.0002536 |
| ENSG00000115718 | PROC | 5.1917621 | 3.501187 | -1.690575 | 2.17E-06 | 3.52E-05 |
| ENSG00000166211 | SPIC | 2.5034625 | 0.8170669 | -1.686396 | 0.0061032 | 0.0334814 |
| ENSG00000095970 | TREM2 | 11.785117 | 10.101471 | -1.683646 | 1.53E-07 | 3.25E-06 |
| ENSG00000164082 | GRM2 | 3.83985 | 2.1582231 | -1.681627 | 0.0035366 | 0.0213614 |
| ENSG00000114770 | ABCC5 | 14.171913 | 12.491525 | -1.680389 | 5.77E-24 | 1.34E-21 |
| ENSG00000169255 | B3GALNT1 | 7.1740531 | 5.4943502 | -1.679703 | 1.51E-08 | 3.95E-07 |
| ENSG00000123570 | RAB9B | 3.7193469 | 2.0400204 | -1.679327 | 3.79E-05 | 0.0004426 |
| ENSG00000274286 | ADRA2B | 8.1602104 | 6.4813354 | -1.678875 | 0.0030583 | 0.0189001 |
| ENSG00000063761 | ADCK1 | 10.021976 | 8.34765 | -1.674326 | 3.12E-12 | 1.53E-10 |
| ENSG00000072858 | SIDT1 | 7.2448133 | 5.5722164 | -1.672597 | 6.51E-05 | 0.0007105 |
| ENSG00000178562 | CD28 | 7.4583998 | 5.7902242 | -1.668176 | 0.0001005 | 0.0010356 |
| ENSG00000142875 | PRKACB | 12.658436 | 10.991664 | -1.666772 | 1.07E-28 | 4.13E-26 |
| ENSG00000137936 | BCAR3 | 9.0507106 | 7.3860942 | -1.664616 | 8.37E-07 | 1.50E-05 |
| ENSG00000071242 | RPS6KA2 | 12.817142 | 11.155753 | -1.66139 | 5.79E-06 | 8.43E-05 |
| ENSG00000167711 | SERPINF2 | 7.7213951 | 6.0608072 | -1.660588 | 3.36E-07 | 6.62E-06 |
| ENSG00000184428 | TOP1MT | 8.4476579 | 6.7890948 | -1.658563 | 1.26E-10 | 4.74E-09 |
| ENSG00000165060 | FXN | 7.5288076 | 5.8754952 | -1.653312 | 0.0003432 | 0.0029834 |
| ENSG00000197520 | FAM177B | 5.5912702 | 3.9384357 | -1.652835 | 1.99E-07 | 4.13E-06 |
| ENSG00000130881 | LRP3 | 7.2538021 | 5.6017548 | -1.652047 | 1.78E-06 | 2.97E-05 |
| ENSG00000112902 | SEMA5A | 4.8436564 | 3.1917892 | -1.651867 | 0.0044015 | 0.025678 |
| ENSG00000165409 | TSHR | 6.7160305 | 5.0651523 | -1.650878 | 2.01E-07 | 4.15E-06 |
| ENSG00000089169 | RPH3A | 1.6442118 | 0 | -1.644212 | 0.0010666 | 0.0078243 |
| ENSG00000185252 | ZNF74 | 7.2391392 | 5.5955233 | -1.643616 | 1.22E-08 | 3.23E-07 |
| ENSG00000172232 | AZU1 | 7.2484237 | 5.6066643 | -1.641759 | 0.0001179 | 0.0011906 |
| ENSG00000136999 | NOV | 8.1353983 | 6.4947378 | -1.64066 | 4.14E-05 | 0.0004791 |
| ENSG00000150054 | MPP7 | 7.6915124 | 6.0529286 | -1.638584 | 7.14E-05 | 0.0007709 |
| ENSG00000166816 | LDHD | 7.3754571 | 5.7373347 | -1.638122 | 0.0007758 | 0.0059627 |
| ENSG00000121769 | FABP3 | 10.852285 | 9.2152525 | -1.637033 | 0.0043216 | 0.0252929 |
| ENSG00000154016 | GRAP | 6.2590726 | 4.6240999 | -1.634973 | 0.0002681 | 0.0024155 |
| ENSG00000035499 | DEPDC1B | 3.3741464 | 1.7398498 | -1.634297 | 0.0001887 | 0.0017894 |
| ENSG00000152990 | ADGRA3 | 6.7757037 | 5.1461296 | -1.629574 | 8.80E-06 | 0.0001225 |
| ENSG00000070759 | TESK2 | 10.013394 | 8.3850145 | -1.628379 | 7.54E-14 | 4.84E-12 |
| ENSG00000155792 | DEPTOR | 8.5254201 | 6.8973707 | -1.628049 | 0.0015246 | 0.010581 |
| ENSG00000158427 | TMSB15B | 2.5035105 | 0.8803973 | -1.623113 | 0.0023732 | 0.0152612 |
| ENSG00000196693 | ZNF33B | 9.0901259 | 7.4733776 | -1.616748 | 0.0004054 | 0.0034326 |
| ENSG00000166025 | AMOTL1 | 6.8783737 | 5.2643638 | -1.61401 | 4.02E-11 | 1.66E-09 |
| ENSG00000153446 | C16orf89 | 4.1491729 | 2.5363031 | -1.61287 | 0.0099418 | 0.0498263 |
| ENSG00000170006 | TMEM154 | 5.4777125 | 3.8787849 | -1.598928 | 5.00E-09 | 1.42E-07 |
| ENSG00000116852 | KIF21B | 10.49369 | 8.8981886 | -1.595501 | 1.92E-14 | 1.36E-12 |
| ENSG00000114554 | PLXNA1 | 13.21951 | 11.62538 | -1.594129 | 3.15E-09 | 9.27E-08 |
| ENSG00000159023 | EPB41 | 12.123335 | 10.530289 | -1.593046 | 3.41E-14 | 2.35E-12 |
| ENSG00000174370 | C11orf45 | 11.603333 | 10.010765 | -1.592568 | 1.67E-09 | 5.18E-08 |
| ENSG00000204252 | HLA-DOA | 10.164813 | 8.5729067 | -1.591907 | 0.0005788 | 0.004644 |
| ENSG00000168646 | AXIN2 | 3.9121172 | 2.3211002 | -1.591017 | 0.0031613 | 0.0194348 |
| ENSG00000130653 | PNPLA7 | 10.414542 | 8.8246992 | -1.589843 | 1.95E-06 | 3.20E-05 |
| ENSG00000164742 | ADCY1 | 7.3143525 | 5.7273426 | -1.58701 | 8.22E-07 | 1.48E-05 |
| ENSG00000163995 | ABLIM2 | 4.7832068 | 3.198124 | -1.585083 | 0.0007724 | 0.005942 |
| ENSG00000099822 | HCN2 | 5.5333978 | 3.9483904 | -1.585007 | 1.12E-05 | 0.0001512 |
| ENSG00000080293 | SCTR | 7.0096455 | 5.4249035 | -1.584742 | 7.00E-08 | 1.60E-06 |
| ENSG00000160145 | KALRN | 6.6008905 | 5.0169994 | -1.583891 | 0.0076796 | 0.0403991 |
| ENSG00000181045 | SLC26A11 | 11.476377 | 9.8931126 | -1.583265 | 0.0043674 | 0.0255162 |
| ENSG00000101444 | AHCY | 11.610149 | 10.028002 | -1.582147 | 1.45E-11 | 6.43E-10 |
| ENSG00000163082 | SGPP2 | 9.9130641 | 8.3314051 | -1.581659 | 4.27E-08 | 1.02E-06 |
| ENSG00000166257 | SCN3B | 5.839223 | 4.2648606 | -1.574362 | 4.17E-06 | 6.29E-05 |
| ENSG00000143845 | ETNK2 | 4.6353016 | 3.0631834 | -1.572118 | 0.0076267 | 0.0401916 |
| ENSG00000112561 | TFEB | 11.751275 | 10.179941 | -1.571333 | 1.31E-09 | 4.17E-08 |
| ENSG00000177106 | EPS8L2 | 2.7644528 | 1.1945778 | -1.569875 | 0.0019305 | 0.0128677 |
| ENSG00000112149 | CD83 | 11.776788 | 10.207288 | -1.5695 | 0.001249 | 0.0089361 |
| ENSG00000162694 | EXTL2 | 7.8813545 | 6.314186 | -1.567169 | 6.32E-15 | 4.79E-13 |
| ENSG00000022567 | SLC45A4 | 12.394627 | 10.829987 | -1.564641 | 1.90E-06 | 3.14E-05 |
| ENSG00000171811 | CFAP46 | 1.7770604 | 0.2138803 | -1.56318 | 0.003427 | 0.0208141 |
| ENSG00000128973 | CLN6 | 10.332825 | 8.7711189 | -1.561706 | 2.80E-15 | 2.27E-13 |
| ENSG00000103356 | EARS2 | 8.3430756 | 6.7872743 | -1.555801 | 1.02E-05 | 0.0001395 |
| ENSG00000132386 | SERPINF1 | 10.962932 | 9.4076151 | -1.555317 | 1.04E-14 | 7.69E-13 |
| ENSG00000148680 | HTR7 | 9.6268856 | 8.0754651 | -1.55142 | 7.94E-15 | 5.97E-13 |
| ENSG00000157873 | TNFRSF14 | 13.14586 | 11.596446 | -1.549414 | 1.67E-07 | 3.52E-06 |
| ENSG00000107833 | NPM3 | 6.4234029 | 4.8746378 | -1.548765 | 0.0003012 | 0.0026723 |
| ENSG00000111247 | RAD51AP1 | 9.2235255 | 7.67563 | -1.547896 | 1.85E-08 | 4.73E-07 |
| ENSG00000183785 | TUBA8 | 5.5839465 | 4.0460697 | -1.537877 | 0.0001555 | 0.0015147 |
| ENSG00000124334 | IL9R | 1.7748155 | 0.2387372 | -1.536078 | 0.0090493 | 0.0462084 |
| ENSG00000197191 | CYSRT1 | 5.1029504 | 3.5676852 | -1.535265 | 1.75E-05 | 0.0002234 |
| ENSG00000036549 | ZZZ3 | 11.532484 | 9.9975561 | -1.534927 | 8.13E-16 | 7.14E-14 |
| ENSG00000165626 | BEND7 | 1.7242701 | 0.1900613 | -1.534209 | 0.0022821 | 0.0147443 |
| ENSG00000139344 | AMDHD1 | 8.5354261 | 7.0014828 | -1.533943 | 2.71E-13 | 1.59E-11 |
| ENSG00000184602 | SNN | 12.857202 | 11.323772 | -1.53343 | 7.23E-10 | 2.40E-08 |
| ENSG00000099260 | PALMD | 4.8749034 | 3.3425369 | -1.532367 | 0.0050295 | 0.0286049 |
| ENSG00000103495 | MAZ | 10.924309 | 9.3951407 | -1.529169 | 5.40E-08 | 1.27E-06 |
| ENSG00000100033 | PRODH | 4.1835654 | 2.6594715 | -1.524094 | 0.0004744 | 0.0039253 |
| ENSG00000136169 | SETDB2 | 12.668891 | 11.14554 | -1.523352 | 1.72E-05 | 0.00022 |
| ENSG00000172159 | FRMD3 | 7.6863691 | 6.1646513 | -1.521718 | 1.82E-08 | 4.67E-07 |
| ENSG00000197980 | LEKR1 | 4.3375898 | 2.8165577 | -1.521032 | 1.40E-05 | 0.0001836 |
| ENSG00000234409 | CCDC188 | 3.4912776 | 1.9718988 | -1.519379 | 0.0006512 | 0.0051418 |
| ENSG00000182378 | PLCXD1 | 10.663608 | 9.145222 | -1.518386 | 2.38E-07 | 4.87E-06 |
| ENSG00000214787 | MS4A4E | 7.9921374 | 6.4747155 | -1.517422 | 9.15E-05 | 0.0009551 |
| ENSG00000169676 | DRD5 | 6.313394 | 4.7975568 | -1.515837 | 4.66E-11 | 1.90E-09 |
| ENSG00000166444 | ST5 | 9.5089606 | 7.9980233 | -1.510937 | 2.59E-11 | 1.10E-09 |
| ENSG00000153002 | CPB1 | 5.0448853 | 3.5341779 | -1.510707 | 1.65E-05 | 0.0002124 |
| ENSG00000101255 | TRIB3 | 6.5889786 | 5.07997 | -1.509009 | 0.0043362 | 0.0253645 |
| ENSG00000171790 | SLFNL1 | 4.782267 | 3.2737597 | -1.508507 | 0.0040643 | 0.0240408 |
| ENSG00000198865 | CCDC152 | 10.804679 | 9.3007621 | -1.503917 | 0.0005659 | 0.0045542 |
| ENSG00000120693 | SMAD9 | 5.7393067 | 4.2387424 | -1.500564 | 3.34E-07 | 6.60E-06 |
| ENSG00000137411 | VARS2 | 9.1622944 | 7.6652249 | -1.497069 | 1.36E-05 | 0.0001795 |
| ENSG00000185215 | TNFAIP2 | 14.268776 | 12.772745 | -1.496031 | 3.10E-08 | 7.62E-07 |
| ENSG00000204248 | COL11A2 | 3.9707781 | 2.474947 | -1.495831 | 0.0037523 | 0.0224534 |
| ENSG00000165138 | ANKS6 | 5.5989723 | 4.1038275 | -1.495145 | 0.001778 | 0.0120178 |
| ENSG00000066926 | FECH | 10.614185 | 9.1207992 | -1.493386 | 1.03E-05 | 0.0001399 |
| ENSG00000171813 | PWWP2B | 9.1748696 | 7.6826441 | -1.492226 | 8.82E-13 | 4.76E-11 |
| ENSG00000136732 | GYPC | 11.073452 | 9.581548 | -1.491904 | 3.76E-11 | 1.56E-09 |
| ENSG00000142694 | EVA1B | 6.3314152 | 4.8413791 | -1.490036 | 0.0001366 | 0.0013547 |
| ENSG00000165192 | ASB11 | 2.7591493 | 1.2704327 | -1.488717 | 0.0003614 | 0.0031179 |
| ENSG00000150687 | PRSS23 | 5.0659158 | 3.5784425 | -1.487473 | 0.0004272 | 0.0035933 |
| ENSG00000073111 | MCM2 | 8.9910806 | 7.5038135 | -1.487267 | 1.93E-05 | 0.0002444 |
| ENSG00000155846 | PPARGC1B | 12.057299 | 10.570282 | -1.487017 | 0.0006016 | 0.0048029 |
| ENSG00000181009 | OR52N5 | 2.0473883 | 0.5604946 | -1.486894 | 0.0087488 | 0.0448992 |
| ENSG00000126351 | THRA | 11.005668 | 9.5196255 | -1.486043 | 1.70E-10 | 6.25E-09 |
| ENSG00000119599 | DCAF4 | 8.2384556 | 6.7535066 | -1.484949 | 4.18E-06 | 6.30E-05 |
| ENSG00000162174 | ASRGL1 | 10.185352 | 8.7043259 | -1.481026 | 5.47E-08 | 1.28E-06 |
| ENSG00000175764 | TTLL11 | 6.6228817 | 5.1421397 | -1.480742 | 1.81E-12 | 9.27E-11 |
| ENSG00000189007 | ADAT2 | 8.0385442 | 6.5584975 | -1.480047 | 2.59E-08 | 6.48E-07 |
| ENSG00000007255 | TRAPPC6A | 8.0885697 | 6.608871 | -1.479699 | 3.32E-07 | 6.55E-06 |
| ENSG00000163564 | PYHIN1 | 7.8899773 | 6.4110755 | -1.478902 | 9.62E-05 | 0.0009987 |
| ENSG00000117984 | CTSD | 16.829185 | 15.350411 | -1.478774 | 3.60E-08 | 8.75E-07 |
| ENSG00000197256 | KANK2 | 9.5371088 | 8.0644041 | -1.472705 | 7.36E-13 | 4.02E-11 |
| ENSG00000180697 | C3orf22 | 5.993149 | 4.5206198 | -1.472529 | 3.50E-10 | 1.24E-08 |
| ENSG00000143819 | EPHX1 | 12.582157 | 11.110342 | -1.471815 | 5.19E-22 | 9.63E-20 |
| ENSG00000022267 | FHL1 | 6.3994952 | 4.9306348 | -1.46886 | 0.0010816 | 0.0079172 |
| ENSG00000106952 | TNFSF8 | 10.532767 | 9.0651351 | -1.467632 | 4.03E-08 | 9.70E-07 |
| ENSG00000226650 | KIF4B | 1.4653538 | 0 | -1.465354 | 0.0013896 | 0.0097758 |
| ENSG00000188833 | ENTPD8 | 4.505721 | 3.0417256 | -1.463995 | 0.0001699 | 0.0016355 |
| ENSG00000206535 | LNP1 | 4.4431624 | 2.9803733 | -1.462789 | 0.0003523 | 0.0030523 |
| ENSG00000214706 | IFRD2 | 7.9152907 | 6.4529054 | -1.462385 | 1.86E-05 | 0.0002359 |
| ENSG00000240891 | PLCXD2 | 5.6837457 | 4.2217428 | -1.462003 | 0.00117 | 0.0084626 |
| ENSG00000057593 | F7 | 1.4601222 | 0 | -1.460122 | 0.0027497 | 0.0172657 |
| ENSG00000164904 | ALDH7A1 | 9.2619302 | 7.8029387 | -1.458992 | 1.21E-16 | 1.19E-14 |
| ENSG00000161544 | CYGB | 6.8577297 | 5.4003039 | -1.457426 | 0.0023708 | 0.0152479 |
| ENSG00000105281 | SLC1A5 | 10.364675 | 8.9112564 | -1.453418 | 2.25E-05 | 0.0002806 |
| ENSG00000250722 | SELENOP | 15.542219 | 14.089341 | -1.452878 | 0.0001939 | 0.0018319 |
| ENSG00000162496 | DHRS3 | 11.797885 | 10.34554 | -1.452345 | 1.32E-14 | 9.60E-13 |
| ENSG00000198203 | SULT1C2 | 11.314532 | 9.863473 | -1.451059 | 0.0017696 | 0.0119707 |
| ENSG00000136444 | RSAD1 | 9.0396168 | 7.589681 | -1.449936 | 2.48E-09 | 7.47E-08 |
| ENSG00000174136 | RGMB | 4.826513 | 3.3769639 | -1.449549 | 0.0033117 | 0.0202037 |
| ENSG00000130751 | NPAS1 | 3.7602892 | 2.3107615 | -1.449528 | 0.000719 | 0.0055949 |
| ENSG00000100364 | KIAA0930 | 14.437065 | 12.989741 | -1.447324 | 9.14E-10 | 2.99E-08 |
| ENSG00000104808 | DHDH | 5.0298625 | 3.5832124 | -1.44665 | 0.0025158 | 0.0160363 |
| ENSG00000100321 | SYNGR1 | 7.998442 | 6.5551491 | -1.443293 | 4.91E-07 | 9.29E-06 |
| ENSG00000089041 | P2RX7 | 11.483196 | 10.040088 | -1.443108 | 3.67E-07 | 7.16E-06 |
| ENSG00000066923 | STAG3 | 8.8423955 | 7.3994919 | -1.442904 | 7.36E-06 | 0.0001048 |
| ENSG00000214063 | TSPAN4 | 11.5252 | 10.083666 | -1.441533 | 7.61E-08 | 1.73E-06 |
| ENSG00000226124 | FTCDNL1 | 6.2007784 | 4.7592637 | -1.441515 | 0.0039538 | 0.0234758 |
| ENSG00000130592 | LSP1 | 13.242963 | 11.80237 | -1.440593 | 4.48E-10 | 1.55E-08 |
| ENSG00000269190 | FBXO17 | 1.8152676 | 0.3756449 | -1.439623 | 0.001576 | 0.0108711 |
| ENSG00000102098 | SCML2 | 5.0959062 | 3.6576847 | -1.438222 | 0.0044164 | 0.0257287 |
| ENSG00000093217 | XYLB | 7.2616035 | 5.8255616 | -1.436042 | 2.04E-08 | 5.21E-07 |
| ENSG00000185745 | IFIT1 | 8.3892003 | 6.9531855 | -1.436015 | 5.71E-06 | 8.33E-05 |
| ENSG00000164236 | ANKRD33B | 5.2953659 | 3.8595203 | -1.435846 | 1.50E-05 | 0.0001952 |
| ENSG00000117266 | CDK18 | 8.8049331 | 7.374366 | -1.430567 | 1.57E-06 | 2.65E-05 |
| ENSG00000105699 | LSR | 9.326613 | 7.8976664 | -1.428947 | 1.18E-06 | 2.04E-05 |
| ENSG00000138439 | FAM117B | 8.9774478 | 7.555154 | -1.422294 | 3.46E-16 | 3.21E-14 |
| ENSG00000101198 | NKAIN4 | 2.1704078 | 0.7481673 | -1.422241 | 0.0037142 | 0.0222772 |
| ENSG00000183048 | SLC25A10 | 6.4592787 | 5.045918 | -1.413361 | 1.48E-06 | 2.51E-05 |
| ENSG00000108960 | MMD | 11.367415 | 9.9540729 | -1.413342 | 1.07E-09 | 3.45E-08 |
| ENSG00000135632 | SMYD5 | 7.83885 | 6.4281347 | -1.410715 | 6.54E-05 | 0.0007136 |
| ENSG00000101546 | RBFA | 7.9393598 | 6.5286449 | -1.410715 | 9.31E-08 | 2.08E-06 |
| ENSG00000136982 | DSCC1 | 5.438585 | 4.0288427 | -1.409742 | 0.0070237 | 0.0376436 |
| ENSG00000256162 | SMLR1 | 4.4423319 | 3.0336712 | -1.408661 | 0.0081742 | 0.0424971 |
| ENSG00000197046 | SIGLEC15 | 9.5590393 | 8.1523117 | -1.406728 | 6.57E-11 | 2.61E-09 |
| ENSG00000057757 | PITHD1 | 12.164065 | 10.757649 | -1.406416 | 4.34E-11 | 1.78E-09 |
| ENSG00000178922 | HYI | 6.6103586 | 5.2057091 | -1.40465 | 4.81E-08 | 1.14E-06 |
| ENSG00000163808 | KIF15 | 5.1724289 | 3.7684014 | -1.404028 | 0.0098974 | 0.0496468 |
| ENSG00000182584 | ACTL10 | 4.2591278 | 2.8588345 | -1.400293 | 0.0011063 | 0.0080645 |
| ENSG00000090621 | PABPC4 | 12.895398 | 11.495775 | -1.399624 | 4.21E-12 | 2.03E-10 |
| ENSG00000112773 | FAM46A | 11.546661 | 10.147596 | -1.399065 | 0.000114 | 0.0011577 |
| ENSG00000198934 | MAGEE1 | 3.8662834 | 2.4719093 | -1.394374 | 0.0013234 | 0.0093843 |
| ENSG00000163956 | LRPAP1 | 13.619717 | 12.225378 | -1.394339 | 2.10E-10 | 7.62E-09 |
| ENSG00000205213 | LGR4 | 9.8042605 | 8.4102363 | -1.394024 | 2.93E-05 | 0.0003527 |
| ENSG00000135929 | CYP27A1 | 14.916532 | 13.52276 | -1.393772 | 0.000286 | 0.002557 |
| ENSG00000104267 | CA2 | 10.458185 | 9.0654624 | -1.392722 | 4.78E-05 | 0.0005447 |
| ENSG00000023171 | GRAMD1B | 10.443501 | 9.0539324 | -1.389569 | 1.89E-07 | 3.95E-06 |
| ENSG00000101003 | GINS1 | 7.9516954 | 6.5629398 | -1.388756 | 2.93E-12 | 1.44E-10 |
| ENSG00000154277 | UCHL1 | 8.6441894 | 7.2565609 | -1.387628 | 0.0035743 | 0.0215593 |
| ENSG00000173511 | VEGFB | 11.327027 | 9.9397238 | -1.387304 | 4.11E-16 | 3.79E-14 |
| ENSG00000178921 | PFAS | 9.0047225 | 7.6221912 | -1.382531 | 3.89E-05 | 0.0004536 |
| ENSG00000170915 | PAQR8 | 10.430965 | 9.0495768 | -1.381388 | 4.33E-06 | 6.50E-05 |
| ENSG00000124181 | PLCG1 | 9.9415344 | 8.5608143 | -1.38072 | 1.21E-15 | 1.04E-13 |
| ENSG00000139187 | KLRG1 | 5.7023744 | 4.3217654 | -1.380609 | 9.06E-06 | 0.0001258 |
| ENSG00000013583 | HEBP1 | 11.807797 | 10.429273 | -1.378523 | 9.75E-17 | 9.83E-15 |
| ENSG00000175602 | CCDC85B | 6.0642275 | 4.6862984 | -1.377929 | 8.86E-05 | 0.0009291 |
| ENSG00000108773 | KAT2A | 9.6214696 | 8.24699 | -1.37448 | 4.00E-13 | 2.29E-11 |
| ENSG00000064201 | TSPAN32 | 6.732996 | 5.3587578 | -1.374238 | 0.000575 | 0.0046154 |
| ENSG00000168350 | DEGS2 | 5.8431111 | 4.470484 | -1.372627 | 6.66E-05 | 0.0007249 |
| ENSG00000049089 | COL9A2 | 5.4110118 | 4.0400945 | -1.370917 | 0.0096066 | 0.0485077 |
| ENSG00000162004 | CCDC78 | 6.4833074 | 5.1134564 | -1.369851 | 3.44E-07 | 6.76E-06 |
| ENSG00000134061 | CD180 | 11.851713 | 10.481963 | -1.36975 | 4.90E-11 | 1.99E-09 |
| ENSG00000117480 | FAAH | 5.6116559 | 4.2421916 | -1.369464 | 2.56E-07 | 5.20E-06 |
| ENSG00000162722 | TRIM58 | 8.1173364 | 6.7486892 | -1.368647 | 7.69E-05 | 0.0008229 |
| ENSG00000196976 | LAGE3 | 5.8917539 | 4.5272998 | -1.364454 | 0.0022517 | 0.0145778 |
| ENSG00000171680 | PLEKHG5 | 1.5012723 | 0.1410998 | -1.360172 | 0.0049813 | 0.0283781 |
| ENSG00000078900 | TP73 | 4.7732102 | 3.4149114 | -1.358299 | 0.0008292 | 0.0063115 |
| ENSG00000110931 | CAMKK2 | 11.188197 | 9.8326951 | -1.355502 | 5.63E-18 | 6.61E-16 |
| ENSG00000118420 | UBE3D | 7.0650166 | 5.7140326 | -1.350984 | 7.45E-08 | 1.69E-06 |
| ENSG00000103599 | IQCH | 5.0433147 | 3.6926149 | -1.3507 | 3.74E-05 | 0.0004379 |
| ENSG00000160293 | VAV2 | 10.032285 | 8.6852563 | -1.347029 | 2.50E-12 | 1.25E-10 |
| ENSG00000162975 | KCNF1 | 3.6479791 | 2.3024328 | -1.345546 | 0.0083534 | 0.0432658 |
| ENSG00000184178 | SCFD2 | 9.0253474 | 7.6803056 | -1.345042 | 8.32E-06 | 0.0001168 |
| ENSG00000015133 | CCDC88C | 10.513092 | 9.1707266 | -1.342366 | 0.0007011 | 0.0054842 |
| ENSG00000004660 | CAMKK1 | 9.4088673 | 8.0669098 | -1.341957 | 7.99E-07 | 1.44E-05 |
| ENSG00000180155 | LYNX1 | 6.8518864 | 5.510351 | -1.341535 | 3.76E-07 | 7.32E-06 |
| ENSG00000188211 | NCR3LG1 | 9.7788326 | 8.4378922 | -1.34094 | 0.0063939 | 0.0347994 |
| ENSG00000006432 | MAP3K9 | 7.316294 | 5.978827 | -1.337467 | 0.0003973 | 0.0033754 |
| ENSG00000108641 | B9D1 | 6.0516477 | 4.7157062 | -1.335941 | 4.70E-07 | 8.94E-06 |
| ENSG00000177191 | B3GNT8 | 8.3241271 | 6.989692 | -1.334435 | 8.13E-08 | 1.84E-06 |
| ENSG00000186603 | HPDL | 4.4029873 | 3.0692376 | -1.33375 | 0.0080871 | 0.0421207 |
| ENSG00000159788 | RGS12 | 11.555102 | 10.222859 | -1.332243 | 1.15E-15 | 9.96E-14 |
| ENSG00000162302 | RPS6KA4 | 10.049626 | 8.7174415 | -1.332184 | 1.07E-10 | 4.05E-09 |
| ENSG00000165912 | PACSIN3 | 4.3481414 | 3.0163222 | -1.331819 | 0.0009457 | 0.0070678 |
| ENSG00000185339 | TCN2 | 12.375716 | 11.046226 | -1.32949 | 2.45E-08 | 6.14E-07 |
| ENSG00000188807 | TMEM201 | 7.7004257 | 6.3715922 | -1.328834 | 9.15E-06 | 0.0001268 |
| ENSG00000186994 | KANK3 | 3.6835799 | 2.3548188 | -1.328761 | 0.0071867 | 0.0383436 |
| ENSG00000157870 | FAM213B | 10.382929 | 9.0555924 | -1.327337 | 2.58E-06 | 4.09E-05 |
| ENSG00000154447 | SH3RF1 | 10.235798 | 8.9087271 | -1.327071 | 1.19E-14 | 8.77E-13 |
| ENSG00000188130 | MAPK12 | 8.9709201 | 7.643995 | -1.326925 | 2.74E-13 | 1.61E-11 |
| ENSG00000184675 | AMER1 | 8.4836258 | 7.1576358 | -1.32599 | 1.06E-08 | 2.85E-07 |
| ENSG00000130005 | GAMT | 5.7205502 | 4.3959777 | -1.324572 | 1.99E-07 | 4.12E-06 |
| ENSG00000133619 | KRBA1 | 8.7166034 | 7.3927216 | -1.323882 | 2.08E-08 | 5.28E-07 |
| ENSG00000105516 | DBP | 10.380896 | 9.0594308 | -1.321465 | 4.00E-08 | 9.64E-07 |
| ENSG00000103264 | FBXO31 | 8.8948657 | 7.5736092 | -1.321257 | 1.33E-07 | 2.87E-06 |
| ENSG00000164048 | ZNF589 | 10.763089 | 9.4421826 | -1.320907 | 1.34E-11 | 5.99E-10 |
| ENSG00000121039 | RDH10 | 9.7145224 | 8.4008636 | -1.313659 | 5.82E-19 | 7.79E-17 |
| ENSG00000169696 | ASPSCR1 | 9.8687519 | 8.5552309 | -1.313521 | 5.00E-07 | 9.43E-06 |
| ENSG00000138030 | KHK | 6.9993234 | 5.6858188 | -1.313505 | 1.18E-08 | 3.14E-07 |
| ENSG00000120896 | SORBS3 | 12.713394 | 11.401559 | -1.311834 | 1.00E-07 | 2.23E-06 |
| ENSG00000065485 | PDIA5 | 7.2519288 | 5.9409846 | -1.310944 | 5.98E-06 | 8.67E-05 |
| ENSG00000101220 | C20orf27 | 9.0855213 | 7.7760176 | -1.309504 | 4.34E-06 | 6.51E-05 |
| ENSG00000084774 | CAD | 10.687281 | 9.3789676 | -1.308313 | 4.02E-10 | 1.40E-08 |
| ENSG00000107798 | LIPA | 15.520366 | 14.212208 | -1.308158 | 1.38E-06 | 2.35E-05 |
| ENSG00000063241 | ISOC2 | 8.8611652 | 7.5538831 | -1.307282 | 6.93E-06 | 9.90E-05 |
| ENSG00000160401 | CFAP157 | 5.802643 | 4.4954428 | -1.3072 | 3.09E-05 | 0.0003696 |
| ENSG00000137843 | PAK6 | 3.8465978 | 2.5414452 | -1.305153 | 0.0078043 | 0.0409244 |
| ENSG00000141540 | TTYH2 | 9.9634107 | 8.6603004 | -1.30311 | 5.81E-05 | 0.0006448 |
| ENSG00000167925 | GHDC | 9.869367 | 8.5702737 | -1.299093 | 6.93E-07 | 1.27E-05 |
| ENSG00000171045 | TSNARE1 | 8.9492989 | 7.6517805 | -1.297518 | 4.81E-08 | 1.14E-06 |
| ENSG00000089220 | PEBP1 | 12.43067 | 11.134651 | -1.296018 | 1.47E-12 | 7.65E-11 |
| ENSG00000197766 | CFD | 11.443838 | 10.148116 | -1.295722 | 7.82E-13 | 4.25E-11 |
| ENSG00000169246 | NPIPB3 | 11.597221 | 10.301813 | -1.295408 | 7.83E-14 | 5.01E-12 |
| ENSG00000162413 | KLHL21 | 10.658419 | 9.3632725 | -1.295146 | 8.99E-06 | 0.000125 |
| ENSG00000152056 | AP1S3 | 6.9600663 | 5.6649974 | -1.295069 | 5.43E-05 | 0.000608 |
| ENSG00000238243 | OR2W3 | 4.0582337 | 2.7635271 | -1.294707 | 0.0020442 | 0.0134598 |
| ENSG00000151491 | EPS8 | 10.312489 | 9.0212987 | -1.29119 | 7.20E-07 | 1.31E-05 |
| ENSG00000146072 | TNFRSF21 | 11.043567 | 9.7573247 | -1.286242 | 3.74E-05 | 0.0004376 |
| ENSG00000108963 | DPH1 | 9.2881705 | 8.0021925 | -1.285978 | 0.0011735 | 0.0084809 |
| ENSG00000134326 | CMPK2 | 7.9085603 | 6.6237366 | -1.284824 | 0.000125 | 0.0012545 |
| ENSG00000107537 | PHYH | 8.6960175 | 7.4120942 | -1.283923 | 1.30E-10 | 4.88E-09 |
| ENSG00000181026 | AEN | 7.1434084 | 5.8604541 | -1.282954 | 0.0042458 | 0.0249492 |
| ENSG00000152689 | RASGRP3 | 13.368486 | 12.085576 | -1.28291 | 5.39E-15 | 4.13E-13 |
| ENSG00000154655 | L3MBTL4 | 9.6106791 | 8.3292418 | -1.281437 | 5.22E-13 | 2.92E-11 |
| ENSG00000197165 | SULT1A2 | 5.0334462 | 3.7528027 | -1.280643 | 0.0011442 | 0.0082952 |
| ENSG00000108784 | NAGLU | 11.123313 | 9.8427174 | -1.280595 | 7.69E-08 | 1.74E-06 |
| ENSG00000179813 | FAM216B | 4.4794555 | 3.1990199 | -1.280436 | 0.0037643 | 0.0225096 |
| ENSG00000197763 | TXNRD3 | 7.2536713 | 5.9732566 | -1.280415 | 0.0003809 | 0.00326 |
| ENSG00000053918 | KCNQ1 | 9.3436766 | 8.0642397 | -1.279437 | 9.74E-13 | 5.23E-11 |
| ENSG00000051128 | HOMER3 | 11.268309 | 9.9892474 | -1.279061 | 3.96E-13 | 2.27E-11 |
| ENSG00000126016 | AMOT | 5.5182668 | 4.240306 | -1.277961 | 5.18E-06 | 7.60E-05 |
| ENSG00000117479 | SLC19A2 | 8.8524619 | 7.5751195 | -1.277342 | 0.001352 | 0.0095484 |
| ENSG00000144130 | NT5DC4 | 4.9667834 | 3.6898551 | -1.276928 | 0.0067091 | 0.0362059 |
| ENSG00000171561 | OR2AT4 | 4.9499262 | 3.6732186 | -1.276708 | 0.0037738 | 0.0225602 |
| ENSG00000076003 | MCM6 | 10.300097 | 9.0239544 | -1.276143 | 3.55E-09 | 1.03E-07 |
| ENSG00000168071 | CCDC88B | 8.4121878 | 7.1372309 | -1.274957 | 2.27E-05 | 0.0002818 |
| ENSG00000138796 | HADH | 9.5265495 | 8.25263 | -1.27392 | 6.12E-20 | 9.26E-18 |
| ENSG00000167716 | WDR81 | 12.957248 | 11.683578 | -1.27367 | 5.34E-08 | 1.25E-06 |
| ENSG00000089248 | ERP29 | 12.59277 | 11.319303 | -1.273467 | 9.23E-17 | 9.35E-15 |
| ENSG00000120049 | KCNIP2 | 6.8180958 | 5.5455573 | -1.272539 | 9.14E-07 | 1.62E-05 |
| ENSG00000175899 | A2M | 16.039168 | 14.767103 | -1.272065 | 1.59E-17 | 1.77E-15 |
| ENSG00000162804 | SNED1 | 9.007754 | 7.7360645 | -1.271689 | 4.70E-26 | 1.31E-23 |
| ENSG00000107736 | CDH23 | 11.693954 | 10.422315 | -1.271639 | 0.0002711 | 0.0024393 |
| ENSG00000167513 | CDT1 | 5.8909974 | 4.6206872 | -1.27031 | 6.50E-05 | 0.0007094 |
| ENSG00000182108 | DEXI | 10.71039 | 9.4401695 | -1.270221 | 3.23E-14 | 2.23E-12 |
| ENSG00000184903 | IMMP2L | 7.1405651 | 5.8707361 | -1.269829 | 1.21E-06 | 2.09E-05 |
| ENSG00000119514 | GALNT12 | 7.4205409 | 6.150821 | -1.26972 | 0.0066141 | 0.035786 |
| ENSG00000188060 | RAB42 | 11.920607 | 10.651474 | -1.269134 | 2.34E-05 | 0.00029 |
| ENSG00000001084 | GCLC | 12.36111 | 11.092846 | -1.268264 | 0.0005049 | 0.0041394 |
| ENSG00000204571 | KRTAP5-11 | 1.5297383 | 0.2618969 | -1.267841 | 0.0060212 | 0.0331228 |
| ENSG00000221869 | CEBPD | 10.320378 | 9.0527378 | -1.26764 | 0.0002105 | 0.0019667 |
| ENSG00000171766 | GATM | 10.065323 | 8.7994686 | -1.265854 | 3.44E-08 | 8.41E-07 |
| ENSG00000137513 | NARS2 | 8.5955794 | 7.3338684 | -1.261711 | 3.13E-06 | 4.86E-05 |
| ENSG00000132915 | PDE6A | 6.7249885 | 5.4641815 | -1.260807 | 0.0048763 | 0.0278793 |
| ENSG00000197971 | MBP | 13.190935 | 11.930242 | -1.260693 | 1.63E-10 | 6.01E-09 |
| ENSG00000120158 | RCL1 | 7.1246063 | 5.8641757 | -1.260431 | 0.0029189 | 0.0181437 |
| ENSG00000123213 | NLN | 12.566571 | 11.306431 | -1.26014 | 0.0054518 | 0.0305119 |
| ENSG00000103260 | METRN | 5.1935375 | 3.9341386 | -1.259399 | 0.000655 | 0.0051687 |
| ENSG00000196935 | SRGAP1 | 9.1024134 | 7.8434958 | -1.258918 | 9.92E-10 | 3.22E-08 |
| ENSG00000145283 | SLC10A6 | 4.1134036 | 2.8553632 | -1.25804 | 0.0046198 | 0.0266848 |
| ENSG00000163093 | BBS5 | 9.7812292 | 8.5236025 | -1.257627 | 1.83E-05 | 0.0002326 |
| ENSG00000158748 | HTR6 | 5.3944496 | 4.137385 | -1.257065 | 4.95E-06 | 7.30E-05 |
| ENSG00000137731 | FXYD2 | 7.1422797 | 5.8873092 | -1.25497 | 1.29E-05 | 0.0001714 |
| ENSG00000002726 | AOC1 | 7.3747177 | 6.1203973 | -1.25432 | 2.50E-05 | 0.0003064 |
| ENSG00000185864 | NPIPB4 | 12.062255 | 10.809504 | -1.252751 | 3.24E-16 | 3.01E-14 |
| ENSG00000100167 | SEPTIN3 | 7.0040391 | 5.7517299 | -1.252309 | 0.0002309 | 0.0021268 |
| ENSG00000105643 | ARRDC2 | 11.480465 | 10.228512 | -1.251953 | 4.92E-14 | 3.27E-12 |
| ENSG00000135446 | CDK4 | 10.262749 | 9.0109603 | -1.251789 | 5.24E-07 | 9.83E-06 |
| ENSG00000143028 | SYPL2 | 4.1222092 | 2.8710002 | -1.251209 | 5.04E-05 | 0.0005709 |
| ENSG00000152518 | ZFP36L2 | 12.507948 | 11.257093 | -1.250855 | 8.10E-15 | 6.08E-13 |
| ENSG00000100350 | FOXRED2 | 10.469411 | 9.2208641 | -1.248547 | 4.14E-08 | 9.96E-07 |
| ENSG00000173114 | LRRN3 | 6.4918513 | 5.2463381 | -1.245513 | 5.62E-06 | 8.20E-05 |
| ENSG00000267673 | FDX2 | 7.2752616 | 6.0314515 | -1.24381 | 0.0004404 | 0.0036889 |
| ENSG00000162881 | OXER1 | 7.7742162 | 6.5309503 | -1.243266 | 2.45E-05 | 0.0003018 |
| ENSG00000249131 | PSD2-AS1 | 3.4117756 | 2.1694393 | -1.242336 | 0.0040678 | 0.0240548 |
| ENSG00000196510 | ANAPC7 | 11.057798 | 9.8154949 | -1.242303 | 1.99E-13 | 1.20E-11 |
| ENSG00000100060 | MFNG | 8.9279694 | 7.6868866 | -1.241083 | 1.76E-06 | 2.93E-05 |
| ENSG00000058335 | RASGRF1 | 6.5066366 | 5.2658823 | -1.240754 | 0.0090992 | 0.0463977 |
| ENSG00000188191 | PRKAR1B | 6.8120541 | 5.5722045 | -1.23985 | 2.95E-11 | 1.24E-09 |
| ENSG00000154122 | ANKH | 12.831238 | 11.591801 | -1.239437 | 1.02E-14 | 7.63E-13 |
| ENSG00000088827 | SIGLEC1 | 14.319244 | 13.081324 | -1.237919 | 1.49E-05 | 0.0001936 |
| ENSG00000242612 | DECR2 | 7.4828882 | 6.2472609 | -1.235627 | 1.15E-06 | 2.00E-05 |
| ENSG00000140968 | IRF8 | 12.596698 | 11.361206 | -1.235492 | 6.99E-11 | 2.76E-09 |
| ENSG00000214193 | SH3D21 | 12.377118 | 11.141811 | -1.235307 | 1.56E-06 | 2.62E-05 |
| ENSG00000088367 | EPB41L1 | 9.936865 | 8.7021305 | -1.234735 | 0.0001086 | 0.0011091 |
| ENSG00000198283 | OR5B21 | 7.0656132 | 5.8314812 | -1.234132 | 0.0003549 | 0.003071 |
| ENSG00000108828 | VAT1 | 14.934709 | 13.700839 | -1.233871 | 8.07E-13 | 4.38E-11 |
| ENSG00000172878 | METAP1D | 4.3229508 | 3.0915408 | -1.23141 | 0.0051764 | 0.0292511 |
| ENSG00000206190 | ATP10A | 5.2653861 | 4.0348071 | -1.230579 | 0.0004071 | 0.0034448 |
| ENSG00000173068 | BNC2 | 11.526462 | 10.300208 | -1.226253 | 6.73E-09 | 1.88E-07 |
| ENSG00000167700 | MFSD3 | 6.3081889 | 5.082866 | -1.225323 | 0.0002306 | 0.0021243 |
| ENSG00000169291 | SHE | 5.5160604 | 4.2909366 | -1.225124 | 0.0001302 | 0.0012991 |
| ENSG00000135596 | MICAL1 | 13.47085 | 12.247406 | -1.223444 | 1.06E-08 | 2.85E-07 |
| ENSG00000135617 | PRADC1 | 8.4748803 | 7.2531336 | -1.221747 | 2.68E-06 | 4.22E-05 |
| ENSG00000180354 | MTURN | 9.6769795 | 8.4559166 | -1.221063 | 4.99E-07 | 9.42E-06 |
| ENSG00000063127 | SLC6A16 | 8.5932765 | 7.3732128 | -1.220064 | 0.0001293 | 0.0012922 |
| ENSG00000204366 | ZBTB12 | 7.4147364 | 6.1947232 | -1.220013 | 2.16E-09 | 6.58E-08 |
| ENSG00000138092 | CENPO | 9.5528508 | 8.333286 | -1.219565 | 7.27E-06 | 0.0001037 |
| ENSG00000002822 | MAD1L1 | 11.23295 | 10.013717 | -1.219233 | 3.27E-15 | 2.63E-13 |
| ENSG00000276600 | RAB7B | 9.7765295 | 8.5576552 | -1.218874 | 0.0001937 | 0.0018309 |
| ENSG00000084207 | GSTP1 | 11.852471 | 10.634323 | -1.218148 | 1.36E-06 | 2.33E-05 |
| ENSG00000111912 | NCOA7 | 10.942527 | 9.7244295 | -1.218097 | 2.41E-38 | 2.34E-35 |
| ENSG00000130748 | TMEM160 | 6.1553552 | 4.9377838 | -1.217571 | 8.29E-05 | 0.000878 |
| ENSG00000165457 | FOLR2 | 10.970705 | 9.7532188 | -1.217486 | 1.45E-08 | 3.80E-07 |
| ENSG00000073060 | SCARB1 | 13.006904 | 11.78947 | -1.217434 | 1.74E-09 | 5.38E-08 |
| ENSG00000164687 | FABP5 | 10.906528 | 9.6908005 | -1.215728 | 5.46E-07 | 1.02E-05 |
| ENSG00000163870 | TPRA1 | 10.531037 | 9.3176342 | -1.213403 | 7.05E-07 | 1.29E-05 |
| ENSG00000196218 | RYR1 | 6.2395629 | 5.0282159 | -1.211347 | 0.0004199 | 0.0035383 |
| ENSG00000170684 | ZNF296 | 6.604268 | 5.3929388 | -1.211329 | 6.38E-05 | 0.0006993 |
| ENSG00000162882 | HAAO | 9.2182817 | 8.0072441 | -1.211038 | 6.39E-05 | 0.0006996 |
| ENSG00000127083 | OMD | 7.2563743 | 6.0460232 | -1.210351 | 3.31E-05 | 0.000393 |
| ENSG00000115112 | TFCP2L1 | 10.070569 | 8.8612033 | -1.209366 | 0.0037415 | 0.0224011 |
| ENSG00000137288 | UQCC2 | 8.43507 | 7.225761 | -1.209309 | 2.03E-06 | 3.32E-05 |
| ENSG00000141994 | DUS3L | 8.9894153 | 7.7802819 | -1.209133 | 7.54E-07 | 1.37E-05 |
| ENSG00000175866 | BAIAP2 | 10.016577 | 8.8092017 | -1.207375 | 6.00E-13 | 3.33E-11 |
| ENSG00000178789 | CD300LB | 9.4291161 | 8.2220694 | -1.207047 | 5.94E-05 | 0.0006565 |
| ENSG00000103489 | XYLT1 | 10.306631 | 9.0998139 | -1.206817 | 8.84E-05 | 0.0009274 |
| ENSG00000152270 | PDE3B | 10.72131 | 9.5152812 | -1.206029 | 5.43E-06 | 7.95E-05 |
| ENSG00000104866 | PPP1R37 | 10.973053 | 9.768042 | -1.205011 | 2.61E-09 | 7.83E-08 |
| ENSG00000166965 | RCCD1 | 6.250091 | 5.045483 | -1.204608 | 1.63E-05 | 0.0002097 |
| ENSG00000139438 | FAM222A | 4.3905512 | 3.1865724 | -1.203979 | 0.0016964 | 0.0115558 |
| ENSG00000174177 | CTU2 | 7.1713108 | 5.9684417 | -1.202869 | 4.31E-06 | 6.48E-05 |
| ENSG00000184307 | ZDHHC23 | 6.3623481 | 5.1613375 | -1.201011 | 0.0003116 | 0.0027495 |
| ENSG00000137310 | TCF19 | 8.4796289 | 7.2836079 | -1.196021 | 4.89E-09 | 1.39E-07 |
| ENSG00000143126 | CELSR2 | 7.5927051 | 6.4008185 | -1.191887 | 4.20E-06 | 6.32E-05 |
| ENSG00000180998 | GPR137C | 5.2654097 | 4.0742425 | -1.191167 | 1.11E-05 | 0.0001504 |
| ENSG00000178999 | AURKB | 5.4424766 | 4.2514246 | -1.191052 | 0.0009132 | 0.0068578 |
| ENSG00000188747 | NOXA1 | 7.8768153 | 6.6882813 | -1.188534 | 4.72E-07 | 8.98E-06 |
| ENSG00000148803 | FUOM | 8.5180334 | 7.3306344 | -1.187399 | 5.02E-05 | 0.000569 |
| ENSG00000142197 | DOPEY2 | 13.334302 | 12.147455 | -1.186847 | 3.58E-08 | 8.71E-07 |
| ENSG00000173083 | HPSE | 11.834618 | 10.648888 | -1.18573 | 6.64E-07 | 1.22E-05 |
| ENSG00000064651 | SLC12A2 | 9.1796553 | 7.9960648 | -1.183591 | 4.68E-06 | 6.96E-05 |
| ENSG00000133321 | RARRES3 | 5.494949 | 4.3131485 | -1.181801 | 0.0011981 | 0.008627 |
| ENSG00000109084 | TMEM97 | 6.9734876 | 5.7940746 | -1.179413 | 0.0077764 | 0.040798 |
| ENSG00000175662 | TOM1L2 | 11.322261 | 10.146377 | -1.175884 | 3.08E-11 | 1.29E-09 |
| ENSG00000171552 | BCL2L1 | 11.10307 | 9.928182 | -1.174888 | 8.32E-06 | 0.0001168 |
| ENSG00000145703 | IQGAP2 | 14.443048 | 13.269331 | -1.173716 | 3.67E-10 | 1.29E-08 |
| ENSG00000163794 | UCN | 4.2030087 | 3.0297375 | -1.173271 | 0.0008976 | 0.0067522 |
| ENSG00000089163 | SIRT4 | 4.0542522 | 2.8841695 | -1.170083 | 0.0019422 | 0.0129298 |
| ENSG00000169972 | PUSL1 | 6.652285 | 5.4822611 | -1.170024 | 6.49E-05 | 0.0007085 |
| ENSG00000146416 | AIG1 | 9.2727246 | 8.1028556 | -1.169869 | 1.31E-09 | 4.16E-08 |
| ENSG00000186283 | TOR3A | 11.761983 | 10.592326 | -1.169658 | 0.0001842 | 0.0017529 |
| ENSG00000136111 | TBC1D4 | 10.297615 | 9.1283207 | -1.169294 | 3.08E-21 | 5.25E-19 |
| ENSG00000178175 | ZNF366 | 8.5953089 | 7.4270219 | -1.168287 | 0.0095141 | 0.0481323 |
| ENSG00000089327 | FXYD5 | 11.987807 | 10.8208 | -1.167007 | 6.02E-14 | 3.95E-12 |
| ENSG00000179057 | IGSF22 | 7.9073043 | 6.7409695 | -1.166335 | 2.32E-06 | 3.72E-05 |
| ENSG00000137809 | ITGA11 | 8.5744498 | 7.4082505 | -1.166199 | 1.90E-09 | 5.83E-08 |
| ENSG00000100099 | HPS4 | 11.997378 | 10.834573 | -1.162805 | 9.23E-08 | 2.06E-06 |
| ENSG00000134508 | CABLES1 | 9.323549 | 8.1614828 | -1.162066 | 4.81E-05 | 0.0005474 |
| ENSG00000165752 | STK32C | 9.4389784 | 8.2770472 | -1.161931 | 5.08E-08 | 1.20E-06 |
| ENSG00000112667 | DNPH1 | 7.5922095 | 6.430534 | -1.161676 | 7.99E-05 | 0.0008512 |
| ENSG00000042317 | SPATA7 | 9.5016365 | 8.3403406 | -1.161296 | 2.30E-21 | 3.97E-19 |
| ENSG00000163811 | WDR43 | 10.85488 | 9.6942215 | -1.160659 | 6.82E-06 | 9.76E-05 |
| ENSG00000204257 | HLA-DMA | 13.319131 | 12.159192 | -1.159939 | 5.18E-11 | 2.09E-09 |
| ENSG00000168495 | POLR3D | 8.2866059 | 7.1266827 | -1.159923 | 3.11E-05 | 0.0003723 |
| ENSG00000133943 | C14orf159 | 11.652467 | 10.49257 | -1.159898 | 3.21E-07 | 6.38E-06 |
| ENSG00000090013 | BLVRB | 11.245854 | 10.086283 | -1.159571 | 1.45E-09 | 4.57E-08 |
| ENSG00000133316 | WDR74 | 8.8496586 | 7.6912128 | -1.158446 | 1.53E-07 | 3.25E-06 |
| ENSG00000161179 | YDJC | 6.4202134 | 5.2622204 | -1.157993 | 2.06E-06 | 3.35E-05 |
| ENSG00000049883 | PTCD2 | 7.9292208 | 6.7713415 | -1.157879 | 0.0008225 | 0.0062666 |
| ENSG00000164855 | TMEM184A | 6.7135216 | 5.5587018 | -1.15482 | 0.0003819 | 0.0032654 |
| ENSG00000165996 | HACD1 | 6.3344295 | 5.1797493 | -1.15468 | 0.0073726 | 0.0391352 |
| ENSG00000170390 | DCLK2 | 4.8034027 | 3.6507803 | -1.152622 | 0.0019717 | 0.0130825 |
| ENSG00000109667 | SLC2A9 | 10.647408 | 9.4984085 | -1.148999 | 2.50E-09 | 7.51E-08 |
| ENSG00000162591 | MEGF6 | 8.8640757 | 7.7182616 | -1.145814 | 0.00478 | 0.0274182 |
| ENSG00000169203 | NPIPB12 | 10.808378 | 9.6630293 | -1.145348 | 7.07E-14 | 4.58E-12 |
| ENSG00000161328 | LRRC56 | 4.6210187 | 3.4760996 | -1.144919 | 0.0016646 | 0.0113869 |
| ENSG00000122778 | KIAA1549 | 5.6640461 | 4.5212075 | -1.142839 | 4.36E-06 | 6.53E-05 |
| ENSG00000102172 | SMS | 13.540298 | 12.397639 | -1.142659 | 1.05E-16 | 1.05E-14 |
| ENSG00000160396 | HIPK4 | 6.144288 | 5.0029 | -1.141388 | 8.60E-05 | 0.0009063 |
| ENSG00000186281 | GPAT2 | 6.5352339 | 5.3941748 | -1.141059 | 0.0029535 | 0.0183351 |
| ENSG00000113296 | THBS4 | 6.5710914 | 5.4318383 | -1.139253 | 0.0006261 | 0.0049635 |
| ENSG00000169740 | ZNF32 | 9.0471913 | 7.9097156 | -1.137476 | 0.008161 | 0.0424397 |
| ENSG00000165689 | SDCCAG3 | 9.8771824 | 8.740255 | -1.136927 | 3.98E-08 | 9.60E-07 |
| ENSG00000082512 | TRAF5 | 9.7878682 | 8.6516322 | -1.136236 | 3.53E-05 | 0.0004151 |
| ENSG00000142207 | URB1 | 9.1870677 | 8.0514871 | -1.135581 | 0.0051889 | 0.0293066 |
| ENSG00000164342 | TLR3 | 6.7682713 | 5.6330359 | -1.135235 | 0.001945 | 0.0129368 |
| ENSG00000205572 | SERF1B | 5.3331577 | 4.1980513 | -1.135106 | 0.0006707 | 0.0052712 |
| ENSG00000166349 | RAG1 | 6.0659687 | 4.9308655 | -1.135103 | 1.72E-05 | 0.0002201 |
| ENSG00000179889 | PDXDC1 | 13.036111 | 11.901188 | -1.134923 | 8.36E-11 | 3.23E-09 |
| ENSG00000174206 | C12orf66 | 7.7404793 | 6.6056493 | -1.13483 | 0.0001867 | 0.0017728 |
| ENSG00000025039 | RRAGD | 12.139588 | 11.006863 | -1.132725 | 7.98E-07 | 1.44E-05 |
| ENSG00000172731 | LRRC20 | 4.5535272 | 3.4208247 | -1.132702 | 0.0037941 | 0.022673 |
| ENSG00000105202 | FBL | 9.4117998 | 8.2799561 | -1.131844 | 6.78E-11 | 2.68E-09 |
| ENSG00000164929 | BAALC | 6.2659927 | 5.134717 | -1.131276 | 0.0021219 | 0.0138909 |
| ENSG00000130066 | SAT1 | 15.180228 | 14.049792 | -1.130436 | 1.13E-13 | 7.10E-12 |
| ENSG00000137496 | IL18BP | 12.182605 | 11.054218 | -1.128388 | 9.92E-06 | 0.0001359 |
| ENSG00000132361 | CLUH | 10.502742 | 9.3766877 | -1.126055 | 6.56E-06 | 9.40E-05 |
| ENSG00000135916 | ITM2C | 6.3783718 | 5.2565777 | -1.121794 | 0.0036294 | 0.0218343 |
| ENSG00000104980 | TIMM44 | 8.8439519 | 7.7235392 | -1.120413 | 7.76E-06 | 0.0001098 |
| ENSG00000128059 | PPAT | 7.7653819 | 6.646818 | -1.118564 | 7.67E-06 | 0.0001086 |
| ENSG00000132514 | CLEC10A | 8.4505366 | 7.3327359 | -1.117801 | 0.0035505 | 0.0214423 |
| ENSG00000112182 | BACH2 | 2.9105966 | 1.7943317 | -1.116265 | 0.0059241 | 0.0326705 |
| ENSG00000167701 | GPT | 5.8069479 | 4.6923971 | -1.114551 | 6.13E-05 | 0.0006746 |
| ENSG00000182199 | SHMT2 | 10.30315 | 9.1895601 | -1.11359 | 3.30E-15 | 2.65E-13 |
| ENSG00000115163 | CENPA | 3.8835438 | 2.7717931 | -1.111751 | 0.0033126 | 0.0202064 |
| ENSG00000197599 | CCDC154 | 6.5329928 | 5.4220243 | -1.110968 | 0.0014824 | 0.0103179 |
| ENSG00000106268 | NUDT1 | 8.7356732 | 7.6252677 | -1.110406 | 1.38E-11 | 6.14E-10 |
| ENSG00000030582 | GRN | 16.057433 | 14.948211 | -1.109222 | 4.72E-09 | 1.35E-07 |
| ENSG00000101096 | NFATC2 | 8.6814466 | 7.5729349 | -1.108512 | 1.02E-05 | 0.0001398 |
| ENSG00000178105 | DDX10 | 9.0965625 | 7.9880569 | -1.108506 | 0.0007793 | 0.0059859 |
| ENSG00000243716 | NPIPB5 | 11.978093 | 10.86959 | -1.108502 | 2.74E-12 | 1.36E-10 |
| ENSG00000149289 | ZC3H12C | 12.214156 | 11.1062 | -1.107956 | 0.0006428 | 0.0050839 |
| ENSG00000248487 | ABHD14A | 6.2907235 | 5.1849264 | -1.105797 | 2.79E-12 | 1.38E-10 |
| ENSG00000173621 | LRFN4 | 8.5635587 | 7.4577804 | -1.105778 | 1.66E-07 | 3.49E-06 |
| ENSG00000183049 | CAMK1D | 11.733963 | 10.628348 | -1.105615 | 4.96E-09 | 1.41E-07 |
| ENSG00000198298 | ZNF485 | 5.5347224 | 4.4326756 | -1.102047 | 0.0024002 | 0.0154096 |
| ENSG00000065057 | NTHL1 | 4.8006791 | 3.7004192 | -1.10026 | 3.76E-05 | 0.0004402 |
| ENSG00000151692 | RNF144A | 5.0547589 | 3.9553876 | -1.099371 | 1.26E-05 | 0.0001682 |
| ENSG00000180448 | ARHGAP45 | 12.063265 | 10.965536 | -1.097729 | 1.07E-10 | 4.05E-09 |
| ENSG00000107521 | HPS1 | 12.693718 | 11.596228 | -1.097491 | 8.28E-12 | 3.87E-10 |
| ENSG00000130511 | SSBP4 | 9.2953446 | 8.1990403 | -1.096304 | 9.46E-15 | 7.07E-13 |
| ENSG00000104835 | SARS2 | 7.6042535 | 6.5082896 | -1.095964 | 0.0002448 | 0.0022368 |
| ENSG00000104081 | BMF | 13.70816 | 12.612666 | -1.095494 | 1.13E-06 | 1.95E-05 |
| ENSG00000170340 | B3GNT2 | 12.279842 | 11.184406 | -1.095436 | 1.82E-08 | 4.68E-07 |
| ENSG00000163864 | NMNAT3 | 7.9194116 | 6.8250974 | -1.094314 | 1.51E-07 | 3.22E-06 |
| ENSG00000107242 | PIP5K1B | 7.7024466 | 6.6082244 | -1.094222 | 0.001098 | 0.0080148 |
| ENSG00000228594 | FNDC10 | 7.5673162 | 6.4748108 | -1.092505 | 1.43E-08 | 3.74E-07 |
| ENSG00000196743 | GM2A | 16.292142 | 15.201869 | -1.090273 | 0.0006358 | 0.005032 |
| ENSG00000214022 | REPIN1 | 11.053442 | 9.963569 | -1.089873 | 0.0016778 | 0.0114611 |
| ENSG00000147576 | ADHFE1 | 8.0114084 | 6.9221973 | -1.089211 | 1.00E-19 | 1.47E-17 |
| ENSG00000100276 | RASL10A | 6.4792652 | 5.3905054 | -1.08876 | 0.0007296 | 0.0056617 |
| ENSG00000075240 | GRAMD4 | 13.025342 | 11.936838 | -1.088503 | 3.82E-07 | 7.43E-06 |
| ENSG00000162869 | PPP1R21 | 11.733251 | 10.644988 | -1.088263 | 3.52E-26 | 1.01E-23 |
| ENSG00000156970 | BUB1B | 6.2053549 | 5.1179009 | -1.087454 | 0.001606 | 0.0110428 |
| ENSG00000159433 | STARD9 | 10.052962 | 8.96618 | -1.086782 | 6.44E-07 | 1.19E-05 |
| ENSG00000197355 | UAP1L1 | 12.211275 | 11.127632 | -1.083643 | 1.42E-07 | 3.05E-06 |
| ENSG00000178809 | TRIM73 | 6.1063478 | 5.0233602 | -1.082988 | 0.0003952 | 0.0033633 |
| ENSG00000132382 | MYBBP1A | 9.3016348 | 8.2191234 | -1.082511 | 0.000286 | 0.002557 |
| ENSG00000176049 | JAKMIP2 | 12.457874 | 11.377786 | -1.080088 | 0.0004411 | 0.0036941 |
| ENSG00000134780 | DAGLA | 10.04155 | 8.9636419 | -1.077909 | 0.0012428 | 0.0088991 |
| ENSG00000161395 | PGAP3 | 8.979299 | 7.9017766 | -1.077522 | 9.36E-05 | 0.0009744 |
| ENSG00000140488 | CELF6 | 8.9990635 | 7.9219725 | -1.077091 | 0.0001037 | 0.0010647 |
| ENSG00000125485 | DDX31 | 8.1157859 | 7.0393392 | -1.076447 | 0.0021043 | 0.0137972 |
| ENSG00000095383 | TBC1D2 | 13.250758 | 12.175013 | -1.075745 | 7.75E-05 | 0.0008283 |
| ENSG00000114786 | ABHD14A-ACY1 | 4.394839 | 3.3200254 | -1.074814 | 0.004957 | 0.0282704 |
| ENSG00000118508 | RAB32 | 11.272977 | 10.198809 | -1.074168 | 6.51E-09 | 1.82E-07 |
| ENSG00000196155 | PLEKHG4 | 5.7148361 | 4.6415113 | -1.073325 | 5.13E-05 | 0.0005792 |
| ENSG00000205903 | ZNF316 | 11.130342 | 10.057723 | -1.072619 | 9.01E-06 | 0.0001252 |
| ENSG00000133597 | ADCK2 | 10.0322 | 8.9599092 | -1.072291 | 4.91E-07 | 9.29E-06 |
| ENSG00000018699 | TTC27 | 8.1794719 | 7.1075744 | -1.071897 | 0.002041 | 0.0134487 |
| ENSG00000061455 | PRDM6 | 4.4929801 | 3.4268953 | -1.066085 | 0.0008967 | 0.006748 |
| ENSG00000155428 | TRIM74 | 5.43346 | 4.368836 | -1.064624 | 0.0013109 | 0.0093117 |
| ENSG00000197746 | PSAP | 19.521074 | 18.456496 | -1.064578 | 1.82E-08 | 4.67E-07 |
| ENSG00000089057 | SLC23A2 | 13.780019 | 12.716382 | -1.063638 | 8.44E-07 | 1.51E-05 |
| ENSG00000161929 | SCIMP | 11.772095 | 10.708958 | -1.063137 | 1.72E-07 | 3.60E-06 |
| ENSG00000171094 | ALK | 9.7487168 | 8.6859691 | -1.062748 | 0.0002182 | 0.0020237 |
| ENSG00000142185 | TRPM2 | 13.213241 | 12.151068 | -1.062173 | 5.09E-05 | 0.0005757 |
| ENSG00000152782 | PANK1 | 7.3334766 | 6.2715841 | -1.061893 | 1.29E-08 | 3.40E-07 |
| ENSG00000174804 | FZD4 | 5.4327187 | 4.3729152 | -1.059804 | 0.000359 | 0.0030996 |
| ENSG00000153395 | LPCAT1 | 11.488705 | 10.430166 | -1.058539 | 7.24E-14 | 4.67E-12 |
| ENSG00000131773 | KHDRBS3 | 5.2753778 | 4.2181223 | -1.057256 | 0.000849 | 0.0064389 |
| ENSG00000106819 | ASPN | 7.0785401 | 6.0213301 | -1.05721 | 0.0044675 | 0.0259728 |
| ENSG00000100604 | CHGA | 4.4807848 | 3.4243506 | -1.056434 | 0.0080189 | 0.0418399 |
| ENSG00000166866 | MYO1A | 6.4760199 | 5.4206608 | -1.055359 | 0.0012576 | 0.0089903 |
| ENSG00000148296 | SURF6 | 9.4885025 | 8.4341497 | -1.054353 | 5.23E-07 | 9.81E-06 |
| ENSG00000161513 | FDXR | 6.8379953 | 5.7845274 | -1.053468 | 1.01E-06 | 1.77E-05 |
| ENSG00000156381 | ANKRD9 | 8.838173 | 7.7848405 | -1.053333 | 3.21E-05 | 0.0003831 |
| ENSG00000139974 | SLC38A6 | 12.044976 | 10.992251 | -1.052725 | 8.95E-06 | 0.0001245 |
| ENSG00000167895 | TMC8 | 9.7666687 | 8.7149463 | -1.051722 | 9.85E-07 | 1.74E-05 |
| ENSG00000151240 | DIP2C | 8.4354363 | 7.3841134 | -1.051323 | 6.41E-05 | 0.0007015 |
| ENSG00000171159 | C9orf16 | 7.4880776 | 6.4369418 | -1.051136 | 3.38E-06 | 5.20E-05 |
| ENSG00000103365 | GGA2 | 13.282453 | 12.233084 | -1.049369 | 6.31E-12 | 2.99E-10 |
| ENSG00000143801 | PSEN2 | 9.9967037 | 8.9479 | -1.048804 | 1.26E-05 | 0.0001682 |
| ENSG00000197647 | ZNF433 | 8.1063846 | 7.0577093 | -1.048675 | 6.11E-10 | 2.06E-08 |
| ENSG00000164465 | DCBLD1 | 9.5891442 | 8.5415151 | -1.047629 | 1.41E-24 | 3.51E-22 |
| ENSG00000128928 | IVD | 9.7991176 | 8.7522194 | -1.046898 | 0.0005177 | 0.0042202 |
| ENSG00000141682 | PMAIP1 | 4.8060127 | 3.759301 | -1.046712 | 0.0033766 | 0.0205533 |
| ENSG00000010803 | SCMH1 | 8.2498635 | 7.2035259 | -1.046338 | 3.50E-10 | 1.24E-08 |
| ENSG00000164109 | MAD2L1 | 5.7684732 | 4.7221745 | -1.046299 | 0.0040189 | 0.0238011 |
| ENSG00000213614 | HEXA | 14.361032 | 13.315543 | -1.045489 | 4.11E-07 | 7.93E-06 |
| ENSG00000149260 | CAPN5 | 7.8796105 | 6.8346508 | -1.04496 | 0.0001721 | 0.0016508 |
| ENSG00000130159 | ECSIT | 7.8846122 | 6.8403149 | -1.044297 | 4.05E-05 | 0.0004709 |
| ENSG00000175463 | TBC1D10C | 7.6218286 | 6.5776754 | -1.044153 | 5.24E-05 | 0.0005896 |
| ENSG00000087076 | HSD17B14 | 11.196302 | 10.152872 | -1.04343 | 1.60E-07 | 3.38E-06 |
| ENSG00000169738 | DCXR | 7.9922886 | 6.9490297 | -1.043259 | 2.06E-06 | 3.36E-05 |
| ENSG00000124299 | PEPD | 11.781658 | 10.738456 | -1.043202 | 2.77E-08 | 6.89E-07 |
| ENSG00000023608 | SNAPC1 | 9.3532457 | 8.3111555 | -1.04209 | 8.53E-05 | 0.0009 |
| ENSG00000119673 | ACOT2 | 9.3783193 | 8.3363693 | -1.04195 | 5.56E-05 | 0.0006208 |
| ENSG00000144550 | CPNE9 | 6.3508911 | 5.3096182 | -1.041273 | 0.000268 | 0.0024152 |
| ENSG00000125901 | MRPS26 | 7.6388033 | 6.5975627 | -1.041241 | 4.74E-06 | 7.03E-05 |
| ENSG00000088854 | C20orf194 | 13.244403 | 12.203507 | -1.040897 | 6.93E-12 | 3.27E-10 |
| ENSG00000169100 | SLC25A6 | 12.627712 | 11.588333 | -1.039379 | 1.42E-16 | 1.39E-14 |
| ENSG00000111110 | PPM1H | 10.854 | 9.8153309 | -1.038669 | 0.0015557 | 0.0107543 |
| ENSG00000140743 | CDR2 | 9.3491865 | 8.3108865 | -1.0383 | 0.0005264 | 0.0042821 |
| ENSG00000184465 | WDR27 | 9.2745029 | 8.2362851 | -1.038218 | 2.89E-12 | 1.42E-10 |
| ENSG00000103254 | FAM173A | 5.7871239 | 4.7495271 | -1.037597 | 0.0002548 | 0.002312 |
| ENSG00000166483 | WEE1 | 6.1854122 | 5.1481472 | -1.037265 | 0.0008153 | 0.0062197 |
| ENSG00000155858 | LSM11 | 7.649738 | 6.6139276 | -1.03581 | 1.21E-14 | 8.87E-13 |
| ENSG00000145945 | FAM50B | 7.0097901 | 5.9749444 | -1.034846 | 0.0007439 | 0.0057624 |
| ENSG00000105655 | ISYNA1 | 7.1552878 | 6.1206939 | -1.034594 | 0.0066912 | 0.0361181 |
| ENSG00000198064 | NPIPB13 | 10.633938 | 9.599778 | -1.03416 | 2.78E-12 | 1.38E-10 |
| ENSG00000126787 | DLGAP5 | 5.6415454 | 4.6076882 | -1.033857 | 0.0009929 | 0.0073688 |
| ENSG00000162377 | COA7 | 9.4381007 | 8.4047253 | -1.033375 | 0.001546 | 0.0106961 |
| ENSG00000247626 | MARS2 | 7.0980021 | 6.0646914 | -1.033311 | 0.0038262 | 0.0228234 |
| ENSG00000152642 | GPD1L | 7.6923771 | 6.6597557 | -1.032621 | 0.0039449 | 0.0234359 |
| ENSG00000167074 | TEF | 10.051616 | 9.0195771 | -1.032039 | 4.64E-06 | 6.90E-05 |
| ENSG00000172197 | MBOAT1 | 9.9345954 | 8.9028755 | -1.03172 | 8.50E-05 | 0.0008974 |
| ENSG00000189227 | C15orf61 | 5.7459883 | 4.7179889 | -1.027999 | 0.001286 | 0.0091684 |
| ENSG00000125652 | ALKBH7 | 9.0375938 | 8.0095965 | -1.027997 | 6.23E-06 | 8.97E-05 |
| ENSG00000126602 | TRAP1 | 10.126107 | 9.1001045 | -1.026002 | 1.46E-06 | 2.48E-05 |
| ENSG00000184227 | ACOT1 | 7.5949587 | 6.5701052 | -1.024854 | 0.00725 | 0.0385961 |
| ENSG00000196436 | NPIPB15 | 8.5824771 | 7.5589002 | -1.023577 | 2.67E-05 | 0.0003245 |
| ENSG00000212123 | PRR22 | 4.7191362 | 3.6957214 | -1.023415 | 0.0036153 | 0.0217676 |
| ENSG00000149476 | TKFC | 9.3457437 | 8.3232993 | -1.022444 | 0.0010247 | 0.007564 |
| ENSG00000107957 | SH3PXD2A | 12.391018 | 11.37063 | -1.020388 | 1.44E-05 | 0.000188 |
| ENSG00000183828 | NUDT14 | 9.3679997 | 8.3482207 | -1.019779 | 6.24E-07 | 1.15E-05 |
| ENSG00000254206 | NPIPB11 | 9.1695714 | 8.1502696 | -1.019302 | 4.15E-12 | 2.01E-10 |
| ENSG00000090861 | AARS | 11.189845 | 10.170933 | -1.018912 | 1.48E-05 | 0.0001927 |
| ENSG00000128604 | IRF5 | 12.091904 | 11.073434 | -1.01847 | 1.13E-07 | 2.47E-06 |
| ENSG00000158716 | DUSP23 | 8.5039291 | 7.485746 | -1.018183 | 2.10E-06 | 3.41E-05 |
| ENSG00000161911 | TREML1 | 8.4114302 | 7.3941076 | -1.017323 | 7.17E-09 | 1.98E-07 |
| ENSG00000048052 | HDAC9 | 8.6301399 | 7.6131798 | -1.01696 | 0.0038701 | 0.0230572 |
| ENSG00000239474 | KLHL41 | 7.2460172 | 6.2296236 | -1.016394 | 0.0083483 | 0.0432443 |
| ENSG00000100100 | PIK3IP1 | 11.376812 | 10.360775 | -1.016037 | 0.0048007 | 0.0275119 |
| ENSG00000152475 | ZNF837 | 4.8317087 | 3.81629 | -1.015419 | 0.0018591 | 0.0124682 |
| ENSG00000147324 | MFHAS1 | 11.550829 | 10.536317 | -1.014512 | 5.81E-11 | 2.32E-09 |
| ENSG00000239264 | TXNDC5 | 11.467287 | 10.454284 | -1.013003 | 2.33E-09 | 7.07E-08 |
| ENSG00000171130 | ATP6V0E2 | 9.1879765 | 8.175881 | -1.012095 | 0.0001652 | 0.001596 |
| ENSG00000116649 | SRM | 8.38664 | 7.3751437 | -1.011496 | 0.0001782 | 0.0017027 |
| ENSG00000131584 | ACAP3 | 10.026475 | 9.0149935 | -1.011482 | 2.70E-09 | 8.07E-08 |
| ENSG00000101104 | PABPC1L | 8.1081142 | 7.0976321 | -1.010482 | 1.49E-08 | 3.90E-07 |
| ENSG00000070785 | EIF2B3 | 9.1779982 | 8.1675667 | -1.010431 | 4.26E-05 | 0.0004911 |
| ENSG00000043143 | JADE2 | 11.430584 | 10.421068 | -1.009515 | 1.99E-10 | 7.23E-09 |
| ENSG00000180902 | D2HGDH | 9.2579445 | 8.2485883 | -1.009356 | 2.54E-07 | 5.16E-06 |
| ENSG00000113552 | GNPDA1 | 12.413228 | 11.40392 | -1.009307 | 1.52E-05 | 0.0001972 |
| ENSG00000128951 | DUT | 7.5639308 | 6.5553954 | -1.008535 | 3.16E-07 | 6.30E-06 |
| ENSG00000143772 | ITPKB | 11.87359 | 10.865426 | -1.008164 | 2.81E-16 | 2.63E-14 |
| ENSG00000139193 | CD27 | 3.6896075 | 2.6831104 | -1.006497 | 0.0082429 | 0.0427796 |
| ENSG00000123933 | MXD4 | 11.131095 | 10.124841 | -1.006254 | 4.81E-08 | 1.14E-06 |
| ENSG00000178585 | CTNNBIP1 | 9.4308677 | 8.4279836 | -1.002884 | 4.20E-07 | 8.08E-06 |
| ENSG00000166682 | TMPRSS5 | 4.7131642 | 3.7103017 | -1.002862 | 0.0013173 | 0.0093483 |
| ENSG00000166387 | PPFIBP2 | 12.251281 | 11.249304 | -1.001977 | 5.02E-12 | 2.41E-10 |
| ENSG00000187024 | PTRH1 | 5.7952245 | 4.7932643 | -1.00196 | 0.0054984 | 0.0307257 |
| ENSG00000063660 | GPC1 | 5.747309 | 4.7456544 | -1.001655 | 2.70E-08 | 6.74E-07 |
| ENSG00000141873 | SLC39A3 | 8.6305916 | 7.6290075 | -1.001584 | 0.0010375 | 0.007642 |
| ENSG00000106823 | ECM2 | 7.9830798 | 6.9817424 | -1.001337 | 0.0007115 | 0.0055514 |
| ENSG00000165271 | NOL6 | 10.206057 | 9.2052136 | -1.000843 | 0.0015712 | 0.0108446 |
| ENSG00000173153 | ESRRA | 9.3619546 | 8.3611721 | -1.000782 | 0.0003322 | 0.002904 |
| ENSG00000160072 | ATAD3B | 8.3909272 | 7.3906424 | -1.000285 | 5.24E-05 | 0.0005893 |
| ENSG00000173898 | SPTBN2 | 6.7774493 | 5.7773149 | -1.000134 | 0.0001092 | 0.001114 |
| ENSG00000117411 | B4GALT2 | 6.2633254 | 5.2636849 | -0.99964 | 0.000111 | 0.0011307 |
| ENSG00000197959 | DNM3 | 8.9301396 | 7.9318565 | -0.998283 | 0.0045283 | 0.0262461 |
| ENSG00000156869 | FRRS1 | 9.2580118 | 8.2600901 | -0.997922 | 0.0017549 | 0.0118873 |
| ENSG00000163815 | CLEC3B | 5.4730715 | 4.4764312 | -0.99664 | 0.0014146 | 0.0099135 |
| ENSG00000171227 | TMEM37 | 10.554641 | 9.558578 | -0.996063 | 6.41E-05 | 0.0007013 |
| ENSG00000115756 | HPCAL1 | 11.059286 | 10.063886 | -0.9954 | 1.63E-07 | 3.44E-06 |
| ENSG00000077713 | SLC25A43 | 9.5856875 | 8.5904156 | -0.995272 | 0.0002495 | 0.0022739 |
| ENSG00000141101 | NOB1 | 8.5743602 | 7.5791622 | -0.995198 | 4.56E-05 | 0.0005214 |
| ENSG00000138363 | ATIC | 10.290137 | 9.2949764 | -0.995161 | 0.0042048 | 0.0247513 |
| ENSG00000168502 | MTCL1 | 5.3951053 | 4.4001063 | -0.994999 | 0.0096819 | 0.048832 |
| ENSG00000148908 | RGS10 | 10.505443 | 9.5108599 | -0.994583 | 2.08E-12 | 1.05E-10 |
| ENSG00000124357 | NAGK | 12.748549 | 11.754458 | -0.99409 | 1.22E-12 | 6.44E-11 |
| ENSG00000099810 | MTAP | 9.3468227 | 8.3528693 | -0.993953 | 0.0012454 | 0.0089147 |
| ENSG00000184979 | USP18 | 6.140264 | 5.14719 | -0.993074 | 0.0056269 | 0.0312917 |
| ENSG00000170779 | CDCA4 | 8.5505117 | 7.5584981 | -0.992014 | 1.95E-07 | 4.05E-06 |
| ENSG00000186451 | SPATA12 | 8.5112509 | 7.5193527 | -0.991898 | 0.0005666 | 0.0045582 |
| ENSG00000134602 | STK26 | 9.0909466 | 8.0991624 | -0.991784 | 4.12E-07 | 7.94E-06 |
| ENSG00000228300 | C19orf24 | 8.8423564 | 7.8507981 | -0.991558 | 2.94E-06 | 4.60E-05 |
| ENSG00000076351 | SLC46A1 | 11.372142 | 10.380925 | -0.991217 | 2.77E-08 | 6.89E-07 |
| ENSG00000167653 | PSCA | 5.2825488 | 4.2913805 | -0.991168 | 0.0018452 | 0.0123935 |
| ENSG00000262814 | MRPL12 | 8.5058173 | 7.5157038 | -0.990114 | 0.0031527 | 0.0193959 |
| ENSG00000140471 | LINS1 | 10.436749 | 9.4473001 | -0.989448 | 1.08E-08 | 2.88E-07 |
| ENSG00000160193 | WDR4 | 7.286649 | 6.2982407 | -0.988408 | 0.0079625 | 0.0416151 |
| ENSG00000176022 | B3GALT6 | 8.9133925 | 7.9283686 | -0.985024 | 1.68E-06 | 2.82E-05 |
| ENSG00000182240 | BACE2 | 6.6779625 | 5.6929614 | -0.985001 | 0.0053652 | 0.0300926 |
| ENSG00000029725 | RABEP1 | 12.99863 | 12.014337 | -0.984293 | 1.22E-12 | 6.44E-11 |
| ENSG00000105552 | BCAT2 | 9.2060242 | 8.2229834 | -0.983041 | 4.69E-09 | 1.34E-07 |
| ENSG00000103404 | USP31 | 9.4015089 | 8.4186416 | -0.982867 | 0.0019219 | 0.0128206 |
| ENSG00000070778 | PTPN21 | 7.5167339 | 6.533979 | -0.982755 | 8.24E-06 | 0.0001158 |
| ENSG00000116962 | NID1 | 10.158976 | 9.1767649 | -0.982211 | 0.0004336 | 0.0036404 |
| ENSG00000170271 | FAXDC2 | 9.9732212 | 8.9932326 | -0.979989 | 2.62E-06 | 4.13E-05 |
| ENSG00000157617 | C2CD2 | 9.6460608 | 8.6668915 | -0.979169 | 7.31E-09 | 2.02E-07 |
| ENSG00000146192 | FGD2 | 11.647238 | 10.668136 | -0.979102 | 2.89E-05 | 0.000348 |
| ENSG00000106070 | GRB10 | 8.951249 | 7.9721556 | -0.979093 | 6.17E-07 | 1.14E-05 |
| ENSG00000262919 | FAM58A | 7.7622089 | 6.7857373 | -0.976472 | 5.06E-05 | 0.0005728 |
| ENSG00000135362 | PRR5L | 8.7116734 | 7.7372119 | -0.974461 | 0.0042783 | 0.0250898 |
| ENSG00000115970 | THADA | 11.015439 | 10.041121 | -0.974317 | 3.97E-09 | 1.15E-07 |
| ENSG00000085465 | OVGP1 | 5.3607251 | 4.3870755 | -0.97365 | 0.003363 | 0.0204795 |
| ENSG00000167136 | ENDOG | 6.6164677 | 5.6438593 | -0.972608 | 7.79E-05 | 0.0008318 |
| ENSG00000188385 | JAKMIP3 | 5.25934 | 4.2899884 | -0.969352 | 0.0001056 | 0.001082 |
| ENSG00000165171 | METTL27 | 5.3512012 | 4.3822858 | -0.968915 | 0.0082143 | 0.0426513 |
| ENSG00000023191 | RNH1 | 11.931623 | 10.963668 | -0.967955 | 1.40E-05 | 0.0001838 |
| ENSG00000134463 | ECHDC3 | 7.4870639 | 6.519855 | -0.967209 | 0.0095896 | 0.0484306 |
| ENSG00000135094 | SDS | 12.435005 | 11.468232 | -0.966774 | 3.34E-05 | 0.0003958 |
| ENSG00000176894 | PXMP2 | 5.4875763 | 4.5210009 | -0.966575 | 0.0004701 | 0.0038956 |
| ENSG00000203772 | SPRN | 5.3572796 | 4.3915061 | -0.965774 | 0.0050901 | 0.028873 |
| ENSG00000161509 | GRIN2C | 6.6529073 | 5.6883362 | -0.964571 | 0.0001577 | 0.0015339 |
| ENSG00000196405 | EVL | 11.389296 | 10.425059 | -0.964237 | 0.0027517 | 0.0172752 |
| ENSG00000099624 | ATP5D | 8.7803479 | 7.8169991 | -0.963349 | 2.05E-06 | 3.34E-05 |
| ENSG00000070882 | OSBPL3 | 11.755005 | 10.792227 | -0.962778 | 6.70E-16 | 5.97E-14 |
| ENSG00000119139 | TJP2 | 9.5991062 | 8.6363325 | -0.962774 | 4.20E-05 | 0.0004852 |
| ENSG00000128185 | DGCR6L | 9.2826233 | 8.3203844 | -0.962239 | 1.01E-06 | 1.77E-05 |
| ENSG00000156110 | ADK | 9.7880162 | 8.8291182 | -0.958898 | 2.66E-07 | 5.38E-06 |
| ENSG00000089558 | KCNH4 | 4.6321629 | 3.6743002 | -0.957863 | 0.0011612 | 0.0084044 |
| ENSG00000163536 | SERPINI1 | 6.4338099 | 5.4761153 | -0.957695 | 0.0001268 | 0.0012717 |
| ENSG00000140750 | ARHGAP17 | 12.455873 | 11.498729 | -0.957144 | 3.95E-07 | 7.63E-06 |
| ENSG00000127415 | IDUA | 8.0357961 | 7.0790181 | -0.956778 | 1.06E-05 | 0.0001437 |
| ENSG00000170088 | TMEM192 | 10.896987 | 9.9412533 | -0.955734 | 5.91E-06 | 8.58E-05 |
| ENSG00000141447 | OSBPL1A | 12.124228 | 11.168903 | -0.955325 | 3.65E-06 | 5.58E-05 |
| ENSG00000198301 | SDAD1 | 10.826351 | 9.8725775 | -0.953773 | 1.09E-07 | 2.40E-06 |
| ENSG00000163606 | CD200R1 | 9.258619 | 8.3062477 | -0.952371 | 0.0082069 | 0.042632 |
| ENSG00000121057 | AKAP1 | 9.8178108 | 8.86751 | -0.950301 | 1.11E-07 | 2.45E-06 |
| ENSG00000187554 | TLR5 | 10.900529 | 9.9504653 | -0.950063 | 9.55E-05 | 0.0009917 |
| ENSG00000123124 | WWP1 | 13.371552 | 12.421812 | -0.949741 | 7.29E-08 | 1.66E-06 |
| ENSG00000176438 | SYNE3 | 11.421698 | 10.472814 | -0.948884 | 2.46E-05 | 0.0003023 |
| ENSG00000183605 | SFXN4 | 7.8799571 | 6.9316106 | -0.948346 | 0.0091903 | 0.0467495 |
| ENSG00000137818 | RPLP1 | 13.112175 | 12.164492 | -0.947683 | 3.85E-06 | 5.85E-05 |
| ENSG00000189283 | FHIT | 8.8218928 | 7.874428 | -0.947465 | 1.91E-07 | 3.98E-06 |
| ENSG00000185219 | ZNF445 | 10.639743 | 9.693553 | -0.94619 | 1.88E-05 | 0.0002388 |
| ENSG00000112799 | LY86 | 11.249465 | 10.303981 | -0.945484 | 1.86E-06 | 3.09E-05 |
| ENSG00000111335 | OAS2 | 10.545094 | 9.6003678 | -0.944727 | 0.0016101 | 0.0110623 |
| ENSG00000214530 | STARD10 | 8.7872155 | 7.8428957 | -0.94432 | 1.78E-10 | 6.55E-09 |
| ENSG00000090857 | PDPR | 12.174683 | 11.230643 | -0.94404 | 0.0009557 | 0.007129 |
| ENSG00000135973 | GPR45 | 6.2497105 | 5.3077452 | -0.941965 | 0.0001498 | 0.0014652 |
| ENSG00000127884 | ECHS1 | 10.318864 | 9.3791602 | -0.939704 | 1.41E-05 | 0.0001844 |
| ENSG00000156398 | SFXN2 | 8.6696561 | 7.730222 | -0.939434 | 0.0045573 | 0.0263832 |
| ENSG00000275835 | TUBGCP5 | 9.6234495 | 8.6848508 | -0.938599 | 4.22E-07 | 8.11E-06 |
| ENSG00000074219 | TEAD2 | 4.5673727 | 3.6294736 | -0.937899 | 0.0083729 | 0.0433564 |
| ENSG00000168961 | LGALS9 | 12.659318 | 11.722095 | -0.937223 | 6.70E-10 | 2.24E-08 |
| ENSG00000123545 | NDUFAF4 | 7.5233103 | 6.586107 | -0.937203 | 0.0030931 | 0.0190852 |
| ENSG00000111206 | FOXM1 | 7.6907855 | 6.754028 | -0.936758 | 2.48E-06 | 3.94E-05 |
| ENSG00000120437 | ACAT2 | 9.5549881 | 8.6190228 | -0.935965 | 0.0010828 | 0.0079236 |
| ENSG00000283297 | AC005841.2 | 5.7660161 | 4.8301231 | -0.935893 | 9.51E-05 | 0.0009884 |
| ENSG00000224877 | NDUFAF8 | 6.3955209 | 5.4604574 | -0.935063 | 0.0002167 | 0.0020126 |
| ENSG00000159374 | M1AP | 8.0473669 | 7.1131754 | -0.934192 | 0.0002198 | 0.0020361 |
| ENSG00000142102 | PGGHG | 8.8792287 | 7.9457301 | -0.933499 | 0.0001626 | 0.0015765 |
| ENSG00000162437 | RAVER2 | 7.0092973 | 6.0783128 | -0.930985 | 0.0002642 | 0.002386 |
| ENSG00000148362 | PAXX | 6.416806 | 5.4863078 | -0.930498 | 7.40E-05 | 0.0007962 |
| ENSG00000180198 | RCC1 | 9.4710208 | 8.5413327 | -0.929688 | 7.01E-09 | 1.94E-07 |
| ENSG00000198890 | PRMT6 | 8.6161456 | 7.6875277 | -0.928618 | 9.98E-08 | 2.22E-06 |
| ENSG00000156030 | ELMSAN1 | 12.421372 | 11.493042 | -0.92833 | 4.86E-14 | 3.24E-12 |
| ENSG00000113272 | THG1L | 7.541743 | 6.614369 | -0.927374 | 5.36E-05 | 0.0006013 |
| ENSG00000123064 | DDX54 | 10.549319 | 9.6224295 | -0.926889 | 1.76E-07 | 3.68E-06 |
| ENSG00000189319 | FAM53B | 11.713968 | 10.78728 | -0.926687 | 1.07E-09 | 3.46E-08 |
| ENSG00000144659 | SLC25A38 | 8.83615 | 7.9101475 | -0.926002 | 3.16E-13 | 1.84E-11 |
| ENSG00000174989 | FBXW8 | 8.9111816 | 7.985619 | -0.925563 | 0.0007753 | 0.00596 |
| ENSG00000112699 | GMDS | 7.5371596 | 6.611774 | -0.925386 | 1.97E-06 | 3.23E-05 |
| ENSG00000166166 | TRMT61A | 7.2866024 | 6.3612462 | -0.925356 | 0.001018 | 0.0075222 |
| ENSG00000072422 | RHOBTB1 | 10.194343 | 9.2693809 | -0.924962 | 2.82E-09 | 8.41E-08 |
| ENSG00000241360 | PDXP | 7.6433368 | 6.7186186 | -0.924718 | 0.0014296 | 0.0100062 |
| ENSG00000144395 | CCDC150 | 6.1107579 | 5.1861564 | -0.924601 | 1.92E-06 | 3.15E-05 |
| ENSG00000171132 | PRKCE | 8.7241298 | 7.7999399 | -0.92419 | 8.11E-07 | 1.46E-05 |
| ENSG00000164220 | F2RL2 | 9.1064911 | 8.1826988 | -0.923792 | 5.75E-07 | 1.07E-05 |
| ENSG00000107954 | NEURL1 | 6.1926947 | 5.2701605 | -0.922534 | 0.0001807 | 0.0017238 |
| ENSG00000134769 | DTNA | 8.9584516 | 8.0364333 | -0.922018 | 4.33E-06 | 6.50E-05 |
| ENSG00000167637 | ZNF283 | 7.9792623 | 7.0586011 | -0.920661 | 0.0025596 | 0.0162709 |
| ENSG00000100413 | POLR3H | 9.9652219 | 9.0447239 | -0.920498 | 2.76E-06 | 4.34E-05 |
| ENSG00000160957 | RECQL4 | 7.2596278 | 6.3393077 | -0.92032 | 1.26E-08 | 3.33E-07 |
| ENSG00000140474 | ULK3 | 9.4137358 | 8.4936493 | -0.920086 | 3.64E-07 | 7.11E-06 |
| ENSG00000108405 | P2RX1 | 8.5573326 | 7.6373515 | -0.919981 | 1.40E-05 | 0.0001833 |
| ENSG00000152767 | FARP1 | 12.309715 | 11.389864 | -0.91985 | 7.63E-07 | 1.38E-05 |
| ENSG00000137880 | GCHFR | 7.8234733 | 6.9054805 | -0.917993 | 5.19E-05 | 0.0005852 |
| ENSG00000235272 | FAM103A2P | 8.9098272 | 7.9930687 | -0.916758 | 0.0047284 | 0.0272043 |
| ENSG00000169682 | SPNS1 | 10.487982 | 9.5712378 | -0.916744 | 5.99E-07 | 1.11E-05 |
| ENSG00000181467 | RAP2B | 14.680192 | 13.764814 | -0.915378 | 1.60E-11 | 7.03E-10 |
| ENSG00000089127 | OAS1 | 10.334544 | 9.4192831 | -0.915261 | 6.27E-08 | 1.45E-06 |
| ENSG00000077348 | EXOSC5 | 7.1812842 | 6.2661047 | -0.91518 | 0.0001943 | 0.0018339 |
| ENSG00000176749 | CDK5R1 | 5.1908829 | 4.2758289 | -0.915054 | 0.0006129 | 0.004872 |
| ENSG00000005022 | SLC25A5 | 12.460071 | 11.546358 | -0.913713 | 2.88E-07 | 5.79E-06 |
| ENSG00000158813 | EDA | 7.4834327 | 6.570142 | -0.913291 | 0.0007053 | 0.0055114 |
| ENSG00000136859 | ANGPTL2 | 5.1893246 | 4.2764395 | -0.912885 | 0.0003632 | 0.0031308 |
| ENSG00000100997 | ABHD12 | 12.495786 | 11.58344 | -0.912346 | 0.0037239 | 0.0223233 |
| ENSG00000105928 | DFNA5 | 12.035272 | 11.123073 | -0.9122 | 3.72E-06 | 5.67E-05 |
| ENSG00000223865 | HLA-DPB1 | 14.058056 | 13.1469 | -0.911156 | 2.21E-05 | 0.0002764 |
| ENSG00000006740 | ARHGAP44 | 5.9035575 | 4.9925252 | -0.911032 | 3.47E-05 | 0.0004095 |
| ENSG00000111181 | SLC6A12 | 8.8079445 | 7.8977305 | -0.910214 | 0.0005841 | 0.0046819 |
| ENSG00000198429 | ZNF69 | 8.4969165 | 7.5890979 | -0.907819 | 0.0003756 | 0.0032225 |
| ENSG00000164086 | DUSP7 | 10.062817 | 9.1572566 | -0.90556 | 1.58E-07 | 3.36E-06 |
| ENSG00000104763 | ASAH1 | 15.015965 | 14.110456 | -0.905509 | 5.32E-05 | 0.0005971 |
| ENSG00000105373 | NOP53 | 10.837857 | 9.9338278 | -0.904029 | 2.24E-08 | 5.65E-07 |
| ENSG00000123992 | DNPEP | 9.7325293 | 8.8292193 | -0.90331 | 2.56E-06 | 4.05E-05 |
| ENSG00000196182 | STK40 | 12.048171 | 11.145334 | -0.902837 | 8.12E-09 | 2.22E-07 |
| ENSG00000127124 | HIVEP3 | 9.5670483 | 8.6646329 | -0.902415 | 9.04E-05 | 0.0009462 |
| ENSG00000144504 | ANKMY1 | 9.9629293 | 9.0606228 | -0.902306 | 6.33E-10 | 2.13E-08 |
| ENSG00000008853 | RHOBTB2 | 10.237359 | 9.3351866 | -0.902172 | 7.14E-07 | 1.30E-05 |
| ENSG00000007944 | MYLIP | 8.7643415 | 7.8625187 | -0.901823 | 0.0001577 | 0.0015341 |
| ENSG00000176978 | DPP7 | 11.364567 | 10.463317 | -0.90125 | 1.35E-09 | 4.27E-08 |
| ENSG00000161981 | SNRNP25 | 8.4459978 | 7.5453979 | -0.9006 | 8.71E-05 | 0.0009156 |
| ENSG00000091622 | PITPNM3 | 5.5992929 | 4.6993604 | -0.899932 | 0.000168 | 0.0016203 |
| ENSG00000181523 | SGSH | 12.017171 | 11.117413 | -0.899759 | 5.69E-05 | 0.0006334 |
| ENSG00000164930 | FZD6 | 4.9598512 | 4.0604174 | -0.899434 | 0.0046527 | 0.0268431 |
| ENSG00000178605 | GTPBP6 | 9.9389151 | 9.040468 | -0.898447 | 1.19E-07 | 2.60E-06 |
| ENSG00000259417 | CTXND1 | 5.1174598 | 4.2191767 | -0.898283 | 0.0096955 | 0.0488808 |
| ENSG00000182154 | MRPL41 | 9.1429609 | 8.2453694 | -0.897592 | 1.85E-06 | 3.06E-05 |
| ENSG00000137309 | HMGA1 | 10.661958 | 9.7643865 | -0.897571 | 5.79E-05 | 0.0006424 |
| ENSG00000100197 | CYP2D6 | 6.114589 | 5.2171353 | -0.897454 | 3.57E-05 | 0.0004204 |
| ENSG00000065268 | WDR18 | 7.8421361 | 6.9473244 | -0.894812 | 0.0003842 | 0.0032827 |
| ENSG00000198771 | RCSD1 | 11.933869 | 11.03959 | -0.894279 | 8.69E-09 | 2.37E-07 |
| ENSG00000112514 | CUTA | 9.0476528 | 8.1537926 | -0.89386 | 2.09E-07 | 4.30E-06 |
| ENSG00000153936 | HS2ST1 | 12.147825 | 11.254756 | -0.893069 | 1.85E-12 | 9.44E-11 |
| ENSG00000089486 | CDIP1 | 9.2151381 | 8.3241025 | -0.891036 | 0.0094497 | 0.0478621 |
| ENSG00000151117 | TMEM86A | 12.3799 | 11.489127 | -0.890773 | 5.14E-07 | 9.67E-06 |
| ENSG00000105963 | ADAP1 | 9.9506962 | 9.0599922 | -0.890704 | 3.83E-07 | 7.43E-06 |
| ENSG00000105676 | ARMC6 | 8.6653615 | 7.7752099 | -0.890152 | 7.55E-06 | 0.0001072 |
| ENSG00000173421 | CCDC36 | 6.7077534 | 5.817613 | -0.89014 | 0.0018511 | 0.0124235 |
| ENSG00000105677 | TMEM147 | 9.5944123 | 8.7051237 | -0.889289 | 5.68E-05 | 0.0006326 |
| ENSG00000169857 | AVEN | 7.0328354 | 6.1437042 | -0.889131 | 5.49E-06 | 8.04E-05 |
| ENSG00000182325 | FBXL6 | 6.8120935 | 5.9235393 | -0.888554 | 0.000791 | 0.0060642 |
| ENSG00000135763 | URB2 | 8.2370025 | 7.3486013 | -0.888401 | 0.0025795 | 0.0163616 |
| ENSG00000162444 | RBP7 | 4.6879003 | 3.7996111 | -0.888289 | 0.0009921 | 0.0073663 |
| ENSG00000155254 | MARVELD1 | 9.3037408 | 8.4155755 | -0.888165 | 6.07E-06 | 8.77E-05 |
| ENSG00000122971 | ACADS | 8.2388218 | 7.3507511 | -0.888071 | 0.0001543 | 0.0015055 |
| ENSG00000214309 | MBLAC1 | 4.8653411 | 3.9772791 | -0.888062 | 0.002223 | 0.0144245 |
| ENSG00000120800 | UTP20 | 9.5227526 | 8.6349994 | -0.887753 | 0.0028175 | 0.0176232 |
| ENSG00000170604 | IRF2BP1 | 8.6148 | 7.7274704 | -0.88733 | 3.03E-06 | 4.73E-05 |
| ENSG00000075702 | WDR62 | 7.7396711 | 6.8528975 | -0.886774 | 1.05E-08 | 2.82E-07 |
| ENSG00000151090 | THRB | 8.6337329 | 7.7470184 | -0.886715 | 0.0001637 | 0.0015857 |
| ENSG00000166938 | DIS3L | 10.512506 | 9.6265075 | -0.885998 | 1.17E-12 | 6.18E-11 |
| ENSG00000105609 | LILRB5 | 11.950511 | 11.06466 | -0.885851 | 0.0034584 | 0.020984 |
| ENSG00000140740 | UQCRC2 | 12.56869 | 11.684357 | -0.884334 | 3.01E-09 | 8.92E-08 |
| ENSG00000129932 | DOHH | 6.6943308 | 5.8124094 | -0.881921 | 0.0002058 | 0.0019304 |
| ENSG00000132846 | ZBED3 | 10.591239 | 9.7093763 | -0.881863 | 9.89E-06 | 0.0001357 |
| ENSG00000142544 | CTU1 | 6.3527514 | 5.4724834 | -0.880268 | 0.0014479 | 0.0101081 |
| ENSG00000184640 | SEPTIN9 | 13.016977 | 12.137144 | -0.879833 | 1.92E-05 | 0.0002429 |
| ENSG00000111666 | CHPT1 | 10.463004 | 9.5832757 | -0.879728 | 8.88E-08 | 2.00E-06 |
| ENSG00000168026 | TTC21A | 7.9517492 | 7.0724408 | -0.879308 | 5.41E-10 | 1.85E-08 |
| ENSG00000069020 | MAST4 | 7.4821722 | 6.6038264 | -0.878346 | 0.0004568 | 0.0038078 |
| ENSG00000196440 | ARMCX4 | 9.2763262 | 8.3990034 | -0.877323 | 2.35E-05 | 0.0002912 |
| ENSG00000076770 | MBNL3 | 10.917111 | 10.040027 | -0.877084 | 6.32E-10 | 2.13E-08 |
| ENSG00000169660 | HEXDC | 9.0781089 | 8.201054 | -0.877055 | 1.25E-08 | 3.31E-07 |
| ENSG00000170425 | ADORA2B | 6.7778078 | 5.9021173 | -0.87569 | 0.0081978 | 0.0426057 |
| ENSG00000180346 | TIGD2 | 8.6932954 | 7.8178299 | -0.875465 | 7.76E-05 | 0.0008288 |
| ENSG00000099284 | H2AFY2 | 8.0781842 | 7.2027851 | -0.875399 | 0.0089111 | 0.0455933 |
| ENSG00000261236 | BOP1 | 8.5539011 | 7.6788602 | -0.875041 | 0.0003781 | 0.0032389 |
| ENSG00000143858 | SYT2 | 6.2535718 | 5.379163 | -0.874409 | 0.0054752 | 0.0306114 |
| ENSG00000179041 | RRS1 | 7.1334703 | 6.2591295 | -0.874341 | 0.0003708 | 0.003187 |
| ENSG00000171714 | ANO5 | 9.3187297 | 8.4447523 | -0.873977 | 0.0008864 | 0.0066757 |
| ENSG00000130193 | THEM6 | 7.9710063 | 7.0975768 | -0.87343 | 0.0001295 | 0.0012939 |
| ENSG00000116830 | TTF2 | 9.9420297 | 9.068849 | -0.873181 | 0.0013855 | 0.0097488 |
| ENSG00000155380 | SLC16A1 | 9.2855857 | 8.4138824 | -0.871703 | 0.0009109 | 0.0068428 |
| ENSG00000119280 | C1orf198 | 7.5846375 | 6.7131529 | -0.871485 | 0.0005999 | 0.0047908 |
| ENSG00000157445 | CACNA2D3 | 7.4299552 | 6.5587331 | -0.871222 | 4.19E-07 | 8.08E-06 |
| ENSG00000129103 | SUMF2 | 11.795241 | 10.924468 | -0.870773 | 1.59E-05 | 0.0002048 |
| ENSG00000168056 | LTBP3 | 7.8314399 | 6.961615 | -0.869825 | 0.0032112 | 0.0196809 |
| ENSG00000089123 | TASP1 | 7.4026077 | 6.5329935 | -0.869614 | 8.19E-06 | 0.0001152 |
| ENSG00000136153 | LMO7 | 7.2999681 | 6.4305288 | -0.869439 | 1.28E-05 | 0.0001698 |
| ENSG00000106809 | OGN | 7.7727543 | 6.9047805 | -0.867974 | 0.0040199 | 0.0238034 |
| ENSG00000185101 | ANO9 | 6.6761851 | 5.809645 | -0.86654 | 0.0009512 | 0.007102 |
| ENSG00000171425 | ZNF581 | 7.9125911 | 7.0460719 | -0.866519 | 0.0002303 | 0.0021235 |
| ENSG00000072134 | EPN2 | 7.3669576 | 6.5036488 | -0.863309 | 0.0050396 | 0.0286491 |
| ENSG00000149201 | CCDC81 | 4.5220624 | 3.658911 | -0.863151 | 0.0019603 | 0.0130231 |
| ENSG00000179886 | TIGD5 | 7.7174802 | 6.8552768 | -0.862203 | 9.01E-06 | 0.0001252 |
| ENSG00000185163 | DDX51 | 8.2526128 | 7.3904772 | -0.862136 | 8.68E-06 | 0.0001212 |
| ENSG00000099901 | RANBP1 | 9.3446319 | 8.482707 | -0.861925 | 9.25E-06 | 0.0001279 |
| ENSG00000071054 | MAP4K4 | 12.261355 | 11.400428 | -0.860927 | 2.67E-08 | 6.67E-07 |
| ENSG00000072121 | ZFYVE26 | 14.27811 | 13.41791 | -0.8602 | 8.03E-11 | 3.12E-09 |
| ENSG00000162542 | TMCO4 | 9.5737114 | 8.7139727 | -0.859739 | 2.51E-06 | 3.99E-05 |
| ENSG00000235194 | PPP1R3E | 8.3649454 | 7.5061506 | -0.858795 | 0.0001192 | 0.0012014 |
| ENSG00000188312 | CENPP | 10.172045 | 9.3133866 | -0.858658 | 1.08E-06 | 1.89E-05 |
| ENSG00000103249 | CLCN7 | 13.27874 | 12.420579 | -0.858161 | 1.23E-07 | 2.68E-06 |
| ENSG00000153574 | RPIA | 6.8872206 | 6.0298033 | -0.857417 | 0.0025235 | 0.0160796 |
| ENSG00000119537 | KDSR | 10.571999 | 9.7149107 | -0.857088 | 9.65E-06 | 0.0001327 |
| ENSG00000103024 | NME3 | 6.503983 | 5.6472076 | -0.856775 | 0.0007146 | 0.0055693 |
| ENSG00000099795 | NDUFB7 | 9.1639238 | 8.3073182 | -0.856606 | 1.56E-05 | 0.0002019 |
| ENSG00000114779 | ABHD14B | 8.1480095 | 7.2918055 | -0.856204 | 1.27E-06 | 2.18E-05 |
| ENSG00000155189 | AGPAT5 | 9.0900349 | 8.2350159 | -0.855019 | 1.26E-05 | 0.0001681 |
| ENSG00000138095 | LRPPRC | 11.935713 | 11.082184 | -0.853529 | 4.09E-05 | 0.0004753 |
| ENSG00000117408 | IPO13 | 10.934323 | 10.082389 | -0.851934 | 1.13E-07 | 2.47E-06 |
| ENSG00000111602 | TIMELESS | 9.8298959 | 8.9781699 | -0.851726 | 4.05E-13 | 2.32E-11 |
| ENSG00000106066 | CPVL | 12.860725 | 12.009371 | -0.851354 | 9.51E-10 | 3.10E-08 |
| ENSG00000126838 | PZP | 4.9783686 | 4.1271656 | -0.851203 | 0.0050162 | 0.0285522 |
| ENSG00000188735 | TMEM120B | 8.751803 | 7.9006423 | -0.851161 | 0.0024274 | 0.0155478 |
| ENSG00000123892 | RAB38 | 7.6522707 | 6.8015433 | -0.850727 | 0.0002588 | 0.0023424 |
| ENSG00000169919 | GUSB | 11.786734 | 10.936509 | -0.850224 | 3.79E-09 | 1.10E-07 |
| ENSG00000116874 | WARS2 | 7.5855457 | 6.7353713 | -0.850174 | 0.0009151 | 0.0068693 |
| ENSG00000137752 | CASP1 | 11.43675 | 10.586721 | -0.85003 | 4.06E-11 | 1.68E-09 |
| ENSG00000078902 | TOLLIP | 11.57457 | 10.725065 | -0.849505 | 3.73E-06 | 5.68E-05 |
| ENSG00000118849 | RARRES1 | 10.353797 | 9.5048226 | -0.848975 | 0.0003216 | 0.0028237 |
| ENSG00000159674 | SPON2 | 6.7878466 | 5.9388947 | -0.848952 | 0.0002169 | 0.0020136 |
| ENSG00000087301 | TXNDC16 | 9.8379961 | 8.9890592 | -0.848937 | 0.0002159 | 0.0020062 |
| ENSG00000131844 | MCCC2 | 9.9914097 | 9.143673 | -0.847737 | 0.0043333 | 0.0253515 |
| ENSG00000167085 | PHB | 10.382365 | 9.5350743 | -0.847291 | 0.0007735 | 0.0059494 |
| ENSG00000048471 | SNX29 | 13.632288 | 12.785225 | -0.847063 | 7.40E-11 | 2.90E-09 |
| ENSG00000173272 | MZT2A | 7.3899875 | 6.5437234 | -0.846264 | 0.0008777 | 0.00663 |
| ENSG00000137822 | TUBGCP4 | 9.0100158 | 8.1645002 | -0.845516 | 0.0007578 | 0.0058491 |
| ENSG00000167972 | ABCA3 | 8.2904121 | 7.4450749 | -0.845337 | 4.75E-05 | 0.0005419 |
| ENSG00000106785 | TRIM14 | 11.766284 | 10.921042 | -0.845242 | 1.33E-15 | 1.14E-13 |
| ENSG00000266074 | BAHCC1 | 7.5373726 | 6.6927726 | -0.8446 | 0.0001365 | 0.0013541 |
| ENSG00000213199 | ASIC3 | 5.5091617 | 4.6650596 | -0.844102 | 0.0054748 | 0.0306114 |
| ENSG00000137098 | SPAG8 | 4.807845 | 3.9640989 | -0.843746 | 0.0050757 | 0.0288121 |
| ENSG00000151623 | NR3C2 | 9.1147118 | 8.2721421 | -0.84257 | 1.37E-06 | 2.34E-05 |
| ENSG00000103202 | NME4 | 9.817743 | 8.9759512 | -0.841792 | 5.78E-06 | 8.42E-05 |
| ENSG00000117461 | PIK3R3 | 9.3149944 | 8.4740988 | -0.840896 | 0.0001853 | 0.001761 |
| ENSG00000166133 | RPUSD2 | 6.7274581 | 5.8883672 | -0.839091 | 0.0002716 | 0.0024432 |
| ENSG00000118162 | KPTN | 6.2863741 | 5.4489331 | -0.837441 | 0.0003569 | 0.0030841 |
| ENSG00000095585 | BLNK | 10.90007 | 10.062642 | -0.837428 | 1.27E-07 | 2.76E-06 |
| ENSG00000156017 | CARNMT1 | 8.2592398 | 7.4223897 | -0.83685 | 0.0003753 | 0.0032205 |
| ENSG00000170382 | LRRN2 | 7.3932966 | 6.5566649 | -0.836632 | 0.0001671 | 0.0016119 |
| ENSG00000174628 | IQCK | 8.2880353 | 7.4529606 | -0.835075 | 9.08E-05 | 0.0009491 |
| ENSG00000198931 | APRT | 8.4510933 | 7.6162125 | -0.834881 | 0.0003341 | 0.0029166 |
| ENSG00000118894 | EEF2KMT | 7.3424308 | 6.5080383 | -0.834393 | 0.0045634 | 0.0264112 |
| ENSG00000008405 | CRY1 | 8.9571155 | 8.1230953 | -0.83402 | 0.0002088 | 0.0019555 |
| ENSG00000158292 | GPR153 | 6.159215 | 5.3253833 | -0.833832 | 0.0017206 | 0.0116955 |
| ENSG00000122965 | RBM19 | 10.625317 | 9.7919286 | -0.833389 | 0.0029301 | 0.0182051 |
| ENSG00000128739 | SNRPN | 10.936647 | 10.104625 | -0.832021 | 4.29E-12 | 2.07E-10 |
| ENSG00000176058 | TPRN | 7.8788255 | 7.0511424 | -0.827683 | 4.15E-05 | 0.0004803 |
| ENSG00000152556 | PFKM | 9.8682881 | 9.0418132 | -0.826475 | 1.09E-06 | 1.90E-05 |
| ENSG00000165644 | COMTD1 | 5.9667991 | 5.1405689 | -0.82623 | 0.001659 | 0.0113523 |
| ENSG00000007237 | GAS7 | 13.853782 | 13.028711 | -0.825071 | 5.42E-08 | 1.27E-06 |
| ENSG00000103066 | PLA2G15 | 10.122909 | 9.2979162 | -0.824992 | 0.0001229 | 0.0012357 |
| ENSG00000162714 | ZNF496 | 10.629108 | 9.8052127 | -0.823895 | 1.49E-05 | 0.0001936 |
| ENSG00000100605 | ITPK1 | 11.605115 | 10.781261 | -0.823854 | 2.54E-05 | 0.0003107 |
| ENSG00000160014 | CALM3 | 13.2674 | 12.443666 | -0.823734 | 0.0002248 | 0.002079 |
| ENSG00000196154 | S100A4 | 11.802521 | 10.979662 | -0.822859 | 6.57E-06 | 9.41E-05 |
| ENSG00000184831 | APOO | 8.2793409 | 7.4570003 | -0.822341 | 3.67E-05 | 0.0004305 |
| ENSG00000158717 | RNF166 | 10.423734 | 9.6016737 | -0.82206 | 1.28E-08 | 3.37E-07 |
| ENSG00000278619 | MRM1 | 6.4025254 | 5.5808311 | -0.821694 | 0.0036619 | 0.0220176 |
| ENSG00000167280 | ENGASE | 10.489658 | 9.6679828 | -0.821675 | 0.0001269 | 0.0012717 |
| ENSG00000176358 | TAC4 | 5.4942476 | 4.6727311 | -0.821516 | 1.58E-05 | 0.000204 |
| ENSG00000006025 | OSBPL7 | 8.1681512 | 7.3476747 | -0.820476 | 9.09E-07 | 1.61E-05 |
| ENSG00000128805 | ARHGAP22 | 11.153321 | 10.333185 | -0.820136 | 1.19E-06 | 2.05E-05 |
| ENSG00000151413 | NUBPL | 8.0172239 | 7.1985562 | -0.818668 | 0.0006584 | 0.0051913 |
| ENSG00000115902 | SLC1A4 | 10.81536 | 9.9967459 | -0.818614 | 8.16E-05 | 0.0008661 |
| ENSG00000197816 | CCDC180 | 7.6543938 | 6.8358986 | -0.818495 | 0.0078521 | 0.0411266 |
| ENSG00000177225 | GATD1 | 9.9225227 | 9.1040704 | -0.818452 | 0.0009986 | 0.0074054 |
| ENSG00000169598 | DFFB | 8.1358784 | 7.3174701 | -0.818408 | 0.0007685 | 0.0059193 |
| ENSG00000143367 | TUFT1 | 8.3193542 | 7.5010886 | -0.818266 | 5.27E-05 | 0.0005933 |
| ENSG00000135436 | FAM186B | 6.3989675 | 5.5816102 | -0.817357 | 0.0001526 | 0.0014915 |
| ENSG00000181619 | GPR135 | 6.7199444 | 5.9033036 | -0.816641 | 0.0055877 | 0.0311134 |
| ENSG00000175322 | ZNF519 | 8.5156348 | 7.7006567 | -0.814978 | 2.28E-06 | 3.66E-05 |
| ENSG00000187824 | TMEM220 | 7.9368513 | 7.1219173 | -0.814934 | 2.74E-05 | 0.0003322 |
| ENSG00000183889 | AC138969.1 | 9.5742959 | 8.7606262 | -0.81367 | 3.36E-06 | 5.18E-05 |
| ENSG00000185420 | SMYD3 | 7.8895142 | 7.0762988 | -0.813215 | 7.98E-07 | 1.44E-05 |
| ENSG00000134987 | WDR36 | 10.605851 | 9.7930089 | -0.812842 | 5.90E-05 | 0.0006537 |
| ENSG00000119227 | PIGZ | 6.6265809 | 5.8137777 | -0.812803 | 6.96E-05 | 0.0007539 |
| ENSG00000074071 | MRPS34 | 9.1115861 | 8.2994166 | -0.812169 | 6.17E-07 | 1.14E-05 |
| ENSG00000182810 | DDX28 | 8.6703342 | 7.8593581 | -0.810976 | 0.0001941 | 0.0018331 |
| ENSG00000214026 | MRPL23 | 8.05107 | 7.240664 | -0.810406 | 3.94E-05 | 0.0004592 |
| ENSG00000112159 | MDN1 | 12.040338 | 11.230739 | -0.8096 | 2.72E-10 | 9.76E-09 |
| ENSG00000141084 | RANBP10 | 10.967066 | 10.158029 | -0.809037 | 3.44E-05 | 0.000407 |
| ENSG00000163344 | PMVK | 8.7618457 | 7.9534127 | -0.808433 | 4.24E-05 | 0.0004887 |
| ENSG00000145375 | SPATA5 | 9.4639554 | 8.6563135 | -0.807642 | 2.63E-09 | 7.89E-08 |
| ENSG00000181163 | NPM1 | 12.789485 | 11.981871 | -0.807614 | 8.35E-09 | 2.28E-07 |
| ENSG00000102032 | RENBP | 11.31392 | 10.506406 | -0.807514 | 0.0001464 | 0.0014372 |
| ENSG00000130338 | TULP4 | 12.697282 | 11.890993 | -0.80629 | 4.22E-08 | 1.01E-06 |
| ENSG00000079308 | TNS1 | 14.445693 | 13.639815 | -0.805879 | 1.53E-06 | 2.59E-05 |
| ENSG00000162461 | SLC25A34 | 7.9101685 | 7.1044249 | -0.805744 | 0.0008168 | 0.0062291 |
| ENSG00000134905 | CARS2 | 10.290484 | 9.4848595 | -0.805625 | 1.66E-09 | 5.16E-08 |
| ENSG00000099331 | MYO9B | 14.809779 | 14.005418 | -0.80436 | 5.58E-11 | 2.24E-09 |
| ENSG00000204394 | VARS | 9.746939 | 8.942735 | -0.804204 | 0.0076435 | 0.0402496 |
| ENSG00000105698 | USF2 | 12.547663 | 11.743532 | -0.804131 | 2.17E-11 | 9.39E-10 |
| ENSG00000038945 | MSR1 | 14.883875 | 14.079875 | -0.804 | 2.48E-06 | 3.95E-05 |
| ENSG00000136158 | SPRY2 | 8.8979693 | 8.0945553 | -0.803414 | 0.0061428 | 0.0336506 |
| ENSG00000186230 | ZNF749 | 7.0905923 | 6.2873021 | -0.80329 | 0.0024059 | 0.0154328 |
| ENSG00000175564 | UCP3 | 7.4089648 | 6.605689 | -0.803276 | 1.72E-05 | 0.00022 |
| ENSG00000099290 | WASHC2A | 12.516917 | 11.713975 | -0.802942 | 1.72E-08 | 4.45E-07 |
| ENSG00000026950 | BTN3A1 | 8.8193842 | 8.016729 | -0.802655 | 4.82E-07 | 9.14E-06 |
| ENSG00000160221 | C21orf33 | 8.9002024 | 8.0988073 | -0.801395 | 0.0001013 | 0.0010432 |
| ENSG00000168010 | ATG16L2 | 12.434382 | 11.633498 | -0.800884 | 4.01E-06 | 6.07E-05 |
| ENSG00000168569 | TMEM223 | 7.9277569 | 7.1278851 | -0.799872 | 6.04E-09 | 1.70E-07 |
| ENSG00000171492 | LRRC8D | 10.585221 | 9.7866478 | -0.798573 | 0.0026897 | 0.0169571 |
| ENSG00000141441 | GAREM1 | 6.2480166 | 5.4496046 | -0.798412 | 0.0008246 | 0.0062819 |
| ENSG00000165724 | ZMYND19 | 8.1535957 | 7.3555774 | -0.798018 | 6.73E-08 | 1.55E-06 |
| ENSG00000055483 | USP36 | 11.12901 | 10.332475 | -0.796535 | 6.48E-09 | 1.81E-07 |
| ENSG00000099800 | TIMM13 | 8.7872672 | 7.9908491 | -0.796418 | 0.0003984 | 0.0033825 |
| ENSG00000162227 | TAF6L | 9.003974 | 8.2079007 | -0.796073 | 1.01E-06 | 1.78E-05 |
| ENSG00000169683 | LRRC45 | 7.7787142 | 6.9831745 | -0.79554 | 1.35E-06 | 2.30E-05 |
| ENSG00000168101 | NUDT16L1 | 8.1145284 | 7.3196773 | -0.794851 | 0.0005226 | 0.0042536 |
| ENSG00000167969 | ECI1 | 7.774334 | 6.9796733 | -0.794661 | 3.14E-05 | 0.0003746 |
| ENSG00000149527 | PLCH2 | 6.0575842 | 5.2633606 | -0.794224 | 0.0016861 | 0.0114926 |
| ENSG00000117724 | CENPF | 8.1093429 | 7.315186 | -0.794157 | 0.0004643 | 0.0038574 |
| ENSG00000105447 | GRWD1 | 8.5928135 | 7.7987959 | -0.794018 | 0.0030685 | 0.0189552 |
| ENSG00000119943 | PYROXD2 | 7.3943929 | 6.6019442 | -0.792449 | 0.0006976 | 0.0054597 |
| ENSG00000167468 | GPX4 | 12.562338 | 11.770653 | -0.791685 | 1.15E-05 | 0.000155 |
| ENSG00000033011 | ALG1 | 9.3534677 | 8.5629165 | -0.790551 | 0.0003366 | 0.0029349 |
| ENSG00000186106 | ANKRD46 | 8.1137073 | 7.3236846 | -0.790023 | 0.0003124 | 0.0027547 |
| ENSG00000027001 | MIPEP | 8.7820414 | 7.9928503 | -0.789191 | 1.06E-05 | 0.0001442 |
| ENSG00000154027 | AK5 | 5.5406668 | 4.7524373 | -0.788229 | 0.0039005 | 0.0232103 |
| ENSG00000197785 | ATAD3A | 8.1776782 | 7.3903428 | -0.787335 | 0.0002026 | 0.0019044 |
| ENSG00000088836 | SLC4A11 | 8.4298677 | 7.6428877 | -0.78698 | 6.20E-05 | 0.0006819 |
| ENSG00000106123 | EPHB6 | 8.6217418 | 7.8355058 | -0.786236 | 1.37E-10 | 5.12E-09 |
| ENSG00000126088 | UROD | 10.313843 | 9.5292238 | -0.784619 | 7.88E-07 | 1.43E-05 |
| ENSG00000177700 | POLR2L | 8.9879766 | 8.2035084 | -0.784468 | 1.84E-05 | 0.0002339 |
| ENSG00000138621 | PPCDC | 8.6711372 | 7.8867578 | -0.784379 | 0.0003093 | 0.0027324 |
| ENSG00000135722 | FBXL8 | 6.4461652 | 5.6624344 | -0.783731 | 0.0078313 | 0.0410426 |
| ENSG00000099904 | ZDHHC8 | 9.8212218 | 9.0384946 | -0.782727 | 4.96E-09 | 1.41E-07 |
| ENSG00000105227 | PRX | 7.6172109 | 6.8346293 | -0.782582 | 0.000195 | 0.0018397 |
| ENSG00000081760 | AACS | 8.8274565 | 8.044915 | -0.782541 | 6.02E-05 | 0.0006641 |
| ENSG00000215012 | RTL10 | 10.08936 | 9.3072556 | -0.782105 | 0.0002704 | 0.0024337 |
| ENSG00000143499 | SMYD2 | 9.5752273 | 8.7931444 | -0.782083 | 2.24E-14 | 1.58E-12 |
| ENSG00000099899 | TRMT2A | 9.6140611 | 8.832171 | -0.78189 | 6.17E-06 | 8.91E-05 |
| ENSG00000126107 | HECTD3 | 11.766132 | 10.984663 | -0.781469 | 8.45E-12 | 3.95E-10 |
| ENSG00000112118 | MCM3 | 9.9545833 | 9.1737391 | -0.780844 | 1.95E-07 | 4.04E-06 |
| ENSG00000180332 | KCTD4 | 7.2406679 | 6.4600947 | -0.780573 | 0.0003463 | 0.003008 |
| ENSG00000185442 | FAM174B | 7.9729208 | 7.1928556 | -0.780065 | 0.0023287 | 0.0150146 |
| ENSG00000065150 | IPO5 | 10.824578 | 10.04499 | -0.779588 | 2.42E-06 | 3.85E-05 |
| ENSG00000132004 | FBXW9 | 5.4322205 | 4.6528546 | -0.779366 | 0.0047519 | 0.027292 |
| ENSG00000182173 | TSEN54 | 7.1184192 | 6.3392791 | -0.77914 | 0.0019417 | 0.0129286 |
| ENSG00000196422 | PPP1R26 | 10.092037 | 9.3131773 | -0.778859 | 8.19E-11 | 3.18E-09 |
| ENSG00000182871 | COL18A1 | 6.4531308 | 5.6747197 | -0.778411 | 0.0027131 | 0.017075 |
| ENSG00000066583 | ISOC1 | 8.4797485 | 7.7015217 | -0.778227 | 0.0007187 | 0.0055941 |
| ENSG00000160712 | IL6R | 12.350831 | 11.573207 | -0.777624 | 8.07E-11 | 3.13E-09 |
| ENSG00000103550 | KNOP1 | 10.169219 | 9.3917675 | -0.777452 | 1.16E-06 | 2.01E-05 |
| ENSG00000185418 | TARSL2 | 9.0315542 | 8.2541917 | -0.777362 | 3.95E-05 | 0.0004597 |
| ENSG00000166348 | USP54 | 10.553015 | 9.7760434 | -0.776972 | 3.27E-07 | 6.48E-06 |
| ENSG00000151729 | SLC25A4 | 6.1355565 | 5.3587639 | -0.776793 | 0.0007917 | 0.0060682 |
| ENSG00000110455 | ACCS | 10.585651 | 9.8096921 | -0.775959 | 0.0032637 | 0.0199633 |
| ENSG00000185324 | CDK10 | 9.3082772 | 8.533506 | -0.774771 | 5.10E-09 | 1.44E-07 |
| ENSG00000159496 | RGL4 | 6.9618978 | 6.1874242 | -0.774474 | 2.86E-09 | 8.51E-08 |
| ENSG00000198246 | SLC29A3 | 10.612384 | 9.8380827 | -0.774301 | 0.0067846 | 0.0365269 |
| ENSG00000127903 | ZNF835 | 5.9364133 | 5.1625353 | -0.773878 | 0.0036756 | 0.0220881 |
| ENSG00000163126 | ANKRD23 | 5.5583628 | 4.7852355 | -0.773127 | 0.0027711 | 0.0173676 |
| ENSG00000143486 | EIF2D | 10.072814 | 9.2997672 | -0.773046 | 2.96E-09 | 8.78E-08 |
| ENSG00000119185 | ITGB1BP1 | 9.6721447 | 8.8991258 | -0.773019 | 0.0015395 | 0.0106625 |
| ENSG00000122378 | FAM213A | 11.350947 | 10.578296 | -0.772651 | 2.55E-08 | 6.39E-07 |
| ENSG00000164808 | SPIDR | 11.12273 | 10.350358 | -0.772372 | 8.51E-13 | 4.60E-11 |
| ENSG00000249471 | ZNF324B | 7.5217949 | 6.7495198 | -0.772275 | 0.0011052 | 0.0080603 |
| ENSG00000121577 | POPDC2 | 5.0398908 | 4.2680179 | -0.771873 | 0.0047007 | 0.0270807 |
| ENSG00000063046 | EIF4B | 13.864404 | 13.095171 | -0.769233 | 1.35E-05 | 0.0001785 |
| ENSG00000197448 | GSTK1 | 11.456752 | 10.68788 | -0.768872 | 3.06E-12 | 1.50E-10 |
| ENSG00000129480 | DTD2 | 6.8814843 | 6.1130898 | -0.768395 | 0.0065474 | 0.0354909 |
| ENSG00000055732 | MCOLN3 | 9.4721062 | 8.7045675 | -0.767539 | 0.0019839 | 0.0131352 |
| ENSG00000053702 | NRIP2 | 8.6079376 | 7.8404399 | -0.767498 | 2.34E-08 | 5.89E-07 |
| ENSG00000153107 | ANAPC1 | 10.464556 | 9.6970882 | -0.767468 | 4.36E-05 | 0.0005014 |
| ENSG00000140836 | ZFHX3 | 13.141365 | 12.374103 | -0.767262 | 5.38E-08 | 1.26E-06 |
| ENSG00000166394 | CYB5R2 | 8.4146854 | 7.6479009 | -0.766784 | 0.000488 | 0.0040218 |
| ENSG00000127334 | DYRK2 | 11.719722 | 10.953277 | -0.766445 | 2.19E-07 | 4.49E-06 |
| ENSG00000171817 | ZNF540 | 6.6511517 | 5.884748 | -0.766404 | 0.000103 | 0.0010576 |
| ENSG00000122376 | FAM35A | 10.764271 | 9.9983784 | -0.765892 | 5.59E-06 | 8.16E-05 |
| ENSG00000119772 | DNMT3A | 11.729427 | 10.963586 | -0.765841 | 2.98E-09 | 8.85E-08 |
| ENSG00000183426 | NPIPA1 | 9.6297964 | 8.864415 | -0.765381 | 1.70E-10 | 6.27E-09 |
| ENSG00000178425 | NT5DC1 | 10.290894 | 9.5272655 | -0.763628 | 6.09E-06 | 8.79E-05 |
| ENSG00000244187 | TMEM141 | 8.4273815 | 7.6645737 | -0.762808 | 4.93E-07 | 9.33E-06 |
| ENSG00000125877 | ITPA | 8.7319895 | 7.9697788 | -0.762211 | 0.0008774 | 0.0066295 |
| ENSG00000108961 | RANGRF | 7.914677 | 7.1536127 | -0.761064 | 2.25E-05 | 0.0002803 |
| ENSG00000181396 | OGFOD3 | 8.944985 | 8.1842861 | -0.760699 | 1.34E-06 | 2.29E-05 |
| ENSG00000088727 | KIF9 | 5.8318922 | 5.0712793 | -0.760613 | 0.0008842 | 0.0066631 |
| ENSG00000172037 | LAMB2 | 11.848366 | 11.088982 | -0.759384 | 3.67E-08 | 8.89E-07 |
| ENSG00000165475 | CRYL1 | 11.734092 | 10.97522 | -0.758872 | 0.0001645 | 0.0015909 |
| ENSG00000146576 | C7orf26 | 9.0936224 | 8.3350598 | -0.758563 | 3.48E-05 | 0.000411 |
| ENSG00000223496 | EXOSC6 | 9.193214 | 8.4349997 | -0.758214 | 0.0017863 | 0.0120539 |
| ENSG00000136104 | RNASEH2B | 11.179026 | 10.421641 | -0.757385 | 3.85E-07 | 7.47E-06 |
| ENSG00000198722 | UNC13B | 11.366787 | 10.610487 | -0.7563 | 2.91E-12 | 1.43E-10 |
| ENSG00000007376 | RPUSD1 | 8.3004355 | 7.5447271 | -0.755708 | 0.0001106 | 0.0011272 |
| ENSG00000090581 | GNPTG | 11.572731 | 10.817119 | -0.755613 | 2.18E-06 | 3.53E-05 |
| ENSG00000170190 | SLC16A5 | 9.0011264 | 8.24554 | -0.755586 | 0.0001392 | 0.0013757 |
| ENSG00000186532 | SMYD4 | 10.631395 | 9.8761571 | -0.755237 | 1.36E-14 | 9.85E-13 |
| ENSG00000125967 | NECAB3 | 7.2185799 | 6.4634608 | -0.755119 | 9.42E-06 | 0.0001299 |
| ENSG00000108679 | LGALS3BP | 10.033846 | 9.2790906 | -0.754756 | 0.0013108 | 0.0093117 |
| ENSG00000167526 | RPL13 | 11.95253 | 11.198025 | -0.754505 | 3.19E-11 | 1.34E-09 |
| ENSG00000211584 | SLC48A1 | 11.025071 | 10.270852 | -0.754219 | 2.81E-07 | 5.66E-06 |
| ENSG00000183943 | PRKX | 11.768604 | 11.014709 | -0.753896 | 0.0003719 | 0.0031954 |
| ENSG00000126790 | L3HYPDH | 7.5419961 | 6.788413 | -0.753583 | 0.0014861 | 0.0103422 |
| ENSG00000138442 | WDR12 | 9.6194827 | 8.8659389 | -0.753544 | 0.0023743 | 0.0152656 |
| ENSG00000198517 | MAFK | 9.5816133 | 8.8283817 | -0.753232 | 5.36E-07 | 1.00E-05 |
| ENSG00000163382 | NAXE | 9.3541992 | 8.6014029 | -0.752796 | 1.15E-06 | 1.99E-05 |
| ENSG00000145476 | CYP4V2 | 11.74422 | 10.991438 | -0.752782 | 0.0003065 | 0.0027128 |
| ENSG00000102547 | CAB39L | 10.171942 | 9.4199428 | -0.751999 | 4.19E-05 | 0.0004838 |
| ENSG00000167281 | RBFOX3 | 5.1987363 | 4.4469359 | -0.7518 | 0.0006362 | 0.0050335 |
| ENSG00000110080 | ST3GAL4 | 8.1342689 | 7.3838137 | -0.750455 | 0.0034985 | 0.0211836 |
| ENSG00000183779 | ZNF703 | 8.8397329 | 8.0896928 | -0.75004 | 0.0001216 | 0.0012242 |
| ENSG00000174306 | ZHX3 | 10.616321 | 9.8683221 | -0.747999 | 2.57E-05 | 0.0003142 |
| ENSG00000006704 | GTF2IRD1 | 9.4045703 | 8.6570489 | -0.747521 | 0.0001042 | 0.0010689 |
| ENSG00000141569 | TRIM65 | 10.056055 | 9.3087951 | -0.74726 | 8.68E-05 | 0.0009135 |
| ENSG00000248712 | CCDC153 | 5.787355 | 5.0404614 | -0.746894 | 0.0038955 | 0.0231868 |
| ENSG00000100324 | TAB1 | 10.587164 | 9.8402966 | -0.746867 | 4.64E-09 | 1.33E-07 |
| ENSG00000172465 | TCEAL1 | 8.6551076 | 7.9083796 | -0.746728 | 0.0002144 | 0.001994 |
| ENSG00000163517 | HDAC11 | 7.9546609 | 7.2083199 | -0.746341 | 2.08E-05 | 0.0002607 |
| ENSG00000079819 | EPB41L2 | 11.581202 | 10.835288 | -0.745915 | 1.53E-12 | 7.92E-11 |
| ENSG00000183527 | PSMG1 | 8.5565997 | 7.8109597 | -0.74564 | 0.0035846 | 0.0216034 |
| ENSG00000240038 | AMY2B | 9.4101882 | 8.6649167 | -0.745272 | 8.51E-07 | 1.52E-05 |
| ENSG00000075884 | ARHGAP15 | 10.767284 | 10.022527 | -0.744757 | 1.31E-19 | 1.90E-17 |
| ENSG00000187650 | VMAC | 8.4710279 | 7.7267521 | -0.744276 | 3.22E-06 | 5.00E-05 |
| ENSG00000102572 | STK24 | 12.411824 | 11.667949 | -0.743875 | 3.71E-09 | 1.08E-07 |
| ENSG00000100365 | NCF4 | 11.915521 | 11.17451 | -0.741011 | 3.15E-08 | 7.73E-07 |
| ENSG00000169718 | DUS1L | 9.8275262 | 9.0868699 | -0.740656 | 9.85E-09 | 2.66E-07 |
| ENSG00000167702 | KIFC2 | 8.4062949 | 7.6657397 | -0.740555 | 8.24E-05 | 0.0008739 |
| ENSG00000144827 | ABHD10 | 9.4281401 | 8.6889789 | -0.739161 | 0.0040969 | 0.0242073 |
| ENSG00000165983 | PTER | 9.6613573 | 8.9230345 | -0.738323 | 2.50E-06 | 3.96E-05 |
| ENSG00000163975 | MELTF | 8.4211531 | 7.6830056 | -0.738147 | 7.63E-05 | 0.0008173 |
| ENSG00000144741 | SLC25A26 | 8.414817 | 7.6768486 | -0.737968 | 0.0010913 | 0.0079749 |
| ENSG00000176476 | SGF29 | 8.7952318 | 8.0575562 | -0.737676 | 7.68E-08 | 1.74E-06 |
| ENSG00000167302 | TEPSIN | 9.6063072 | 8.8687792 | -0.737528 | 2.98E-07 | 5.98E-06 |
| ENSG00000185813 | PCYT2 | 8.2617529 | 7.5248818 | -0.736871 | 0.0071081 | 0.0379893 |
| ENSG00000021762 | OSBPL5 | 9.8184911 | 9.0823248 | -0.736166 | 1.28E-05 | 0.00017 |
| ENSG00000230124 | ACBD6 | 9.4755382 | 8.7401847 | -0.735353 | 5.04E-07 | 9.50E-06 |
| ENSG00000145349 | CAMK2D | 9.7745789 | 9.039422 | -0.735157 | 0.0001803 | 0.0017208 |
| ENSG00000100994 | PYGB | 11.959194 | 11.224217 | -0.734977 | 4.83E-06 | 7.15E-05 |
| ENSG00000093072 | ADA2 | 15.770715 | 15.036114 | -0.734601 | 1.05E-08 | 2.83E-07 |
| ENSG00000204152 | TIMM23B | 7.871661 | 7.1375054 | -0.734156 | 0.0001238 | 0.0012432 |
| ENSG00000137274 | BPHL | 7.7445619 | 7.0112515 | -0.73331 | 1.18E-06 | 2.03E-05 |
| ENSG00000087086 | FTL | 18.732794 | 17.999855 | -0.732938 | 1.11E-05 | 0.00015 |
| ENSG00000095002 | MSH2 | 8.5207668 | 7.7887843 | -0.731983 | 0.0025029 | 0.0159731 |
| ENSG00000126432 | PRDX5 | 10.357424 | 9.6254529 | -0.731971 | 1.53E-11 | 6.76E-10 |
| ENSG00000130826 | DKC1 | 9.6984056 | 8.9664807 | -0.731925 | 3.93E-05 | 0.0004575 |
| ENSG00000141295 | SCRN2 | 7.9986283 | 7.26682 | -0.731808 | 1.03E-06 | 1.81E-05 |
| ENSG00000169413 | RNASE6 | 11.571992 | 10.841327 | -0.730665 | 6.00E-05 | 0.0006622 |
| ENSG00000154760 | SLFN13 | 7.4417937 | 6.7115091 | -0.730285 | 1.79E-07 | 3.75E-06 |
| ENSG00000179364 | PACS2 | 11.478 | 10.747741 | -0.730259 | 9.00E-10 | 2.94E-08 |
| ENSG00000104731 | KLHDC4 | 9.1984822 | 8.4688665 | -0.729616 | 3.89E-06 | 5.90E-05 |
| ENSG00000083093 | PALB2 | 9.763823 | 9.0356024 | -0.728221 | 2.26E-05 | 0.000281 |
| ENSG00000112619 | PRPH2 | 5.6577217 | 4.9298558 | -0.727866 | 0.0024345 | 0.015584 |
| ENSG00000215440 | NPEPL1 | 10.528301 | 9.8005024 | -0.727799 | 4.89E-06 | 7.24E-05 |
| ENSG00000131446 | MGAT1 | 14.159572 | 13.432588 | -0.726984 | 7.18E-05 | 0.0007753 |
| ENSG00000196391 | ZNF774 | 7.4756438 | 6.7493966 | -0.726247 | 0.0005221 | 0.0042512 |
| ENSG00000105851 | PIK3CG | 12.009372 | 11.283433 | -0.725939 | 1.50E-09 | 4.70E-08 |
| ENSG00000140691 | ARMC5 | 7.6588082 | 6.9330447 | -0.725763 | 0.0017951 | 0.0121002 |
| ENSG00000137133 | HINT2 | 7.1556271 | 6.429866 | -0.725761 | 2.82E-09 | 8.40E-08 |
| ENSG00000112293 | GPLD1 | 5.8544185 | 5.1294082 | -0.72501 | 0.0064205 | 0.034911 |
| ENSG00000162909 | CAPN2 | 11.150492 | 10.426546 | -0.723946 | 0.0002119 | 0.0019744 |
| ENSG00000137054 | POLR1E | 7.4464186 | 6.7232591 | -0.72316 | 0.0098734 | 0.0495438 |
| ENSG00000085871 | MGST2 | 9.5909836 | 8.867864 | -0.72312 | 5.54E-05 | 0.0006191 |
| ENSG00000140398 | NEIL1 | 8.2767736 | 7.5537058 | -0.723068 | 0.0010829 | 0.0079236 |
| ENSG00000179918 | SEPHS2 | 12.449201 | 11.726455 | -0.722745 | 0.0023238 | 0.0149872 |
| ENSG00000213551 | DNAJC9 | 8.9425774 | 8.2213849 | -0.721193 | 5.34E-13 | 2.98E-11 |
| ENSG00000106605 | BLVRA | 10.759105 | 10.037987 | -0.721118 | 1.63E-07 | 3.44E-06 |
| ENSG00000165526 | RPUSD4 | 8.452723 | 7.7319934 | -0.72073 | 0.0004386 | 0.0036769 |
| ENSG00000175197 | DDIT3 | 8.3782478 | 7.6575763 | -0.720671 | 0.0002449 | 0.0022375 |
| ENSG00000079215 | SLC1A3 | 12.580025 | 11.859601 | -0.720424 | 0.0069165 | 0.0371556 |
| ENSG00000126953 | TIMM8A | 7.0434242 | 6.3243 | -0.719124 | 0.0020961 | 0.0137521 |
| ENSG00000004975 | DVL2 | 10.113379 | 9.3943491 | -0.71903 | 1.70E-09 | 5.25E-08 |
| ENSG00000171970 | ZNF57 | 5.8122638 | 5.0938366 | -0.718427 | 0.0098099 | 0.049291 |
| ENSG00000134278 | SPIRE1 | 12.384648 | 11.668388 | -0.716261 | 3.94E-07 | 7.62E-06 |
| ENSG00000119285 | HEATR1 | 10.178577 | 9.4637992 | -0.714778 | 0.0012876 | 0.0091784 |
| ENSG00000131242 | RAB11FIP4 | 8.1720471 | 7.4574402 | -0.714607 | 0.0098107 | 0.049291 |
| ENSG00000154743 | TSEN2 | 7.6944564 | 6.9799919 | -0.714464 | 0.0083243 | 0.0431405 |
| ENSG00000182179 | UBA7 | 10.935621 | 10.221757 | -0.713864 | 1.30E-07 | 2.82E-06 |
| ENSG00000136783 | NIPSNAP3A | 8.8058236 | 8.0925755 | -0.713248 | 4.32E-08 | 1.03E-06 |
| ENSG00000136856 | SLC2A8 | 9.1162066 | 8.4044244 | -0.711782 | 0.0003653 | 0.0031477 |
| ENSG00000204160 | ZDHHC18 | 10.312276 | 9.6006189 | -0.711657 | 0.0013 | 0.0092406 |
| ENSG00000073910 | FRY | 9.7526114 | 9.0412449 | -0.711366 | 0.0057011 | 0.0316284 |
| ENSG00000136720 | HS6ST1 | 10.936842 | 10.22558 | -0.711262 | 8.69E-09 | 2.37E-07 |
| ENSG00000125257 | ABCC4 | 9.7470249 | 9.0359904 | -0.711035 | 0.0003946 | 0.0033594 |
| ENSG00000101361 | NOP56 | 9.9921609 | 9.2825982 | -0.709563 | 4.79E-05 | 0.0005456 |
| ENSG00000125703 | ATG4C | 10.490397 | 9.7811029 | -0.709294 | 1.44E-07 | 3.08E-06 |
| ENSG00000161999 | JMJD8 | 9.2403257 | 8.5311678 | -0.709158 | 7.25E-05 | 0.0007814 |
| ENSG00000177599 | ZNF491 | 6.3617346 | 5.6534692 | -0.708265 | 0.0004767 | 0.0039427 |
| ENSG00000111716 | LDHB | 11.777912 | 11.070461 | -0.707451 | 7.72E-06 | 0.0001091 |
| ENSG00000063177 | RPL18 | 11.994721 | 11.288491 | -0.70623 | 3.53E-07 | 6.91E-06 |
| ENSG00000117691 | NENF | 9.568187 | 8.8624468 | -0.70574 | 8.89E-05 | 0.0009324 |
| ENSG00000153179 | RASSF3 | 12.625045 | 11.920244 | -0.704801 | 3.10E-06 | 4.83E-05 |
| ENSG00000105671 | DDX49 | 9.0462324 | 8.3418437 | -0.704389 | 3.32E-06 | 5.13E-05 |
| ENSG00000216490 | IFI30 | 16.800413 | 16.096269 | -0.704145 | 0.000125 | 0.0012545 |
| ENSG00000140365 | COMMD4 | 8.7842618 | 8.0802773 | -0.703984 | 0.000434 | 0.0036427 |
| ENSG00000170275 | CRTAP | 12.973098 | 12.26952 | -0.703579 | 4.22E-10 | 1.47E-08 |
| ENSG00000198816 | ZNF358 | 9.7248377 | 9.0215835 | -0.703254 | 0.0080054 | 0.0417896 |
| ENSG00000171608 | PIK3CD | 12.291499 | 11.589519 | -0.70198 | 5.50E-07 | 1.03E-05 |
| ENSG00000105854 | PON2 | 8.8771344 | 8.1759593 | -0.701175 | 8.53E-06 | 0.0001197 |
| ENSG00000152082 | MZT2B | 7.5781214 | 6.8773295 | -0.700792 | 0.0006788 | 0.005327 |
| ENSG00000116251 | RPL22 | 11.370143 | 10.671118 | -0.699024 | 5.93E-06 | 8.60E-05 |
| ENSG00000106344 | RBM28 | 10.743988 | 10.045001 | -0.698987 | 0.002752 | 0.0172752 |
| ENSG00000167515 | TRAPPC2L | 9.1446945 | 8.4457809 | -0.698914 | 2.67E-05 | 0.0003243 |
| ENSG00000100401 | RANGAP1 | 10.694504 | 9.9957094 | -0.698795 | 0.0001428 | 0.0014072 |
| ENSG00000188321 | ZNF559 | 8.3556475 | 7.6578473 | -0.6978 | 0.0047154 | 0.0271507 |
| ENSG00000172661 | WASHC2C | 12.67302 | 11.975549 | -0.697471 | 6.82E-08 | 1.57E-06 |
| ENSG00000163219 | ARHGAP25 | 12.450964 | 11.753656 | -0.697308 | 2.58E-07 | 5.24E-06 |
| ENSG00000185187 | SIGIRR | 7.7104973 | 7.0133778 | -0.69712 | 0.0012192 | 0.0087573 |
| ENSG00000105607 | GCDH | 8.0830288 | 7.3861938 | -0.696835 | 0.0009761 | 0.0072642 |
| ENSG00000178226 | PRSS36 | 9.0253698 | 8.3286971 | -0.696673 | 0.0004217 | 0.0035519 |
| ENSG00000162825 | NBPF20 | 8.4362344 | 7.7405039 | -0.695731 | 1.35E-05 | 0.0001786 |
| ENSG00000163528 | CHCHD4 | 7.8098446 | 7.114236 | -0.695609 | 0.0009992 | 0.0074085 |
| ENSG00000159714 | ZDHHC1 | 5.6822408 | 4.987722 | -0.694519 | 0.0064266 | 0.0349355 |
| ENSG00000077684 | JADE1 | 9.0066568 | 8.312608 | -0.694049 | 0.0009189 | 0.006895 |
| ENSG00000135951 | TSGA10 | 8.9889975 | 8.2955442 | -0.693453 | 0.0010155 | 0.0075098 |
| ENSG00000083635 | NUFIP1 | 7.4878546 | 6.7946084 | -0.693246 | 0.0030532 | 0.0188739 |
| ENSG00000167658 | EEF2 | 14.7385 | 14.045311 | -0.693189 | 1.96E-10 | 7.14E-09 |
| ENSG00000152620 | NADK2 | 9.4026492 | 8.7104258 | -0.692223 | 0.0002991 | 0.0026564 |
| ENSG00000068383 | INPP5A | 8.6839544 | 7.9918729 | -0.692082 | 9.83E-05 | 0.0010166 |
| ENSG00000131368 | MRPS25 | 9.895613 | 9.2037452 | -0.691868 | 0.0008602 | 0.0065112 |
| ENSG00000174738 | NR1D2 | 11.724955 | 11.033488 | -0.691467 | 0.0004438 | 0.0037105 |
| ENSG00000058453 | CROCC | 8.7960108 | 8.1050367 | -0.690974 | 0.0025311 | 0.0161173 |
| ENSG00000103126 | AXIN1 | 10.259708 | 9.5688623 | -0.690846 | 6.34E-06 | 9.12E-05 |
| ENSG00000184162 | NR2C2AP | 6.5813267 | 5.8917848 | -0.689542 | 0.0002721 | 0.0024471 |
| ENSG00000122026 | RPL21 | 12.072757 | 11.383462 | -0.689295 | 2.04E-08 | 5.21E-07 |
| ENSG00000166261 | ZNF202 | 8.513511 | 7.824306 | -0.689205 | 0.0002055 | 0.0019282 |
| ENSG00000173473 | SMARCC1 | 11.493959 | 10.80485 | -0.689109 | 8.09E-16 | 7.13E-14 |
| ENSG00000115425 | PECR | 6.8473839 | 6.1584122 | -0.688972 | 0.0005717 | 0.0045936 |
| ENSG00000234719 | NPIPB2 | 7.4278305 | 6.7394045 | -0.688426 | 2.52E-07 | 5.12E-06 |
| ENSG00000104870 | FCGRT | 13.415959 | 12.727541 | -0.688418 | 1.93E-05 | 0.0002444 |
| ENSG00000105135 | ILVBL | 8.9054005 | 8.2173795 | -0.688021 | 0.000177 | 0.0016932 |
| ENSG00000107262 | BAG1 | 10.230897 | 9.5430871 | -0.68781 | 3.22E-05 | 0.0003839 |
| ENSG00000148384 | INPP5E | 8.9910808 | 8.303283 | -0.687798 | 1.07E-08 | 2.87E-07 |
| ENSG00000273749 | CYFIP1 | 14.239537 | 13.551889 | -0.687648 | 4.10E-05 | 0.0004761 |
| ENSG00000168763 | CNNM3 | 10.034718 | 9.3470835 | -0.687635 | 1.31E-07 | 2.83E-06 |
| ENSG00000105472 | CLEC11A | 8.0977764 | 7.4104974 | -0.687279 | 0.001311 | 0.0093117 |
| ENSG00000179627 | ZBTB42 | 6.6100743 | 5.9232013 | -0.686873 | 0.0002484 | 0.002264 |
| ENSG00000135912 | TTLL4 | 11.100605 | 10.413767 | -0.686838 | 0.0001374 | 0.0013609 |
| ENSG00000134461 | ANKRD16 | 6.1347689 | 5.4485402 | -0.686229 | 0.0049445 | 0.0282142 |
| ENSG00000167747 | C19orf48 | 7.0712746 | 6.3852112 | -0.686063 | 0.0011337 | 0.0082273 |
| ENSG00000159228 | CBR1 | 9.9521242 | 9.2662251 | -0.685899 | 0.0017646 | 0.0119423 |
| ENSG00000167840 | ZNF232 | 6.8431498 | 6.1575671 | -0.685583 | 0.0002522 | 0.0022946 |
| ENSG00000124370 | MCEE | 6.8596282 | 6.1741961 | -0.685432 | 4.05E-05 | 0.0004709 |
| ENSG00000105197 | TIMM50 | 9.0502329 | 8.3648997 | -0.685333 | 0.0007604 | 0.0058659 |
| ENSG00000205609 | EIF3CL | 11.648201 | 10.963075 | -0.685126 | 0.0023545 | 0.0151608 |
| ENSG00000123395 | ATG101 | 9.0573336 | 8.372457 | -0.684877 | 0.0004881 | 0.0040218 |
| ENSG00000128309 | MPST | 8.9988111 | 8.3140353 | -0.684776 | 0.0001519 | 0.001485 |
| ENSG00000071794 | HLTF | 9.3255879 | 8.6408392 | -0.684749 | 2.07E-05 | 0.0002602 |
| ENSG00000100029 | PES1 | 10.304169 | 9.6211423 | -0.683027 | 0.0037137 | 0.0222772 |
| ENSG00000183751 | TBL3 | 9.0327455 | 8.349993 | -0.682752 | 0.0002195 | 0.0020337 |
| ENSG00000135218 | CD36 | 13.063328 | 12.380641 | -0.682687 | 0.0003188 | 0.0028026 |
| ENSG00000104907 | TRMT1 | 8.4331078 | 7.7505278 | -0.68258 | 8.81E-05 | 0.0009246 |
| ENSG00000147119 | CHST7 | 7.5397411 | 6.8573799 | -0.682361 | 0.0030812 | 0.0190224 |
| ENSG00000169689 | CENPX | 7.6802159 | 6.9981044 | -0.682111 | 0.0003049 | 0.0027018 |
| ENSG00000155714 | PDZD9 | 5.7203677 | 5.0384485 | -0.681919 | 0.0080822 | 0.0421003 |
| ENSG00000103047 | TANGO6 | 10.04405 | 9.3624139 | -0.681636 | 0.0001097 | 0.0011179 |
| ENSG00000187098 | MITF | 12.425182 | 11.743609 | -0.681573 | 0.0001332 | 0.0013258 |
| ENSG00000188846 | RPL14 | 11.360508 | 10.678983 | -0.681525 | 2.54E-06 | 4.03E-05 |
| ENSG00000100084 | HIRA | 11.112566 | 10.431491 | -0.681075 | 1.02E-08 | 2.75E-07 |
| ENSG00000167797 | CDK2AP2 | 8.5200403 | 7.8390756 | -0.680965 | 1.65E-05 | 0.0002118 |
| ENSG00000141524 | TMC6 | 11.064792 | 10.384307 | -0.680484 | 0.0001054 | 0.0010797 |
| ENSG00000087077 | TRIP6 | 9.0605849 | 8.3801879 | -0.680397 | 7.20E-05 | 0.0007759 |
| ENSG00000196126 | HLA-DRB1 | 14.551025 | 13.871533 | -0.679492 | 0.0020794 | 0.0136605 |
| ENSG00000035862 | TIMP2 | 14.610033 | 13.930894 | -0.679139 | 0.0006048 | 0.0048227 |
| ENSG00000204568 | MRPS18B | 9.7170485 | 9.0379509 | -0.679098 | 4.56E-07 | 8.70E-06 |
| ENSG00000137106 | GRHPR | 10.162007 | 9.4831506 | -0.678857 | 2.60E-05 | 0.0003169 |
| ENSG00000184110 | EIF3C | 11.499019 | 10.82038 | -0.678639 | 0.0021632 | 0.0141134 |
| ENSG00000100503 | NIN | 13.827212 | 13.148864 | -0.678347 | 1.85E-10 | 6.77E-09 |
| ENSG00000087245 | MMP2 | 12.034197 | 11.356921 | -0.677276 | 0.0013202 | 0.009366 |
| ENSG00000144283 | PKP4 | 8.8362028 | 8.1590439 | -0.677159 | 7.11E-06 | 0.0001015 |
| ENSG00000131323 | TRAF3 | 11.402762 | 10.725686 | -0.677076 | 0.0036673 | 0.0220443 |
| ENSG00000169499 | PLEKHA2 | 13.047304 | 12.370391 | -0.676912 | 2.26E-06 | 3.63E-05 |
| ENSG00000243056 | EIF4EBP3 | 5.7959002 | 5.1192663 | -0.676634 | 0.0012493 | 0.0089369 |
| ENSG00000240563 | L1TD1 | 3.9135104 | 3.2372578 | -0.676253 | 0.0056181 | 0.031255 |
| ENSG00000183617 | MRPL54 | 8.1509545 | 7.4748201 | -0.676134 | 0.0067587 | 0.0364064 |
| ENSG00000140992 | PDPK1 | 11.964807 | 11.289431 | -0.675376 | 0.0005571 | 0.0044911 |
| ENSG00000236320 | SLFN14 | 4.5704228 | 3.8951292 | -0.675294 | 0.0067853 | 0.0365269 |
| ENSG00000007520 | TSR3 | 8.1430086 | 7.4680938 | -0.674915 | 0.0013716 | 0.0096695 |
| ENSG00000187609 | EXD3 | 8.1232272 | 7.4492944 | -0.673933 | 0.0002446 | 0.0022358 |
| ENSG00000139988 | RDH12 | 6.8855433 | 6.2118096 | -0.673734 | 0.0002617 | 0.0023654 |
| ENSG00000130713 | EXOSC2 | 8.4745734 | 7.802437 | -0.672136 | 8.36E-05 | 0.0008836 |
| ENSG00000071655 | MBD3 | 10.733719 | 10.0623 | -0.671419 | 0.0001664 | 0.0016074 |
| ENSG00000273590 | SMIM11B | 6.5133315 | 5.8424861 | -0.670845 | 2.36E-05 | 0.0002916 |
| ENSG00000143870 | PDIA6 | 12.91966 | 12.249133 | -0.670528 | 1.30E-06 | 2.23E-05 |
| ENSG00000243678 | NME2 | 10.844371 | 10.174082 | -0.670289 | 2.10E-05 | 0.0002637 |
| ENSG00000154237 | LRRK1 | 11.479384 | 10.810004 | -0.66938 | 2.99E-09 | 8.87E-08 |
| ENSG00000204316 | MRPL38 | 8.9393887 | 8.2711545 | -0.668234 | 3.03E-07 | 6.07E-06 |
| ENSG00000118418 | HMGN3 | 9.3995647 | 8.7324284 | -0.667136 | 0.0003767 | 0.0032303 |
| ENSG00000139354 | GAS2L3 | 10.544084 | 9.8777783 | -0.666306 | 0.0002583 | 0.0023393 |
| ENSG00000188243 | COMMD6 | 8.3024212 | 7.6369877 | -0.665434 | 6.57E-05 | 0.0007158 |
| ENSG00000126814 | TRMT5 | 9.3338235 | 8.6685454 | -0.665278 | 0.0079312 | 0.0414816 |
| ENSG00000113269 | RNF130 | 13.775951 | 13.110704 | -0.665247 | 8.46E-12 | 3.95E-10 |
| ENSG00000114054 | PCCB | 10.541768 | 9.8772424 | -0.664526 | 0.005862 | 0.0323772 |
| ENSG00000167792 | NDUFV1 | 10.354377 | 9.6914243 | -0.662953 | 9.14E-05 | 0.0009546 |
| ENSG00000083123 | BCKDHB | 7.7721092 | 7.1094683 | -0.662641 | 0.0013511 | 0.0095434 |
| ENSG00000121989 | ACVR2A | 9.6924472 | 9.0310241 | -0.661423 | 4.93E-09 | 1.41E-07 |
| ENSG00000229809 | ZNF688 | 8.4132229 | 7.7524557 | -0.660767 | 0.002019 | 0.0133398 |
| ENSG00000100949 | RABGGTA | 9.5687828 | 8.9082186 | -0.660564 | 0.0015753 | 0.0108679 |
| ENSG00000167114 | SLC27A4 | 9.6639359 | 9.0049636 | -0.658972 | 0.0019941 | 0.0131913 |
| ENSG00000170468 | RIOX1 | 9.1618589 | 8.5032117 | -0.658647 | 0.0003953 | 0.0033634 |
| ENSG00000110057 | UNC93B1 | 12.011266 | 11.352984 | -0.658281 | 1.20E-05 | 0.0001613 |
| ENSG00000168924 | LETM1 | 10.657842 | 9.9995734 | -0.658268 | 0.0011785 | 0.0085111 |
| ENSG00000185917 | SETD4 | 8.7375163 | 8.0799616 | -0.657555 | 1.53E-10 | 5.68E-09 |
| ENSG00000125089 | SH3TC1 | 11.144311 | 10.487195 | -0.657116 | 0.0002108 | 0.0019675 |
| ENSG00000178982 | EIF3K | 11.350338 | 10.693388 | -0.656951 | 0.0001707 | 0.0016408 |
| ENSG00000145425 | RPS3A | 12.419435 | 11.762518 | -0.656916 | 2.09E-06 | 3.40E-05 |
| ENSG00000159335 | PTMS | 11.268784 | 10.611914 | -0.65687 | 0.0005425 | 0.0043949 |
| ENSG00000168395 | ING5 | 9.7874791 | 9.1311146 | -0.656365 | 1.08E-06 | 1.88E-05 |
| ENSG00000183597 | TANGO2 | 11.81182 | 11.155464 | -0.656357 | 1.56E-05 | 0.0002017 |
| ENSG00000119333 | WDR34 | 6.1817458 | 5.5258079 | -0.655938 | 0.0027563 | 0.0172873 |
| ENSG00000250565 | ATP6V1E2 | 6.2682505 | 5.6123598 | -0.655891 | 0.0005316 | 0.0043166 |
| ENSG00000164818 | DNAAF5 | 8.6684594 | 8.0128232 | -0.655636 | 0.0003819 | 0.0032654 |
| ENSG00000127951 | FGL2 | 12.048452 | 11.393295 | -0.655158 | 0.0011815 | 0.0085289 |
| ENSG00000187051 | RPS19BP1 | 10.014357 | 9.3596811 | -0.654676 | 0.000131 | 0.0013063 |
| ENSG00000132199 | ENOSF1 | 11.970259 | 11.316789 | -0.653469 | 0.0004611 | 0.0038386 |
| ENSG00000129226 | CD68 | 14.821956 | 14.169025 | -0.652932 | 0.0001321 | 0.0013152 |
| ENSG00000103423 | DNAJA3 | 9.2174455 | 8.5648416 | -0.652604 | 6.66E-05 | 0.0007241 |
| ENSG00000196275 | GTF2IRD2 | 8.4430102 | 7.7904413 | -0.652569 | 9.96E-06 | 0.0001364 |
| ENSG00000143891 | GALM | 11.013708 | 10.362396 | -0.651312 | 4.58E-05 | 0.0005231 |
| ENSG00000188026 | RILPL1 | 10.215617 | 9.5648014 | -0.650815 | 0.0005812 | 0.0046625 |
| ENSG00000140650 | PMM2 | 9.9719043 | 9.3214838 | -0.65042 | 0.00575 | 0.0318581 |
| ENSG00000108474 | PIGL | 8.0353895 | 7.3852407 | -0.650149 | 3.20E-05 | 0.0003814 |
| ENSG00000172315 | TP53RK | 8.9253567 | 8.2753397 | -0.650017 | 0.0017801 | 0.0120265 |
| ENSG00000184216 | IRAK1 | 11.7876 | 11.137723 | -0.649877 | 0.0039367 | 0.0233965 |
| ENSG00000184887 | BTBD6 | 9.4793353 | 8.8318165 | -0.647519 | 1.37E-05 | 0.0001805 |
| ENSG00000121716 | PILRB | 8.7853005 | 8.1378851 | -0.647415 | 0.0037315 | 0.0223511 |
| ENSG00000145780 | FEM1C | 11.746327 | 11.099193 | -0.647134 | 4.48E-06 | 6.69E-05 |
| ENSG00000123500 | COL10A1 | 8.0988097 | 7.4520762 | -0.646734 | 0.0001569 | 0.0015271 |
| ENSG00000132274 | TRIM22 | 11.735981 | 11.089336 | -0.646644 | 5.06E-06 | 7.45E-05 |
| ENSG00000115286 | NDUFS7 | 9.5386462 | 8.8920969 | -0.646549 | 7.25E-05 | 0.0007814 |
| ENSG00000137070 | IL11RA | 7.9118566 | 7.2654761 | -0.64638 | 0.0008445 | 0.006409 |
| ENSG00000278540 | ACACA | 10.546367 | 9.9003046 | -0.646062 | 0.0011238 | 0.0081686 |
| ENSG00000242110 | AMACR | 8.5389812 | 7.893588 | -0.645393 | 0.0004697 | 0.0038932 |
| ENSG00000141385 | AFG3L2 | 11.338386 | 10.693181 | -0.645205 | 0.0001082 | 0.0011059 |
| ENSG00000157911 | PEX10 | 8.2344555 | 7.5897046 | -0.644751 | 0.0022056 | 0.0143363 |
| ENSG00000185379 | RAD51D | 8.5982999 | 7.9537097 | -0.64459 | 3.24E-07 | 6.43E-06 |
| ENSG00000118965 | WDR35 | 8.3540083 | 7.709843 | -0.644165 | 0.0009664 | 0.0072038 |
| ENSG00000174428 | GTF2IRD2B | 8.8925912 | 8.2488178 | -0.643773 | 9.40E-06 | 0.0001296 |
| ENSG00000111203 | ITFG2 | 9.8423139 | 9.1987487 | -0.643565 | 9.18E-06 | 0.0001271 |
| ENSG00000158062 | UBXN11 | 11.140942 | 10.498443 | -0.642499 | 5.91E-05 | 0.0006546 |
| ENSG00000125449 | ARMC7 | 8.7040421 | 8.0629157 | -0.641126 | 0.0075423 | 0.039835 |
| ENSG00000130304 | SLC27A1 | 11.440719 | 10.799871 | -0.640849 | 0.0007924 | 0.0060694 |
| ENSG00000204946 | ZNF783 | 9.4934675 | 8.8545119 | -0.638956 | 0.0003366 | 0.0029349 |
| ENSG00000001497 | LAS1L | 9.4946163 | 8.8562148 | -0.638401 | 6.57E-05 | 0.0007158 |
| ENSG00000180694 | TMEM64 | 8.4005962 | 7.762503 | -0.638093 | 0.0065882 | 0.0356767 |
| ENSG00000165802 | NSMF | 8.540278 | 7.9034726 | -0.636805 | 0.0001062 | 0.0010873 |
| ENSG00000142327 | RNPEPL1 | 10.670109 | 10.033316 | -0.636792 | 0.0001184 | 0.0011955 |
| ENSG00000142541 | RPL13A | 13.617178 | 12.9806 | -0.636578 | 5.51E-09 | 1.55E-07 |
| ENSG00000152127 | MGAT5 | 13.196495 | 12.56036 | -0.636135 | 1.21E-05 | 0.0001615 |
| ENSG00000206561 | COLQ | 6.401196 | 5.7664934 | -0.634703 | 0.0010242 | 0.0075617 |
| ENSG00000157240 | FZD1 | 10.093829 | 9.459191 | -0.634638 | 0.0001602 | 0.0015561 |
| ENSG00000087269 | NOP14 | 10.035271 | 9.4018636 | -0.633408 | 0.0002033 | 0.0019097 |
| ENSG00000100154 | TTC28 | 10.05147 | 9.4183391 | -0.633131 | 0.0077286 | 0.0406012 |
| ENSG00000197312 | DDI2 | 13.100086 | 12.467163 | -0.632923 | 0.0053801 | 0.0301606 |
| ENSG00000095319 | NUP188 | 11.210833 | 10.578086 | -0.632747 | 0.0040092 | 0.0237471 |
| ENSG00000143793 | C1orf35 | 7.2110352 | 6.5785628 | -0.632472 | 0.0004619 | 0.0038426 |
| ENSG00000116729 | WLS | 9.7433576 | 9.1112625 | -0.632095 | 0.000714 | 0.0055651 |
| ENSG00000153933 | DGKE | 8.7305225 | 8.0992275 | -0.631295 | 0.0079456 | 0.0415367 |
| ENSG00000126561 | STAT5A | 12.598285 | 11.967435 | -0.63085 | 0.0004616 | 0.003841 |
| ENSG00000188986 | NELFB | 10.220901 | 9.5908956 | -0.630006 | 0.0003528 | 0.0030555 |
| ENSG00000243989 | ACY1 | 8.0734706 | 7.443467 | -0.630004 | 0.0051971 | 0.0293302 |
| ENSG00000121454 | LHX4 | 9.0950877 | 8.4651871 | -0.629901 | 1.54E-06 | 2.60E-05 |
| ENSG00000175426 | PCSK1 | 8.7419963 | 8.1121766 | -0.62982 | 0.0070431 | 0.0377129 |
| ENSG00000110446 | SLC15A3 | 12.887711 | 12.258133 | -0.629578 | 0.002813 | 0.0176004 |
| ENSG00000105519 | CAPS | 7.8568986 | 7.2277708 | -0.629128 | 0.0015457 | 0.0106957 |
| ENSG00000104823 | ECH1 | 10.087273 | 9.4582392 | -0.629034 | 5.86E-06 | 8.52E-05 |
| ENSG00000100814 | CCNB1IP1 | 6.7061977 | 6.0776034 | -0.628594 | 0.0003419 | 0.002975 |
| ENSG00000197162 | ZNF785 | 8.6215522 | 7.9932747 | -0.628278 | 8.50E-05 | 0.0008974 |
| ENSG00000146066 | HIGD2A | 9.8415873 | 9.2134716 | -0.628116 | 2.81E-07 | 5.66E-06 |
| ENSG00000149716 | ORAOV1 | 9.0475169 | 8.4200855 | -0.627431 | 0.0002112 | 0.0019701 |
| ENSG00000103415 | HMOX2 | 9.7029755 | 9.0766826 | -0.626293 | 5.69E-05 | 0.0006336 |
| ENSG00000101439 | CST3 | 13.844615 | 13.218493 | -0.626123 | 0.0019311 | 0.0128688 |
| ENSG00000154079 | SDHAF4 | 6.6891636 | 6.0633759 | -0.625788 | 6.77E-05 | 0.0007353 |
| ENSG00000162104 | ADCY9 | 10.447829 | 9.8220943 | -0.625735 | 2.97E-06 | 4.64E-05 |
| ENSG00000137161 | CNPY3 | 11.768116 | 11.142462 | -0.625654 | 5.99E-05 | 0.0006612 |
| ENSG00000108298 | RPL19 | 13.202994 | 12.577384 | -0.625611 | 1.09E-12 | 5.79E-11 |
| ENSG00000168675 | LDLRAD4 | 11.59543 | 10.970328 | -0.625102 | 8.06E-07 | 1.45E-05 |
| ENSG00000116786 | PLEKHM2 | 13.248871 | 12.624677 | -0.624194 | 4.92E-06 | 7.27E-05 |
| ENSG00000154803 | FLCN | 11.718535 | 11.094747 | -0.623788 | 0.0047355 | 0.0272275 |
| ENSG00000205339 | IPO7 | 12.540875 | 11.918548 | -0.622327 | 1.22E-07 | 2.66E-06 |
| ENSG00000188917 | TRMT2B | 9.6228241 | 9.0008214 | -0.622003 | 3.73E-09 | 1.08E-07 |
| ENSG00000155368 | DBI | 11.256331 | 10.634458 | -0.621873 | 3.52E-06 | 5.39E-05 |
| ENSG00000177370 | TIMM22 | 10.24012 | 9.6190057 | -0.621114 | 3.87E-07 | 7.49E-06 |
| ENSG00000101160 | CTSZ | 16.965363 | 16.344509 | -0.620855 | 3.43E-05 | 0.0004066 |
| ENSG00000187122 | SLIT1 | 6.2835664 | 5.6633748 | -0.620192 | 0.0010178 | 0.0075222 |
| ENSG00000164576 | SAP30L | 10.181239 | 9.562182 | -0.619057 | 5.41E-08 | 1.27E-06 |
| ENSG00000116016 | EPAS1 | 13.537521 | 12.919086 | -0.618435 | 0.0006034 | 0.0048137 |
| ENSG00000172869 | DMXL1 | 14.033815 | 13.416339 | -0.617476 | 2.39E-05 | 0.000295 |
| ENSG00000168906 | MAT2A | 12.556261 | 11.938867 | -0.617394 | 0.0017116 | 0.0116439 |
| ENSG00000039523 | RIPOR1 | 12.090133 | 11.473269 | -0.616865 | 4.89E-06 | 7.23E-05 |
| ENSG00000188878 | FBF1 | 8.0835866 | 7.4667346 | -0.616852 | 0.0085746 | 0.0441865 |
| ENSG00000155506 | LARP1 | 13.165982 | 12.549476 | -0.616506 | 5.04E-07 | 9.50E-06 |
| ENSG00000185669 | SNAI3 | 7.3862544 | 6.7698648 | -0.61639 | 0.0090891 | 0.0463684 |
| ENSG00000151552 | QDPR | 9.3794859 | 8.7633152 | -0.616171 | 1.52E-06 | 2.57E-05 |
| ENSG00000182670 | TTC3 | 13.088291 | 12.473517 | -0.614775 | 2.52E-13 | 1.49E-11 |
| ENSG00000131233 | GJA9 | 8.1465059 | 7.5322666 | -0.614239 | 0.0011179 | 0.0081357 |
| ENSG00000105176 | URI1 | 10.165585 | 9.5517521 | -0.613833 | 2.35E-05 | 0.0002912 |
| ENSG00000164323 | CFAP97 | 9.805852 | 9.1920587 | -0.613793 | 0.0014333 | 0.0100266 |
| ENSG00000273173 | SNURF | 6.2873736 | 5.6736544 | -0.613719 | 0.0005975 | 0.0047749 |
| ENSG00000187109 | NAP1L1 | 12.769015 | 12.155337 | -0.613678 | 3.84E-07 | 7.44E-06 |
| ENSG00000188542 | DUSP28 | 8.0231808 | 7.4112683 | -0.611912 | 0.0051762 | 0.0292511 |
| ENSG00000113504 | SLC12A7 | 12.877423 | 12.265593 | -0.61183 | 4.28E-06 | 6.43E-05 |
| ENSG00000013306 | SLC25A39 | 9.7768162 | 9.165102 | -0.611714 | 0.0006362 | 0.0050335 |
| ENSG00000105204 | DYRK1B | 8.7205153 | 8.1088903 | -0.611625 | 0.000128 | 0.001281 |
| ENSG00000242259 | C22orf39 | 8.7078389 | 8.0962937 | -0.611545 | 1.06E-08 | 2.84E-07 |
| ENSG00000019582 | CD74 | 17.171207 | 16.559761 | -0.611446 | 2.97E-05 | 0.0003573 |
| ENSG00000137760 | ALKBH8 | 7.6207509 | 7.0093507 | -0.6114 | 0.0066444 | 0.0359098 |
| ENSG00000148300 | REXO4 | 9.5068549 | 8.8964825 | -0.610372 | 1.09E-06 | 1.90E-05 |
| ENSG00000168028 | RPSA | 12.010479 | 11.400802 | -0.609677 | 6.09E-07 | 1.13E-05 |
| ENSG00000134490 | TMEM241 | 7.1362131 | 6.5265439 | -0.609669 | 0.0001713 | 0.001645 |
| ENSG00000100292 | HMOX1 | 13.325681 | 12.716064 | -0.609617 | 0.0057443 | 0.0318316 |
| ENSG00000104228 | TRIM35 | 9.2848513 | 8.6757767 | -0.609075 | 0.0002362 | 0.0021677 |
| ENSG00000205670 | SMIM11A | 6.7250949 | 6.1161277 | -0.608967 | 0.000219 | 0.0020297 |
| ENSG00000150990 | DHX37 | 9.145186 | 8.5362322 | -0.608954 | 0.0063978 | 0.0348137 |
| ENSG00000124532 | MRS2 | 10.053041 | 9.4441559 | -0.608885 | 1.06E-05 | 0.0001442 |
| ENSG00000130313 | PGLS | 9.8682653 | 9.2595877 | -0.608678 | 2.76E-05 | 0.0003342 |
| ENSG00000162650 | ATXN7L2 | 7.153333 | 6.5452165 | -0.608117 | 0.0008136 | 0.0062096 |
| ENSG00000127540 | UQCR11 | 10.082879 | 9.4760865 | -0.606792 | 0.0063484 | 0.0345793 |
| ENSG00000101417 | PXMP4 | 7.867735 | 7.2613888 | -0.606346 | 2.49E-05 | 0.0003061 |
| ENSG00000136478 | TEX2 | 11.178334 | 10.572689 | -0.605644 | 0.0022711 | 0.0146861 |
| ENSG00000143507 | DUSP10 | 11.012478 | 10.407049 | -0.605429 | 2.19E-05 | 0.000274 |
| ENSG00000141564 | RPTOR | 11.060629 | 10.455936 | -0.604693 | 5.75E-05 | 0.0006389 |
| ENSG00000176454 | LPCAT4 | 9.3007735 | 8.6961821 | -0.604591 | 0.0002388 | 0.0021884 |
| ENSG00000130038 | CRACR2A | 8.16233 | 7.5578986 | -0.604431 | 0.002869 | 0.0178753 |
| ENSG00000163029 | SMC6 | 10.578234 | 9.9744505 | -0.603784 | 3.04E-06 | 4.73E-05 |
| ENSG00000165688 | PMPCA | 10.289709 | 9.6862314 | -0.603478 | 7.38E-07 | 1.34E-05 |
| ENSG00000183161 | FANCF | 7.1318615 | 6.5286057 | -0.603256 | 0.0020969 | 0.0137547 |
| ENSG00000126882 | FAM78A | 11.543346 | 10.940462 | -0.602884 | 0.0046112 | 0.0266493 |
| ENSG00000186017 | ZNF566 | 7.9673738 | 7.3646951 | -0.602679 | 0.0001826 | 0.0017394 |
| ENSG00000125375 | ATP5S | 7.5454289 | 6.9428506 | -0.602578 | 0.0028237 | 0.0176519 |
| ENSG00000270629 | NBPF14 | 12.943921 | 12.341443 | -0.602478 | 3.53E-05 | 0.0004151 |
| ENSG00000128626 | MRPS12 | 7.0711375 | 6.4701526 | -0.600985 | 0.0030969 | 0.019106 |
| ENSG00000099821 | POLRMT | 9.6957211 | 9.0956972 | -0.600024 | 0.0001497 | 0.0014648 |
| ENSG00000145919 | BOD1 | 8.2995756 | 7.7004043 | -0.599171 | 0.0001078 | 0.0011022 |
| ENSG00000126091 | ST3GAL3 | 8.1659811 | 7.5674095 | -0.598572 | 0.0005167 | 0.004217 |
| ENSG00000174917 | C19orf70 | 7.8091177 | 7.2108731 | -0.598245 | 0.0003143 | 0.0027657 |
| ENSG00000110031 | LPXN | 12.867771 | 12.269766 | -0.598005 | 2.07E-06 | 3.36E-05 |
| ENSG00000100403 | ZC3H7B | 11.46586 | 10.867857 | -0.598004 | 9.99E-06 | 0.0001368 |
| ENSG00000257365 | FNTB | 9.7853903 | 9.1878786 | -0.597512 | 2.62E-05 | 0.0003198 |
| ENSG00000111801 | BTN3A3 | 9.7014602 | 9.1042564 | -0.597204 | 0.0011059 | 0.0080639 |
| ENSG00000247077 | PGAM5 | 8.6570251 | 8.060058 | -0.596967 | 0.003208 | 0.019672 |
| ENSG00000229474 | PATL2 | 8.112131 | 7.5152294 | -0.596902 | 0.0011325 | 0.0082221 |
| ENSG00000197548 | ATG7 | 12.973085 | 12.376339 | -0.596746 | 6.27E-06 | 9.03E-05 |
| ENSG00000179981 | TSHZ1 | 10.386857 | 9.7902546 | -0.596602 | 0.0007498 | 0.0057985 |
| ENSG00000089053 | ANAPC5 | 11.189276 | 10.592811 | -0.596465 | 2.02E-10 | 7.35E-09 |
| ENSG00000198680 | TUSC1 | 6.8408539 | 6.2444498 | -0.596404 | 0.005273 | 0.0296858 |
| ENSG00000125995 | ROMO1 | 7.6573064 | 7.0609189 | -0.596387 | 0.0049153 | 0.0280767 |
| ENSG00000163521 | GLB1L | 9.2888342 | 8.6925081 | -0.596326 | 0.0011537 | 0.0083597 |
| ENSG00000079950 | STX7 | 13.74204 | 13.145993 | -0.596047 | 8.54E-05 | 0.0009008 |
| ENSG00000173456 | RNF26 | 9.3350377 | 8.7394421 | -0.595596 | 0.0005662 | 0.0045558 |
| ENSG00000135541 | AHI1 | 9.9020278 | 9.3066901 | -0.595338 | 0.0035184 | 0.0212745 |
| ENSG00000182774 | RPS17 | 10.655432 | 10.060274 | -0.595158 | 0.0013766 | 0.0096937 |
| ENSG00000168970 | JMJD7-PLA2G4B | 8.7445005 | 8.1493495 | -0.595151 | 0.0005064 | 0.0041469 |
| ENSG00000240230 | COX19 | 9.9210348 | 9.3262082 | -0.594827 | 1.70E-07 | 3.58E-06 |
| ENSG00000105248 | CCDC94 | 9.1522968 | 8.5592874 | -0.593009 | 0.0002087 | 0.0019551 |
| ENSG00000134250 | NOTCH2 | 15.515633 | 14.92273 | -0.592903 | 3.39E-10 | 1.20E-08 |
| ENSG00000006625 | GGCT | 8.5328515 | 7.9405756 | -0.592276 | 0.006642 | 0.0359015 |
| ENSG00000143353 | LYPLAL1 | 9.6802743 | 9.0880157 | -0.592259 | 4.94E-05 | 0.0005615 |
| ENSG00000107404 | DVL1 | 8.7458699 | 8.1545344 | -0.591335 | 1.44E-05 | 0.0001874 |
| ENSG00000124215 | CDH26 | 6.9149678 | 6.3236911 | -0.591277 | 1.25E-05 | 0.000166 |
| ENSG00000146729 | NIPSNAP2 | 11.451401 | 10.860442 | -0.590959 | 4.59E-07 | 8.74E-06 |
| ENSG00000102053 | ZC3H12B | 5.5580158 | 4.9673238 | -0.590692 | 0.0032095 | 0.0196771 |
| ENSG00000166441 | RPL27A | 11.95778 | 11.367173 | -0.590607 | 4.46E-07 | 8.54E-06 |
| ENSG00000173581 | CCDC106 | 8.7614369 | 8.1709285 | -0.590508 | 0.0003957 | 0.0033658 |
| ENSG00000164062 | APEH | 11.101488 | 10.511299 | -0.59019 | 0.0003038 | 0.0026927 |
| ENSG00000156709 | AIFM1 | 10.676783 | 10.086864 | -0.589919 | 0.0001114 | 0.0011344 |
| ENSG00000198917 | SPOUT1 | 8.4756689 | 7.8857719 | -0.589897 | 0.0091408 | 0.0465343 |
| ENSG00000164081 | TEX264 | 10.196449 | 9.606656 | -0.589793 | 9.61E-07 | 1.70E-05 |
| ENSG00000107679 | PLEKHA1 | 11.039567 | 10.449804 | -0.589763 | 0.0002252 | 0.0020816 |
| ENSG00000125520 | SLC2A4RG | 7.7510204 | 7.1616552 | -0.589365 | 0.0087187 | 0.0447808 |
| ENSG00000180263 | FGD6 | 12.726488 | 12.137412 | -0.589077 | 8.73E-06 | 0.0001217 |
| ENSG00000090674 | MCOLN1 | 11.712893 | 11.12386 | -0.589033 | 0.002215 | 0.0143807 |
| ENSG00000266967 | AARSD1 | 7.671067 | 7.0821892 | -0.588878 | 0.0021338 | 0.0139533 |
| ENSG00000163131 | CTSS | 15.642089 | 15.053325 | -0.588764 | 0.0003223 | 0.0028282 |
| ENSG00000136940 | PDCL | 9.8385497 | 9.2498516 | -0.588698 | 3.80E-06 | 5.79E-05 |
| ENSG00000008382 | MPND | 9.1120007 | 8.5234721 | -0.588529 | 0.0045186 | 0.0262076 |
| ENSG00000147155 | EBP | 8.75935 | 8.1710699 | -0.58828 | 0.0016058 | 0.0110428 |
| ENSG00000159445 | THEM4 | 7.9130965 | 7.324975 | -0.588121 | 0.0014905 | 0.0103711 |
| ENSG00000186767 | SPIN4 | 7.0563944 | 6.469583 | -0.586811 | 0.0046011 | 0.026598 |
| ENSG00000167965 | MLST8 | 8.7255697 | 8.1388888 | -0.586681 | 6.37E-06 | 9.16E-05 |
| ENSG00000141506 | PIK3R5 | 12.897741 | 12.311159 | -0.586583 | 7.78E-08 | 1.76E-06 |
| ENSG00000163938 | GNL3 | 9.5577141 | 8.9715497 | -0.586164 | 0.0005042 | 0.0041351 |
| ENSG00000115053 | NCL | 12.744207 | 12.158828 | -0.585379 | 3.21E-07 | 6.37E-06 |
| ENSG00000168564 | CDKN2AIP | 9.4045608 | 8.8195115 | -0.585049 | 0.004483 | 0.0260319 |

**Table S2** DEGs in FM+Mlep vs FM.

| **Gene_ID** | **Symbol** | **FM** | **FM+Mlep** | **FM+Mlep_vs_FM_log2FoldChange** | **FM+Mlep_vs_FM_Pvalue** | **FM+Mlep_vs_FM_FDR** |
| --- | --- | --- | --- | --- | --- | --- |
| ENSG00000205358 | MT1H | 1.5301955 | 11.682449 | 10.152253 | 2.46E-28 | 3.37E-25 |
| ENSG00000125144 | MT1G | 2.2150927 | 11.924338 | 9.7092451 | 1.08E-37 | 3.65E-34 |
| ENSG00000163735 | CXCL5 | 5.9672304 | 15.233497 | 9.2662664 | 5.84E-82 | 2.56E-77 |
| ENSG00000168685 | IL7R | 5.4325526 | 14.136818 | 8.7042649 | 8.68E-33 | 1.73E-29 |
| ENSG00000205364 | MT1M | 0.2009975 | 8.1175734 | 7.9165759 | 3.80E-26 | 3.79E-23 |
| ENSG00000186407 | CD300E | 6.2512572 | 13.55479 | 7.3035324 | 5.40E-24 | 3.65E-21 |
| ENSG00000196611 | MMP1 | 1.8693816 | 8.8602156 | 6.9908341 | 4.76E-14 | 8.26E-12 |
| ENSG00000163736 | PPBP | 0 | 6.8970679 | 6.8970679 | 6.97E-22 | 3.64E-19 |
| ENSG00000108702 | CCL1 | 0.9895795 | 7.8492729 | 6.8596934 | 8.99E-13 | 1.25E-10 |
| ENSG00000143546 | S100A8 | 5.9348558 | 12.657771 | 6.722915 | 6.12E-31 | 1.07E-27 |
| ENSG00000108688 | CCL7 | 4.238301 | 10.935828 | 6.6975268 | 1.89E-42 | 9.22E-39 |
| ENSG00000169715 | MT1E | 3.904842 | 10.495082 | 6.5902397 | 3.99E-35 | 1.25E-31 |
| ENSG00000198417 | MT1F | 4.2447032 | 10.766828 | 6.5221252 | 4.77E-55 | 1.05E-50 |
| ENSG00000125148 | MT2A | 6.1331564 | 12.403639 | 6.2704826 | 4.75E-33 | 1.09E-29 |
| ENSG00000152766 | ANKRD22 | 0.1588355 | 6.4051924 | 6.2463569 | 1.41E-25 | 1.21E-22 |
| ENSG00000187193 | MT1X | 5.6674913 | 11.807424 | 6.1399327 | 5.95E-41 | 2.37E-37 |
| ENSG00000138135 | CH25H | 1.777328 | 7.8909926 | 6.1136646 | 3.78E-17 | 1.09E-14 |
| ENSG00000115008 | IL1A | 3.0035477 | 9.0418117 | 6.038264 | 2.63E-20 | 1.10E-17 |
| ENSG00000117090 | SLAMF1 | 2.715816 | 8.7221116 | 6.0062956 | 1.12E-48 | 8.22E-45 |
| ENSG00000275718 | CCL15 | 2.2828604 | 8.2625143 | 5.9796538 | 1.91E-08 | 9.93E-07 |
| ENSG00000144681 | STAC | 4.8700774 | 10.804766 | 5.9346884 | 5.66E-08 | 2.62E-06 |
| ENSG00000163221 | S100A12 | 0.260922 | 6.0771521 | 5.81623 | 1.66E-14 | 3.16E-12 |
| ENSG00000149968 | MMP3 | 1.3663164 | 7.1300758 | 5.7637594 | 3.34E-13 | 4.95E-11 |
| ENSG00000117594 | HSD11B1 | 6.3951727 | 12.117525 | 5.7223526 | 1.39E-17 | 4.27E-15 |
| ENSG00000050730 | TNIP3 | 5.1657653 | 10.835075 | 5.6693102 | 5.98E-26 | 5.35E-23 |
| ENSG00000074410 | CA12 | 2.8478041 | 8.4950087 | 5.6472046 | 2.90E-07 | 1.10E-05 |
| ENSG00000101331 | CCM2L | 1.7991764 | 7.4275166 | 5.6283402 | 4.24E-12 | 5.06E-10 |
| ENSG00000125538 | IL1B | 7.1241415 | 12.739847 | 5.6157051 | 4.98E-33 | 1.09E-29 |
| ENSG00000106178 | CCL24 | 5.4630162 | 10.880223 | 5.4172071 | 6.02E-19 | 2.15E-16 |
| ENSG00000160307 | S100B | 1.7966247 | 6.9010916 | 5.1044669 | 9.50E-09 | 5.28E-07 |
| ENSG00000099998 | GGT5 | 1.0578716 | 6.1496554 | 5.0917838 | 1.07E-13 | 1.75E-11 |
| ENSG00000136960 | ENPP2 | 6.123015 | 11.181036 | 5.0580209 | 1.86E-08 | 9.70E-07 |
| ENSG00000038427 | VCAN | 7.7743823 | 12.795523 | 5.0211409 | 6.63E-30 | 1.08E-26 |
| ENSG00000112195 | TREML2 | 1.6999019 | 6.6350755 | 4.9351735 | 3.57E-08 | 1.72E-06 |
| ENSG00000170956 | CEACAM3 | 2.3383581 | 7.2728975 | 4.9345394 | 5.54E-11 | 5.36E-09 |
| ENSG00000104918 | RETN | 1.5966453 | 6.3337109 | 4.7370656 | 0.0062681 | 0.0472259 |
| ENSG00000139178 | C1RL | 5.0203346 | 9.7304029 | 4.7100684 | 4.90E-10 | 3.80E-08 |
| ENSG00000100336 | APOL4 | 1.8885866 | 6.5248216 | 4.636235 | 4.41E-06 | 0.0001156 |
| ENSG00000124731 | TREM1 | 6.0425313 | 10.651147 | 4.6086159 | 1.26E-43 | 7.93E-40 |
| ENSG00000174837 | ADGRE1 | 6.3748063 | 10.955849 | 4.5810425 | 1.61E-33 | 3.93E-30 |
| ENSG00000138316 | ADAMTS14 | 3.6077271 | 8.1619336 | 4.5542065 | 3.75E-09 | 2.31E-07 |
| ENSG00000171049 | FPR2 | 4.1540437 | 8.7034478 | 4.5494041 | 2.53E-27 | 3.18E-24 |
| ENSG00000105639 | JAK3 | 6.2350133 | 10.725178 | 4.4901645 | 8.62E-16 | 2.07E-13 |
| ENSG00000112394 | SLC16A10 | 6.4901001 | 10.959854 | 4.4697542 | 7.18E-22 | 3.71E-19 |
| ENSG00000274736 | CCL23 | 2.2957037 | 6.762176 | 4.4664723 | 9.48E-12 | 1.05E-09 |
| ENSG00000136244 | IL6 | 3.3067677 | 7.7025898 | 4.395822 | 8.16E-24 | 5.35E-21 |
| ENSG00000102794 | ACOD1 | 1.2369545 | 5.6227538 | 4.3857993 | 1.31E-06 | 4.05E-05 |
| ENSG00000122641 | INHBA | 4.9120808 | 9.2608728 | 4.348792 | 3.55E-26 | 3.63E-23 |
| ENSG00000172551 | MUCL1 | 0.8028021 | 5.1214048 | 4.3186027 | 1.23E-07 | 5.25E-06 |
| ENSG00000105855 | ITGB8 | 5.4454852 | 9.7630395 | 4.3175542 | 1.83E-43 | 1.00E-39 |
| ENSG00000110436 | SLC1A2 | 2.9491985 | 7.2166163 | 4.2674179 | 0.0009798 | 0.0109073 |
| ENSG00000262406 | MMP12 | 2.8575541 | 7.1231073 | 4.2655532 | 9.79E-06 | 0.0002295 |
| ENSG00000131203 | IDO1 | 0.7063015 | 4.9537635 | 4.247462 | 2.24E-10 | 1.87E-08 |
| ENSG00000142949 | PTPRF | 2.7397666 | 6.945311 | 4.2055444 | 5.80E-13 | 8.35E-11 |
| ENSG00000132965 | ALOX5AP | 6.7404652 | 10.942018 | 4.2015524 | 8.11E-20 | 3.21E-17 |
| ENSG00000254521 | SIGLEC12 | 3.0102202 | 7.2116763 | 4.2014561 | 2.21E-05 | 0.000463 |
| ENSG00000183019 | MCEMP1 | 6.2791641 | 10.439415 | 4.160251 | 1.57E-07 | 6.51E-06 |
| ENSG00000078081 | LAMP3 | 0.5474031 | 4.6783831 | 4.13098 | 4.76E-10 | 3.72E-08 |
| ENSG00000137757 | CASP5 | 3.0506077 | 7.169189 | 4.1185813 | 8.21E-17 | 2.22E-14 |
| ENSG00000148344 | PTGES | 2.4995344 | 6.5941949 | 4.0946605 | 5.53E-08 | 2.57E-06 |
| ENSG00000123689 | G0S2 | 5.8596358 | 9.9523167 | 4.0926809 | 8.42E-16 | 2.03E-13 |
| ENSG00000166670 | MMP10 | 0.5631213 | 4.6087733 | 4.045652 | 4.13E-10 | 3.29E-08 |
| ENSG00000163739 | CXCL1 | 8.5260566 | 12.547853 | 4.0217961 | 2.94E-15 | 6.53E-13 |
| ENSG00000139194 | RBP5 | 0.8730035 | 4.8744009 | 4.0013974 | 2.32E-14 | 4.23E-12 |
| ENSG00000166689 | PLEKHA7 | 4.5673843 | 8.5645064 | 3.9971221 | 1.44E-24 | 1.07E-21 |
| ENSG00000138821 | SLC39A8 | 10.75857 | 14.735763 | 3.9771936 | 2.92E-41 | 1.28E-37 |
| ENSG00000157551 | KCNJ15 | 3.9054981 | 7.8697148 | 3.9642168 | 0.0041712 | 0.034436 |
| ENSG00000258227 | CLEC5A | 8.5621715 | 12.522623 | 3.9604518 | 2.32E-26 | 2.49E-23 |
| ENSG00000167105 | TMEM92 | 0 | 3.9375901 | 3.9375901 | 1.65E-08 | 8.66E-07 |
| ENSG00000184557 | SOCS3 | 7.6767188 | 11.603586 | 3.9268675 | 3.00E-20 | 1.24E-17 |
| ENSG00000120833 | SOCS2 | 3.0888277 | 7.00698 | 3.9181523 | 0.0006451 | 0.0077995 |
| ENSG00000105246 | EBI3 | 3.7137299 | 7.631875 | 3.9181452 | 1.83E-17 | 5.51E-15 |
| ENSG00000166527 | CLEC4D | 5.15834 | 9.0404413 | 3.8821013 | 8.81E-12 | 9.87E-10 |
| ENSG00000150510 | FAM124A | 4.077877 | 7.9484866 | 3.8706096 | 1.47E-10 | 1.28E-08 |
| ENSG00000172986 | GXYLT2 | 0.9525223 | 4.8180114 | 3.8654892 | 1.17E-10 | 1.06E-08 |
| ENSG00000124875 | CXCL6 | 0.4529469 | 4.29563 | 3.842683 | 8.68E-11 | 8.12E-09 |
| ENSG00000114805 | PLCH1 | 0.981396 | 4.8187861 | 3.8373901 | 1.70E-06 | 5.09E-05 |
| ENSG00000205846 | CLEC6A | 5.0533413 | 8.8890858 | 3.8357445 | 2.21E-18 | 7.28E-16 |
| ENSG00000105976 | MET | 2.1696569 | 5.9101963 | 3.7405394 | 4.85E-10 | 3.76E-08 |
| ENSG00000133116 | KL | 1.156941 | 4.8575416 | 3.7006006 | 2.16E-05 | 0.0004544 |
| ENSG00000101333 | PLCB4 | 1.6186977 | 5.2413133 | 3.6226156 | 6.73E-08 | 3.07E-06 |
| ENSG00000124479 | NDP | 0.2075964 | 3.8298223 | 3.6222259 | 3.81E-07 | 1.39E-05 |
| ENSG00000129521 | EGLN3 | 2.1843858 | 5.8012278 | 3.616842 | 0.0014326 | 0.0148386 |
| ENSG00000271503 | CCL5 | 7.6496425 | 11.256153 | 3.6065106 | 5.35E-26 | 5.11E-23 |
| ENSG00000136237 | RAPGEF5 | 1.6360717 | 5.2366976 | 3.6006259 | 3.36E-05 | 0.0006622 |
| ENSG00000183762 | KREMEN1 | 4.7376973 | 8.3294867 | 3.5917895 | 1.97E-10 | 1.66E-08 |
| ENSG00000166920 | C15orf48 | 8.0905727 | 11.66725 | 3.5766771 | 5.73E-24 | 3.81E-21 |
| ENSG00000151948 | GLT1D1 | 5.3351917 | 8.9039896 | 3.5687979 | 5.99E-23 | 3.55E-20 |
| ENSG00000130413 | STK33 | 0.6965566 | 4.2541519 | 3.5575953 | 3.55E-06 | 9.61E-05 |
| ENSG00000259207 | ITGB3 | 7.5766154 | 11.118033 | 3.5414179 | 4.44E-26 | 4.33E-23 |
| ENSG00000105889 | STEAP1B | 5.0519644 | 8.5791612 | 3.5271968 | 2.72E-12 | 3.38E-10 |
| ENSG00000165272 | AQP3 | 3.0232089 | 6.5316131 | 3.5084042 | 6.77E-15 | 1.38E-12 |
| ENSG00000123610 | TNFAIP6 | 5.950434 | 9.4497902 | 3.4993562 | 1.65E-11 | 1.75E-09 |
| ENSG00000172724 | CCL19 | 0.2003816 | 3.6653327 | 3.4649511 | 6.60E-07 | 2.24E-05 |
| ENSG00000128274 | A4GALT | 2.3406535 | 5.7777706 | 3.4371172 | 0.0002123 | 0.0031456 |
| ENSG00000108821 | COL1A1 | 1.2541 | 4.6884916 | 3.4343916 | 7.75E-06 | 0.0001873 |
| ENSG00000115457 | IGFBP2 | 0.6877258 | 4.111063 | 3.4233372 | 0.0020537 | 0.0198921 |
| ENSG00000162433 | AK4 | 6.6023423 | 10.004791 | 3.4024484 | 2.29E-16 | 5.91E-14 |
| ENSG00000134460 | IL2RA | 5.871598 | 9.2508055 | 3.3792075 | 6.96E-11 | 6.64E-09 |
| ENSG00000138411 | HECW2 | 1.4261097 | 4.8002862 | 3.3741765 | 2.86E-10 | 2.35E-08 |
| ENSG00000185291 | IL3RA | 7.4964733 | 10.839949 | 3.3434753 | 6.39E-29 | 9.34E-26 |
| ENSG00000123405 | NFE2 | 0.4012458 | 3.7388145 | 3.3375687 | 0.0001913 | 0.0028798 |
| ENSG00000142512 | SIGLEC10 | 8.5040221 | 11.834018 | 3.329996 | 9.03E-13 | 1.25E-10 |
| ENSG00000182782 | HCAR2 | 1.4825565 | 4.810054 | 3.3274975 | 1.82E-11 | 1.92E-09 |
| ENSG00000150637 | CD226 | 4.9934207 | 8.3115051 | 3.3180844 | 1.16E-17 | 3.58E-15 |
| ENSG00000104921 | FCER2 | 5.8501565 | 9.1535878 | 3.3034313 | 5.56E-19 | 2.00E-16 |
| ENSG00000188056 | TREML4 | 1.3521723 | 4.6291778 | 3.2770055 | 3.05E-05 | 0.0006075 |
| ENSG00000169908 | TM4SF1 | 0.5348356 | 3.7867201 | 3.2518845 | 4.42E-06 | 0.0001158 |
| ENSG00000103196 | CRISPLD2 | 4.6108219 | 7.8616481 | 3.2508262 | 1.88E-10 | 1.60E-08 |
| ENSG00000229859 | PGA3 | 1.4966887 | 4.7400108 | 3.2433221 | 2.72E-07 | 1.04E-05 |
| ENSG00000275385 | CCL18 | 8.040764 | 11.258893 | 3.2181291 | 6.18E-32 | 1.18E-28 |
| ENSG00000163220 | S100A9 | 10.977834 | 14.160657 | 3.182823 | 3.54E-14 | 6.21E-12 |
| ENSG00000152952 | PLOD2 | 5.7249429 | 8.9027476 | 3.1778048 | 0.0008765 | 0.0100171 |
| ENSG00000101000 | PROCR | 6.3119768 | 9.4686018 | 3.156625 | 7.20E-33 | 1.50E-29 |
| ENSG00000213949 | ITGA1 | 3.0786855 | 6.2320267 | 3.1533412 | 9.06E-10 | 6.61E-08 |
| ENSG00000173918 | C1QTNF1 | 0.4202367 | 3.5661464 | 3.1459097 | 2.54E-07 | 9.76E-06 |
| ENSG00000130707 | ASS1 | 1.3225636 | 4.4657123 | 3.1431487 | 4.77E-06 | 0.0001235 |
| ENSG00000124785 | NRN1 | 0.4127511 | 3.5519739 | 3.1392228 | 1.72E-07 | 7.03E-06 |
| ENSG00000148483 | TMEM236 | 7.0142395 | 10.104501 | 3.0902617 | 3.27E-08 | 1.60E-06 |
| ENSG00000146592 | CREB5 | 6.2839352 | 9.3549966 | 3.0710614 | 8.07E-27 | 9.08E-24 |
| ENSG00000120162 | MOB3B | 8.6579958 | 11.725198 | 3.0672023 | 8.26E-54 | 1.21E-49 |
| ENSG00000172379 | ARNT2 | 0.3286554 | 3.3935355 | 3.0648802 | 0.0010244 | 0.0113162 |
| ENSG00000105835 | NAMPT | 10.951743 | 13.98154 | 3.029797 | 1.41E-16 | 3.71E-14 |
| ENSG00000196878 | LAMB3 | 7.5702964 | 10.590667 | 3.0203708 | 2.90E-25 | 2.40E-22 |
| ENSG00000120885 | CLU | 5.3241685 | 8.3230453 | 2.9988768 | 1.63E-08 | 8.60E-07 |
| ENSG00000173391 | OLR1 | 8.1377654 | 11.129299 | 2.9915331 | 7.42E-18 | 2.34E-15 |
| ENSG00000273003 | ARL2-SNX15 | 0.3589625 | 3.350069 | 2.9911065 | 0.0054852 | 0.0426292 |
| ENSG00000100116 | GCAT | 2.2390888 | 5.221166 | 2.9820772 | 0.0023644 | 0.0221585 |
| ENSG00000171051 | FPR1 | 9.049733 | 12.029792 | 2.9800591 | 6.06E-28 | 7.82E-25 |
| ENSG00000178726 | THBD | 5.2879947 | 8.2645482 | 2.9765535 | 2.81E-15 | 6.26E-13 |
| ENSG00000133317 | LGALS12 | 1.2284339 | 4.184443 | 2.9560091 | 0.0003109 | 0.004302 |
| ENSG00000140563 | MCTP2 | 4.7493605 | 7.7045384 | 2.955178 | 3.18E-11 | 3.18E-09 |
| ENSG00000171033 | PKIA | 2.1153829 | 5.0659347 | 2.9505518 | 1.72E-05 | 0.0003713 |
| ENSG00000111424 | VDR | 9.4652447 | 12.398765 | 2.9335203 | 5.22E-53 | 5.72E-49 |
| ENSG00000157064 | NMNAT2 | 1.6096505 | 4.5424864 | 2.932836 | 5.80E-06 | 0.0001457 |
| ENSG00000153707 | PTPRD | 0.4574089 | 3.3901507 | 2.9327418 | 5.47E-07 | 1.91E-05 |
| ENSG00000111913 | RIPOR2 | 1.9293874 | 4.856844 | 2.9274566 | 0.0001348 | 0.0021418 |
| ENSG00000099985 | OSM | 5.5946183 | 8.5063564 | 2.9117381 | 7.87E-19 | 2.76E-16 |
| ENSG00000078295 | ADCY2 | 0 | 2.9060012 | 2.9060012 | 1.81E-09 | 1.21E-07 |
| ENSG00000127954 | STEAP4 | 3.7822584 | 6.6809456 | 2.8986871 | 5.38E-05 | 0.0009877 |
| ENSG00000165757 | JCAD | 0.6943869 | 3.5870045 | 2.8926176 | 7.07E-06 | 0.0001736 |
| ENSG00000121807 | CCR2 | 4.2665581 | 7.1523227 | 2.8857646 | 2.32E-06 | 6.66E-05 |
| ENSG00000125430 | HS3ST3B1 | 7.0841387 | 9.9580281 | 2.8738893 | 8.56E-11 | 8.03E-09 |
| ENSG00000182566 | CLEC4G | 2.8871393 | 5.7463348 | 2.8591955 | 1.76E-08 | 9.26E-07 |
| ENSG00000197632 | SERPINB2 | 5.1097011 | 7.9676903 | 2.8579892 | 4.08E-05 | 0.0007794 |
| ENSG00000118473 | SGIP1 | 0.2535251 | 3.101596 | 2.8480709 | 1.38E-06 | 4.21E-05 |
| ENSG00000136696 | IL36B | 1.4435117 | 4.2892239 | 2.8457122 | 0.0001201 | 0.0019469 |
| ENSG00000120160 | EQTN | 0.998126 | 3.8427702 | 2.8446443 | 7.56E-06 | 0.0001836 |
| ENSG00000184838 | PRR16 | 2.1669527 | 5.0018681 | 2.8349155 | 0.0015112 | 0.0155137 |
| ENSG00000151136 | BTBD11 | 3.8034425 | 6.6286055 | 2.825163 | 6.00E-08 | 2.77E-06 |
| ENSG00000169429 | CXCL8 | 12.452298 | 15.262353 | 2.8100544 | 8.02E-06 | 0.0001929 |
| ENSG00000087510 | TFAP2C | 3.0034533 | 5.8127177 | 2.8092644 | 2.76E-15 | 6.21E-13 |
| ENSG00000103569 | AQP9 | 11.069379 | 13.876003 | 2.8066238 | 1.53E-21 | 7.45E-19 |
| ENSG00000125810 | CD93 | 6.7838311 | 9.588941 | 2.8051099 | 1.19E-08 | 6.51E-07 |
| ENSG00000105374 | NKG7 | 1.8891977 | 4.6897373 | 2.8005396 | 1.20E-07 | 5.15E-06 |
| ENSG00000135426 | TESPA1 | 0.867326 | 3.6645702 | 2.7972442 | 0.0033075 | 0.0287723 |
| ENSG00000076554 | TPD52 | 4.3493334 | 7.1337126 | 2.7843792 | 8.02E-09 | 4.55E-07 |
| ENSG00000275688 | CCL15-CCL14 | 0.2901009 | 3.0673525 | 2.7772515 | 9.50E-06 | 0.0002237 |
| ENSG00000163568 | AIM2 | 2.5110371 | 5.280147 | 2.7691099 | 8.04E-07 | 2.66E-05 |
| ENSG00000135111 | TBX3 | 1.2346863 | 3.997782 | 2.7630957 | 0.0050689 | 0.0401045 |
| ENSG00000005448 | WDR54 | 1.9677519 | 4.7269093 | 2.7591573 | 0.0001866 | 0.0028196 |
| ENSG00000163421 | PROK2 | 2.8045418 | 5.5415317 | 2.7369899 | 5.76E-05 | 0.0010458 |
| ENSG00000173597 | SULT1B1 | 2.2081693 | 4.9403619 | 2.7321926 | 0.0008686 | 0.0099404 |
| ENSG00000188766 | SPRED3 | 1.8726996 | 4.6040449 | 2.7313453 | 0.0007802 | 0.0091265 |
| ENSG00000147724 | FAM135B | 1.6012226 | 4.3309678 | 2.7297452 | 0.0003687 | 0.0049632 |
| ENSG00000159403 | C1R | 2.279916 | 4.9955188 | 2.7156028 | 0.0010173 | 0.0112559 |
| ENSG00000185499 | MUC1 | 2.8658888 | 5.5773661 | 2.7114774 | 9.77E-10 | 7.07E-08 |
| ENSG00000160179 | ABCG1 | 8.0262743 | 10.737514 | 2.7112392 | 1.81E-10 | 1.56E-08 |
| ENSG00000018280 | SLC11A1 | 10.506361 | 13.214056 | 2.7076954 | 5.76E-20 | 2.34E-17 |
| ENSG00000185033 | SEMA4B | 6.7653637 | 9.4727189 | 2.7073552 | 1.35E-21 | 6.74E-19 |
| ENSG00000197769 | MAP1LC3C | 1.1537244 | 3.8517381 | 2.6980137 | 0.00011 | 0.0018115 |
| ENSG00000119681 | LTBP2 | 8.0699654 | 10.766631 | 2.6966658 | 9.08E-12 | 1.01E-09 |
| ENSG00000115828 | QPCT | 8.9184359 | 11.609806 | 2.69137 | 1.85E-23 | 1.14E-20 |
| ENSG00000129682 | FGF13 | 0.2082275 | 2.8929685 | 2.684741 | 1.80E-06 | 5.36E-05 |
| ENSG00000186648 | CARMIL3 | 6.7099268 | 9.3748657 | 2.6649389 | 1.45E-17 | 4.41E-15 |
| ENSG00000104415 | WISP1 | 1.0062073 | 3.6631602 | 2.6569529 | 0.0004284 | 0.0055952 |
| ENSG00000139572 | GPR84 | 8.8753322 | 11.532262 | 2.6569301 | 2.06E-19 | 7.78E-17 |
| ENSG00000167617 | CDC42EP5 | 0.9184858 | 3.5689974 | 2.6505116 | 2.54E-06 | 7.20E-05 |
| ENSG00000276409 | CCL14 | 0.2721769 | 2.9177253 | 2.6455484 | 3.89E-05 | 0.0007489 |
| ENSG00000260903 | XKR7 | 3.2163778 | 5.8607777 | 2.6443998 | 0.0003117 | 0.0043106 |
| ENSG00000169385 | RNASE2 | 4.0985626 | 6.7390642 | 2.6405016 | 4.89E-11 | 4.77E-09 |
| ENSG00000111012 | CYP27B1 | 6.3752961 | 9.0101135 | 2.6348174 | 2.47E-08 | 1.26E-06 |
| ENSG00000168421 | RHOH | 2.0326842 | 4.6648365 | 2.6321523 | 4.08E-05 | 0.0007794 |
| ENSG00000205362 | MT1A | 0 | 2.6243608 | 2.6243608 | 0.0003851 | 0.0051446 |
| ENSG00000248099 | INSL3 | 0 | 2.6191996 | 2.6191996 | 1.45E-07 | 6.07E-06 |
| ENSG00000181374 | CCL13 | 6.4178951 | 9.0274243 | 2.6095291 | 1.04E-08 | 5.78E-07 |
| ENSG00000134954 | ETS1 | 5.130429 | 7.7389005 | 2.6084715 | 1.23E-12 | 1.64E-10 |
| ENSG00000104341 | LAPTM4B | 4.4240074 | 7.0202279 | 2.5962205 | 8.31E-16 | 2.02E-13 |
| ENSG00000137801 | THBS1 | 7.6880439 | 10.278785 | 2.5907413 | 2.58E-08 | 1.31E-06 |
| ENSG00000163737 | PF4 | 0 | 2.5843819 | 2.5843819 | 2.04E-07 | 8.11E-06 |
| ENSG00000159618 | ADGRG5 | 0.2831896 | 2.8645844 | 2.5813948 | 0.0027209 | 0.0247896 |
| ENSG00000152760 | TCTEX1D1 | 3.956652 | 6.5363838 | 2.5797318 | 4.44E-08 | 2.11E-06 |
| ENSG00000143847 | PPFIA4 | 4.1102579 | 6.6869625 | 2.5767047 | 4.11E-06 | 0.0001085 |
| ENSG00000108700 | CCL8 | 7.571974 | 10.145028 | 2.573054 | 0.0033009 | 0.0287263 |
| ENSG00000101188 | NTSR1 | 0.3078509 | 2.8740222 | 2.5661712 | 9.17E-05 | 0.0015511 |
| ENSG00000139053 | PDE6H | 1.5620503 | 4.1128979 | 2.5508475 | 1.92E-07 | 7.73E-06 |
| ENSG00000198753 | PLXNB3 | 2.1552413 | 4.7023089 | 2.5470677 | 2.71E-09 | 1.72E-07 |
| ENSG00000162878 | PKDCC | 0.8843957 | 3.4311692 | 2.5467735 | 0.0023126 | 0.0217566 |
| ENSG00000185052 | SLC24A3 | 4.2218386 | 6.7651343 | 2.5432957 | 1.54E-11 | 1.64E-09 |
| ENSG00000104974 | LILRA1 | 7.221721 | 9.7602617 | 2.5385407 | 2.56E-12 | 3.21E-10 |
| ENSG00000164683 | HEY1 | 0.6087463 | 3.1334845 | 2.5247382 | 1.24E-05 | 0.0002802 |
| ENSG00000157168 | NRG1 | 0.3628293 | 2.8829867 | 2.5201573 | 0.003539 | 0.0302704 |
| ENSG00000256660 | CLEC12B | 2.4596333 | 4.9700384 | 2.5104052 | 7.69E-07 | 2.56E-05 |
| ENSG00000105352 | CEACAM4 | 3.8002347 | 6.2966837 | 2.496449 | 7.53E-09 | 4.29E-07 |
| ENSG00000024422 | EHD2 | 1.1047517 | 3.5889179 | 2.4841662 | 0.0002757 | 0.0039038 |
| ENSG00000162745 | OLFML2B | 10.915233 | 13.385311 | 2.470078 | 1.12E-12 | 1.52E-10 |
| ENSG00000229183 | PGA4 | 2.2163908 | 4.6802554 | 2.4638646 | 4.01E-06 | 0.0001064 |
| ENSG00000182580 | EPHB3 | 0.6907847 | 3.1424146 | 2.4516299 | 0.0049361 | 0.0392871 |
| ENSG00000143797 | MBOAT2 | 5.7204663 | 8.1628921 | 2.4424258 | 8.09E-10 | 6.04E-08 |
| ENSG00000059804 | SLC2A3 | 10.082689 | 12.523093 | 2.440404 | 7.26E-23 | 4.19E-20 |
| ENSG00000170837 | GPR27 | 3.8341864 | 6.2745552 | 2.4403688 | 0.0019285 | 0.0189131 |
| ENSG00000109321 | AREG | 0.7549677 | 3.193146 | 2.4381783 | 0.0002675 | 0.0038102 |
| ENSG00000004468 | CD38 | 9.0207639 | 11.457032 | 2.4362681 | 1.39E-15 | 3.27E-13 |
| ENSG00000104154 | SLC30A4 | 8.0414985 | 10.475965 | 2.4344663 | 1.42E-16 | 3.72E-14 |
| ENSG00000119915 | ELOVL3 | 0.7015854 | 3.1344534 | 2.4328681 | 8.37E-05 | 0.0014387 |
| ENSG00000130396 | AFDN | 9.081792 | 11.501349 | 2.4195569 | 6.00E-29 | 9.08E-26 |
| ENSG00000127530 | OR7C1 | 1.5682 | 3.9870375 | 2.4188375 | 0.0008085 | 0.0093913 |
| ENSG00000162747 | FCGR3B | 4.259976 | 6.6779694 | 2.4179934 | 7.93E-08 | 3.53E-06 |
| ENSG00000128918 | ALDH1A2 | 8.1504427 | 10.564997 | 2.4145541 | 4.84E-06 | 0.0001249 |
| ENSG00000186431 | FCAR | 8.1414419 | 10.541645 | 2.4002035 | 1.53E-11 | 1.64E-09 |
| ENSG00000113749 | HRH2 | 9.0952128 | 11.493195 | 2.397982 | 1.86E-11 | 1.95E-09 |
| ENSG00000072952 | MRVI1 | 3.0596203 | 5.454539 | 2.3949187 | 0.0001903 | 0.0028663 |
| ENSG00000175130 | MARCKSL1 | 5.4929935 | 7.874993 | 2.3819994 | 1.06E-14 | 2.10E-12 |
| ENSG00000090659 | CD209 | 9.9919283 | 12.373175 | 2.3812471 | 5.22E-31 | 9.55E-28 |
| ENSG00000166523 | CLEC4E | 9.0133946 | 11.390045 | 2.3766502 | 4.75E-09 | 2.84E-07 |
| ENSG00000159166 | LAD1 | 0.1955338 | 2.5676154 | 2.3720816 | 9.07E-06 | 0.0002152 |
| ENSG00000160963 | COL26A1 | 0.6014948 | 2.966619 | 2.3651242 | 0.0036657 | 0.0311169 |
| ENSG00000176105 | YES1 | 4.8904095 | 7.2462506 | 2.3558412 | 3.34E-09 | 2.08E-07 |
| ENSG00000114737 | CISH | 7.7839497 | 10.132996 | 2.3490465 | 1.54E-24 | 1.13E-21 |
| ENSG00000019169 | MARCO | 11.032534 | 13.380534 | 2.3480003 | 2.88E-08 | 1.44E-06 |
| ENSG00000183625 | CCR3 | 0.8156928 | 3.1606661 | 2.3449732 | 0.002084 | 0.0201145 |
| ENSG00000106366 | SERPINE1 | 7.1575217 | 9.4970837 | 2.339562 | 4.57E-09 | 2.75E-07 |
| ENSG00000185897 | FFAR3 | 0.1014604 | 2.4370167 | 2.3355564 | 2.79E-07 | 1.06E-05 |
| ENSG00000165795 | NDRG2 | 5.5411241 | 7.8714914 | 2.3303672 | 1.24E-07 | 5.27E-06 |
| ENSG00000197208 | SLC22A4 | 5.6648958 | 7.990224 | 2.3253282 | 4.09E-20 | 1.68E-17 |
| ENSG00000064886 | CHI3L2 | 5.893866 | 8.2104217 | 2.3165558 | 7.15E-12 | 8.17E-10 |
| ENSG00000244405 | ETV5 | 9.7521597 | 12.057591 | 2.3054311 | 1.31E-10 | 1.16E-08 |
| ENSG00000132623 | ANKEF1 | 5.0468491 | 7.3514997 | 2.3046506 | 2.36E-09 | 1.52E-07 |
| ENSG00000104972 | LILRB1 | 10.598929 | 12.894572 | 2.2956429 | 4.07E-15 | 8.83E-13 |
| ENSG00000164181 | ELOVL7 | 4.0783725 | 6.3633599 | 2.2849874 | 1.57E-09 | 1.07E-07 |
| ENSG00000168209 | DDIT4 | 7.0303239 | 9.299817 | 2.2694931 | 2.73E-11 | 2.79E-09 |
| ENSG00000258083 | OR9A4 | 1.0372317 | 3.3064776 | 2.2692459 | 4.44E-05 | 0.0008374 |
| ENSG00000256713 | PGA5 | 2.2139156 | 4.4818164 | 2.2679008 | 0.0001352 | 0.0021469 |
| ENSG00000105048 | TNNT1 | 3.8132386 | 6.0714805 | 2.2582419 | 0.0002562 | 0.0036877 |
| ENSG00000123096 | SSPN | 4.9431481 | 7.1943031 | 2.251155 | 2.26E-05 | 0.0004694 |
| ENSG00000188215 | DCUN1D3 | 8.8858891 | 11.126908 | 2.241019 | 6.14E-27 | 7.49E-24 |
| ENSG00000117394 | SLC2A1 | 6.5064883 | 8.7470138 | 2.2405255 | 3.06E-11 | 3.07E-09 |
| ENSG00000223923 | AC010136.1 | 0.1970582 | 2.4260299 | 2.2289717 | 2.34E-05 | 0.0004836 |
| ENSG00000203747 | FCGR3A | 11.200822 | 13.421835 | 2.2210128 | 2.41E-11 | 2.50E-09 |
| ENSG00000137331 | IER3 | 10.553518 | 12.769657 | 2.2161391 | 8.20E-05 | 0.0014112 |
| ENSG00000143217 | NECTIN4 | 7.0179453 | 9.2280659 | 2.2101207 | 4.61E-09 | 2.76E-07 |
| ENSG00000118193 | KIF14 | 3.8622551 | 6.0714669 | 2.2092118 | 4.47E-07 | 1.59E-05 |
| ENSG00000124491 | F13A1 | 7.1046912 | 9.312453 | 2.2077619 | 6.68E-06 | 0.0001649 |
| ENSG00000153064 | BANK1 | 3.8918655 | 6.0945622 | 2.2026967 | 8.62E-07 | 2.82E-05 |
| ENSG00000174705 | SH3PXD2B | 11.013992 | 13.211126 | 2.1971337 | 6.41E-16 | 1.57E-13 |
| ENSG00000187583 | PLEKHN1 | 2.0667689 | 4.2552404 | 2.1884715 | 0.0002474 | 0.0035772 |
| ENSG00000171517 | LPAR3 | 2.1760718 | 4.3602691 | 2.1841973 | 8.53E-05 | 0.0014587 |
| ENSG00000169116 | PARM1 | 1.5571358 | 3.7355077 | 2.1783719 | 0.0001436 | 0.0022636 |
| ENSG00000164023 | SGMS2 | 8.4414603 | 10.61936 | 2.1778995 | 6.95E-27 | 8.02E-24 |
| ENSG00000182541 | LIMK2 | 9.528499 | 11.695046 | 2.1665471 | 1.32E-25 | 1.16E-22 |
| ENSG00000073756 | PTGS2 | 6.6962713 | 8.8624819 | 2.1662105 | 1.35E-05 | 0.0003017 |
| ENSG00000103522 | IL21R | 6.7073874 | 8.8610807 | 2.1536933 | 2.39E-07 | 9.28E-06 |
| ENSG00000175040 | CHST2 | 8.5427785 | 10.681055 | 2.1382761 | 0.0003319 | 0.0045521 |
| ENSG00000075426 | FOSL2 | 10.210256 | 12.344629 | 2.1343727 | 1.21E-14 | 2.36E-12 |
| ENSG00000154262 | ABCA6 | 3.5869769 | 5.7204896 | 2.1335127 | 0.0056677 | 0.043638 |
| ENSG00000124942 | AHNAK | 14.548919 | 16.678841 | 2.1299214 | 0.0065932 | 0.0490686 |
| ENSG00000050030 | NEXMIF | 0.2676096 | 2.3912459 | 2.1236363 | 0.0006859 | 0.008234 |
| ENSG00000127507 | ADGRE2 | 11.058435 | 13.175099 | 2.1166641 | 6.54E-19 | 2.31E-16 |
| ENSG00000151012 | SLC7A11 | 11.914322 | 14.030684 | 2.1163616 | 1.31E-07 | 5.52E-06 |
| ENSG00000138772 | ANXA3 | 0.2552238 | 2.3667467 | 2.1115229 | 0.0010286 | 0.0113513 |
| ENSG00000163710 | PCOLCE2 | 4.8026122 | 6.9110219 | 2.1084097 | 2.59E-08 | 1.31E-06 |
| ENSG00000148143 | ZNF462 | 6.1240169 | 8.2217902 | 2.0977733 | 3.74E-09 | 2.31E-07 |
| ENSG00000029153 | ARNTL2 | 7.9795526 | 10.070438 | 2.0908856 | 5.05E-05 | 0.0009331 |
| ENSG00000185885 | IFITM1 | 1.2800013 | 3.3682351 | 2.0882337 | 0.0003955 | 0.0052524 |
| ENSG00000041982 | TNC | 9.0952794 | 11.182846 | 2.0875664 | 2.30E-05 | 0.0004769 |
| ENSG00000172322 | CLEC12A | 7.6718178 | 9.7425341 | 2.0707163 | 1.58E-12 | 2.08E-10 |
| ENSG00000182326 | C1S | 5.9112685 | 7.9794687 | 2.0682002 | 6.52E-06 | 0.0001618 |
| ENSG00000149328 | GLB1L2 | 0.1970582 | 2.2587383 | 2.0616801 | 6.98E-05 | 0.001233 |
| ENSG00000132003 | ZSWIM4 | 7.3371613 | 9.3945336 | 2.0573722 | 5.24E-08 | 2.45E-06 |
| ENSG00000171657 | GPR82 | 6.5590733 | 8.6163791 | 2.0573058 | 2.20E-07 | 8.65E-06 |
| ENSG00000136052 | SLC41A2 | 9.31636 | 11.365994 | 2.0496336 | 1.30E-19 | 5.04E-17 |
| ENSG00000145428 | RNF175 | 6.2100583 | 8.2540646 | 2.0440063 | 2.24E-08 | 1.15E-06 |
| ENSG00000172594 | SMPDL3A | 7.9219534 | 9.9546133 | 2.0326599 | 1.04E-13 | 1.70E-11 |
| ENSG00000163734 | CXCL3 | 8.8350525 | 10.859732 | 2.0246793 | 1.49E-08 | 7.96E-07 |
| ENSG00000133816 | MICAL2 | 9.8168127 | 11.829039 | 2.0122263 | 5.04E-13 | 7.35E-11 |
| ENSG00000118113 | MMP8 | 7.4963932 | 9.5079798 | 2.0115865 | 0.0035741 | 0.0305283 |
| ENSG00000257335 | MGAM | 6.3171022 | 8.3182089 | 2.0011067 | 1.12E-16 | 2.99E-14 |
| ENSG00000123685 | BATF3 | 3.4957816 | 5.4964028 | 2.0006213 | 0.0001354 | 0.0021496 |
| ENSG00000196083 | IL1RAP | 8.0699873 | 10.064989 | 1.9950022 | 5.24E-24 | 3.59E-21 |
| ENSG00000139610 | CELA1 | 0.6479086 | 2.6375354 | 1.9896268 | 0.0008503 | 0.0097582 |
| ENSG00000100558 | PLEK2 | 4.4718707 | 6.4596718 | 1.9878011 | 4.34E-15 | 9.34E-13 |
| ENSG00000166068 | SPRED1 | 11.573151 | 13.557975 | 1.9848245 | 2.50E-17 | 7.31E-15 |
| ENSG00000170153 | RNF150 | 0.6981503 | 2.6829375 | 1.9847872 | 0.000811 | 0.009408 |
| ENSG00000183185 | GABRR3 | 0 | 1.9843696 | 1.9843696 | 9.94E-06 | 0.0002327 |
| ENSG00000102445 | RUBCNL | 8.1132508 | 10.097502 | 1.9842508 | 3.21E-08 | 1.58E-06 |
| ENSG00000058085 | LAMC2 | 0.716987 | 2.6946289 | 1.9776419 | 0.0025417 | 0.0234491 |
| ENSG00000108691 | CCL2 | 13.9201 | 15.895398 | 1.9752977 | 1.26E-12 | 1.69E-10 |
| ENSG00000115009 | CCL20 | 3.3731553 | 5.3479423 | 1.974787 | 0.00014 | 0.0022142 |
| ENSG00000169083 | AR | 3.0880555 | 5.048247 | 1.9601915 | 5.29E-07 | 1.85E-05 |
| ENSG00000174807 | CD248 | 0.9628512 | 2.9189979 | 1.9561467 | 0.0020686 | 0.0200009 |
| ENSG00000128641 | MYO1B | 5.2662052 | 7.2151383 | 1.9489331 | 0.000451 | 0.0058418 |
| ENSG00000170379 | TCAF2 | 5.0741104 | 7.0218981 | 1.9477877 | 2.08E-09 | 1.37E-07 |
| ENSG00000101916 | TLR8 | 11.194142 | 13.132766 | 1.9386235 | 2.57E-19 | 9.64E-17 |
| ENSG00000143226 | FCGR2A | 12.017485 | 13.955398 | 1.9379132 | 7.42E-12 | 8.42E-10 |
| ENSG00000125910 | S1PR4 | 1.1455038 | 3.0825599 | 1.9370561 | 0.0003692 | 0.0049689 |
| ENSG00000198814 | GK | 10.985854 | 12.918389 | 1.9325347 | 2.16E-13 | 3.28E-11 |
| ENSG00000169439 | SDC2 | 10.457519 | 12.375496 | 1.9179773 | 1.33E-10 | 1.17E-08 |
| ENSG00000113657 | DPYSL3 | 7.4910535 | 9.4086542 | 1.9176007 | 3.11E-06 | 8.57E-05 |
| ENSG00000148482 | SLC39A12 | 3.4479589 | 5.3619697 | 1.9140108 | 1.11E-05 | 0.0002549 |
| ENSG00000170542 | SERPINB9 | 9.5158599 | 11.422411 | 1.9065515 | 9.91E-19 | 3.40E-16 |
| ENSG00000260314 | MRC1 | 14.887739 | 16.790942 | 1.903203 | 1.75E-13 | 2.71E-11 |
| ENSG00000149798 | CDC42EP2 | 4.1365534 | 6.0392592 | 1.9027058 | 0.000453 | 0.0058603 |
| ENSG00000014914 | MTMR11 | 2.2183066 | 4.1162311 | 1.8979246 | 0.0017977 | 0.0178416 |
| ENSG00000184221 | OLIG1 | 2.9355433 | 4.8262693 | 1.8907259 | 8.69E-06 | 0.000207 |
| ENSG00000101460 | MAP1LC3A | 4.69105 | 6.5774236 | 1.8863736 | 0.0006056 | 0.0074306 |
| ENSG00000016391 | CHDH | 6.1438005 | 8.0283032 | 1.8845027 | 3.61E-09 | 2.23E-07 |
| ENSG00000026508 | CD44 | 13.341418 | 15.219927 | 1.8785085 | 6.47E-15 | 1.32E-12 |
| ENSG00000275126 | HIST1H4L | 4.3945646 | 6.2656557 | 1.8710912 | 4.30E-06 | 0.000113 |
| ENSG00000143924 | EML4 | 11.834067 | 13.702511 | 1.8684445 | 1.75E-21 | 8.36E-19 |
| ENSG00000057704 | TMCC3 | 8.206428 | 10.071756 | 1.8653285 | 8.23E-07 | 2.72E-05 |
| ENSG00000108950 | FAM20A | 8.3689123 | 10.222152 | 1.8532398 | 4.62E-10 | 3.63E-08 |
| ENSG00000131095 | GFAP | 2.6557243 | 4.5088179 | 1.8530936 | 0.0002854 | 0.0040134 |
| ENSG00000119326 | CTNNAL1 | 5.8451544 | 7.6963681 | 1.8512137 | 1.39E-07 | 5.83E-06 |
| ENSG00000152207 | CYSLTR2 | 5.5027913 | 7.3538927 | 1.8511014 | 1.45E-05 | 0.0003212 |
| ENSG00000152229 | PSTPIP2 | 10.454522 | 12.299596 | 1.8450746 | 1.37E-18 | 4.59E-16 |
| ENSG00000099139 | PCSK5 | 9.2410382 | 11.085024 | 1.843986 | 7.05E-18 | 2.24E-15 |
| ENSG00000141068 | KSR1 | 8.3103139 | 10.151994 | 1.8416803 | 9.02E-24 | 5.82E-21 |
| ENSG00000138166 | DUSP5 | 6.6678416 | 8.5013594 | 1.8335178 | 0.0005391 | 0.0067678 |
| ENSG00000169432 | SCN9A | 4.8525673 | 6.6857603 | 1.833193 | 0.0001217 | 0.0019646 |
| ENSG00000113070 | HBEGF | 8.7526027 | 10.582321 | 1.8297182 | 1.02E-09 | 7.32E-08 |
| ENSG00000005486 | RHBDD2 | 8.1837269 | 10.012021 | 1.8282945 | 1.48E-13 | 2.35E-11 |
| ENSG00000134333 | LDHA | 12.224241 | 14.051247 | 1.8270063 | 5.57E-13 | 8.07E-11 |
| ENSG00000152784 | PRDM8 | 2.9197439 | 4.7424121 | 1.8226682 | 6.86E-05 | 0.0012152 |
| ENSG00000275302 | CCL4 | 9.7743009 | 11.596118 | 1.8218171 | 2.14E-05 | 0.000451 |
| ENSG00000133048 | CHI3L1 | 13.592237 | 15.396603 | 1.8043652 | 1.64E-06 | 4.92E-05 |
| ENSG00000179299 | NSUN7 | 3.0112076 | 4.8145876 | 1.80338 | 0.0052031 | 0.0409008 |
| ENSG00000198734 | F5 | 3.0965583 | 4.8968839 | 1.8003257 | 0.0004119 | 0.0054176 |
| ENSG00000100985 | MMP9 | 15.685478 | 17.484796 | 1.7993175 | 0.001383 | 0.014417 |
| ENSG00000154229 | PRKCA | 10.19911 | 11.995612 | 1.7965023 | 3.81E-09 | 2.33E-07 |
| ENSG00000102359 | SRPX2 | 4.4023775 | 6.1948868 | 1.7925093 | 1.74E-07 | 7.10E-06 |
| ENSG00000162493 | PDPN | 8.6314024 | 10.421374 | 1.7899715 | 5.07E-06 | 0.0001298 |
| ENSG00000156265 | MAP3K7CL | 5.3667415 | 7.1544102 | 1.7876686 | 1.82E-08 | 9.48E-07 |
| ENSG00000170522 | ELOVL6 | 3.979986 | 5.7652694 | 1.7852834 | 2.59E-06 | 7.33E-05 |
| ENSG00000140450 | ARRDC4 | 9.4389921 | 11.222973 | 1.7839807 | 4.89E-19 | 1.78E-16 |
| ENSG00000147036 | LANCL3 | 4.0093498 | 5.7910503 | 1.7817006 | 7.84E-05 | 0.0013616 |
| ENSG00000165682 | CLEC1B | 4.1290515 | 5.9074059 | 1.7783544 | 6.38E-06 | 0.0001589 |
| ENSG00000158258 | CLSTN2 | 0.9282261 | 2.7021678 | 1.7739417 | 0.0043792 | 0.0358564 |
| ENSG00000072694 | FCGR2B | 11.486244 | 13.256079 | 1.7698344 | 9.55E-08 | 4.18E-06 |
| ENSG00000012779 | ALOX5 | 9.5577092 | 11.32563 | 1.7679212 | 1.86E-11 | 1.95E-09 |
| ENSG00000107485 | GATA3 | 0 | 1.7630978 | 1.7630978 | 0.0011607 | 0.0125374 |
| ENSG00000265972 | TXNIP | 11.098738 | 12.856964 | 1.7582264 | 6.76E-08 | 3.08E-06 |
| ENSG00000135480 | KRT7 | 1.5605057 | 3.3187269 | 1.7582213 | 0.0064374 | 0.0482033 |
| ENSG00000196781 | TLE1 | 6.4299562 | 8.1877748 | 1.7578187 | 1.67E-06 | 5.01E-05 |
| ENSG00000164038 | SLC9B2 | 2.5267585 | 4.2844184 | 1.7576599 | 0.0006476 | 0.0078256 |
| ENSG00000168546 | GFRA2 | 4.8627017 | 6.6160109 | 1.7533092 | 0.0028666 | 0.0258275 |
| ENSG00000136830 | FAM129B | 11.503191 | 13.248278 | 1.7450869 | 1.44E-10 | 1.26E-08 |
| ENSG00000152256 | PDK1 | 9.1436357 | 10.888643 | 1.7450073 | 1.95E-07 | 7.82E-06 |
| ENSG00000169607 | CKAP2L | 2.3802872 | 4.1244589 | 1.7441717 | 0.0010785 | 0.0118036 |
| ENSG00000167077 | MEI1 | 6.5400483 | 8.2835146 | 1.7434663 | 7.73E-08 | 3.47E-06 |
| ENSG00000088826 | SMOX | 7.8289793 | 9.5690475 | 1.7400682 | 2.71E-12 | 3.38E-10 |
| ENSG00000166922 | SCG5 | 3.6913524 | 5.4309893 | 1.7396369 | 0.0001636 | 0.0025284 |
| ENSG00000147145 | LPAR4 | 0.420597 | 2.1489481 | 1.7283511 | 0.0029478 | 0.0263912 |
| ENSG00000188313 | PLSCR1 | 9.8919762 | 11.619759 | 1.7277824 | 2.10E-16 | 5.46E-14 |
| ENSG00000124257 | NEURL2 | 2.2946724 | 4.0198128 | 1.7251404 | 0.0008263 | 0.0095462 |
| ENSG00000179542 | SLITRK4 | 5.4311576 | 7.1455044 | 1.7143468 | 0.0001135 | 0.0018591 |
| ENSG00000197982 | C1orf122 | 8.182604 | 9.8906176 | 1.7080135 | 1.30E-11 | 1.41E-09 |
| ENSG00000139567 | ACVRL1 | 8.1909057 | 9.8982507 | 1.707345 | 1.60E-05 | 0.0003501 |
| ENSG00000138080 | EMILIN1 | 7.7733724 | 9.4757419 | 1.7023695 | 0.0022517 | 0.0213669 |
| ENSG00000006468 | ETV1 | 0.2721769 | 1.9698809 | 1.697704 | 0.0064336 | 0.0481923 |
| ENSG00000161640 | SIGLEC11 | 6.2606549 | 7.9574937 | 1.6968388 | 0.0005961 | 0.0073514 |
| ENSG00000198483 | ANKRD35 | 1.6560006 | 3.3518414 | 1.6958408 | 0.0065615 | 0.0488737 |
| ENSG00000173281 | PPP1R3B | 7.7639261 | 9.4597325 | 1.6958064 | 4.13E-14 | 7.19E-12 |
| ENSG00000072274 | TFRC | 13.321058 | 15.014886 | 1.6938274 | 2.14E-10 | 1.79E-08 |
| ENSG00000103257 | SLC7A5 | 6.8939574 | 8.5843397 | 1.6903823 | 1.55E-06 | 4.70E-05 |
| ENSG00000139318 | DUSP6 | 10.382872 | 12.070172 | 1.6873005 | 7.12E-06 | 0.0001746 |
| ENSG00000276070 | CCL4L2 | 8.2394948 | 9.919848 | 1.6803532 | 0.00103 | 0.0113633 |
| ENSG00000140022 | STON2 | 6.1206169 | 7.798164 | 1.6775471 | 4.06E-05 | 0.0007778 |
| ENSG00000013619 | MAMLD1 | 5.2878755 | 6.9639194 | 1.676044 | 9.25E-06 | 0.0002187 |
| ENSG00000183023 | SLC8A1 | 11.968216 | 13.637224 | 1.6690074 | 3.73E-15 | 8.14E-13 |
| ENSG00000197646 | PDCD1LG2 | 9.0384463 | 10.707313 | 1.6688667 | 3.94E-17 | 1.12E-14 |
| ENSG00000070729 | CNGB1 | 2.8907064 | 4.5591671 | 1.6684607 | 2.39E-09 | 1.54E-07 |
| ENSG00000111817 | DSE | 10.973102 | 12.639388 | 1.666286 | 1.99E-11 | 2.08E-09 |
| ENSG00000136286 | MYO1G | 10.587569 | 12.253049 | 1.6654798 | 2.45E-14 | 4.43E-12 |
| ENSG00000002587 | HS3ST1 | 8.5448505 | 10.207172 | 1.6623217 | 3.71E-10 | 2.98E-08 |
| ENSG00000120318 | ARAP3 | 7.5882589 | 9.2461311 | 1.6578721 | 1.18E-05 | 0.0002688 |
| ENSG00000119686 | FLVCR2 | 9.3800745 | 11.023566 | 1.6434914 | 2.14E-12 | 2.74E-10 |
| ENSG00000205927 | OLIG2 | 0.8854291 | 2.5281958 | 1.6427667 | 0.0050971 | 0.0402549 |
| ENSG00000143382 | ADAMTSL4 | 10.73083 | 12.370059 | 1.6392287 | 4.05E-17 | 1.14E-14 |
| ENSG00000104112 | SCG3 | 3.9308798 | 5.5674068 | 1.636527 | 4.32E-07 | 1.55E-05 |
| ENSG00000112303 | VNN2 | 9.1593564 | 10.793333 | 1.6339769 | 5.45E-06 | 0.0001379 |
| ENSG00000172817 | CYP7B1 | 5.189211 | 6.8228308 | 1.6336198 | 6.82E-05 | 0.0012107 |
| ENSG00000179776 | CDH5 | 0.1970582 | 1.8285316 | 1.6314734 | 0.0042326 | 0.0348969 |
| ENSG00000213889 | PPM1N | 5.39847 | 7.0252162 | 1.6267462 | 4.91E-05 | 0.0009127 |
| ENSG00000114251 | WNT5A | 6.9864143 | 8.6120313 | 1.625617 | 1.51E-07 | 6.29E-06 |
| ENSG00000148677 | ANKRD1 | 1.5543018 | 3.1762823 | 1.6219806 | 0.0005141 | 0.0064984 |
| ENSG00000112096 | SOD2 | 13.634577 | 15.249179 | 1.6146023 | 3.38E-05 | 0.0006646 |
| ENSG00000184500 | PROS1 | 7.0555325 | 8.6677748 | 1.6122424 | 6.09E-10 | 4.66E-08 |
| ENSG00000134569 | LRP4 | 3.4877425 | 5.0996429 | 1.6119003 | 0.0020399 | 0.0197798 |
| ENSG00000198019 | FCGR1B | 7.9872518 | 9.5966392 | 1.6093874 | 1.84E-12 | 2.39E-10 |
| ENSG00000117228 | GBP1 | 9.578173 | 11.18741 | 1.6092367 | 4.38E-07 | 1.57E-05 |
| ENSG00000160870 | CYP3A7 | 3.454861 | 5.0630812 | 1.6082202 | 0.0007638 | 0.0089752 |
| ENSG00000120156 | TEK | 1.3744877 | 2.9761917 | 1.601704 | 0.0005114 | 0.0064701 |
| ENSG00000186891 | TNFRSF18 | 3.8500693 | 5.4416592 | 1.5915898 | 0.000692 | 0.0082912 |
| ENSG00000069667 | RORA | 7.6337791 | 9.2214784 | 1.5876993 | 5.21E-07 | 1.83E-05 |
| ENSG00000152953 | STK32B | 5.2672371 | 6.8525946 | 1.5853575 | 5.64E-09 | 3.32E-07 |
| ENSG00000110203 | FOLR3 | 3.8032695 | 5.3843216 | 1.5810521 | 0.0011612 | 0.0125393 |
| ENSG00000167850 | CD300C | 7.9176533 | 9.4953413 | 1.577688 | 3.10E-05 | 0.0006146 |
| ENSG00000111674 | ENO2 | 7.4227809 | 8.9996782 | 1.5768973 | 1.71E-09 | 1.15E-07 |
| ENSG00000073150 | PANX2 | 4.4718783 | 6.0472038 | 1.5753255 | 0.0005011 | 0.0063595 |
| ENSG00000165935 | SMCO2 | 1.0622067 | 2.6372796 | 1.5750728 | 0.0041289 | 0.0341381 |
| ENSG00000277258 | PCGF2 | 3.2152101 | 4.7896552 | 1.5744452 | 4.00E-05 | 0.0007665 |
| ENSG00000143545 | RAB13 | 10.33349 | 11.906323 | 1.5728322 | 2.02E-21 | 9.43E-19 |
| ENSG00000181458 | TMEM45A | 5.4367241 | 7.0083729 | 1.5716488 | 0.0005822 | 0.007212 |
| ENSG00000188786 | MTF1 | 11.295145 | 12.866584 | 1.5714397 | 1.64E-17 | 4.96E-15 |
| ENSG00000136689 | IL1RN | 11.839409 | 13.409891 | 1.5704816 | 0.0011478 | 0.0124261 |
| ENSG00000138061 | CYP1B1 | 13.863607 | 15.433959 | 1.5703523 | 2.39E-13 | 3.63E-11 |
| ENSG00000126251 | GPR42 | 0.2345223 | 1.8036063 | 1.569084 | 0.0033949 | 0.0293405 |
| ENSG00000185090 | MANEAL | 5.8705542 | 7.4392815 | 1.5687273 | 8.97E-11 | 8.36E-09 |
| ENSG00000134317 | GRHL1 | 2.8122888 | 4.3787781 | 1.5664892 | 0.0039077 | 0.0327131 |
| ENSG00000000971 | CFH | 5.4553256 | 7.0215777 | 1.566252 | 5.22E-06 | 0.0001331 |
| ENSG00000165474 | GJB2 | 7.2182149 | 8.7757704 | 1.5575555 | 0.000156 | 0.0024256 |
| ENSG00000206052 | DOK6 | 1.5225016 | 3.0783428 | 1.5558412 | 0.0013641 | 0.0142738 |
| ENSG00000154310 | TNIK | 11.375814 | 12.926945 | 1.5511312 | 7.18E-25 | 5.52E-22 |
| ENSG00000183153 | GJD3 | 5.1226749 | 6.6716814 | 1.5490065 | 5.90E-06 | 0.0001481 |
| ENSG00000183160 | TMEM119 | 6.7840562 | 8.3329464 | 1.5488902 | 0.000794 | 0.0092485 |
| ENSG00000102265 | TIMP1 | 10.752134 | 12.295934 | 1.5437997 | 1.48E-08 | 7.92E-07 |
| ENSG00000105825 | TFPI2 | 0.1857144 | 1.7287931 | 1.5430788 | 0.0018388 | 0.018184 |
| ENSG00000130477 | UNC13A | 4.0601608 | 5.601018 | 1.5408572 | 1.02E-05 | 0.0002382 |
| ENSG00000173110 | HSPA6 | 7.6586051 | 9.1967299 | 1.5381247 | 5.14E-05 | 0.0009471 |
| ENSG00000108342 | CSF3 | 0.7172598 | 2.2544429 | 1.5371831 | 0.0033237 | 0.0288557 |
| ENSG00000249751 | ECSCR | 0 | 1.5355114 | 1.5355114 | 0.004044 | 0.0336265 |
| ENSG00000106571 | GLI3 | 2.1128771 | 3.6469465 | 1.5340694 | 0.0058609 | 0.0447643 |
| ENSG00000086300 | SNX10 | 10.818925 | 12.352804 | 1.5338783 | 1.70E-15 | 3.92E-13 |
| ENSG00000001561 | ENPP4 | 9.3939505 | 10.926033 | 1.5320826 | 5.48E-26 | 5.12E-23 |
| ENSG00000067057 | PFKP | 9.3823607 | 10.913981 | 1.53162 | 2.00E-10 | 1.69E-08 |
| ENSG00000196209 | SIRPB2 | 9.0964972 | 10.628012 | 1.5315148 | 5.65E-06 | 0.0001424 |
| ENSG00000106089 | STX1A | 5.2366693 | 6.7648062 | 1.5281368 | 2.37E-08 | 1.21E-06 |
| ENSG00000197506 | SLC28A3 | 8.3909617 | 9.9186485 | 1.5276867 | 0.0033981 | 0.0293626 |
| ENSG00000122862 | SRGN | 12.975944 | 14.501999 | 1.5260553 | 5.38E-17 | 1.48E-14 |
| ENSG00000082397 | EPB41L3 | 12.647598 | 14.168981 | 1.5213828 | 3.04E-21 | 1.37E-18 |
| ENSG00000116285 | ERRFI1 | 6.1473308 | 7.6673999 | 1.5200692 | 4.03E-05 | 0.0007719 |
| ENSG00000164741 | DLC1 | 6.4204895 | 7.9395378 | 1.5190482 | 1.56E-06 | 4.71E-05 |
| ENSG00000090376 | IRAK3 | 11.894042 | 13.409922 | 1.5158808 | 1.84E-34 | 5.40E-31 |
| ENSG00000085117 | CD82 | 10.692476 | 12.204765 | 1.5122895 | 9.36E-14 | 1.56E-11 |
| ENSG00000113763 | UNC5A | 7.9772033 | 9.4869428 | 1.5097395 | 0.0029882 | 0.0266715 |
| ENSG00000133019 | CHRM3 | 0 | 1.5084347 | 1.5084347 | 0.0045442 | 0.0369247 |
| ENSG00000111058 | ACSS3 | 3.4152586 | 4.9218007 | 1.5065421 | 0.0002061 | 0.0030701 |
| ENSG00000158428 | CATIP | 4.097499 | 5.598456 | 1.500957 | 0.0001207 | 0.0019524 |
| ENSG00000102007 | PLP2 | 9.5523223 | 11.052298 | 1.4999755 | 8.61E-14 | 1.45E-11 |
| ENSG00000186047 | DLEU7 | 6.8857729 | 8.3856579 | 1.4998849 | 4.05E-11 | 4.01E-09 |
| ENSG00000155511 | GRIA1 | 0 | 1.4977654 | 1.4977654 | 0.0013496 | 0.014156 |
| ENSG00000130202 | NECTIN2 | 9.98077 | 11.474602 | 1.4938319 | 1.10E-14 | 2.18E-12 |
| ENSG00000155659 | VSIG4 | 11.082094 | 12.575372 | 1.4932786 | 9.49E-09 | 5.28E-07 |
| ENSG00000120217 | CD274 | 8.6249325 | 10.114742 | 1.4898092 | 0.0008204 | 0.0094926 |
| ENSG00000197555 | SIPA1L1 | 10.021198 | 11.510974 | 1.4897759 | 4.34E-09 | 2.63E-07 |
| ENSG00000189221 | MAOA | 7.8376874 | 9.3228013 | 1.4851139 | 6.38E-07 | 2.17E-05 |
| ENSG00000116299 | KIAA1324 | 2.2006555 | 3.6834392 | 1.4827837 | 0.0060189 | 0.0457092 |
| ENSG00000132718 | SYT11 | 9.5455367 | 11.027553 | 1.4820167 | 8.26E-15 | 1.66E-12 |
| ENSG00000197249 | SERPINA1 | 12.118912 | 13.599478 | 1.4805654 | 0.0002752 | 0.003898 |
| ENSG00000147896 | IFNK | 6.2110669 | 7.6880545 | 1.4769876 | 1.65E-15 | 3.82E-13 |
| ENSG00000138378 | STAT4 | 6.1711656 | 7.6399949 | 1.4688294 | 4.92E-05 | 0.0009141 |
| ENSG00000145901 | TNIP1 | 11.893989 | 13.360446 | 1.4664571 | 1.17E-12 | 1.57E-10 |
| ENSG00000167703 | SLC43A2 | 12.063501 | 13.524716 | 1.4612149 | 7.70E-13 | 1.08E-10 |
| ENSG00000198805 | PNP | 8.8557541 | 10.313188 | 1.4574336 | 4.43E-09 | 2.68E-07 |
| ENSG00000022556 | NLRP2 | 7.5240723 | 8.9814532 | 1.4573809 | 0.0002649 | 0.0037812 |
| ENSG00000137462 | TLR2 | 10.18908 | 11.645828 | 1.456748 | 1.16E-10 | 1.05E-08 |
| ENSG00000184584 | TMEM173 | 7.4199657 | 8.8767045 | 1.4567387 | 5.65E-09 | 3.32E-07 |
| ENSG00000188372 | ZP3 | 5.6111763 | 7.0661322 | 1.4549559 | 0.002518 | 0.0232697 |
| ENSG00000111729 | CLEC4A | 9.7015149 | 11.152216 | 1.4507014 | 1.04E-30 | 1.76E-27 |
| ENSG00000151715 | TMEM45B | 4.695246 | 6.1419425 | 1.4466965 | 1.85E-05 | 0.0003966 |
| ENSG00000166825 | ANPEP | 13.403075 | 14.849679 | 1.4466035 | 2.61E-11 | 2.70E-09 |
| ENSG00000196352 | CD55 | 9.0136725 | 10.458959 | 1.4452863 | 2.38E-10 | 1.98E-08 |
| ENSG00000122729 | ACO1 | 11.239897 | 12.6819 | 1.4420023 | 8.89E-18 | 2.79E-15 |
| ENSG00000184678 | HIST2H2BE | 8.0351363 | 9.4768254 | 1.4416891 | 4.89E-08 | 2.30E-06 |
| ENSG00000243649 | CFB | 7.6497098 | 9.0896222 | 1.4399124 | 3.07E-09 | 1.93E-07 |
| ENSG00000151693 | ASAP2 | 7.2696611 | 8.7078237 | 1.4381625 | 3.06E-08 | 1.52E-06 |
| ENSG00000112715 | VEGFA | 8.7734512 | 10.210808 | 1.437357 | 3.55E-06 | 9.59E-05 |
| ENSG00000146476 | ARMT1 | 9.0299425 | 10.465141 | 1.4351984 | 2.83E-09 | 1.79E-07 |
| ENSG00000169994 | MYO7B | 3.7898014 | 5.2249361 | 1.4351347 | 0.0001889 | 0.0028504 |
| ENSG00000101017 | CD40 | 9.4838795 | 10.918202 | 1.4343227 | 6.84E-08 | 3.11E-06 |
| ENSG00000100731 | PCNX1 | 12.306078 | 13.738771 | 1.4326933 | 8.43E-09 | 4.74E-07 |
| ENSG00000005379 | TSPOAP1 | 6.5153622 | 7.9475138 | 1.4321516 | 4.33E-05 | 0.0008186 |
| ENSG00000157557 | ETS2 | 11.796437 | 13.224221 | 1.4277834 | 0.0003537 | 0.0048024 |
| ENSG00000255398 | HCAR3 | 3.3915532 | 4.8143742 | 1.422821 | 0.0016015 | 0.0162317 |
| ENSG00000170458 | CD14 | 13.872264 | 15.293726 | 1.4214615 | 1.00E-07 | 4.37E-06 |
| ENSG00000005249 | PRKAR2B | 6.8378774 | 8.2563591 | 1.4184817 | 0.0001305 | 0.0020839 |
| ENSG00000060558 | GNA15 | 10.388391 | 11.806231 | 1.4178399 | 8.19E-15 | 1.66E-12 |
| ENSG00000056558 | TRAF1 | 8.3690167 | 9.7854331 | 1.4164164 | 3.63E-05 | 0.0007069 |
| ENSG00000175294 | CATSPER1 | 3.2961897 | 4.7088676 | 1.4126779 | 0.0049622 | 0.0394309 |
| ENSG00000102683 | SGCG | 4.0322643 | 5.4430186 | 1.4107544 | 4.18E-07 | 1.50E-05 |
| ENSG00000175592 | FOSL1 | 4.7006315 | 6.099779 | 1.3991475 | 1.83E-06 | 5.42E-05 |
| ENSG00000143771 | CNIH4 | 8.9467734 | 10.344 | 1.3972263 | 3.69E-12 | 4.44E-10 |
| ENSG00000128917 | DLL4 | 0 | 1.3970034 | 1.3970034 | 0.0034185 | 0.0295043 |
| ENSG00000120262 | CCDC170 | 8.1798613 | 9.5720544 | 1.3921931 | 1.25E-05 | 0.0002835 |
| ENSG00000161921 | CXCL16 | 11.544385 | 12.932972 | 1.3885869 | 1.14E-20 | 4.97E-18 |
| ENSG00000203499 | IQANK1 | 1.816791 | 3.204222 | 1.3874311 | 0.0007537 | 0.008869 |
| ENSG00000136379 | ABHD17C | 3.5602887 | 4.9462349 | 1.3859462 | 0.0007032 | 0.0084047 |
| ENSG00000148175 | STOM | 12.130449 | 13.514179 | 1.38373 | 2.06E-26 | 2.27E-23 |
| ENSG00000116574 | RHOU | 9.9485727 | 11.328411 | 1.379838 | 3.52E-08 | 1.70E-06 |
| ENSG00000186583 | SPATC1 | 6.5926538 | 7.9675456 | 1.3748918 | 1.19E-05 | 0.0002719 |
| ENSG00000180549 | FUT7 | 5.2373415 | 6.6082037 | 1.3708622 | 0.0014358 | 0.0148533 |
| ENSG00000180316 | PNPLA1 | 4.3755126 | 5.7463395 | 1.3708268 | 0.0053419 | 0.0417304 |
| ENSG00000104783 | KCNN4 | 9.479152 | 10.849605 | 1.3704533 | 0.0001645 | 0.0025384 |
| ENSG00000107738 | VSIR | 11.01561 | 12.380411 | 1.3648006 | 7.49E-09 | 4.28E-07 |
| ENSG00000147852 | VLDLR | 5.8671548 | 7.2313543 | 1.3641994 | 0.0006006 | 0.0073849 |
| ENSG00000008517 | IL32 | 6.5034254 | 7.8652794 | 1.361854 | 0.00215 | 0.0206339 |
| ENSG00000090924 | PLEKHG2 | 9.5087096 | 10.870146 | 1.3614366 | 3.63E-07 | 1.34E-05 |
| ENSG00000151062 | CACNA2D4 | 8.1358371 | 9.4927949 | 1.3569578 | 2.12E-13 | 3.25E-11 |
| ENSG00000087903 | RFX2 | 7.3817546 | 8.7385462 | 1.3567916 | 7.02E-09 | 4.04E-07 |
| ENSG00000069974 | RAB27A | 9.1714571 | 10.52532 | 1.3538629 | 1.49E-14 | 2.86E-12 |
| ENSG00000116260 | QSOX1 | 11.688364 | 13.040199 | 1.3518346 | 5.16E-14 | 8.89E-12 |
| ENSG00000156011 | PSD3 | 9.9623665 | 11.306805 | 1.344438 | 1.15E-06 | 3.61E-05 |
| ENSG00000102897 | LYRM1 | 7.7821524 | 9.1212813 | 1.3391289 | 2.86E-13 | 4.29E-11 |
| ENSG00000058866 | DGKG | 6.2199104 | 7.5549579 | 1.3350474 | 0.0002402 | 0.0034933 |
| ENSG00000163291 | PAQR3 | 8.8176558 | 10.14869 | 1.331034 | 3.63E-05 | 0.0007069 |
| ENSG00000178127 | NDUFV2 | 10.194402 | 11.520675 | 1.3262721 | 1.59E-20 | 6.84E-18 |
| ENSG00000169764 | UGP2 | 11.528013 | 12.851365 | 1.3233521 | 1.65E-22 | 9.18E-20 |
| ENSG00000135821 | GLUL | 15.261154 | 16.584352 | 1.3231981 | 1.00E-06 | 3.21E-05 |
| ENSG00000163563 | MNDA | 11.29167 | 12.61362 | 1.3219498 | 3.00E-13 | 4.48E-11 |
| ENSG00000085733 | CTTN | 9.1624295 | 10.482712 | 1.3202828 | 3.90E-05 | 0.0007507 |
| ENSG00000184588 | PDE4B | 9.5643812 | 10.88447 | 1.3200888 | 3.57E-05 | 0.0006975 |
| ENSG00000083720 | OXCT1 | 5.2177201 | 6.5377462 | 1.3200261 | 0.0004257 | 0.0055687 |
| ENSG00000118094 | TREH | 2.9164775 | 4.2359955 | 1.3195179 | 0.0022231 | 0.0211772 |
| ENSG00000278588 | HIST1H2BI | 4.742659 | 6.0614205 | 1.3187615 | 9.34E-06 | 0.0002203 |
| ENSG00000148926 | ADM | 9.7791444 | 11.095086 | 1.3159419 | 6.93E-05 | 0.0012261 |
| ENSG00000150938 | CRIM1 | 11.558562 | 12.866915 | 1.3083533 | 4.82E-06 | 0.0001246 |
| ENSG00000170385 | SLC30A1 | 12.020883 | 13.327326 | 1.3064431 | 1.49E-22 | 8.39E-20 |
| ENSG00000142621 | FHAD1 | 6.5241231 | 7.8303146 | 1.3061916 | 4.02E-06 | 0.0001065 |
| ENSG00000161955 | TNFSF13 | 10.847769 | 12.153146 | 1.3053777 | 9.64E-09 | 5.36E-07 |
| ENSG00000135048 | TMEM2 | 11.801058 | 13.104156 | 1.3030978 | 0.0002344 | 0.0034232 |
| ENSG00000124839 | RAB17 | 3.1013469 | 4.4041615 | 1.3028146 | 0.0042521 | 0.0350381 |
| ENSG00000017260 | ATP2C1 | 11.659037 | 12.961143 | 1.3021061 | 1.53E-33 | 3.93E-30 |
| ENSG00000099954 | CECR2 | 4.4368963 | 5.738572 | 1.3016757 | 8.25E-05 | 0.0014198 |
| ENSG00000129355 | CDKN2D | 4.5578643 | 5.8590525 | 1.3011881 | 7.20E-05 | 0.001269 |
| ENSG00000138134 | STAMBPL1 | 4.2232836 | 5.5206687 | 1.297385 | 2.37E-05 | 0.0004891 |
| ENSG00000174125 | TLR1 | 11.301747 | 12.596899 | 1.2951517 | 4.49E-16 | 1.11E-13 |
| ENSG00000113369 | ARRDC3 | 10.540077 | 11.833949 | 1.2938718 | 6.51E-06 | 0.0001617 |
| ENSG00000157227 | MMP14 | 14.067252 | 15.356332 | 1.2890804 | 7.26E-20 | 2.92E-17 |
| ENSG00000128340 | RAC2 | 11.185195 | 12.473444 | 1.2882488 | 0.0001167 | 0.0019028 |
| ENSG00000163874 | ZC3H12A | 9.6123679 | 10.900298 | 1.2879299 | 2.49E-05 | 0.0005096 |
| ENSG00000175354 | PTPN2 | 10.231346 | 11.514347 | 1.283001 | 9.62E-24 | 6.12E-21 |
| ENSG00000135503 | ACVR1B | 9.8207089 | 11.103302 | 1.2825932 | 8.90E-17 | 2.40E-14 |
| ENSG00000179104 | TMTC2 | 7.8022587 | 9.082465 | 1.2802063 | 2.20E-09 | 1.44E-07 |
| ENSG00000153208 | MERTK | 11.973379 | 13.248123 | 1.274744 | 5.08E-08 | 2.39E-06 |
| ENSG00000176595 | KBTBD11 | 4.3809532 | 5.6556075 | 1.2746543 | 0.0004685 | 0.0060266 |
| ENSG00000145936 | KCNMB1 | 7.936313 | 9.2080461 | 1.2717331 | 3.47E-08 | 1.68E-06 |
| ENSG00000126860 | EVI2A | 9.4526271 | 10.716982 | 1.2643547 | 0.0030901 | 0.0273912 |
| ENSG00000106258 | CYP3A5 | 7.4224415 | 8.6845304 | 1.2620888 | 1.35E-05 | 0.0003021 |
| ENSG00000121297 | TSHZ3 | 9.0721887 | 10.329128 | 1.2569394 | 1.46E-13 | 2.31E-11 |
| ENSG00000102921 | N4BP1 | 11.75543 | 13.010246 | 1.2548154 | 1.99E-50 | 1.74E-46 |
| ENSG00000106780 | MEGF9 | 11.116254 | 12.367405 | 1.2511504 | 1.27E-09 | 8.92E-08 |
| ENSG00000198121 | LPAR1 | 9.2581201 | 10.506581 | 1.248461 | 2.39E-07 | 9.28E-06 |
| ENSG00000011422 | PLAUR | 12.063865 | 13.307995 | 1.2441293 | 3.88E-08 | 1.86E-06 |
| ENSG00000131979 | GCH1 | 7.691135 | 8.9350655 | 1.2439305 | 0.0001189 | 0.0019322 |
| ENSG00000181631 | P2RY13 | 8.9960161 | 10.239648 | 1.2436318 | 0.000152 | 0.0023764 |
| ENSG00000162734 | PEA15 | 12.303023 | 13.546 | 1.242977 | 1.04E-05 | 0.0002421 |
| ENSG00000125384 | PTGER2 | 8.2606559 | 9.5028793 | 1.2422235 | 2.03E-05 | 0.000431 |
| ENSG00000141497 | ZMYND15 | 7.4535372 | 8.6950208 | 1.2414836 | 7.53E-06 | 0.0001829 |
| ENSG00000047597 | XK | 3.6343299 | 4.8716914 | 1.2373615 | 0.0002386 | 0.0034736 |
| ENSG00000144802 | NFKBIZ | 9.8794622 | 11.115549 | 1.236087 | 0.0001938 | 0.0029072 |
| ENSG00000111640 | GAPDH | 14.632834 | 15.868891 | 1.2360573 | 1.56E-14 | 3.00E-12 |
| ENSG00000102755 | FLT1 | 8.0967216 | 9.3325795 | 1.2358579 | 0.0008238 | 0.0095219 |
| ENSG00000168214 | RBPJ | 11.999057 | 13.227916 | 1.2288587 | 1.32E-16 | 3.51E-14 |
| ENSG00000172575 | RASGRP1 | 7.6061047 | 8.8339342 | 1.2278295 | 3.81E-06 | 0.0001019 |
| ENSG00000166780 | C16orf45 | 6.1611915 | 7.3883824 | 1.2271909 | 3.78E-07 | 1.39E-05 |
| ENSG00000167460 | TPM4 | 13.377784 | 14.600642 | 1.222858 | 2.35E-12 | 2.96E-10 |
| ENSG00000187240 | DYNC2H1 | 8.0351373 | 9.2577463 | 1.222609 | 2.51E-07 | 9.68E-06 |
| ENSG00000164604 | GPR85 | 5.90057 | 7.1184345 | 1.2178645 | 7.79E-05 | 0.001355 |
| ENSG00000122694 | GLIPR2 | 10.411435 | 11.629039 | 1.2176031 | 2.25E-05 | 0.0004678 |
| ENSG00000254087 | LYN | 13.256395 | 14.473752 | 1.2173577 | 5.74E-26 | 5.25E-23 |
| ENSG00000183386 | FHL3 | 8.2441156 | 9.4607767 | 1.2166611 | 5.62E-07 | 1.95E-05 |
| ENSG00000115271 | GCA | 9.3669061 | 10.582354 | 1.2154482 | 1.12E-18 | 3.79E-16 |
| ENSG00000204577 | LILRB3 | 10.069857 | 11.285231 | 1.2153738 | 1.78E-06 | 5.29E-05 |
| ENSG00000173530 | TNFRSF10D | 8.6768112 | 9.8900665 | 1.2132553 | 9.46E-06 | 0.0002228 |
| ENSG00000271321 | CTAGE6 | 1.9572228 | 3.170366 | 1.2131432 | 0.0003327 | 0.0045589 |
| ENSG00000104951 | IL4I1 | 11.08846 | 12.301397 | 1.2129378 | 0.0007122 | 0.008496 |
| ENSG00000187556 | NANOS3 | 2.0928715 | 3.2981265 | 1.205255 | 0.0066117 | 0.0491725 |
| ENSG00000110876 | SELPLG | 10.574397 | 11.77655 | 1.2021532 | 4.11E-07 | 1.49E-05 |
| ENSG00000101966 | XIAP | 9.7901831 | 10.992207 | 1.202024 | 0.0002411 | 0.0035022 |
| ENSG00000091136 | LAMB1 | 6.9247712 | 8.124466 | 1.1996948 | 3.11E-08 | 1.54E-06 |
| ENSG00000189060 | H1F0 | 10.124134 | 11.322278 | 1.1981433 | 8.07E-06 | 0.0001937 |
| ENSG00000087589 | CASS4 | 9.1845093 | 10.379304 | 1.1947942 | 1.58E-06 | 4.78E-05 |
| ENSG00000147168 | IL2RG | 9.2689823 | 10.46156 | 1.1925773 | 0.001432 | 0.0148386 |
| ENSG00000106701 | FSD1L | 8.7753717 | 9.9674476 | 1.1920759 | 1.17E-07 | 5.01E-06 |
| ENSG00000050405 | LIMA1 | 9.7847247 | 10.973706 | 1.1889809 | 7.21E-07 | 2.42E-05 |
| ENSG00000163697 | APBB2 | 7.8527475 | 9.0397205 | 1.186973 | 0.0003345 | 0.0045788 |
| ENSG00000184357 | HIST1H1B | 6.3254729 | 7.5084204 | 1.1829475 | 0.0001823 | 0.0027689 |
| ENSG00000196923 | PDLIM7 | 9.6890161 | 10.871406 | 1.1823902 | 7.05E-09 | 4.05E-07 |
| ENSG00000174749 | C4orf32 | 7.4011725 | 8.5826339 | 1.1814613 | 0.000331 | 0.0045433 |
| ENSG00000169136 | ATF5 | 10.851058 | 12.03143 | 1.1803712 | 3.23E-07 | 1.21E-05 |
| ENSG00000007129 | CEACAM21 | 4.0466657 | 5.2266654 | 1.1799997 | 0.0015856 | 0.0161084 |
| ENSG00000131435 | PDLIM4 | 6.0609523 | 7.2383535 | 1.1774012 | 0.0023577 | 0.0221098 |
| ENSG00000244482 | LILRA6 | 10.149888 | 11.327182 | 1.1772943 | 1.94E-06 | 5.70E-05 |
| ENSG00000101336 | HCK | 12.807294 | 13.982752 | 1.1754579 | 6.17E-18 | 1.98E-15 |
| ENSG00000197272 | IL27 | 3.6269013 | 4.8013943 | 1.174493 | 1.50E-05 | 0.0003308 |
| ENSG00000148848 | ADAM12 | 5.3458565 | 6.5119344 | 1.1660779 | 0.0001036 | 0.0017233 |
| ENSG00000100368 | CSF2RB | 11.957874 | 13.122066 | 1.1641921 | 7.48E-14 | 1.27E-11 |
| ENSG00000198223 | CSF2RA | 10.26956 | 11.42869 | 1.1591293 | 8.78E-12 | 9.85E-10 |
| ENSG00000100902 | PSMA6 | 11.142064 | 12.299579 | 1.1575151 | 5.27E-09 | 3.12E-07 |
| ENSG00000135926 | TMBIM1 | 12.206865 | 13.364285 | 1.1574198 | 0.0001529 | 0.0023879 |
| ENSG00000185245 | GP1BA | 7.4506362 | 8.6035434 | 1.1529072 | 3.45E-05 | 0.0006768 |
| ENSG00000163162 | RNF149 | 10.765232 | 11.912627 | 1.1473955 | 7.17E-13 | 1.02E-10 |
| ENSG00000027697 | IFNGR1 | 11.822565 | 12.969822 | 1.147257 | 3.76E-06 | 0.0001006 |
| ENSG00000099250 | NRP1 | 13.554256 | 14.700398 | 1.1461412 | 1.43E-08 | 7.71E-07 |
| ENSG00000143554 | SLC27A3 | 8.4579761 | 9.6035055 | 1.1455294 | 7.64E-05 | 0.0013327 |
| ENSG00000155893 | PXYLP1 | 5.1366836 | 6.2817706 | 1.1450871 | 2.73E-06 | 7.64E-05 |
| ENSG00000075420 | FNDC3B | 13.332353 | 14.476669 | 1.1443165 | 5.96E-07 | 2.05E-05 |
| ENSG00000104419 | NDRG1 | 11.202482 | 12.346678 | 1.144196 | 1.65E-14 | 3.15E-12 |
| ENSG00000150337 | FCGR1A | 10.723539 | 11.862925 | 1.1393862 | 2.54E-09 | 1.63E-07 |
| ENSG00000133574 | GIMAP4 | 9.9497835 | 11.082493 | 1.1327099 | 1.79E-06 | 5.32E-05 |
| ENSG00000090339 | ICAM1 | 13.046198 | 14.178391 | 1.1321925 | 1.07E-06 | 3.38E-05 |
| ENSG00000163661 | PTX3 | 6.0175284 | 7.1488845 | 1.1313561 | 0.0066635 | 0.0494738 |
| ENSG00000140368 | PSTPIP1 | 9.7503496 | 10.880822 | 1.1304724 | 4.35E-05 | 0.0008223 |
| ENSG00000164440 | TXLNB | 7.3560349 | 8.4863566 | 1.1303217 | 0.002482 | 0.0229997 |
| ENSG00000009790 | TRAF3IP3 | 8.2467842 | 9.3765776 | 1.1297934 | 2.24E-10 | 1.87E-08 |
| ENSG00000158457 | TSPAN33 | 10.003699 | 11.131367 | 1.1276682 | 0.0014018 | 0.0145813 |
| ENSG00000135047 | CTSL | 14.578894 | 15.703559 | 1.1246654 | 3.59E-06 | 9.69E-05 |
| ENSG00000173334 | TRIB1 | 10.878238 | 12.002613 | 1.124375 | 0.0002516 | 0.0036322 |
| ENSG00000122884 | P4HA1 | 11.006365 | 12.129701 | 1.1233364 | 1.13E-12 | 1.53E-10 |
| ENSG00000261594 | TPBGL | 4.7397289 | 5.8606839 | 1.120955 | 0.0010211 | 0.0112846 |
| ENSG00000273802 | HIST1H2BG | 6.2498603 | 7.3685711 | 1.1187108 | 9.15E-06 | 0.0002168 |
| ENSG00000064199 | SPA17 | 5.6784659 | 6.794382 | 1.1159161 | 0.0008167 | 0.0094651 |
| ENSG00000049249 | TNFRSF9 | 7.7028818 | 8.8152515 | 1.1123697 | 0.0065758 | 0.0489481 |
| ENSG00000118276 | B4GALT6 | 5.1457279 | 6.2570876 | 1.1113597 | 0.0003627 | 0.0049059 |
| ENSG00000100889 | PCK2 | 10.539437 | 11.649525 | 1.1100884 | 2.32E-06 | 6.64E-05 |
| ENSG00000139970 | RTN1 | 9.6306608 | 10.738453 | 1.1077923 | 0.0007439 | 0.0087903 |
| ENSG00000136026 | CKAP4 | 10.776872 | 11.883996 | 1.1071231 | 1.55E-07 | 6.42E-06 |
| ENSG00000187325 | TAF9B | 7.9746455 | 9.0802597 | 1.1056142 | 1.31E-07 | 5.52E-06 |
| ENSG00000025708 | TYMP | 11.422898 | 12.524539 | 1.1016415 | 9.99E-05 | 0.00167 |
| ENSG00000277632 | CCL3 | 10.481756 | 11.583032 | 1.1012751 | 0.0009322 | 0.0104994 |
| ENSG00000005844 | ITGAL | 10.479348 | 11.580463 | 1.1011152 | 1.31E-05 | 0.0002938 |
| ENSG00000166927 | MS4A7 | 12.035487 | 13.136267 | 1.1007797 | 2.94E-07 | 1.11E-05 |
| ENSG00000101384 | JAG1 | 9.5686737 | 10.668971 | 1.1002972 | 0.0002092 | 0.003104 |
| ENSG00000159399 | HK2 | 12.545083 | 13.641525 | 1.0964425 | 4.65E-12 | 5.52E-10 |
| ENSG00000079332 | SAR1A | 11.100433 | 12.196308 | 1.0958746 | 4.77E-09 | 2.85E-07 |
| ENSG00000157933 | SKI | 10.46955 | 11.564074 | 1.0945239 | 1.18E-10 | 1.06E-08 |
| ENSG00000171631 | P2RY6 | 9.1750743 | 10.268101 | 1.0930264 | 8.99E-05 | 0.0015245 |
| ENSG00000133561 | GIMAP6 | 8.6786437 | 9.7715835 | 1.0929398 | 0.0003189 | 0.0043973 |
| ENSG00000171860 | C3AR1 | 12.549528 | 13.640012 | 1.090484 | 5.66E-07 | 1.96E-05 |
| ENSG00000153214 | TMEM87B | 11.102181 | 12.190947 | 1.0887658 | 9.51E-12 | 1.05E-09 |
| ENSG00000113966 | ARL6 | 5.4381824 | 6.5262693 | 1.0880869 | 3.26E-06 | 8.91E-05 |
| ENSG00000146376 | ARHGAP18 | 12.86417 | 13.951973 | 1.087803 | 4.43E-25 | 3.60E-22 |
| ENSG00000158714 | SLAMF8 | 13.078968 | 14.16523 | 1.0862619 | 1.59E-09 | 1.08E-07 |
| ENSG00000197321 | SVIL | 9.4654559 | 10.551599 | 1.0861431 | 3.00E-05 | 0.0005987 |
| ENSG00000278463 | HIST1H2AB | 3.5999715 | 4.685434 | 1.0854625 | 0.0017331 | 0.017275 |
| ENSG00000157193 | LRP8 | 8.7298221 | 9.8146558 | 1.0848337 | 0.0016538 | 0.0166584 |
| ENSG00000229894 | GK3P | 6.8678336 | 7.9514075 | 1.0835739 | 1.80E-10 | 1.55E-08 |
| ENSG00000142405 | NLRP12 | 7.6321064 | 8.7130673 | 1.0809609 | 3.95E-05 | 0.000759 |
| ENSG00000112576 | CCND3 | 9.0128046 | 10.086356 | 1.0735518 | 1.41E-09 | 9.75E-08 |
| ENSG00000148459 | PDSS1 | 8.1318579 | 9.2020179 | 1.07016 | 1.95E-07 | 7.82E-06 |
| ENSG00000136603 | SKIL | 11.11149 | 12.180825 | 1.0693355 | 6.84E-07 | 2.31E-05 |
| ENSG00000115107 | STEAP3 | 10.479968 | 11.548313 | 1.0683449 | 4.12E-09 | 2.50E-07 |
| ENSG00000111669 | TPI1 | 12.861182 | 13.926449 | 1.0652673 | 2.68E-11 | 2.75E-09 |
| ENSG00000122861 | PLAU | 12.174513 | 13.239051 | 1.0645381 | 0.0018647 | 0.01839 |
| ENSG00000123104 | ITPR2 | 14.258555 | 15.321564 | 1.0630095 | 7.82E-16 | 1.91E-13 |
| ENSG00000002549 | LAP3 | 11.758191 | 12.820382 | 1.0621911 | 1.35E-11 | 1.46E-09 |
| ENSG00000137193 | PIM1 | 11.291046 | 12.35279 | 1.0617437 | 4.79E-09 | 2.86E-07 |
| ENSG00000186074 | CD300LF | 10.363111 | 11.422055 | 1.0589439 | 0.0002619 | 0.0037491 |
| ENSG00000064932 | SBNO2 | 10.574849 | 11.626216 | 1.0513666 | 8.36E-13 | 1.17E-10 |
| ENSG00000175591 | P2RY2 | 6.3516236 | 7.4023196 | 1.050696 | 1.84E-05 | 0.0003946 |
| ENSG00000120708 | TGFBI | 15.109972 | 16.160028 | 1.0500553 | 5.45E-06 | 0.0001379 |
| ENSG00000121552 | CSTA | 8.3655154 | 9.4153312 | 1.0498158 | 7.02E-10 | 5.26E-08 |
| ENSG00000139055 | ERP27 | 4.5333905 | 5.5823491 | 1.0489586 | 2.85E-05 | 0.000572 |
| ENSG00000007968 | E2F2 | 5.227852 | 6.2753147 | 1.0474627 | 0.0032156 | 0.0281855 |
| ENSG00000127947 | PTPN12 | 12.079458 | 13.126319 | 1.0468617 | 3.27E-08 | 1.60E-06 |
| ENSG00000146700 | SSC4D | 5.571008 | 6.615973 | 1.044965 | 0.0002708 | 0.003848 |
| ENSG00000136167 | LCP1 | 14.868318 | 15.912972 | 1.0446539 | 3.79E-05 | 0.0007338 |
| ENSG00000165030 | NFIL3 | 8.5157965 | 9.5561395 | 1.040343 | 2.27E-06 | 6.52E-05 |
| ENSG00000066697 | MSANTD3 | 8.9648854 | 10.003754 | 1.0388689 | 1.90E-06 | 5.60E-05 |
| ENSG00000140859 | KIFC3 | 10.837584 | 11.876364 | 1.0387806 | 3.00E-09 | 1.89E-07 |
| ENSG00000121594 | CD80 | 8.4295543 | 9.4681553 | 1.038601 | 1.11E-06 | 3.50E-05 |
| ENSG00000215114 | UBXN2B | 10.561206 | 11.598698 | 1.0374911 | 8.92E-12 | 9.96E-10 |
| ENSG00000179921 | GPBAR1 | 5.3824014 | 6.4195813 | 1.0371799 | 0.0001861 | 0.0028146 |
| ENSG00000111052 | LIN7A | 6.6889748 | 7.7258233 | 1.0368485 | 0.0006432 | 0.0077791 |
| ENSG00000161638 | ITGA5 | 12.030837 | 13.067678 | 1.0368416 | 7.64E-12 | 8.64E-10 |
| ENSG00000203814 | HIST2H2BF | 7.6758117 | 8.7123796 | 1.0365678 | 2.58E-07 | 9.92E-06 |
| ENSG00000153815 | CMIP | 11.911382 | 12.947094 | 1.0357117 | 4.98E-07 | 1.77E-05 |
| ENSG00000183696 | UPP1 | 9.3354485 | 10.370517 | 1.0350684 | 5.30E-08 | 2.47E-06 |
| ENSG00000089351 | GRAMD1A | 11.216471 | 12.250044 | 1.0335726 | 0.0002664 | 0.0037984 |
| ENSG00000198369 | SPRED2 | 9.6081878 | 10.641673 | 1.0334853 | 7.58E-08 | 3.41E-06 |
| ENSG00000143570 | SLC39A1 | 10.237253 | 11.270476 | 1.0332235 | 0.0003154 | 0.0043515 |
| ENSG00000120949 | TNFRSF8 | 6.5590035 | 7.5909162 | 1.0319127 | 0.0008447 | 0.0097154 |
| ENSG00000148737 | TCF7L2 | 9.1249494 | 10.156754 | 1.0318048 | 3.35E-06 | 9.12E-05 |
| ENSG00000180611 | MB21D2 | 8.3884933 | 9.4186776 | 1.0301843 | 1.78E-05 | 0.0003832 |
| ENSG00000101665 | SMAD7 | 9.5945111 | 10.620975 | 1.0264639 | 4.80E-08 | 2.26E-06 |
| ENSG00000138119 | MYOF | 12.790879 | 13.817195 | 1.0263162 | 6.21E-05 | 0.0011138 |
| ENSG00000129667 | RHBDF2 | 10.571381 | 11.596945 | 1.0255645 | 0.0002018 | 0.0030141 |
| ENSG00000176597 | B3GNT5 | 8.5486304 | 9.5720598 | 1.0234294 | 0.0016489 | 0.0166207 |
| ENSG00000183722 | LHFPL6 | 4.9804272 | 6.0032675 | 1.0228403 | 0.0031461 | 0.0277688 |
| ENSG00000125730 | C3 | 13.358398 | 14.379923 | 1.0215253 | 2.26E-08 | 1.16E-06 |
| ENSG00000197747 | S100A10 | 11.217686 | 12.238865 | 1.021179 | 7.53E-11 | 7.15E-09 |
| ENSG00000183484 | GPR132 | 7.6737136 | 8.6936373 | 1.0199237 | 1.71E-07 | 6.98E-06 |
| ENSG00000135749 | PCNX2 | 7.6767456 | 8.6961666 | 1.019421 | 1.44E-06 | 4.40E-05 |
| ENSG00000164466 | SFXN1 | 8.6229872 | 9.6416948 | 1.0187075 | 7.67E-07 | 2.56E-05 |
| ENSG00000177663 | IL17RA | 13.22969 | 14.248375 | 1.0186853 | 2.30E-09 | 1.50E-07 |
| ENSG00000109743 | BST1 | 8.8979042 | 9.9149858 | 1.0170816 | 2.11E-05 | 0.0004455 |
| ENSG00000151239 | TWF1 | 10.299063 | 11.315855 | 1.0167918 | 1.51E-08 | 8.08E-07 |
| ENSG00000056972 | TRAF3IP2 | 7.9252778 | 8.9414188 | 1.0161411 | 6.65E-06 | 0.0001647 |
| ENSG00000165168 | CYBB | 16.24417 | 17.259394 | 1.0152234 | 1.85E-10 | 1.58E-08 |
| ENSG00000162746 | FCRLB | 5.4982723 | 6.5131979 | 1.0149256 | 0.0011388 | 0.0123378 |
| ENSG00000114446 | IFT57 | 7.3441573 | 8.3585635 | 1.0144062 | 2.38E-08 | 1.22E-06 |
| ENSG00000006327 | TNFRSF12A | 6.5137849 | 7.5269595 | 1.0131747 | 0.0008123 | 0.0094167 |
| ENSG00000125629 | INSIG2 | 8.8004854 | 9.8129367 | 1.0124512 | 1.57E-08 | 8.38E-07 |
| ENSG00000064393 | HIPK2 | 13.270127 | 14.282135 | 1.012008 | 0.0004531 | 0.0058603 |
| ENSG00000147650 | LRP12 | 9.6933641 | 10.705206 | 1.011842 | 3.80E-07 | 1.39E-05 |
| ENSG00000179715 | PCED1B | 5.2244282 | 6.2360883 | 1.0116601 | 0.0016941 | 0.0169512 |
| ENSG00000143384 | MCL1 | 12.29652 | 13.307574 | 1.0110542 | 1.38E-12 | 1.82E-10 |
| ENSG00000086062 | B4GALT1 | 12.638926 | 13.649239 | 1.0103126 | 1.72E-19 | 6.56E-17 |
| ENSG00000171488 | LRRC8C | 10.914134 | 11.924419 | 1.0102848 | 8.41E-12 | 9.47E-10 |
| ENSG00000170791 | CHCHD7 | 8.8318916 | 9.8418409 | 1.0099493 | 2.80E-15 | 6.26E-13 |
| ENSG00000168899 | VAMP5 | 6.0901542 | 7.0983755 | 1.0082213 | 9.45E-05 | 0.001593 |
| ENSG00000205710 | C17orf107 | 3.9340984 | 4.9392074 | 1.0051091 | 0.0055103 | 0.0427791 |
| ENSG00000147459 | DOCK5 | 11.913957 | 12.918499 | 1.0045425 | 5.27E-07 | 1.85E-05 |
| ENSG00000160791 | CCR5 | 11.877359 | 12.881106 | 1.0037468 | 7.29E-07 | 2.44E-05 |
| ENSG00000161653 | NAGS | 5.294927 | 6.298482 | 1.003555 | 8.11E-06 | 0.0001945 |
| ENSG00000075399 | VPS9D1 | 8.77727 | 9.7731729 | 0.9959029 | 2.77E-07 | 1.05E-05 |
| ENSG00000160117 | ANKLE1 | 4.9259363 | 5.9207218 | 0.9947855 | 0.000614 | 0.00751 |
| ENSG00000166507 | NDST2 | 9.9445428 | 10.938474 | 0.9939308 | 5.80E-12 | 6.76E-10 |
| ENSG00000165304 | MELK | 5.5010439 | 6.4908068 | 0.9897629 | 0.0038361 | 0.0322521 |
| ENSG00000163251 | FZD5 | 9.7761191 | 10.763405 | 0.9872862 | 1.13E-05 | 0.000259 |
| ENSG00000105339 | DENND3 | 11.18764 | 12.174888 | 0.9872481 | 1.28E-09 | 8.96E-08 |
| ENSG00000134575 | ACP2 | 11.729073 | 12.716152 | 0.9870792 | 5.65E-05 | 0.0010299 |
| ENSG00000182853 | VMO1 | 5.284597 | 6.271242 | 0.9866451 | 0.0036565 | 0.0310516 |
| ENSG00000167600 | CYP2S1 | 9.8582377 | 10.84477 | 0.9865318 | 7.27E-06 | 0.0001777 |
| ENSG00000137101 | CD72 | 5.85246 | 6.8384958 | 0.9860358 | 0.0001272 | 0.0020422 |
| ENSG00000132356 | PRKAA1 | 10.851358 | 11.837123 | 0.9857658 | 3.03E-12 | 3.73E-10 |
| ENSG00000187037 | GPR141 | 9.47174 | 10.455831 | 0.9840906 | 4.80E-17 | 1.33E-14 |
| ENSG00000141526 | SLC16A3 | 12.127448 | 13.108917 | 0.9814691 | 1.20E-08 | 6.55E-07 |
| ENSG00000137563 | GGH | 7.2885156 | 8.2695064 | 0.9809909 | 1.15E-05 | 0.0002642 |
| ENSG00000150347 | ARID5B | 11.118684 | 12.09914 | 0.9804556 | 5.18E-07 | 1.83E-05 |
| ENSG00000170854 | RIOX2 | 8.7123471 | 9.6922354 | 0.9798883 | 1.08E-10 | 9.85E-09 |
| ENSG00000065911 | MTHFD2 | 10.750271 | 11.729279 | 0.9790082 | 1.56E-06 | 4.71E-05 |
| ENSG00000104881 | PPP1R13L | 4.1305164 | 5.1081966 | 0.9776803 | 0.0022922 | 0.0216344 |
| ENSG00000076356 | PLXNA2 | 8.5216118 | 9.4990302 | 0.9774184 | 0.0003358 | 0.0045911 |
| ENSG00000006459 | KDM7A | 12.038769 | 13.012048 | 0.9732787 | 2.15E-14 | 3.92E-12 |
| ENSG00000105948 | TTC26 | 3.7377923 | 4.7085095 | 0.9707171 | 0.0005707 | 0.0071016 |
| ENSG00000150667 | FSIP1 | 3.3143964 | 4.2782399 | 0.9638435 | 0.0046461 | 0.0375136 |
| ENSG00000197930 | ERO1A | 11.629163 | 12.592093 | 0.9629301 | 1.08E-13 | 1.76E-11 |
| ENSG00000142089 | IFITM3 | 9.32259 | 10.285468 | 0.9628777 | 0.0020896 | 0.0201551 |
| ENSG00000175489 | LRRC25 | 11.185777 | 12.147065 | 0.9612885 | 2.02E-09 | 1.33E-07 |
| ENSG00000165507 | C10orf10 | 9.2259943 | 10.186551 | 0.9605565 | 5.01E-05 | 0.0009274 |
| ENSG00000155926 | SLA | 11.746422 | 12.706969 | 0.9605472 | 2.41E-06 | 6.88E-05 |
| ENSG00000131042 | LILRB2 | 11.604007 | 12.562087 | 0.9580805 | 2.11E-05 | 0.0004452 |
| ENSG00000158373 | HIST1H2BD | 7.41768 | 8.3749892 | 0.9573092 | 3.57E-08 | 1.72E-06 |
| ENSG00000151726 | ACSL1 | 12.705345 | 13.661131 | 0.9557862 | 2.29E-07 | 8.92E-06 |
| ENSG00000010810 | FYN | 7.4287091 | 8.3840466 | 0.9553375 | 0.0023091 | 0.021737 |
| ENSG00000058056 | USP13 | 8.2235348 | 9.177183 | 0.9536482 | 0.0001108 | 0.0018225 |
| ENSG00000158270 | COLEC12 | 12.573558 | 13.525455 | 0.951897 | 0.0004491 | 0.0058233 |
| ENSG00000119403 | PHF19 | 7.8817263 | 8.8333377 | 0.9516115 | 0.0021055 | 0.0202831 |
| ENSG00000072682 | P4HA2 | 7.9409875 | 8.8908658 | 0.9498783 | 6.96E-06 | 0.0001713 |
| ENSG00000074181 | NOTCH3 | 10.13764 | 11.086927 | 0.9492873 | 0.0048416 | 0.0386852 |
| ENSG00000171155 | C1GALT1C1 | 9.5658131 | 10.513294 | 0.9474805 | 0.0066163 | 0.0491982 |
| ENSG00000196569 | LAMA2 | 7.2368027 | 8.1833212 | 0.9465185 | 0.0004056 | 0.0053616 |
| ENSG00000197557 | TTC30A | 6.7249761 | 7.6707008 | 0.9457246 | 7.85E-05 | 0.0013631 |
| ENSG00000102144 | PGK1 | 12.945257 | 13.889734 | 0.9444768 | 1.68E-10 | 1.44E-08 |
| ENSG00000109861 | CTSC | 14.340106 | 15.282654 | 0.9425473 | 5.10E-08 | 2.39E-06 |
| ENSG00000267534 | S1PR2 | 10.228735 | 11.17062 | 0.941885 | 1.36E-08 | 7.31E-07 |
| ENSG00000026751 | SLAMF7 | 12.161711 | 13.100928 | 0.9392178 | 0.0001591 | 0.002468 |
| ENSG00000109107 | ALDOC | 6.9178129 | 7.8537471 | 0.9359342 | 2.15E-05 | 0.0004517 |
| ENSG00000061656 | SPAG4 | 4.0242671 | 4.9567813 | 0.9325142 | 0.0042609 | 0.0350844 |
| ENSG00000136048 | DRAM1 | 12.629046 | 13.559372 | 0.9303258 | 0.0014741 | 0.0151827 |
| ENSG00000177674 | AGTRAP | 8.4109232 | 9.3400989 | 0.9291757 | 3.50E-06 | 9.47E-05 |
| ENSG00000075213 | SEMA3A | 8.0849607 | 9.013924 | 0.9289633 | 0.002009 | 0.0195367 |
| ENSG00000137393 | RNF144B | 11.777322 | 12.70461 | 0.9272886 | 4.70E-05 | 0.0008816 |
| ENSG00000054965 | FAM168A | 11.822406 | 12.749192 | 0.9267862 | 3.64E-16 | 9.18E-14 |
| ENSG00000137491 | SLCO2B1 | 14.079297 | 15.004246 | 0.9249482 | 0.0046486 | 0.0375145 |
| ENSG00000111348 | ARHGDIB | 13.1584 | 14.08217 | 0.92377 | 2.05E-05 | 0.000434 |
| ENSG00000183741 | CBX6 | 11.185169 | 12.108907 | 0.923738 | 0.0002948 | 0.0041205 |
| ENSG00000019144 | PHLDB1 | 9.0735175 | 9.9961952 | 0.9226777 | 0.0004099 | 0.0053979 |
| ENSG00000165169 | DYNLT3 | 9.9256912 | 10.846837 | 0.921146 | 3.86E-05 | 0.0007438 |
| ENSG00000140030 | GPR65 | 10.418791 | 11.339169 | 0.9203775 | 9.26E-09 | 5.18E-07 |
| ENSG00000257594 | GALNT4 | 8.5273949 | 9.4475323 | 0.9201374 | 0.0003726 | 0.005009 |
| ENSG00000204267 | TAP2 | 10.486852 | 11.403326 | 0.9164737 | 9.49E-07 | 3.07E-05 |
| ENSG00000086730 | LAT2 | 10.278001 | 11.193529 | 0.9155278 | 1.05E-06 | 3.32E-05 |
| ENSG00000106034 | CPED1 | 10.466999 | 11.381052 | 0.9140532 | 2.26E-06 | 6.51E-05 |
| ENSG00000134242 | PTPN22 | 10.25165 | 11.16415 | 0.9124999 | 1.27E-09 | 8.89E-08 |
| ENSG00000069399 | BCL3 | 9.061804 | 9.9722074 | 0.9104035 | 0.000345 | 0.0047003 |
| ENSG00000173744 | AGFG1 | 10.788185 | 11.698491 | 0.9103056 | 2.74E-11 | 2.79E-09 |
| ENSG00000173627 | APOBEC4 | 4.4270076 | 5.3367029 | 0.9096953 | 0.0029055 | 0.0260978 |
| ENSG00000159176 | CSRP1 | 11.242454 | 12.151287 | 0.9088334 | 7.93E-07 | 2.64E-05 |
| ENSG00000115919 | KYNU | 12.658731 | 13.564761 | 0.9060298 | 0.0003676 | 0.0049518 |
| ENSG00000074935 | TUBE1 | 6.454921 | 7.3588343 | 0.9039133 | 1.26E-08 | 6.81E-07 |
| ENSG00000096433 | ITPR3 | 6.1492169 | 7.0509086 | 0.9016917 | 0.0019537 | 0.0191004 |
| ENSG00000196776 | CD47 | 11.643121 | 12.544497 | 0.9013765 | 3.13E-14 | 5.51E-12 |
| ENSG00000180817 | PPA1 | 8.1164073 | 9.0142207 | 0.8978134 | 5.09E-05 | 0.0009394 |
| ENSG00000084764 | MAPRE3 | 8.4274169 | 9.3249472 | 0.8975302 | 2.61E-06 | 7.37E-05 |
| ENSG00000136997 | MYC | 7.959221 | 8.8556225 | 0.8964015 | 0.0024666 | 0.0229101 |
| ENSG00000095794 | CREM | 8.4433976 | 9.3396713 | 0.8962737 | 1.97E-05 | 0.0004186 |
| ENSG00000118292 | C1orf54 | 8.3777908 | 9.2726407 | 0.8948499 | 4.99E-05 | 0.0009249 |
| ENSG00000139505 | MTMR6 | 11.029222 | 11.923792 | 0.8945701 | 1.61E-13 | 2.53E-11 |
| ENSG00000134352 | IL6ST | 11.776414 | 12.669604 | 0.89319 | 3.28E-12 | 3.99E-10 |
| ENSG00000179241 | LDLRAD3 | 8.9339903 | 9.826343 | 0.8923527 | 0.0019153 | 0.0188029 |
| ENSG00000110435 | PDHX | 9.7204838 | 10.612735 | 0.8922511 | 2.40E-10 | 1.99E-08 |
| ENSG00000161647 | MPP3 | 5.1831414 | 6.0748718 | 0.8917304 | 0.0010685 | 0.0117259 |
| ENSG00000136867 | SLC31A2 | 9.4629822 | 10.354002 | 0.8910201 | 0.0003002 | 0.0041803 |
| ENSG00000105784 | RUNDC3B | 6.4889668 | 7.3789703 | 0.8900035 | 0.00291 | 0.0261225 |
| ENSG00000135046 | ANXA1 | 12.744677 | 13.630004 | 0.8853267 | 0.0002133 | 0.0031588 |
| ENSG00000249992 | TMEM158 | 5.1514038 | 6.0353652 | 0.8839615 | 0.0036208 | 0.0308279 |
| ENSG00000127838 | PNKD | 12.128562 | 13.010817 | 0.8822545 | 4.01E-06 | 0.0001064 |
| ENSG00000135698 | MPHOSPH6 | 8.7391336 | 9.6196047 | 0.8804711 | 5.48E-05 | 0.0010034 |
| ENSG00000249502 | AC006160.1 | 7.1174713 | 7.9969183 | 0.879447 | 0.0002883 | 0.0040436 |
| ENSG00000275713 | HIST1H2BH | 5.2894175 | 6.1675833 | 0.8781658 | 0.0019361 | 0.0189664 |
| ENSG00000111490 | TBC1D30 | 8.2931191 | 9.1705945 | 0.8774754 | 0.0001383 | 0.0021915 |
| ENSG00000125744 | RTN2 | 7.7959962 | 8.6717081 | 0.8757119 | 1.88E-07 | 7.62E-06 |
| ENSG00000123989 | CHPF | 4.8669345 | 5.7426069 | 0.8756724 | 0.002703 | 0.024648 |
| ENSG00000112033 | PPARD | 11.597918 | 12.473061 | 0.8751434 | 1.77E-10 | 1.52E-08 |
| ENSG00000131873 | CHSY1 | 10.074435 | 10.949368 | 0.8749327 | 7.80E-12 | 8.80E-10 |
| ENSG00000267060 | PTGES3L | 3.1011609 | 3.9752746 | 0.8741137 | 0.0038691 | 0.0324673 |
| ENSG00000244115 | DNAJC25-GNG10 | 8.6675055 | 9.5390377 | 0.8715322 | 8.49E-05 | 0.001454 |
| ENSG00000260170 | AC090527.2 | 5.150366 | 6.0209928 | 0.8706268 | 0.0014309 | 0.0148359 |
| ENSG00000116701 | NCF2 | 13.86625 | 14.735072 | 0.8688213 | 1.60E-06 | 4.82E-05 |
| ENSG00000180776 | ZDHHC20 | 11.604924 | 12.473557 | 0.8686335 | 2.40E-08 | 1.22E-06 |
| ENSG00000134755 | DSC2 | 10.489178 | 11.356326 | 0.8671472 | 0.0001394 | 0.0022078 |
| ENSG00000153989 | NUS1 | 11.320435 | 12.183044 | 0.862609 | 8.84E-08 | 3.91E-06 |
| ENSG00000107130 | NCS1 | 8.7397015 | 9.6013465 | 0.8616451 | 0.0056099 | 0.0433378 |
| ENSG00000169508 | GPR183 | 9.9875286 | 10.84813 | 0.8606014 | 9.53E-05 | 0.0016042 |
| ENSG00000142166 | IFNAR1 | 12.888854 | 13.749166 | 0.8603123 | 2.81E-18 | 9.21E-16 |
| ENSG00000081320 | STK17B | 10.828395 | 11.688115 | 0.8597203 | 1.20E-06 | 3.73E-05 |
| ENSG00000167553 | TUBA1C | 12.498087 | 13.357676 | 0.8595897 | 6.20E-10 | 4.73E-08 |
| ENSG00000114686 | MRPL3 | 9.0927795 | 9.952197 | 0.8594175 | 0.0055383 | 0.0429208 |
| ENSG00000103005 | USB1 | 10.141993 | 10.998821 | 0.8568275 | 2.28E-12 | 2.90E-10 |
| ENSG00000160999 | SH2B2 | 7.56465 | 8.4198614 | 0.8552114 | 0.0027318 | 0.0248684 |
| ENSG00000165029 | ABCA1 | 13.846686 | 14.700181 | 0.853495 | 5.07E-05 | 0.0009349 |
| ENSG00000154640 | BTG3 | 7.6469417 | 8.5003911 | 0.8534494 | 0.0007595 | 0.0089285 |
| ENSG00000163466 | ARPC2 | 13.41384 | 14.266014 | 0.852174 | 9.71E-20 | 3.81E-17 |
| ENSG00000107614 | TRDMT1 | 8.2662977 | 9.1173766 | 0.8510789 | 0.0050283 | 0.0398417 |
| ENSG00000197142 | ACSL5 | 10.780859 | 11.629455 | 0.8485962 | 4.08E-05 | 0.0007796 |
| ENSG00000102780 | DGKH | 10.111875 | 10.959879 | 0.8480043 | 0.0004064 | 0.0053702 |
| ENSG00000183307 | TMEM121B | 8.0982055 | 8.9431366 | 0.8449311 | 0.003474 | 0.0298984 |
| ENSG00000174791 | RIN1 | 6.7258775 | 7.5700683 | 0.8441908 | 0.0001142 | 0.0018681 |
| ENSG00000186567 | CEACAM19 | 5.5465291 | 6.3903183 | 0.8437891 | 0.0029266 | 0.0262286 |
| ENSG00000172216 | CEBPB | 10.744479 | 11.587819 | 0.8433405 | 0.0047844 | 0.0383274 |
| ENSG00000135318 | NT5E | 5.693676 | 6.5368759 | 0.8431999 | 0.0041017 | 0.0339961 |
| ENSG00000184988 | TMEM106A | 11.129957 | 11.973076 | 0.8431185 | 5.13E-05 | 0.0009448 |
| ENSG00000072571 | HMMR | 5.3492518 | 6.1906731 | 0.8414212 | 0.0006858 | 0.008234 |
| ENSG00000139496 | NUP58 | 11.448138 | 12.289231 | 0.841093 | 1.49E-09 | 1.02E-07 |
| ENSG00000035403 | VCL | 11.885788 | 12.725786 | 0.839998 | 1.84E-09 | 1.23E-07 |
| ENSG00000148180 | GSN | 13.434984 | 14.270733 | 0.8357493 | 8.97E-05 | 0.0015219 |
| ENSG00000118564 | FBXL5 | 11.40027 | 12.233367 | 0.8330967 | 4.58E-10 | 3.61E-08 |
| ENSG00000187116 | LILRA5 | 8.2608744 | 9.0936303 | 0.8327559 | 0.005649 | 0.0435247 |
| ENSG00000075303 | SLC25A40 | 10.060614 | 10.8932 | 0.8325866 | 9.17E-08 | 4.04E-06 |
| ENSG00000277224 | HIST1H2BF | 6.460393 | 7.2920704 | 0.8316774 | 0.0004918 | 0.0062677 |
| ENSG00000125753 | VASP | 11.466963 | 12.296057 | 0.8290941 | 4.68E-06 | 0.0001213 |
| ENSG00000068366 | ACSL4 | 12.010471 | 12.839488 | 0.8290165 | 6.66E-06 | 0.0001648 |
| ENSG00000056736 | IL17RB | 6.936793 | 7.764081 | 0.827288 | 7.11E-07 | 2.40E-05 |
| ENSG00000147894 | C9orf72 | 10.949425 | 11.776368 | 0.8269437 | 4.34E-08 | 2.06E-06 |
| ENSG00000101608 | MYL12A | 12.721599 | 13.548263 | 0.8266641 | 9.56E-11 | 8.87E-09 |
| ENSG00000126803 | HSPA2 | 4.651841 | 5.4688773 | 0.8170363 | 0.0054201 | 0.0422434 |
| ENSG00000149177 | PTPRJ | 13.173566 | 13.989011 | 0.8154444 | 5.76E-10 | 4.43E-08 |
| ENSG00000131724 | IL13RA1 | 12.324065 | 13.138947 | 0.8148822 | 7.94E-07 | 2.64E-05 |
| ENSG00000133794 | ARNTL | 9.6705596 | 10.484938 | 0.8143781 | 3.68E-07 | 1.36E-05 |
| ENSG00000179331 | RAB39A | 8.8291104 | 9.643441 | 0.8143307 | 0.0002352 | 0.00343 |
| ENSG00000187630 | DHRS4L2 | 8.2321391 | 9.0458179 | 0.8136788 | 0.0013414 | 0.0140807 |
| ENSG00000164105 | SAP30 | 6.3006851 | 7.1130166 | 0.8123315 | 3.48E-05 | 0.0006813 |
| ENSG00000109762 | SNX25 | 7.9412159 | 8.7533176 | 0.8121017 | 2.28E-07 | 8.89E-06 |
| ENSG00000159216 | RUNX1 | 11.515093 | 12.327005 | 0.811912 | 1.61E-10 | 1.39E-08 |
| ENSG00000137845 | ADAM10 | 13.131315 | 13.942916 | 0.8116016 | 1.23E-09 | 8.67E-08 |
| ENSG00000113916 | BCL6 | 11.263609 | 12.074713 | 0.8111041 | 3.00E-07 | 1.13E-05 |
| ENSG00000111254 | AKAP3 | 5.448466 | 6.2592603 | 0.8107943 | 0.0065307 | 0.0487147 |
| ENSG00000169403 | PTAFR | 12.900813 | 13.711552 | 0.8107386 | 1.75E-05 | 0.0003776 |
| ENSG00000049449 | RCN1 | 9.3730441 | 10.183568 | 0.8105242 | 0.0004435 | 0.0057601 |
| ENSG00000138496 | PARP9 | 11.106833 | 11.916292 | 0.8094592 | 0.0001308 | 0.0020847 |
| ENSG00000113583 | C5orf15 | 9.9545857 | 10.763378 | 0.8087921 | 1.24E-09 | 8.75E-08 |
| ENSG00000184900 | SUMO3 | 11.424347 | 12.232555 | 0.8082083 | 0.0005678 | 0.0070694 |
| ENSG00000165915 | SLC39A13 | 9.158741 | 9.9666207 | 0.8078797 | 0.0003205 | 0.0044158 |
| ENSG00000081237 | PTPRC | 14.099146 | 14.904961 | 0.8058149 | 1.56E-11 | 1.66E-09 |
| ENSG00000085514 | PILRA | 12.395338 | 13.201059 | 0.8057206 | 9.30E-13 | 1.28E-10 |
| ENSG00000141298 | SSH2 | 11.444199 | 12.249795 | 0.805596 | 1.85E-06 | 5.47E-05 |
| ENSG00000163602 | RYBP | 9.854014 | 10.659443 | 0.8054287 | 2.46E-09 | 1.58E-07 |
| ENSG00000163624 | CDS1 | 7.7527205 | 8.5573244 | 0.8046039 | 0.0007407 | 0.0087692 |
| ENSG00000008311 | AASS | 5.6550733 | 6.4589708 | 0.8038975 | 0.001702 | 0.0170193 |
| ENSG00000112297 | CRYBG1 | 12.457551 | 13.261139 | 0.8035883 | 0.0009727 | 0.0108569 |
| ENSG00000133657 | ATP13A3 | 12.76147 | 13.56462 | 0.8031501 | 0.00013 | 0.0020785 |
| ENSG00000198668 | CALM1 | 12.66169 | 13.464455 | 0.8027651 | 0.0003766 | 0.005053 |
| ENSG00000105383 | CD33 | 9.9653315 | 10.767781 | 0.8024498 | 5.77E-06 | 0.000145 |
| ENSG00000174442 | ZWILCH | 7.3919734 | 8.1943537 | 0.8023804 | 0.0004874 | 0.0062206 |
| ENSG00000104343 | UBE2W | 10.010185 | 10.812123 | 0.8019376 | 1.09E-05 | 0.0002508 |
| ENSG00000162636 | FAM102B | 10.867546 | 11.66896 | 0.8014142 | 2.06E-14 | 3.79E-12 |
| ENSG00000203812 | HIST2H2AA3 | 9.3380404 | 10.139448 | 0.8014078 | 1.39E-05 | 0.0003096 |
| ENSG00000112419 | PHACTR2 | 11.310458 | 12.111218 | 0.8007604 | 3.02E-06 | 8.36E-05 |
| ENSG00000185262 | UBALD2 | 9.0149012 | 9.8153902 | 0.800489 | 3.44E-05 | 0.0006753 |
| ENSG00000159388 | BTG2 | 11.506343 | 12.305601 | 0.7992576 | 0.0007051 | 0.0084201 |
| ENSG00000103528 | SYT17 | 8.4109004 | 9.2092832 | 0.7983828 | 0.0013939 | 0.0145237 |
| ENSG00000272196 | HIST2H2AA4 | 9.3382207 | 10.136171 | 0.7979499 | 1.52E-05 | 0.0003343 |
| ENSG00000136630 | HLX | 9.3246872 | 10.121771 | 0.7970837 | 0.0002842 | 0.0040001 |
| ENSG00000134802 | SLC43A3 | 11.646439 | 12.442972 | 0.7965328 | 1.12E-10 | 1.03E-08 |
| ENSG00000128872 | TMOD2 | 8.846527 | 9.6428418 | 0.7963148 | 0.0003853 | 0.005146 |
| ENSG00000116663 | FBXO6 | 7.8896515 | 8.682942 | 0.7932905 | 0.0001885 | 0.0028454 |
| ENSG00000165312 | OTUD1 | 9.6344678 | 10.427634 | 0.7931658 | 1.32E-06 | 4.07E-05 |
| ENSG00000004478 | FKBP4 | 11.397504 | 12.187378 | 0.7898736 | 1.94E-06 | 5.69E-05 |
| ENSG00000158869 | FCER1G | 12.999743 | 13.788593 | 0.7888498 | 5.91E-07 | 2.03E-05 |
| ENSG00000137364 | TPMT | 10.188247 | 10.974533 | 0.7862861 | 1.32E-06 | 4.07E-05 |
| ENSG00000113532 | ST8SIA4 | 10.903644 | 11.689836 | 0.7861919 | 0.0001481 | 0.0023259 |
| ENSG00000241258 | CRCP | 8.4548486 | 9.2403514 | 0.7855028 | 0.001092 | 0.0119185 |
| ENSG00000155629 | PIK3AP1 | 14.412208 | 15.194824 | 0.7826156 | 3.65E-17 | 1.05E-14 |
| ENSG00000168297 | PXK | 10.424644 | 11.206726 | 0.7820824 | 4.84E-10 | 3.76E-08 |
| ENSG00000177666 | PNPLA2 | 9.7224414 | 10.50205 | 0.7796091 | 0.000105 | 0.0017429 |
| ENSG00000116514 | RNF19B | 10.905214 | 11.683273 | 0.7780589 | 1.24E-05 | 0.0002803 |
| ENSG00000197965 | MPZL1 | 9.9767171 | 10.75389 | 0.7771728 | 0.0008008 | 0.0093169 |
| ENSG00000134851 | TMEM165 | 11.141838 | 11.91895 | 0.7771112 | 1.41E-16 | 3.71E-14 |
| ENSG00000197405 | C5AR1 | 12.842463 | 13.619393 | 0.7769302 | 0.0007286 | 0.008655 |
| ENSG00000125454 | SLC25A19 | 9.7932643 | 10.568966 | 0.7757022 | 1.28E-05 | 0.0002887 |
| ENSG00000109436 | TBC1D9 | 12.142674 | 12.917284 | 0.7746099 | 1.04E-07 | 4.51E-06 |
| ENSG00000151151 | IPMK | 10.27061 | 11.044117 | 0.7735074 | 7.55E-11 | 7.16E-09 |
| ENSG00000103811 | CTSH | 13.04923 | 13.822558 | 0.7733278 | 5.59E-07 | 1.95E-05 |
| ENSG00000147536 | GINS4 | 6.2026817 | 6.9758859 | 0.7732042 | 0.0037718 | 0.0318148 |
| ENSG00000010244 | ZNF207 | 12.444906 | 13.217388 | 0.7724819 | 8.55E-10 | 6.31E-08 |
| ENSG00000159128 | IFNGR2 | 12.263671 | 13.034414 | 0.7707438 | 1.77E-08 | 9.29E-07 |
| ENSG00000134046 | MBD2 | 11.894751 | 12.665013 | 0.7702614 | 0.0008186 | 0.0094762 |
| ENSG00000113356 | POLR3G | 5.4984632 | 6.2685833 | 0.7701201 | 8.96E-06 | 0.0002128 |
| ENSG00000135842 | FAM129A | 11.026429 | 11.796157 | 0.7697276 | 0.0002565 | 0.0036877 |
| ENSG00000156313 | RPGR | 8.3795494 | 9.1488439 | 0.7692945 | 1.65E-09 | 1.12E-07 |
| ENSG00000111726 | CMAS | 9.2036463 | 9.9720812 | 0.7684349 | 3.00E-07 | 1.13E-05 |
| ENSG00000242616 | GNG10 | 9.9732383 | 10.739886 | 0.7666482 | 4.68E-09 | 2.81E-07 |
| ENSG00000028116 | VRK2 | 9.2686546 | 10.033737 | 0.7650829 | 2.36E-06 | 6.75E-05 |
| ENSG00000145779 | TNFAIP8 | 10.211835 | 10.976844 | 0.7650094 | 1.02E-06 | 3.25E-05 |
| ENSG00000163430 | FSTL1 | 2.5484403 | 3.3121983 | 0.763758 | 0.0045776 | 0.0371068 |
| ENSG00000116128 | BCL9 | 7.2419573 | 8.0048894 | 0.7629321 | 0.0003955 | 0.0052524 |
| ENSG00000064666 | CNN2 | 10.071493 | 10.83395 | 0.7624575 | 2.19E-06 | 6.33E-05 |
| ENSG00000181885 | CLDN7 | 5.7710563 | 6.5334862 | 0.7624299 | 0.0007756 | 0.0090848 |
| ENSG00000133678 | TMEM254 | 7.9321171 | 8.6944168 | 0.7622998 | 0.0002993 | 0.0041706 |
| ENSG00000156642 | NPTN | 11.700156 | 12.462158 | 0.7620015 | 1.45E-05 | 0.0003212 |
| ENSG00000054967 | RELT | 11.120499 | 11.881856 | 0.7613567 | 7.09E-06 | 0.000174 |
| ENSG00000135049 | AGTPBP1 | 10.689341 | 11.450172 | 0.7608319 | 1.58E-10 | 1.37E-08 |
| ENSG00000138801 | PAPSS1 | 11.454925 | 12.21452 | 0.759595 | 5.00E-09 | 2.97E-07 |
| ENSG00000180008 | SOCS4 | 9.6953495 | 10.454843 | 0.7594937 | 0.0007491 | 0.0088381 |
| ENSG00000173950 | XXYLT1 | 7.6889605 | 8.4483302 | 0.7593697 | 1.88E-06 | 5.54E-05 |
| ENSG00000184260 | HIST2H2AC | 9.3091917 | 10.068226 | 0.759034 | 8.43E-10 | 6.23E-08 |
| ENSG00000101974 | ATP11C | 9.9661022 | 10.7224 | 0.7562981 | 4.49E-06 | 0.0001173 |
| ENSG00000105122 | RASAL3 | 7.8472232 | 8.6030065 | 0.7557833 | 0.0055709 | 0.0431124 |
| ENSG00000101888 | NXT2 | 8.3031751 | 9.0587819 | 0.7556068 | 2.33E-05 | 0.0004825 |
| ENSG00000175066 | GK5 | 9.1579735 | 9.9128982 | 0.7549247 | 0.0002075 | 0.0030842 |
| ENSG00000176171 | BNIP3 | 9.618393 | 10.373189 | 0.7547964 | 2.48E-05 | 0.0005087 |
| ENSG00000169398 | PTK2 | 8.3115695 | 9.0656094 | 0.7540399 | 0.0007712 | 0.0090421 |
| ENSG00000172081 | MOB3A | 11.402789 | 12.156207 | 0.7534181 | 3.85E-07 | 1.41E-05 |
| ENSG00000012061 | ERCC1 | 9.8954727 | 10.648835 | 0.7533626 | 2.59E-06 | 7.32E-05 |
| ENSG00000078687 | TNRC6C | 8.7706826 | 9.5230407 | 0.7523581 | 3.61E-06 | 9.74E-05 |
| ENSG00000028839 | TBPL1 | 7.0268799 | 7.7791543 | 0.7522744 | 4.87E-06 | 0.0001257 |
| ENSG00000081014 | AP4E1 | 9.754399 | 10.50484 | 0.7504408 | 4.64E-05 | 0.0008711 |
| ENSG00000142192 | APP | 12.743376 | 13.492792 | 0.7494155 | 5.41E-06 | 0.000137 |
| ENSG00000107551 | RASSF4 | 12.8026 | 13.551671 | 0.7490709 | 1.95E-07 | 7.82E-06 |
| ENSG00000152213 | ARL11 | 9.8784159 | 10.627342 | 0.7489258 | 9.10E-14 | 1.52E-11 |
| ENSG00000166845 | C18orf54 | 6.6140145 | 7.3625685 | 0.748554 | 0.0015405 | 0.0157451 |
| ENSG00000120742 | SERP1 | 10.948955 | 11.697394 | 0.7484388 | 9.71E-05 | 0.0016293 |
| ENSG00000145088 | EAF2 | 7.7696397 | 8.5178511 | 0.7482114 | 1.95E-06 | 5.72E-05 |
| ENSG00000072401 | UBE2D1 | 10.700053 | 11.447967 | 0.7479143 | 1.59E-05 | 0.000349 |
| ENSG00000147454 | SLC25A37 | 8.3273928 | 9.0743803 | 0.7469875 | 0.0001496 | 0.0023426 |
| ENSG00000127314 | RAP1B | 12.399378 | 13.144351 | 0.7449734 | 1.98E-05 | 0.0004222 |
| ENSG00000196396 | PTPN1 | 11.903502 | 12.646088 | 0.7425869 | 1.59E-07 | 6.56E-06 |
| ENSG00000106546 | AHR | 12.356106 | 13.098682 | 0.7425763 | 1.62E-05 | 0.0003536 |
| ENSG00000271605 | MILR1 | 9.8818755 | 10.622955 | 0.7410797 | 1.07E-05 | 0.0002488 |
| ENSG00000091073 | DTX2 | 10.188372 | 10.92805 | 0.7396776 | 1.97E-07 | 7.86E-06 |
| ENSG00000030419 | IKZF2 | 6.3752389 | 7.1136302 | 0.7383913 | 0.0012198 | 0.0130283 |
| ENSG00000171314 | PGAM1 | 11.505415 | 12.24372 | 0.738305 | 2.55E-08 | 1.29E-06 |
| ENSG00000136810 | TXN | 12.763491 | 13.501395 | 0.7379039 | 0.0035002 | 0.0300436 |
| ENSG00000197147 | LRRC8B | 9.2513379 | 9.988837 | 0.7374991 | 1.62E-05 | 0.0003533 |
| ENSG00000104812 | GYS1 | 10.773707 | 11.509255 | 0.7355477 | 3.20E-07 | 1.20E-05 |
| ENSG00000153094 | BCL2L11 | 8.3625381 | 9.0974098 | 0.7348717 | 0.0018541 | 0.018312 |
| ENSG00000080200 | CRYBG3 | 11.811775 | 12.546052 | 0.7342764 | 9.00E-10 | 6.57E-08 |
| ENSG00000170027 | YWHAG | 13.027818 | 13.761518 | 0.7336999 | 8.28E-10 | 6.16E-08 |
| ENSG00000074370 | ATP2A3 | 10.171573 | 10.904762 | 0.7331888 | 0.0047373 | 0.0380569 |
| ENSG00000213390 | ARHGAP19 | 8.9300721 | 9.6632254 | 0.7331533 | 2.24E-05 | 0.0004678 |
| ENSG00000197170 | PSMD12 | 10.468467 | 11.20143 | 0.7329635 | 3.00E-10 | 2.45E-08 |
| ENSG00000162129 | CLPB | 10.860367 | 11.591862 | 0.7314948 | 1.27E-09 | 8.92E-08 |
| ENSG00000081041 | CXCL2 | 9.9353783 | 10.666196 | 0.7308179 | 0.0028076 | 0.0253962 |
| ENSG00000168769 | TET2 | 12.202137 | 12.928064 | 0.7259274 | 2.75E-05 | 0.0005544 |
| ENSG00000148690 | FRA10AC1 | 8.1889929 | 8.9149172 | 0.7259243 | 0.0002841 | 0.0040001 |
| ENSG00000139629 | GALNT6 | 10.792125 | 11.517219 | 0.725094 | 1.17E-10 | 1.05E-08 |
| ENSG00000139645 | ANKRD52 | 11.260573 | 11.985583 | 0.7250104 | 0.0049871 | 0.0395716 |
| ENSG00000165704 | HPRT1 | 8.6078394 | 9.3326483 | 0.7248088 | 0.0001508 | 0.0023599 |
| ENSG00000109771 | LRP2BP | 6.0611503 | 6.7855851 | 0.7244347 | 0.004932 | 0.0392621 |
| ENSG00000172243 | CLEC7A | 11.956393 | 12.679809 | 0.7234162 | 1.23E-05 | 0.000278 |
| ENSG00000002933 | TMEM176A | 11.041232 | 11.763892 | 0.7226604 | 0.004782 | 0.0383274 |
| ENSG00000144228 | SPOPL | 9.605647 | 10.328217 | 0.7225701 | 0.0005891 | 0.0072833 |
| ENSG00000066405 | CLDN18 | 6.0358089 | 6.7571181 | 0.7213092 | 0.0023898 | 0.0223178 |
| ENSG00000120519 | SLC10A7 | 8.7297065 | 9.4497719 | 0.7200655 | 9.15E-07 | 2.97E-05 |
| ENSG00000116815 | CD58 | 10.225883 | 10.945375 | 0.7194928 | 0.0006039 | 0.0074133 |
| ENSG00000132589 | FLOT2 | 10.958151 | 11.676591 | 0.7184408 | 0.0001732 | 0.0026548 |
| ENSG00000188641 | DPYD | 12.384652 | 13.10271 | 0.7180577 | 2.23E-09 | 1.46E-07 |
| ENSG00000178980 | SELENOW | 9.2221979 | 9.9396386 | 0.7174407 | 0.000303 | 0.0042115 |
| ENSG00000177272 | KCNA3 | 7.6739458 | 8.389823 | 0.7158773 | 0.0010028 | 0.0111219 |
| ENSG00000008323 | PLEKHG6 | 6.4811543 | 7.196557 | 0.7154027 | 0.0004314 | 0.0056262 |
| ENSG00000068024 | HDAC4 | 10.122554 | 10.837415 | 0.7148605 | 1.70E-07 | 6.97E-06 |
| ENSG00000169379 | ARL13B | 7.87009 | 8.5848369 | 0.714747 | 2.71E-06 | 7.60E-05 |
| ENSG00000154783 | FGD5 | 9.1941481 | 9.9085729 | 0.7144249 | 0.0053254 | 0.0416756 |
| ENSG00000138835 | RGS3 | 8.0115116 | 8.7250617 | 0.7135501 | 8.42E-05 | 0.0014452 |
| ENSG00000233087 | WTH3DI | 4.6045609 | 5.3181072 | 0.7135463 | 0.0041154 | 0.0340717 |
| ENSG00000173846 | PLK3 | 9.6922736 | 10.405778 | 0.7135042 | 0.0003793 | 0.0050763 |
| ENSG00000155975 | VPS37A | 9.9109982 | 10.624221 | 0.7132233 | 3.71E-05 | 0.0007207 |
| ENSG00000130052 | STARD8 | 9.8712311 | 10.582966 | 0.7117345 | 8.92E-05 | 0.0015144 |
| ENSG00000113441 | LNPEP | 12.338066 | 13.049783 | 0.7117164 | 6.05E-10 | 4.63E-08 |
| ENSG00000107960 | STN1 | 7.5016251 | 8.2133257 | 0.7117007 | 0.0010471 | 0.0115233 |
| ENSG00000160584 | SIK3 | 11.112778 | 11.824462 | 0.7116842 | 1.11E-05 | 0.0002544 |
| ENSG00000123700 | KCNJ2 | 8.4514894 | 9.1610815 | 0.7095921 | 0.005102 | 0.0402797 |
| ENSG00000065357 | DGKA | 8.808505 | 9.515179 | 0.7066739 | 0.0031208 | 0.0275798 |
| ENSG00000130449 | ZSWIM6 | 11.487985 | 12.19268 | 0.7046945 | 1.56E-07 | 6.46E-06 |
| ENSG00000158161 | EYA3 | 10.977239 | 11.681126 | 0.7038875 | 1.44E-06 | 4.38E-05 |
| ENSG00000075223 | SEMA3C | 10.142797 | 10.845719 | 0.7029216 | 2.49E-06 | 7.09E-05 |
| ENSG00000117533 | VAMP4 | 9.6276091 | 10.330084 | 0.7024745 | 2.08E-06 | 6.05E-05 |
| ENSG00000117009 | KMO | 9.9342085 | 10.636587 | 0.7023781 | 0.0011564 | 0.012503 |
| ENSG00000005059 | MCUB | 8.7872916 | 9.489255 | 0.7019635 | 0.0002952 | 0.0041229 |
| ENSG00000158019 | BABAM2 | 9.8259565 | 10.527013 | 0.7010569 | 6.60E-12 | 7.60E-10 |
| ENSG00000180353 | HCLS1 | 12.767192 | 13.467975 | 0.7007829 | 4.46E-05 | 0.0008416 |
| ENSG00000178719 | GRINA | 11.677628 | 12.377679 | 0.7000515 | 4.45E-06 | 0.0001164 |
| ENSG00000130766 | SESN2 | 8.6475275 | 9.3470563 | 0.6995288 | 0.0018282 | 0.0180913 |
| ENSG00000168264 | IRF2BP2 | 11.55841 | 12.257012 | 0.698602 | 5.06E-05 | 0.0009346 |
| ENSG00000189308 | LIN54 | 9.7510485 | 10.449398 | 0.6983493 | 7.80E-05 | 0.001356 |
| ENSG00000143119 | CD53 | 12.588648 | 13.286915 | 0.6982673 | 2.30E-06 | 6.59E-05 |
| ENSG00000140577 | CRTC3 | 11.186838 | 11.884741 | 0.6979033 | 2.71E-13 | 4.10E-11 |
| ENSG00000186818 | LILRB4 | 13.531418 | 14.228728 | 0.69731 | 7.29E-06 | 0.0001782 |
| ENSG00000145331 | TRMT10A | 6.1723558 | 6.8693956 | 0.6970398 | 0.0013762 | 0.0143564 |
| ENSG00000087586 | AURKA | 7.5369974 | 8.2339035 | 0.6969061 | 1.10E-05 | 0.0002535 |
| ENSG00000122591 | FAM126A | 9.4003646 | 10.097108 | 0.6967437 | 0.00098 | 0.0109073 |
| ENSG00000144848 | ATG3 | 10.902301 | 11.59752 | 0.6952189 | 8.57E-10 | 6.31E-08 |
| ENSG00000155657 | TTN | 11.054525 | 11.749399 | 0.6948746 | 0.0001005 | 0.0016775 |
| ENSG00000134686 | PHC2 | 11.091369 | 11.785315 | 0.693946 | 2.99E-06 | 8.30E-05 |
| ENSG00000162664 | ZNF326 | 9.2474642 | 9.9411004 | 0.6936362 | 1.55E-05 | 0.0003417 |
| ENSG00000173598 | NUDT4 | 9.2089207 | 9.9003535 | 0.6914328 | 0.0009244 | 0.0104316 |
| ENSG00000178695 | KCTD12 | 14.195787 | 14.886736 | 0.690949 | 2.06E-05 | 0.0004365 |
| ENSG00000167613 | LAIR1 | 12.387515 | 13.078219 | 0.6907044 | 0.0004521 | 0.0058512 |
| ENSG00000095209 | TMEM38B | 9.1860982 | 9.8766499 | 0.6905517 | 0.0002731 | 0.0038764 |
| ENSG00000166188 | ZNF319 | 8.8686665 | 9.5591738 | 0.6905074 | 8.68E-06 | 0.0002069 |
| ENSG00000131236 | CAP1 | 13.244727 | 13.935146 | 0.6904191 | 1.86E-10 | 1.59E-08 |
| ENSG00000121350 | PYROXD1 | 9.8256155 | 10.51508 | 0.6894649 | 6.67E-05 | 0.0011887 |
| ENSG00000168404 | MLKL | 10.037673 | 10.725066 | 0.6873934 | 1.78E-05 | 0.0003835 |
| ENSG00000196712 | NF1 | 12.397671 | 13.084884 | 0.6872132 | 3.45E-16 | 8.74E-14 |
| ENSG00000033170 | FUT8 | 8.8887823 | 9.5753305 | 0.6865483 | 0.000465 | 0.0059913 |
| ENSG00000144597 | EAF1 | 11.101019 | 11.787281 | 0.6862621 | 1.16E-05 | 0.0002647 |
| ENSG00000167470 | MIDN | 10.386326 | 11.070766 | 0.6844405 | 0.0004214 | 0.0055194 |
| ENSG00000184922 | FMNL1 | 12.388304 | 13.07231 | 0.6840063 | 3.37E-05 | 0.0006637 |
| ENSG00000163823 | CCR1 | 13.42534 | 14.10833 | 0.6829897 | 1.40E-11 | 1.51E-09 |
| ENSG00000255823 | MTRNR2L8 | 7.3511218 | 8.0339818 | 0.68286 | 0.0008686 | 0.0099404 |
| ENSG00000155307 | SAMSN1 | 11.7193 | 12.402159 | 0.6828595 | 0.0010133 | 0.0112219 |
| ENSG00000244687 | UBE2V1 | 10.878601 | 11.561292 | 0.6826908 | 0.0009169 | 0.0103765 |
| ENSG00000104689 | TNFRSF10A | 8.915688 | 9.5973391 | 0.6816511 | 0.000972 | 0.0108516 |
| ENSG00000197694 | SPTAN1 | 13.419079 | 14.100721 | 0.6816421 | 2.00E-07 | 7.99E-06 |
| ENSG00000188157 | AGRN | 10.612775 | 11.293285 | 0.6805103 | 0.0028225 | 0.025482 |
| ENSG00000135766 | EGLN1 | 10.168926 | 10.848988 | 0.6800621 | 1.78E-05 | 0.0003835 |
| ENSG00000070540 | WIPI1 | 10.517565 | 11.197544 | 0.679979 | 1.59E-05 | 0.0003479 |
| ENSG00000125885 | MCM8 | 7.2939858 | 7.973387 | 0.6794012 | 0.0009773 | 0.0108883 |
| ENSG00000091106 | NLRC4 | 10.053397 | 10.731892 | 0.6784949 | 2.49E-05 | 0.0005098 |
| ENSG00000164506 | STXBP5 | 11.003713 | 11.681945 | 0.6782316 | 0.0001233 | 0.0019887 |
| ENSG00000132436 | FIGNL1 | 7.9298424 | 8.6079591 | 0.6781167 | 6.24E-07 | 2.13E-05 |
| ENSG00000124222 | STX16 | 10.24846 | 10.925892 | 0.6774322 | 0.0062248 | 0.0469802 |
| ENSG00000104093 | DMXL2 | 15.229916 | 15.907165 | 0.6772487 | 5.16E-07 | 1.82E-05 |
| ENSG00000135365 | PHF21A | 10.608605 | 11.28553 | 0.6769248 | 0.0014932 | 0.0153467 |
| ENSG00000101350 | KIF3B | 10.137897 | 10.813924 | 0.676027 | 7.94E-07 | 2.64E-05 |
| ENSG00000103494 | RPGRIP1L | 5.7888484 | 6.464333 | 0.6754847 | 0.001171 | 0.0126289 |
| ENSG00000058091 | CDK14 | 9.6693471 | 10.34437 | 0.6750227 | 0.006236 | 0.0470561 |
| ENSG00000198860 | TSEN15 | 8.4972762 | 9.1714113 | 0.6741351 | 0.0002738 | 0.0038832 |
| ENSG00000157625 | TAB3 | 10.934003 | 11.607646 | 0.6736437 | 6.94E-05 | 0.001228 |
| ENSG00000198898 | CAPZA2 | 11.789815 | 12.462559 | 0.6727446 | 0.0001967 | 0.0029446 |
| ENSG00000184009 | ACTG1 | 14.643503 | 15.316114 | 0.6726112 | 0.0006102 | 0.0074717 |
| ENSG00000270276 | HIST2H4B | 6.3781476 | 7.049742 | 0.6715944 | 0.0066207 | 0.0492149 |
| ENSG00000087253 | LPCAT2 | 10.441036 | 11.1124 | 0.6713644 | 3.17E-05 | 0.0006282 |
| ENSG00000188906 | LRRK2 | 11.945988 | 12.617302 | 0.671314 | 1.68E-05 | 0.0003657 |
| ENSG00000259330 | INAFM2 | 10.302137 | 10.973412 | 0.6712758 | 1.22E-09 | 8.66E-08 |
| ENSG00000169991 | IFFO2 | 7.72112 | 8.3919721 | 0.6708522 | 0.0051408 | 0.0405344 |
| ENSG00000102879 | CORO1A | 10.576816 | 11.247251 | 0.6704348 | 0.0033366 | 0.0289509 |
| ENSG00000131378 | RFTN1 | 10.481313 | 11.149469 | 0.6681563 | 0.0009248 | 0.0104337 |
| ENSG00000137628 | DDX60 | 9.3828709 | 10.050887 | 0.6680159 | 0.0025392 | 0.0234311 |
| ENSG00000196230 | TUBB | 14.088613 | 14.75638 | 0.6677672 | 0.0011078 | 0.0120698 |
| ENSG00000166839 | ANKDD1A | 8.4996394 | 9.1665177 | 0.6668783 | 0.0007218 | 0.0085961 |
| ENSG00000127952 | STYXL1 | 8.4895374 | 9.1563894 | 0.666852 | 1.28E-07 | 5.42E-06 |
| ENSG00000196954 | CASP4 | 11.047484 | 11.713786 | 0.6663015 | 6.89E-10 | 5.17E-08 |
| ENSG00000166664 | CHRFAM7A | 7.3732994 | 8.0395959 | 0.6662965 | 0.0002952 | 0.0041229 |
| ENSG00000089195 | TRMT6 | 7.9343783 | 8.6002342 | 0.6658559 | 0.0001754 | 0.0026805 |
| ENSG00000196792 | STRN3 | 9.9090863 | 10.57491 | 0.6658241 | 7.60E-11 | 7.19E-09 |
| ENSG00000166446 | CDYL2 | 10.209707 | 10.873696 | 0.6639893 | 1.07E-06 | 3.37E-05 |
| ENSG00000173926 | MARCHF3 | 6.6599951 | 7.3221828 | 0.6621878 | 0.0005336 | 0.0067056 |
| ENSG00000239282 | CASTOR1 | 6.18221 | 6.8439362 | 0.6617262 | 0.0062582 | 0.0471752 |
| ENSG00000182578 | CSF1R | 14.383394 | 15.044688 | 0.6612933 | 0.0002142 | 0.0031662 |
| ENSG00000104671 | DCTN6 | 8.7724127 | 9.433494 | 0.6610814 | 0.0002092 | 0.003104 |
| ENSG00000213190 | MLLT11 | 8.9403438 | 9.6007561 | 0.6604123 | 1.20E-06 | 3.72E-05 |
| ENSG00000181036 | FCRL6 | 6.3517931 | 7.0119008 | 0.6601076 | 0.002492 | 0.0230684 |
| ENSG00000123983 | ACSL3 | 11.535934 | 12.195895 | 0.6599609 | 3.16E-06 | 8.67E-05 |
| ENSG00000171310 | CHST11 | 12.802332 | 13.462131 | 0.659799 | 0.0001039 | 0.0017274 |
| ENSG00000077232 | DNAJC10 | 11.721091 | 12.380674 | 0.6595832 | 1.64E-07 | 6.73E-06 |
| ENSG00000082213 | C5orf22 | 9.5628266 | 10.221789 | 0.6589626 | 4.19E-07 | 1.51E-05 |
| ENSG00000153250 | RBMS1 | 11.641485 | 12.300094 | 0.6586091 | 2.70E-05 | 0.0005454 |
| ENSG00000140564 | FURIN | 11.730886 | 12.388268 | 0.6573817 | 0.0003612 | 0.0048878 |
| ENSG00000152422 | XRCC4 | 8.3874131 | 9.0446684 | 0.6572553 | 9.81E-07 | 3.15E-05 |
| ENSG00000151748 | SAV1 | 9.3612576 | 10.018163 | 0.6569058 | 8.56E-07 | 2.81E-05 |
| ENSG00000026103 | FAS | 7.5957699 | 8.2522603 | 0.6564904 | 0.0045223 | 0.0367813 |
| ENSG00000005189 | REXO5 | 5.9978324 | 6.6535628 | 0.6557304 | 0.0007862 | 0.0091816 |
| ENSG00000169220 | RGS14 | 8.1309756 | 8.786352 | 0.6553764 | 0.0051424 | 0.0405396 |
| ENSG00000125505 | MBOAT7 | 10.055386 | 10.709633 | 0.6542466 | 2.17E-06 | 6.30E-05 |
| ENSG00000086065 | CHMP5 | 10.350995 | 11.005192 | 0.6541973 | 5.26E-07 | 1.84E-05 |
| ENSG00000126458 | RRAS | 9.2936097 | 9.9468035 | 0.6531937 | 6.79E-05 | 0.0012057 |
| ENSG00000100911 | PSME2 | 10.301141 | 10.954088 | 0.6529473 | 0.0003122 | 0.0043143 |
| ENSG00000163947 | ARHGEF3 | 9.9044027 | 10.557293 | 0.6528906 | 0.0016082 | 0.0162883 |
| ENSG00000140280 | LYSMD2 | 6.8416744 | 7.4937379 | 0.6520635 | 0.0034373 | 0.0296368 |
| ENSG00000196659 | TTC30B | 6.9400358 | 7.5917871 | 0.6517514 | 5.19E-06 | 0.0001325 |
| ENSG00000111145 | ELK3 | 9.966761 | 10.616973 | 0.6502117 | 0.0009641 | 0.0107857 |
| ENSG00000032444 | PNPLA6 | 12.305524 | 12.95417 | 0.6486465 | 7.46E-06 | 0.0001815 |
| ENSG00000078177 | N4BP2 | 9.7792412 | 10.427448 | 0.648207 | 2.64E-07 | 1.01E-05 |
| ENSG00000148288 | GBGT1 | 9.1919452 | 9.8400836 | 0.6481385 | 5.90E-11 | 5.68E-09 |
| ENSG00000106692 | FKTN | 8.3079182 | 8.9559478 | 0.6480296 | 0.0047323 | 0.0380241 |
| ENSG00000217555 | CKLF | 8.1266631 | 8.7745541 | 0.647891 | 6.37E-07 | 2.17E-05 |
| ENSG00000186174 | BCL9L | 10.701518 | 11.349196 | 0.6476788 | 0.0019143 | 0.0187995 |
| ENSG00000092929 | UNC13D | 10.819809 | 11.462965 | 0.6431562 | 0.0007339 | 0.0086989 |
| ENSG00000095303 | PTGS1 | 11.367222 | 12.010206 | 0.6429834 | 4.41E-05 | 0.0008325 |
| ENSG00000116489 | CAPZA1 | 12.517781 | 13.15941 | 0.6416294 | 2.99E-06 | 8.30E-05 |
| ENSG00000144746 | ARL6IP5 | 12.678745 | 13.320097 | 0.6413513 | 1.49E-07 | 6.22E-06 |
| ENSG00000108666 | C17orf75 | 6.1227963 | 6.7635429 | 0.6407465 | 0.0012734 | 0.0134989 |
| ENSG00000168268 | NT5DC2 | 9.3837013 | 10.023283 | 0.6395814 | 0.0011208 | 0.0121751 |
| ENSG00000204389 | HSPA1A | 12.198031 | 12.837587 | 0.6395559 | 0.0049392 | 0.0392975 |
| ENSG00000185650 | ZFP36L1 | 13.025822 | 13.66521 | 0.6393878 | 0.0011685 | 0.0126088 |
| ENSG00000100504 | PYGL | 12.096486 | 12.735689 | 0.6392037 | 0.0003057 | 0.0042385 |
| ENSG00000185010 | F8 | 7.2166088 | 7.855671 | 0.6390622 | 0.0058528 | 0.0447183 |
| ENSG00000186352 | ANKRD37 | 6.8189484 | 7.4569208 | 0.6379724 | 0.0004456 | 0.0057804 |
| ENSG00000128245 | YWHAH | 11.210225 | 11.84812 | 0.6378947 | 0.0001732 | 0.0026548 |
| ENSG00000115963 | RND3 | 8.3106497 | 8.9483544 | 0.6377047 | 0.0050113 | 0.0397346 |
| ENSG00000204116 | CHIC1 | 6.6810924 | 7.3170442 | 0.6359518 | 0.006336 | 0.0476551 |
| ENSG00000131037 | EPS8L1 | 5.8549393 | 6.490773 | 0.6358338 | 0.0025875 | 0.0237918 |
| ENSG00000106392 | C1GALT1 | 9.1318718 | 9.7668559 | 0.6349842 | 0.0007489 | 0.0088375 |
| ENSG00000115392 | FANCL | 7.2333618 | 7.8673422 | 0.6339804 | 0.0006548 | 0.0078992 |
| ENSG00000175105 | ZNF654 | 10.008081 | 10.64013 | 0.6320497 | 5.58E-05 | 0.001019 |
| ENSG00000144566 | RAB5A | 10.855532 | 11.486899 | 0.6313669 | 4.47E-05 | 0.0008424 |
| ENSG00000035687 | ADSS | 10.235041 | 10.866139 | 0.6310986 | 1.99E-05 | 0.0004237 |
| ENSG00000138071 | ACTR2 | 13.868237 | 14.499233 | 0.6309959 | 7.35E-07 | 2.46E-05 |
| ENSG00000144161 | ZC3H8 | 7.1470135 | 7.7768916 | 0.6298781 | 0.001369 | 0.014312 |
| ENSG00000125772 | GPCPD1 | 11.918984 | 12.548367 | 0.6293825 | 0.0047121 | 0.0378966 |
| ENSG00000111679 | PTPN6 | 12.402767 | 13.031657 | 0.6288899 | 9.51E-07 | 3.07E-05 |
| ENSG00000077238 | IL4R | 10.898922 | 11.527794 | 0.6288713 | 2.75E-05 | 0.0005554 |
| ENSG00000012174 | MBTPS2 | 9.1588076 | 9.7869836 | 0.628176 | 0.0053486 | 0.0417754 |
| ENSG00000166002 | SMCO4 | 7.7463183 | 8.3735572 | 0.6272389 | 0.0011199 | 0.0121687 |
| ENSG00000143702 | CEP170 | 11.793877 | 12.420807 | 0.6269296 | 2.13E-09 | 1.40E-07 |
| ENSG00000106829 | TLE4 | 10.242174 | 10.868992 | 0.626818 | 6.67E-13 | 9.54E-11 |
| ENSG00000132669 | RIN2 | 12.219149 | 12.845766 | 0.6266168 | 0.0005981 | 0.007364 |
| ENSG00000076641 | PAG1 | 11.606854 | 12.233399 | 0.6265449 | 7.84E-06 | 0.0001891 |
| ENSG00000151876 | FBXO4 | 7.1499214 | 7.7762785 | 0.626357 | 4.72E-08 | 2.23E-06 |
| ENSG00000163297 | ANTXR2 | 11.669221 | 12.295344 | 0.626123 | 0.0016957 | 0.0169634 |
| ENSG00000136933 | RABEPK | 8.394588 | 9.0206539 | 0.6260659 | 0.0041186 | 0.0340851 |
| ENSG00000188994 | ZNF292 | 11.520771 | 12.144809 | 0.6240384 | 7.30E-05 | 0.0012839 |
| ENSG00000235910 | APOA1-AS | 5.074194 | 5.6979316 | 0.6237376 | 0.0038127 | 0.0320857 |
| ENSG00000054282 | SDCCAG8 | 10.533142 | 11.156152 | 0.6230095 | 1.00E-07 | 4.37E-06 |
| ENSG00000140853 | NLRC5 | 10.261861 | 10.882061 | 0.6201999 | 0.0001787 | 0.0027205 |
| ENSG00000148110 | MFSD14B | 11.471849 | 12.090207 | 0.6183578 | 4.95E-08 | 2.33E-06 |
| ENSG00000133704 | IPO8 | 11.372512 | 11.99003 | 0.6175173 | 1.14E-10 | 1.04E-08 |
| ENSG00000170017 | ALCAM | 12.713825 | 13.331174 | 0.6173487 | 0.0002796 | 0.0039517 |
| ENSG00000170464 | DNAJC18 | 6.9819254 | 7.5986721 | 0.6167467 | 0.0001288 | 0.0020622 |
| ENSG00000128708 | HAT1 | 9.5420542 | 10.158359 | 0.6163043 | 7.90E-09 | 4.49E-07 |
| ENSG00000188827 | SLX4 | 9.7235398 | 10.339309 | 0.6157695 | 1.93E-06 | 5.67E-05 |
| ENSG00000115520 | COQ10B | 9.3516745 | 9.9670135 | 0.615339 | 5.50E-08 | 2.56E-06 |
| ENSG00000109756 | RAPGEF2 | 11.471939 | 12.086515 | 0.6145759 | 1.33E-09 | 9.25E-08 |
| ENSG00000140307 | GTF2A2 | 8.7212767 | 9.3358123 | 0.6145356 | 0.002303 | 0.021713 |
| ENSG00000198554 | WDHD1 | 7.6206162 | 8.2342534 | 0.6136372 | 0.0005123 | 0.0064775 |
| ENSG00000176155 | CCDC57 | 10.495344 | 11.108855 | 0.6135117 | 0.0010184 | 0.0112613 |
| ENSG00000141503 | MINK1 | 11.963369 | 12.576065 | 0.6126958 | 7.78E-05 | 0.0013535 |
| ENSG00000178694 | NSUN3 | 9.7793905 | 10.391472 | 0.6120812 | 7.37E-05 | 0.0012933 |
| ENSG00000145685 | LHFPL2 | 13.764885 | 14.374911 | 0.6100258 | 1.31E-06 | 4.05E-05 |
| ENSG00000134571 | MYBPC3 | 6.9279919 | 7.5372606 | 0.6092688 | 0.003575 | 0.0305302 |
| ENSG00000155903 | RASA2 | 10.40416 | 11.012888 | 0.6087278 | 1.62E-05 | 0.0003535 |
| ENSG00000145012 | LPP | 12.601826 | 13.21051 | 0.6086844 | 0.0063952 | 0.0479772 |
| ENSG00000108352 | RAPGEFL1 | 5.8583872 | 6.4670042 | 0.6086169 | 0.0040044 | 0.0333598 |
| ENSG00000164011 | ZNF691 | 7.4406054 | 8.0485536 | 0.6079483 | 0.0021632 | 0.0207326 |
| ENSG00000107789 | MINPP1 | 8.4314641 | 9.0392487 | 0.6077846 | 4.13E-06 | 0.0001089 |
| ENSG00000155115 | GTF3C6 | 9.5084307 | 10.115337 | 0.6069059 | 6.46E-08 | 2.96E-06 |
| ENSG00000185515 | BRCC3 | 9.213915 | 9.8205176 | 0.6066026 | 2.98E-05 | 0.0005964 |
| ENSG00000137767 | SQOR | 11.579372 | 12.185875 | 0.606502 | 9.99E-05 | 0.00167 |
| ENSG00000106991 | ENG | 12.920434 | 13.526539 | 0.6061055 | 0.001329 | 0.0139907 |
| ENSG00000115652 | UXS1 | 10.030371 | 10.635115 | 0.6047442 | 0.0001928 | 0.002897 |
| ENSG00000115091 | ACTR3 | 13.922124 | 14.526838 | 0.6047142 | 1.43E-05 | 0.0003164 |
| ENSG00000115159 | GPD2 | 11.948535 | 12.553082 | 0.6045471 | 0.0001202 | 0.0019469 |
| ENSG00000105401 | CDC37 | 10.866626 | 11.47044 | 0.6038139 | 5.03E-05 | 0.0009309 |
| ENSG00000186280 | KDM4D | 4.4993027 | 5.1030582 | 0.6037555 | 0.0031695 | 0.0279091 |
| ENSG00000163872 | YEATS2 | 10.942205 | 11.54544 | 0.6032357 | 4.30E-10 | 3.39E-08 |
| ENSG00000164167 | LSM6 | 7.8080977 | 8.41124 | 0.6031423 | 0.0013413 | 0.0140807 |
| ENSG00000166479 | TMX3 | 10.38817 | 10.990724 | 0.602554 | 2.67E-07 | 1.02E-05 |
| ENSG00000106733 | NMRK1 | 9.0818299 | 9.6842856 | 0.6024557 | 0.002693 | 0.0245816 |
| ENSG00000198804 | MT-CO1 | 17.297904 | 17.900028 | 0.6021233 | 0.0057548 | 0.0441462 |
| ENSG00000065989 | PDE4A | 10.524284 | 11.126158 | 0.6018733 | 0.0003236 | 0.0044545 |
| ENSG00000138756 | BMP2K | 13.500148 | 14.101578 | 0.6014297 | 0.00018 | 0.0027366 |
| ENSG00000165417 | GTF2A1 | 10.623371 | 11.223219 | 0.5998475 | 3.52E-09 | 2.18E-07 |
| ENSG00000136147 | PHF11 | 9.503874 | 10.103393 | 0.5995189 | 1.00E-05 | 0.0002339 |
| ENSG00000170396 | ZNF804A | 8.319083 | 8.9183774 | 0.5992943 | 0.00627 | 0.0472318 |
| ENSG00000115145 | STAM2 | 10.463314 | 11.062595 | 0.599281 | 0.0001787 | 0.0027205 |
| ENSG00000197429 | IPP | 6.5755189 | 7.1740943 | 0.5985754 | 0.0002362 | 0.003441 |
| ENSG00000143549 | TPM3 | 12.762133 | 13.360345 | 0.5982118 | 5.34E-06 | 0.0001358 |
| ENSG00000177565 | TBL1XR1 | 12.232489 | 12.830313 | 0.5978242 | 1.80E-05 | 0.0003863 |
| ENSG00000114978 | MOB1A | 13.198 | 13.795252 | 0.5972524 | 1.36E-08 | 7.30E-07 |
| ENSG00000108561 | C1QBP | 10.125241 | 10.720985 | 0.5957444 | 4.69E-05 | 0.00088 |
| ENSG00000196981 | WDR5B | 8.2466944 | 8.8422643 | 0.5955699 | 0.0001293 | 0.00207 |
| ENSG00000183726 | TMEM50A | 11.740185 | 12.335426 | 0.5952414 | 2.89E-08 | 1.44E-06 |
| ENSG00000183520 | UTP11 | 9.0971056 | 9.6917006 | 0.594595 | 5.24E-06 | 0.0001333 |
| ENSG00000102218 | RP2 | 10.392408 | 10.986362 | 0.5939542 | 1.91E-07 | 7.72E-06 |
| ENSG00000129219 | PLD2 | 9.6860709 | 10.279696 | 0.5936253 | 7.44E-05 | 0.0013032 |
| ENSG00000068697 | LAPTM4A | 12.053699 | 12.647062 | 0.593363 | 1.54E-05 | 0.0003392 |
| ENSG00000169504 | CLIC4 | 12.564138 | 13.157092 | 0.5929536 | 2.74E-05 | 0.0005524 |
| ENSG00000082212 | ME2 | 12.538996 | 13.131626 | 0.5926293 | 0.0009217 | 0.0104117 |
| ENSG00000087470 | DNM1L | 10.905738 | 11.498097 | 0.5923592 | 0.0006565 | 0.0079156 |
| ENSG00000179361 | ARID3B | 8.8334649 | 9.4254904 | 0.5920255 | 0.0019531 | 0.0190988 |
| ENSG00000073921 | PICALM | 13.287196 | 13.878048 | 0.5908528 | 4.49E-06 | 0.0001173 |
| ENSG00000119048 | UBE2B | 9.0019227 | 9.5925094 | 0.5905867 | 6.44E-06 | 0.0001603 |
| ENSG00000113368 | LMNB1 | 7.3744334 | 7.9647071 | 0.5902737 | 0.0003784 | 0.0050661 |
| ENSG00000137168 | PPIL1 | 8.1009224 | 8.6901963 | 0.5892738 | 0.0014867 | 0.0152949 |
| ENSG00000138600 | SPPL2A | 12.143042 | 12.732141 | 0.589099 | 2.21E-06 | 6.38E-05 |
| ENSG00000164924 | YWHAZ | 13.76059 | 14.34964 | 0.5890499 | 6.31E-08 | 2.90E-06 |
| ENSG00000143401 | ANP32E | 9.596828 | 10.185781 | 0.5889531 | 0.0001 | 0.0016714 |
| ENSG00000152749 | GPR180 | 8.879244 | 9.4679129 | 0.5886689 | 0.0021557 | 0.0206772 |
| ENSG00000149346 | SLX4IP | 9.1994424 | 9.7876913 | 0.5882489 | 5.14E-12 | 6.05E-10 |
| ENSG00000129071 | MBD4 | 10.786812 | 11.37478 | 0.587968 | 9.70E-08 | 4.24E-06 |
| ENSG00000175324 | LSM1 | 8.7036665 | 9.291571 | 0.5879046 | 3.45E-05 | 0.0006768 |
| ENSG00000126804 | ZBTB1 | 11.002238 | 11.589596 | 0.5873576 | 0.0015241 | 0.0156204 |
| ENSG00000183255 | PTTG1IP | 12.727109 | 13.314045 | 0.5869362 | 3.15E-07 | 1.18E-05 |
| ENSG00000139323 | POC1B | 9.4565278 | 10.041996 | 0.5854686 | 1.17E-05 | 0.0002663 |
| ENSG00000017427 | IGF1 | 10.154793 | 5.2778079 | -4.876985 | 2.33E-09 | 1.51E-07 |
| ENSG00000162692 | VCAM1 | 5.9609652 | 1.1572713 | -4.803694 | 4.50E-12 | 5.35E-10 |
| ENSG00000172350 | ABCG4 | 6.1355111 | 1.3941705 | -4.741341 | 1.30E-13 | 2.08E-11 |
| ENSG00000172572 | PDE3A | 6.6184682 | 1.9953775 | -4.623091 | 3.45E-09 | 2.15E-07 |
| ENSG00000239961 | LILRA4 | 6.9200238 | 2.3222343 | -4.597789 | 0.0014057 | 0.0146191 |
| ENSG00000003096 | KLHL13 | 7.4133121 | 3.0069404 | -4.406372 | 9.97E-16 | 2.37E-13 |
| ENSG00000174500 | GCSAM | 4.6357504 | 0.2632382 | -4.372512 | 1.18E-08 | 6.45E-07 |
| ENSG00000122254 | HS3ST2 | 13.808147 | 9.6813079 | -4.126839 | 2.01E-06 | 5.87E-05 |
| ENSG00000069702 | TGFBR3 | 6.8471826 | 2.7939547 | -4.053228 | 2.85E-08 | 1.42E-06 |
| ENSG00000255819 | KLRC4-KLRK1 | 4.6397895 | 0.6232885 | -4.016501 | 2.69E-06 | 7.55E-05 |
| ENSG00000166949 | SMAD3 | 8.6177457 | 4.899776 | -3.71797 | 3.80E-28 | 5.06E-25 |
| ENSG00000159713 | TPPP3 | 4.0811902 | 0.4620924 | -3.619098 | 8.57E-09 | 4.82E-07 |
| ENSG00000101197 | BIRC7 | 5.8981267 | 2.2934512 | -3.604676 | 2.93E-06 | 8.15E-05 |
| ENSG00000120457 | KCNJ5 | 13.137342 | 9.6001896 | -3.537152 | 2.00E-18 | 6.66E-16 |
| ENSG00000213809 | KLRK1 | 5.0002759 | 1.469479 | -3.530797 | 0.0002766 | 0.0039133 |
| ENSG00000120471 | TP53AIP1 | 4.8388121 | 1.3122606 | -3.526552 | 1.07E-08 | 5.93E-07 |
[truncated: 177,256 more chars]
